# Why do people use exotic plants in their local medical systems? A systematic review based on Brazilian local communities

**DOI:** 10.1371/journal.pone.0185358

**Published:** 2017-09-27

**Authors:** Patrícia Muniz de Medeiros, Washington Soares Ferreira Júnior, Marcelo Alves Ramos, Taline Cristina da Silva, Ana Haydée Ladio, Ulysses Paulino Albuquerque

**Affiliations:** 1 Laboratory of Biocultural Ecology, Conservation and Evolution (LECEB). Universidade Federal de Alagoas, Alagoas, Brazil; 2 Universidade de Pernambuco, Campus Petrolina, Pernambuco, Brazil; 3 Universidade de Pernambuco, Campus Mata Norte, Pernambuco, Brazil; 4 Universidade Estadual de Alagoas, Campus Santana do Ipanema, Alagoas, Brazil; 5 Laboratorio Ecotono, Instituto de Investigaciones en Biodiversidad y Medio Ambiente (INIBIOMA), Consejo Nacional de Investigaciones Científicas y Técnicas (CONICET-Universidad Nacional del Comahue), San Carlos de Bariloche, Argentina; 6 Laboratory of Ecology and Evolution of Social-ecological Systems (LEA). Departamento de Botânica, Centro de Biociências. Universidade Federal de Pernambuco, Pernambuco, Brazil; Oklahoma State University, UNITED STATES

## Abstract

Efforts have been made to understand the processes that lead to the introduction of exotic species into local pharmacopoeias. Among those efforts, the diversification hypothesis predicts that exotic plants are introduced in local medical systems to amplify the repertoire of knowledge related to the treatment of diseases, filling blanks that were not occupied by native species. Based on such hypothesis, this study aimed to contribute to this discussion using the context of local Brazilian populations. We performed a systematic review of Brazilian studies up to 2011 involving medicinal plants, excluding those studies that presented a high risk of bias (because of sampling or plant identification problems). An analysis of similarities (ANOSIM) was conducted in different scales to test for differences in the repertoire of therapeutic indications treated using native and exotic species. We have found that although there is some overlap between native and exotic plants regarding their therapeutic indications and the body systems (BSs) that they treat, there are clear gaps present, that is, there are therapeutic indications and BSs treated that are exclusive to exotic species. This scenario enables the postulation of two alternative unfoldings of the diversification hypothesis, namely, (1) exotic species are initially introduced to fill gaps and undergo subsequent expansion of their use for medical purposes already addressed using native species and (2) exotic species are initially introduced to address problems already addressed using native species to diversify the repertoire of medicinal plants and to increase the resilience of medical systems. The reasons why exotic species may have a competitive advantage over the native ones, the implications of the introduction of exotic species for the resilience of medical systems, and the contexts in which autochthonous plants can gain strength to remain in pharmacopoeias are also discussed.

## Introduction

In ethnobiology, an interesting model to study the evolutionary dynamics of cultural systems is the process of incorporation of exotic plants into local medical systems. These systems are cultural systems formed by a set of concepts and practices related to health and disease, including interpretations and symptoms of diseases recognized by a human group and the strategies and alternatives for treatment as well as the evaluation of therapeutic results [1,2]. The importance of exotic species for those medicinal systems is well known and is supported by their chemical efficiency and ease of acquisition [3]. The rapid spread of allochthonous species into traditional medical systems has generated concerns based on associations of this phenomenon with the loss or erosion of knowledge. These interpretations should be viewed with caution because we often ignore that knowledge systems are dynamic and contain a strong adaptive component [4].

Recent studies have provided new insights into the popularization of exotic species, linking the use of such species to adaptive strategies. Palmer [5] noted the occurrence of a loss in the number of native species used in the local pharmacopoeia and an increase in the number of introduced plants in a study conducted in Hawaii. The author attributed those changes to an adaptive behavior in response to ecological and cultural changes. The adaptive and dynamic nature of traditional knowledge associated with a decrease in isolation and increase in communication between communities ultimately spurs changes. The key question, however, lies in how to interpret those changes. Would those changes be negative for the communities or an adjustment that expands the ability of people to interact with the environment? Does the widespread use of exotic species contribute to the resilience of local medical systems [6]?

One way to contribute to that discussion is to examine the reasons why exotic species are included in local pharmacopoeias. Accordingly, Bennett and Prance [7] proposed a hypothesis based on plant versatility, whereby exotic species would be introduced into the communities for purposes other than medical, and, a posteriori, the species’ medicinal uses would be assessed and disseminated. This process means that species that are more versatile would have a greater likelihood of being included in local pharmacopoeias.

Albuquerque [8] proposed the hypothesis of diversification to try to understand the process of inclusion of exotic plants in pharmacopoeias. According to his hypothesis, exotic species would enter into local pharmacopoeias to fill the gaps not met by native plants and to diversify the local repertoire of medicinal plants. Indeed, Alencar *et al*. [9] found different classes of chemical compounds in exotic species compared with those predominant in native species. However, no overlap between diseases treated by native and exotic species would be theoretically expected if exotic species merely acted as diversifiers.

Therefore, this study aimed to broaden the discussion of the role of exotic species in local pharmacopoeias, taking into consideration the context of Brazilian local communities. We propose the following questions: (1) Is there a tendency of exotic species to be used for the treatment of a set of different diseases than those treated by native species in the different Brazilian ecosystems? (2) Do exotic species occupy gaps in pharmacopoeias that are not filled by native species? The answer to these questions may help in eliciting the advantages and disadvantages of the incorporation of exotic species in local medical systems, which can guide public policies regarding the valorization of native species and the maintenance of local traditions.

To answer these questions, a study was performed guided by a systematic review, focusing on three levels of spatial scale: national, regional, and local. From the present research questions, this study also discusses the role of exotic plants in the resilience of medical systems based on the use of medicinal plants.

## Methods

### Data collection

This study was conducted through a systematic literature review, following the methodological rigor commonly applied for meta-analytical studies (S1 Checklist). Our protocol for this systematic review has not been registered, although previous studies of our group have already employed it [10–12]. We searched for ethnobotanical and ethnopharmacological articles concerning medicinal plant use in local Brazilian communities. The study could be only regarding medicinal plants or a general ethnobotanical study with information about medicinal plants (in this case, we considered only data for this purpose). The studies would have to present a species list, together with their therapeutic indications. Bibliographic search considered only articles published up to 2011 (when our literature search was finished) and we did not consider books, book chapters, and technical reports; we considered studies published in English and Portuguese for this systematic review.

We used the keywords “Medicinal plants + Brazil,” “Plantas medicinais + Brasil”, “Ethnobotany + Brazil,” “Etnobotânica + Brasil”, “Ethnopharmacology + Brazil,” and “Etnofarmacologia + Brasil” in the major databases and publishers of scientific journals (Scopus, Scirus, and Scielo). We are aware that other databases could have been used in addition to the chosen ones. However, we opted to access those which commonly return more ethnobotanical literature and (specifically for Scielo) the most representative to capture studies developed in Brazil.

The search was performed from November 2010 to June 2011. For each paper found, its references were analyzed with the aim of identifying additional studies that were not uncovered using the established search criteria. In studies that were unavailable online, we accessed them by visiting the libraries of “Universidade Federal de Pernambuco,” “Universidade Federal Rural de Pernambuco,” and “Universidade Federal de Brasília.”

**Fig 1:**
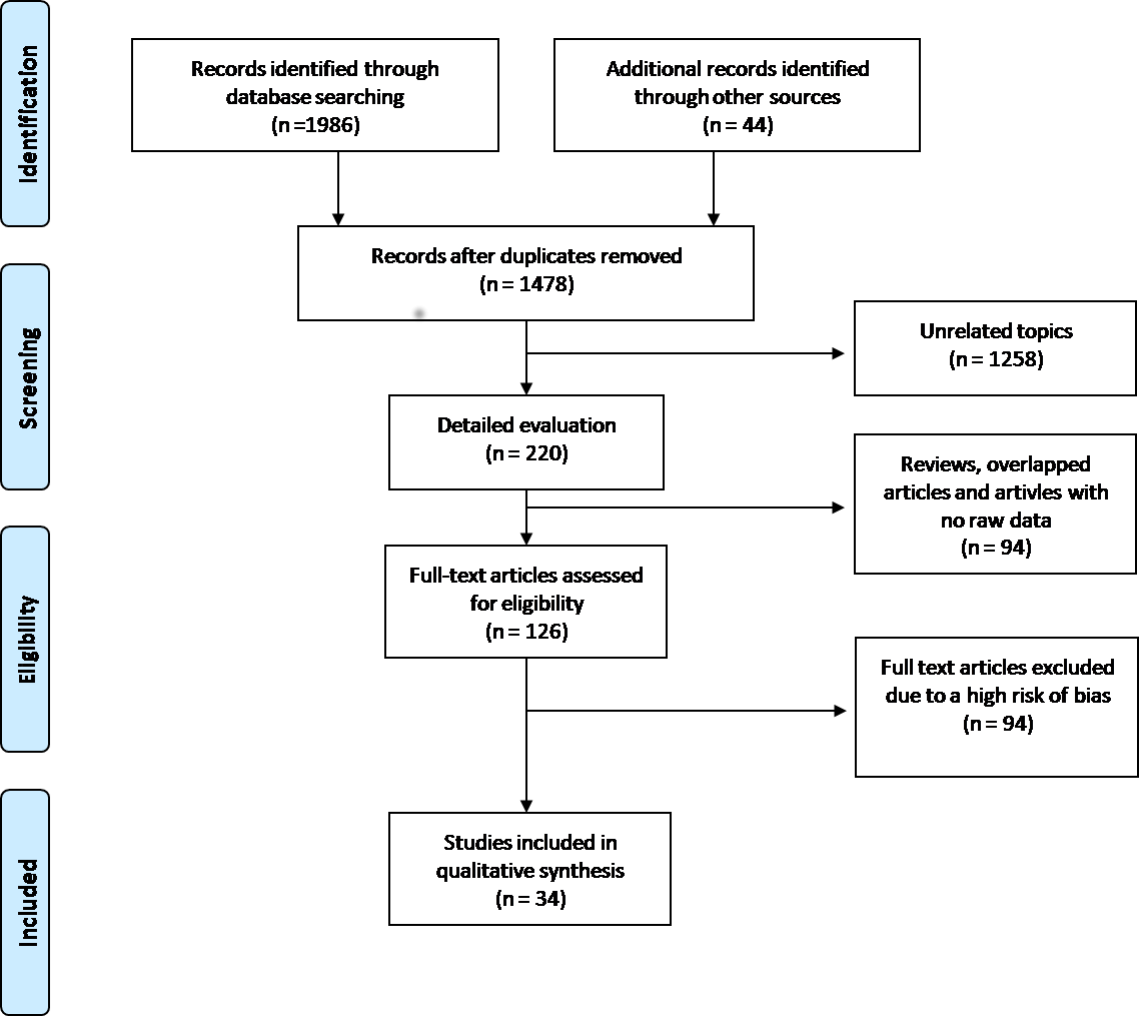
Flow diagram with the number of selected studies.

A total of 126 articles matched our inclusion criteria and were classified according to their risk of bias, into “low risk”, “moderate risk” and “high risk” (S1 File). Aiming to select only articles with more rigorous study designs, we included in this systematic review only the studies with low or moderate risk of bias, resulting in 34 studies [13–46] (Fig 1).

The scientific names of plant species were reviewed using the databases of the Brazilian Flora Checklist (http://floradobrasil.jbrj.gov.br/2012) and the Missouri Botanical Garden (www.tropicos.org). These databases were also used to classify the species, based on their origin, into native or exotic Brazilian species and native or exotic species from the ecosystem in which each study was inserted. The APG III classification system was used [47]. We could not control the quality of the plant material identification performed in the selected studies and this inability may be considered as a limitation of this systematic review. However, our criteria for classifying studies according to their risk of bias also considered issues related to specimen identification and herbarium deposition [10–12].

The therapeutic indications (TIs) were classified into the following 19 body systems (BSs) according to the World Health Organization [48]: Certain infectious and parasitic diseases; diseases of the genitourinary system; injury, poisoning, and certain other consequences of external causes; diseases of the respiratory system; diseases of the circulatory system; diseases of the musculoskeletal system and connective tissue; diseases of the digestive system; mental and behavioral disorders; endocrine, nutritional, and metabolic diseases; diseases of the blood and blood-forming organs and certain disorders involving the immune mechanism (DBI); diseases of the skin and subcutaneous tissue; pregnancy, childbirth, and the puerperium; factors influencing health status and contact with health services (FHS); diseases of the eye and adnexa (DEA); diseases of the ear and mastoid process (DEMP); certain conditions originating in the perinatal period (CPP); neoplasms (NEO); diseases of the nervous system; and unspecified symptoms and signs (USS). The indications regarding “cultural diseases” were not considered [49].

### Data analysis

This systematic review evaluated the role of exotic species in three levels of spatial analysis: national, regional, and local. We chose to consider different scales because (1) the application of “exotic species” concept may vary (plants are sometimes characterized as native to an ecosystem, a region, a country, or a continent) and we wanted to theoretically account for such variations; (2) we wanted to check if patterns of exotic species’ entrance into traditional pharmacopoeias would vary according to the scale that is being evaluated.

Data from all 34 studies (Table 1) were pooled for the national analysis to generate a database containing the species, their origin (native or exotic to Brazil), their TIs, and the BSs that they treat.

**Table 1:** Studies with low and moderate risks of bias included in this systematic review of medicinal plant use in Brazilian local communities.

Reference	Ecosystem	Area	Risk of bias
Albertasse et al. [13]	Atlantic Forest	Urban	Moderate
Albuquerque e Andrade [14]	Caatinga	Rural	Low
Almeida et al. [15]	Atlantic Forest	Urban	Moderate
Andrade et al.[16]	Caatinga	Rural	Moderate
Baldauf et al. [17]	Pampa	Urban and Rural	Moderate
Begossi et al. [18]	Atlantic Forest	No information	Moderate
Brandão et al. [19]	Amazon	Urban and Rural	Moderate
Brandão et al. [20]	Cerrado and Atlantic Forest	Urban	Low
Cartaxo et al. [21]	Caatinga	Rural	Moderate
Cruz-Silva et al. [22]	Atlantic Forest	Urban	Low
Dorigoni et al. [23]	Atlantic Forest	Urban	Moderate
Franco and Barros [24]	Cerrado and Mata dos cocais	Rural	Low
Freitas and Fernandes [25]	Amazon	Rural	Low
Garlet and Irgang [26]	Pampa and Atlantic Forest	Rural	Moderate
Hanazaki et al. [27]	Atlantic Forest	Urban	Low
Lima et al. [28]	Atlantic Forest	Urban	Moderate
Lima et al. [29]	Atlantic Forest	Urban	Low
Macedo et al. [30]	Cerrado and Atlantic Forest	Urban	Low
Maciel and Guarim-Neto [31]	Amazon	Urban and Rural	Moderate
Merétika et al. [32]	Atlantic Forest	Urban	Moderate
Moreira and Guarim-Neto [33]	Cerrado	Rural	Moderate
Negrelle and Fornazzari [34]	Atlantic Forest	Rural	Low
Oliveira et al. [35]	Caatinga	Rural	Low
Oliveira et al. [36]	Atlantic Forest	Urban	Moderate
Pereira et al. [37]	Cerrado and Atlantic Forest	Urban	Moderate
Pilla et al. [38]	Cerrado and Atlantic Forest	Rural	Low
Ritter et al. [39]	Atlantic Forest	Urban and Rural	Moderate
Rizzo et al. [40]	Cerrado	Urban	Moderate
Roque et al. [41]	Caatinga	Rural	Low
Silva et al. [42]	Caatinga	Rural	Low
Souza [43]	Cerrado	Urban	Moderate
Ustulin et al. [44]	Cerrado	Urban	Low
Vendruscolo and Mentz [45]	Pampa	Urban	Moderate
Vila Verde et al. [46]	Cerrado	Urban	Moderate

The regional analysis considered each individual ecosystem so that each ecosystem encompassed part of the studies from the national analysis. In this case, the classification of native and exotic species used the ecosystems as a reference so that species were classified as native or exotic to the ecosystem in question, rather than to Brazil in its entirety. Six studies that were considered in the national analysis [20, 24, 26, 30, 37, 38] were excluded from the regional analysis because they were research studies conducted in ecotonal areas (for example, Atlantic Forest and Cerrado) (see Table 1). The following ecosystems were included: Atlantic Forest *lato sensu* (12 studies), Amazon (Tropical forest) (3), Caatinga (Seasonal dry forest) (6), Cerrado (Neotropical Savana) (5), and Pampa (Grassland) (2). A search based on the Brazilian Institute of Geography and Statistics (IBGE; http://www.ibge.gov.br/cidadesat) was conducted for studies that failed to mention the ecosystem.

Only six case studies were considered in the local analysis [14, 21, 31, 34, 39, 42], which were chosen from the 34 used in the national analysis, and were conducted in rural and urban-rural areas that were non-ecotonal. Their study design encompassed all TIs, origins, and habits. Therefore, these studies were considered quite relevant for local analysis. The classification of native and exotic species in this case also considered the ecosystem in which each study was conducted.

To answer our first question, multivariate binary data matrices were generated for the total dataset (national analysis) and for each ecosystem (regional analysis) and each selected study (local analysis), with the species as objects and the TIs as descriptors. An analysis of similarities (ANOSIM) was used to test for differences in the repertoire of TIs treated using native and exotic species. In a second step, ANOSIM was performed using the BSs as descriptors. This test compares similarities (in our case, Jaccard) within groups (native and exotic) with similarities between groups. When similarities within groups are higher than similarities between groups, p-values tent to be less than 0.05 and groups are considered to be distinct. Therefore, we consider that when similarities within groups are lower or equal to the similarities between groups there is a high overlap between those groups.

Those analyses were conducted using the packages Rcmdr and Vegan of the statistical software R, version 2.13.2 (The R Foundation for Statistical Computing), considering α = 0.05. For those analyses, “unspecified” TIs and USS BSs were not part of the data matrix.

In order to answer our second question, the number of native and exotic species corresponding with each therapeutic indication and BS was recorded for both the national analysis and the regional and local analyses. The gaps (TIs without native species, only treated using exotic species) and semi-gaps (TIs treated only using one native species and one or more exotic species) were determined based on that information.

The raw data of this systematic review is presented in the supplementary material (S1 Table).

## Results

### Therapeutic differences between native and exotic species

Table 1 exhibits crucial information regarding the studies that were part of this systematic review.

In the national analysis we did not find differences between native and exotic species regarding TIs (R = –0.008; p > 0.05) or regarding the BSs treated by the species (R = –0.002; p > 0.05). This observation indicates a strong overlap of native and exotic species for treating the same diseases and BSs.

The results of the regional analysis (evaluation of ecosystems) showed that the Caatinga zone exhibited the greatest differentiation of native species from exotic species regarding the target BSs (R = 0.03, p < 0.05) and TIs (R = 0.05; p < 0.001). However, the low R values show there was still a high overlap in the treatment of diseases by native and exotic species, despite the significance of the results. There were differences between native and exotic species regarding the set of BSs treated by the species (R = 0.01, p < 0.05) in the Atlantic Forest, whereas such differences were not observed considering the TIs (R = 0.005; p > 0.05). The remaining ecosystems (Cerrado, Amazon, and Pampa) indicated a strong overlap of native and exotic species regarding TIs and the BSs that the species were used to treat (p > 0.05).

The local analyses obtained results ranging from a slight difference between native and exotic species to a strong overlap regarding the origin. The analysis based on the study performed by Maciel and Guarim-Neto [31] in the Amazon region showed differences between the origins regarding the TIs (R = 0.07; p < 0.001) and the target BSs (R = 0.096; p < 0.001). Analysis for Albuquerque and Andrade [15] and Cartaxo *et al*. [21], both in the Caatinga, showed statistically significant differences between native and exotic species only regarding the TIs (R = 0.16; p < 0.01 and R = 0.03; p < 0.05, respectively). The other three studies of the local analysis showed no differences between the origins (p > 0.05). Two of them were developed in Atlantic Forest areas [34, 39] and one in the Caatinga [42].

At first glance, some of the results found herein could indicate a weakness in the diversification hypothesis because exotic species would be expected to treat different diseases from those treated by native species if the exotic species are included into local pharmacopoeias to fill gaps. However, the presence of gaps in pharmacopoeias must be assessed; that is, whether there are TIs or BSs exclusively treated with exotic species, as shown in the following sections.

### Identifying gaps

The number of gaps in the pharmacopoeias according to the national analysis and the regional and local analyses are shown in Table 2 (gaps of BSs) and Table 3 (gaps of TIs). In general, all of the analyses identified some type of gap, and the number of gaps increased from the national analysis to the regional and local analyses. No native species noticeably occurred for the system “certain conditions originating in the perinatal period” (CPP), with four exotic species for the treatment of these disorders in the overall analysis of BSs. Eighty-three gaps and 46 semi-gaps were evidenced among the TIs.

**Table 2:** List of BSs without native species (gaps) or treated using only one native species (semi-gaps) in general, regional (by ecosystem) and local (by study) analyses in Brazilian ethnobotanical studies. Further detail is given according to gaps and semi-gaps with 1–3 exotic species, 4–6 exotic species and more than six exotic species.

Focus	Gaps >6	Gaps 4–6	Gaps 1–3	Semi-gaps >6	Semi-gaps 4–6	Semi-gaps 1–3
**General**	0	1	0	0	0	0
**Regional**						
Amazon	0	0	3	0	1	2
Caatinga	0	0	0	0	0	3
Cerrado	0	0	0	0	0	1
Atlantic Forest	0	0	0	1	0	0
Pampas	1	1	3	1	0	1
**Local**						
Albuquerque and Andrade [14]	0	0	2	0	0	1
Cartaxo et al. [21]	0	0	3	0	0	2
Maciel and Guarim-Neto [31]	1	0	7	0	2	3
Negrelle and Fornazzari [34]	2	1	2	0	1	2
Ritter et al. [39]	0	0	4	1	0	0
Silva et al. [42]	0	0	1	0	0	3

**Table 3:** List of TIs without native species (gaps) or treated using only one native species (semi-gaps) in general, regional (by ecosystem) and local (by study) analyses in Brazilian ethnobotanical studies. Further detail is given according to gaps and semi-gaps with 1–3 exotic species, 4–6 exotic species and more than six exotic species.

Focus	Gaps >6	Gaps 4–6	Gaps 1–3	Semi-gaps >6	Semi-gaps 4–6	Semi-gaps 1–3
**General**	0	5	76	2	7	38
**Regional**						
Amazon	0	2	32	0	0	6
Caatinga	1	0	25	0	2	12
Cerrado	0	0	26	1	0	22
Atlantic Forest	2	4	57	3	6	27
Pampas	2	15	77	7	3	11
**Local**						
Albuquerque and Andrade [14]	0	0	7	0	0	2
Cartaxo et al. [21]	0	1	2	2	3	11
Maciel and Guarim-Neto [33]	1	3	24	0	0	4
Negrelle and Fornazzari [34]	2	3	15	0	1	8
Ritter et al. [39]	0	3	37	0	1	17
Silva et al. [42]	0	0	18	0	1	13

The use of exotic species for diseases not treated by native species could be interpreted as a mere sampling artifact, because most of the gaps (67%) are occupied by a single exotic species. However, there are gaps and semi-gaps of native species in some cases that are filled by 4, 5, 6 or more exotic species, which strengthens the notion that exotic species were actually included in the pharmacopoeias to fill gaps.

In the regional analyses, the BSs that were considered as gaps or semi-gaps most often were “diseases of the ear and mastoid process” (DEMP; in four out of five ecosystems) and “diseases of the eye and adnexa” (DEA; in three ecosystems), which indicates that these systems have deficiencies in the number of native species that treat them at the regional level. The therapeutic indication with the greatest absence of native plants was “dandruff” (absent or with only one representative in four ecosystems). Indications such as “labyrinthitis,” “high blood pressure,” “migraine,” “sedative,” and 13 others were considered gaps or semi-gaps in the three ecosystems.

From the local standpoint, the systems that most often showed an absence or only one occurrence of native species (four out of six studies) were the “neoplasms” (NEO) and “factors influencing health status and contact with health services” (FHS), the latter consisting of TIs such as “stress,” “aphrodisiac,” and others. The indications most frequently considered as gaps or semi-gaps were “fever” and “sedative” (4 studies). TIs such as cancer and high blood pressure, in addition to eight others, constituted gaps and semi-gaps in three studies.

## Discussion

### Hypotheses explaining the inclusion of exotic species in pharmacopoeias

The results do not support a clear division between the contribution of native and exotic species regarding the repertoire of diseases treated by medicinal plants. This occurs primarily when we analyze the data on a national scale. Such results could be explained because the diseases treated by native species in one environment may be treated by exotic species in another, for reasons of preferred biochemical pathways [50] or even cultural aspects, although there is a tendency for using native species to treat a set of diseases different from that addressed by the exotic species in local pharmacopoeias. Therefore, the combination of different environments in the national analysis may have overlapping native and exotic species used for treating diseases, which apparently affected the result.

The lower overlap between native and exotic species show that the consideration of more than one level of spatial analysis may show different responses, as they are differently sensible to the influence of cultural, ecological, and evolutionary processes that make people–nature relationships heterogeneous.

The presence of gaps in pharmacopoeias is quite noticeable. Based on these findings, we suggest a new perspective on the diversification hypothesis based on two parallel unfoldings.

1. Exotic species as fillers of gaps: exotic species would actually be included into pharmacopoeias to fill gaps not filled by native species, as suggested by the diversification hypothesis [8]. However, the spread of these allochthonous species upon their introduction could occur to occupy spaces already taken by native species. If the data from this study are interpreted according to this hypothesis, the gaps identified would be the entryway of the exotic species, and subsequently, the species’ use would be extrapolated to treat other diseases that are already treated using native species, which could explain the overlap in the use of native and exotic plants.
2. Introduction of exotic species overlapping the use of native species: exotic plants would be introduced into local pharmacopoeias not to fill gaps but to expand the repertoire of plants, leading to utilitarian redundancy idea [51]. In this case, the overlap in the use of native and exotic species could be explained by the introduction of exotic species to treat conditions already treated by native species.

Considering the different subdivisions of the diversification hypothesis, the presence of gaps could be interpreted as (a) a natural absence of native plants used to treat conditions, especially those negligible or new diseases, which would not require the previous presence of these autochthonous species to treat them; (b) a sampling artifact because people do not always remember to convey all information relating to the use of plants; or (c) the loss of knowledge regarding the use of native plants to treat certain diseases, derived from the significant use of exotic species and interruption in the transmission of knowledge regarding autochthonous plants.

Thus, would the introduction of exotic plants into spaces previously occupied by native plants involve a process of acculturation or erosion of traditional knowledge? To answer that question, we assume that traditional knowledge has an adaptive nature [52], which is guided by the perpetuation of traits responsible for a greater adaptability and for more effectively meeting the local needs. Thus, exotic species might often provide a greater adaptive advantage considering the costs and benefits of their use.

Studies have already suggested that exotic species have the advantage of greater palatability [8, 53], which could increase the benefit of their consumption. Furthermore, considering that exotic species occupy areas that are more easily accessible [3], including backyards and roadsides, among other areas, the costs associated with the collection of such species would be lower. Thus, the popularization of exotic species in local pharmacopoeias may be interpreted as a behavioral adaptation to ecological and cultural changes, as suggested by Palmer [4], rather than interpreted, without detail or further questioning, as loss and erosion of knowledge.

### The role of exotic plants in the resilience of local medical systems

The above-stated subdivision of the diversification hypothesis may be interpreted from a perspective of the resilience of medical systems. Systems in which many species perform the same functions tend to be more resilient because the loss of a species in the system resulting from external disturbances would not affect the functions of the system [54–57]. Considering the local medical systems, these disturbances may be of various types, including environmental or anthropic, and may lead to the local extinction of some medicinal species or changes in the mechanisms of cultural transmission that interrupt the communication of information on the use of particular species.

From the perspective of resilience, the introduction of exotic plants into categories already occupied by native species, whether *a priori* or *a posteriori*, may be connected to an adaptive strategy of local communities to favor the resilience of their medical systems, increasing the utilitarian redundancy of local medicinal categories. In that case, with a more redundant category, people will have more alternatives when facing different events of the same disease or even when facing external disturbances to the system that compromise some of the species. This redundancy allows a greater flexibility of the medical system and, therefore, of resilience [51, 56]. However, some peculiarities should be considered when analyzing the introduction of exotic species into categories already occupied by native species, which are listed below.

#### Reservoir of resilience

Walker et al. [58] advocated the existence of species in an ecological system that “drive” the system and are responsible for the ecosystem function and of “passenger” species that are “reservoirs of resilience” for the system. The “passenger” species would maintain the system’s operation under some disturbance that affected the dominant species [58]. Accordingly, those exotic species may either maintain the active use of plants in medicinal categories within the system or represent a “reservoir of resilience” for some categories.

#### Transmission of knowledge

If the exotic species are part of the “reservoir of resilience” and there is a tendency to convey only the knowledge about species that drive the system, the “reservoir” exotic species are expected to have no function in the system in the next generations because their use will be forgotten. Similarly, the introduction of exotic plants may also move knowledge of native species to the stock knowledge (this concept is introduced in Albuquerque [8]). Stock knowledge may be considered fragile because it is not integrated in people’s daily lives as significantly as the former.

Therefore, the efficiency of transmission of stock knowledge will clearly define the permanence of specific plants in medical systems. A greater effectiveness of this transmission would ensure a resilient system, with different alternatives of use, while breaks in that transmission would reduce such resilience. In this last case, the conditions for persistence of autochthonous resources of medicinal use would be quite unfavorable if native species were moved to the stock knowledge and exotic plants occupied the mass knowledge.

### Competitive advantage of native species

There is common concern regarding the future of the native species in local medical systems, even considering the popularization of exotic species as something that is part of the “natural evolution” of pharmacopoeias. For how long will native species endure in local pharmacopoeias if exotic species have a greater optimization of the cost/benefit ratio? A key issue is that the cost/benefit ratio does not always favor exotic plants. Some examples of situations that may increase the competitive advantage of native species will be discussed below.

#### Therapeutic efficacy

Some native plants may be so efficient in curing some diseases that the advantage of their consumption would offset the costs of obtaining such resource. Different research studies conducted in local communities in the Brazilian northeast region have found that although a high number of exotic species is associated with local pharmacopoeias [8, 51], native species are indicated by those communities as preferred for the treatment of different diseases, especially considering the local perception of their therapeutic efficacy [59]. For example, Albuquerque and Oliveira [51] found that three native species (*Amburana cearensis* (Allemão) A.C. Sm., *Anacardium occidentale* L. and *Myracrodruon urundeuva* Allemão) were locally the most important. Accordingly, the exotic species analyzed in the research studies above would tend to occupy the function of “reservoir of resilience” in those systems.

#### Exclusivity of use

Some TIs are exclusive to native species. In these cases, the lack of exotic species in treating specific diseases would ensure the permanence of native plants in traditional medical systems. For the data of the present study, in the national analysis, these indications include “syphilis,”–treated by *Bowdichia virgilioides* Kunth, *Cereus jamacaru* DC., *Croton antisyphiliticus* Mart., *Maclura tinctoria* (L.) D.Don ex Steud. and *Rudgea viburnoides* (Cham.) Benth.–“Chagas disease,”–treated by *Echinodorus grandiflorus* (Cham. & Schltr.) Micheli–and “back pain,”–treated by *Ageratum conyzoides* L., *Varronia curassavica* Jacq., *Anadenanthera colubrina* (Vell.) Brenan and *Solidago chilensis* Meyen–among others.

#### Medicinal versatility

Native species used to treat a wide range of diseases would remain in pharmacopoeias because their multiple uses would favor their permanence in the medical system. Many studies developed in the Brazilian Caatinga, for example, have shown that native species are more versatile than exotics (especially *M*. *urundeuva*, which commonly outstands in many Brazilian ethnobotanical studies [11, 60]

#### Symbolic aspects

Some species may have a cultural importance so great that the “benefit” of their use would be associated not only with chemical efficiency but also with a “cultural reward.” The cultural keystone species [61] are interesting examples of the importance of symbolic power in the use of plant species. The use of those species may be perpetuated even in contexts of intercontinental migrations, in which some species continue to be used despite difficulties in their acquisition [62].

#### Access to native vegetation areas

The system of use of plant resources by local populations is complex and multivariate [52, 63]. Therefore, the cost–benefit ratios in the acquisition of native and exotic species may not consider the dynamics of the use of medicinal plants as a separate entity from the use of other plant resources. Accordingly, communities that still have ties to the native vegetation areas, to collect firewood and fruits and to perform agricultural activities, among others, will tend to more often use native species for medicinal purposes. This use would occur because the cost of obtaining autochthonous medicinal plants would be relativized by the fact that people already enter native vegetation areas for other purposes, and the people may conveniently collect native medicinal resources.

## Limitations and prospects

This study established rigorous criteria for article selection and such choice has positive and negative implications. We have opted to discard possibly biased data but it also results in lost information. Therefore, our systematic review is far from representing all Brazilian medicinal flora and it may have limited its predictive power. However, we judged that such loss would be less problematic than the inclusion studies with a high risk of bias.

Our finding reinforces that studies on socioecological systems should consider different spatial scales since they can elicit different patterns. Finally, the fact that we did not find differential “niches” for native and exotic species may not have directly supported the diversification hypothesis, but future studies are needed to evaluate if (and when) the gaps (indications with no exotic species) are primary (following the diversification hypothesis) or secondary (indicating competitive exclusion). To achieve such answer we recommend long-term studies that try to “follow the track” of recent medicinal plant introductions.

## Supporting information

S1 Checklist Prisma Checklist for this systematic review. (DOC)

S1 File Criteria to define the study risk of bias (extracted from Medeiros et al. [11, 12]) for this meta-analysis on the role of exotic medicinal plants in local Brazilian communities. (DOC)

S1 Table Raw data for the systematic review on medicinal plant use by Brazilian local populations. H: Herb; S: Shrub; T: Tree; N: Native; E: Exotic; CIPD: Certain infectious and parasitic diseases, PCP: Pregnancy, childbirth and the puerperium; DEA: Diseases of the eye and adnexa; DEMP: Diseases of the ear and mastoid process; DSST: Diseases of the skin and subcutaneous tissue; DCS: Diseases of the circulatory system; DDS: Diseases of the digestive system; DGS: Diseases of the genitourinary system; DBBO: Diseases of the blood and blood-forming organs; DNS: Diseases of the nervous system; DMS: Diseases of the musculoskeletal system and connective tissue; DRS: Diseases of the respiratory system; IPEC: Injury, poisoning and certain other consequences of external causes; ENMD: Endocrine, nutritional and metabolic diseases; COP: Certain conditions originating in the perinatal period; MBD: Mental and behavioural disorders; NEO: Neoplasms; SSNEC: Symptoms, signs and abnormal clinical and laboratory findings, not elsewhere classified.
(DOCX)
