# Supplementary material for: Why do people use exotic plants in their local medical systems? A systematic review based on Brazilian local communities
Source: PLoS One. 2017 Sep 27;12(9):e0185358. doi: 10.1371/journal.pone.0185358 (PMC5617200; doi:10.1371/journal.pone.0185358)
Supplement: S1 File — (DOC) [file pone.0185358.s002.doc]

**S1 File.** Criteria to define the study risk of bias (extracted from Medeiros et al. [11, 12]) for this meta-analysis on the role of exotic medicinal plants in local Brazilian communities.

1. ***General procedure***

Studies acquired with the bibliographic search that met the initial inclusion criteria were classified according to the risk of bias as ‘low risk’, ‘moderate risk’ and ‘high risk’. The reliability of the sample was the main criterion, with other criteria used to assess a possible increase in the degree of risk, as detailed below.

1. Classification criteria for the three degrees of risk of bias

- Sample reliability—high risk of bias for studies with serious sample problems, moderate risk of bias for studies with small sample problems and low risk of bias for studies without sampling problems or whose problems were not evident. Common sampling problems are (1) non representativeness of samples based on the whole community or on family chiefs; (2) lack of indication about the population (universe) and (3) lack of information about the criteria adopted in cases of intentional samples.
- Identification of the plant material—high risk of bias when less than 60% of the taxa were identified to species level, moderate risk of bias when 60% to 80% of the taxa were identified and low risk of bias when more than 80% of the taxa were identified to the species level.

If a study had two distinct classifications (e.g. low risk of bias when analyzing sampling problems and moderate risk of bias when analyzing identification of the plant material) the most restrictive classification would prevail (in this case, a moderate risk of bias).

(B) Criteria for an increase in risk level (from low to moderate risk of bias)

- Lack of specification that the material was identified by comparing voucher specimens or consulting experts.
- Presentation of a partial list of the species used (e.g., only the 20 most cited). Investigations that presented complete lists of plants or plants cited by at least 80% of the respondents were still considered to have a low risk of bias.
- Presence of restrictions of the studied habit, distribution, therapeutic indications or taxonomic groups. For example, studies with only herbs, forest species for malaria or studies with only Cactaceae.

Studies with low and moderate risks of bias were selected for the analyses, whereas studies with a high risk of bias were completely disregarded.

1. ***Detailed procedure for analyzing risk of bias based on sampling reliability***

1) When sample is extracted from the total number of people or from an age

interval

LOW

a) When the sample size (N) reaches the universe (U);

b) When N is representative of U, with sample randomness and considering a margin of error of up to 5%;

c) When N is at least 80% of U, considering that some respondents may refuse to participate of interviews or may not be in their household even after successive trials.

MODERATE

a) When N is extracted from U, with sample randomness and a margin of error higher than 5% and lower than 10%;

b) When N is at least 80% of the value which is needed for representativeness, considering a margin of error of up to 5%;

c) When N could be considered to be representative of U (with a margin of error of up to 10%) if only the numbers are considered, but in situations which sample is occasional or when there is no specificity about randomness.

HIGH

a) When N is extracted from U with a margin of error higher than 10%;

b) When N is less than 80% of the value which is necessary for representativeness, considering a margin of error of up to 5%;

c) When there is no information about the universe (U), or when there is no

information about the sample (N).

2) When sample is based on the family heads (one or two per household)

LOW

a) When all family heads were interviewed;

b) When a representative number of family heads was interviewed, with sample randomness and a margin of error up to 5%;

c) When N is at least 80% of the family heads.

MODERATE

a) When N is extracted from the universe (U) of family chiefs, with sample randomness and a margin of error higher than 5% and lower than 10%;

b) When N is at least 80% of the value which is necessary for the representativeness of the family heads, considering a margin of error of up to 5%.

c) When N could be considered to be representative of the number of family heads (with a margin of error of up to 10%), if only the numbers are considered, but in situations which sample is occasional or when there is no specificity about randomness.

HIGH

a) When N is extracted from the universe (U) of family heads with a margin of error of up to 10%;

b) When N is less than 80% of the value which is needed for representativeness of the family chiefs, considering a margin of error of up to 5%;

c) When there is no information about the number of family heads, or when there is no information about the sample (N).

3) When sample is based on households

LOW

a) When one member of each household was interviewed;

b) When a representative number of households had one of its members interviewed, with sample randomness and a margin of error up to 5%;

c) When N is at least 80% of the households.

MODERATE

a) When N is extracted from the universe (U) of households, with sample randomness and a margin of error higher than 5% and lower than 10%;

b) When N is at least 80% of the value which is necessary for sample representativeness, considering a margin of error of 5%;

c) When N could be considered to be representative of the households (with a margin of error of up to 10%) if only the numbers are considered, but in situations which sample is occasional or when there is no specificity about randomness.

HIGH

a) When N is extracted from the universe (U) of households with a margin of error higher than 10%;

b) When N is less than 80% of the value which is necessary for household representativeness, considering a margin of error of up to 5%;

c) When there is no information about the number of households (U), or when there is no information about the sample (N).

4) When sample is intentional, focusing on a group of interest (e.g. midwives, healers, local specialists)

LOW

a) When sample corresponds to the totality of the specific group;

b) When sample is representative of the specific group, with sample randomness and a margin of error or up to 5%);

c) When sample is at least 80% of the specific group;

d) In cases of local specialists, when the snowball technique is used and there is an indication of the total number of dwellers;

e) In cases of local specialists, when they are selected based on clear and well established criteria.

MODERATE

a) When N is extracted from the universe (U) of the specific group, with sample randomness and a margin of error higher than 5% and lower than 10%;

b) When N is at least 80% of the value which is necessary of representativeness of the specific group, considering a margin of error of up to 5%;

c) When N could be considered to be representative of the specific group (with a margin of error of up to 10%) if only the numbers are considered, but in situations which sample is occasional or when there is no specificity about randomness;

d) In cases of local specialists, when there is no indication of the universe (U), but the snowball technique is applied to select the key informants.

HIGH

a) When N is extracted from the universe (U) of the specific group with a margin of error higher than 10%;

b) When N is less than 80% of the value which is necessary for representativeness of the specific group, considering a margin of error of up to 5%;

c) When there is no information about the specific group (U), or when there is no information about the sample (N), except for the use of the snowball technique, when there is no information about the U;

d) In cases of local specialists, when they are selected based on arbitrary or obscure criteria.

5) When rarefaction curves are used

LOW

a) When there is information about N and U and when the rarefaction curve gets stable, regardless of sample representativeness and the criteria for informant selection.

MODERATE

a) When there is no information about U, but the rarefaction curve gets stabilized;

b) When there is no information about N and U and when the rarefaction curve gets close to stabilization.

HIGH

a) When there is no information about N, regardless of the rarefaction curve behavior;

b) When the rarefaction curve gets far from stabilization;

c) When the study claims to have performed a rarefaction curve, but does not exhibit its result and does not affirm that there was stabilization.

6) When participatory methods are used

LOW

a) When the number of participants corresponds to a representative amount of the population or specific group (with a margin of error of up to 5%, but not considering the precepts of randomness, that mostly does not apply to participatory methods).

MODERATE

a) When the number of participants is not representative of the population or specific group;

b) When there is no information about the universe (population as a whole or specific group), but there is information about the number of participants.

HIGH

1. When there is no information about the number of participants;

7) Diffuse selection criteria

HIGH

a) When there is no information about N or U;

b) When there are various diffuse criteria for selecting the same sample.

1. **Prevalence criteria in situations when there is an overlap of classifications according to the risk of bias (only for sampling problems).**

| **Situation** | **Prevalence** | **Example** |
| --- | --- | --- |
| A study is classified by two or more routes and all of these routes lead to the same risk of bias. | All classifications are considered | 4–High–c e 4–High–d *Prevalence of a high risk* |
| Study with composite samples (for more than one place or more than one type of respondent). | More restrictive criterion | 1-Moderate-a e 1-High-a  *Prevalence of a high risk* |
| Studies with rarefaction curves | Criteria for rarefaction curve | 1-Moderate-a e 5-Low-a  *Prevalence of a low risk* |
