# Supplementary material for: Why do people use exotic plants in their local medical systems? A systematic review based on Brazilian local communities
Source: PLoS One. 2017 Sep 27;12(9):e0185358. doi: 10.1371/journal.pone.0185358 (PMC5617200; doi:10.1371/journal.pone.0185358)
Supplement: S1 Table — H: Herb; S: Shrub; T: Tree; N: Native; E: Exotic; CIPD: Certain infectious and parasitic diseases, PCP: Pregnancy, childbirth and the puerperium; DEA: Diseases of the eye and adnexa; DEMP: Diseases of the ear and mastoid process; DSST: Diseases of the skin and subcutaneous tissue; DCS: Diseases of the circulatory system; DDS: Diseases of the digestive system; DGS: Diseases of the genitourinary system; DBBO: Diseases of the blood and blood-forming organs; DNS: Diseases of the nervous system; DMS: Diseases of the musculoskeletal system and connective tissue; DRS: Diseases of the respiratory system; IPEC: Injury, poisoning and certain other consequences of external causes; ENMD: Endocrine, nutritional and metabolic diseases; COP: Certain conditions originating in the perinatal period; MBD: Mental and behavioural disorders; NEO: Neoplasms; SSNEC: Symptoms, signs and abnormal clinical and laboratory findings, not elsewhere classified. (DOCX) [file pone.0185358.s003.docx]

| **S1 Table.** Raw data for the systematic review on medicinal plant use by Brazilian local populations. H: Herb; S: Shrub; T: Tree; N: Native; E: Exotic; CIPD: Certain infectious and parasitic diseases, PCP: Pregnancy, childbirth and the puerperium; DEA: Diseases of the eye and adnexa; DEMP: Diseases of the ear and mastoid process; DSST: Diseases of the skin and subcutaneous tissue; DCS: Diseases of the circulatory system; DDS: Diseases of the digestive system; DGS: Diseases of the genitourinary system; DBBO: Diseases of the blood and blood-forming organs; DNS: Diseases of the nervous system; DMS: Diseases of the musculoskeletal system and connective tissue; DRS: Diseases of the respiratory system; IPEC: Injury, poisoning and certain other consequences of external causes; ENMD: Endocrine, nutritional and metabolic diseases; COP: Certain conditions originating in the perinatal period; MBD: Mental and behavioural disorders; NEO: Neoplasms; SSNEC: Symptoms, signs and abnormal clinical and laboratory findings, not elsewhere classified. | | | | | | | | |
| --- | --- | --- | --- | --- | --- | --- | --- | --- |
| **Article** | **Ecosystem** | **Species (reviewed names)** | **Family** | **Habit** | **Origin (Brasil)** | **Origin (Ecosystem)** | **Indication** | **Body system** |
| Albertasse et al. 2010 | Atlantic Forest | *Acanthospermum australe* (Loefl.) Kuntze | Asteraceae | H | N | N | Toothache | DDS |
| Albertasse et al. 2010 | Atlantic Forest | *Agave americana* L. | Agavaceae | H | E | E | Depurative | DBBO |
| Albertasse et al. 2010 | Atlantic Forest | *Agave americana* L. | Agavaceae | H | E | E | Skin cleanser | DSST |
| Albertasse et al. 2010 | Atlantic Forest | *Allium cepa* L. | Alliaceae | H | E | E | Hoarseness | DRS |
| Albertasse et al. 2010 | Atlantic Forest | *Allium cepa* L. | Alliaceae | H | E | E | Hoarseness | DRS |
| Albertasse et al. 2010 | Atlantic Forest | *Allium cepa* L. | Alliaceae | H | E | E | Influenza | DRS |
| Albertasse et al. 2010 | Atlantic Forest | *Allium cepa* L. | Alliaceae | H | E | E | Influenza | DRS |
| Albertasse et al. 2010 | Atlantic Forest | *Allium cepa* L. | Alliaceae | H | E | E | Throat pain | DRS |
| Albertasse et al. 2010 | Atlantic Forest | *Allium cepa* L. | Alliaceae | H | E | E | Throat pain | DRS |
| Albertasse et al. 2010 | Atlantic Forest | *Allium cepa* L. | Alliaceae | H | E | E | Voice | SSNEC |
| Albertasse et al. 2010 | Atlantic Forest | *Allium cepa* L. | Alliaceae | H | E | E | Voice | SSNEC |
| Albertasse et al. 2010 | Atlantic Forest | *Allium sativum* L. | Alliaceae | H | E | E | Hoarseness | DRS |
| Albertasse et al. 2010 | Atlantic Forest | *Allium sativum* L. | Alliaceae | H | E | E | Hoarseness | DRS |
| Albertasse et al. 2010 | Atlantic Forest | *Allium sativum* L. | Alliaceae | H | E | E | Influenza | DRS |
| Albertasse et al. 2010 | Atlantic Forest | *Allium sativum* L. | Alliaceae | H | E | E | Influenza | DRS |
| Albertasse et al. 2010 | Atlantic Forest | *Allium sativum* L. | Alliaceae | H | E | E | Throat pain | DRS |
| Albertasse et al. 2010 | Atlantic Forest | *Allium sativum* L. | Alliaceae | H | E | E | Throat pain | DRS |
| Albertasse et al. 2010 | Atlantic Forest | *Allium sativum* L. | Alliaceae | H | E | E | Voice | SSNEC |
| Albertasse et al. 2010 | Atlantic Forest | *Allium sativum* L. | Alliaceae | H | E | E | Voice | SSNEC |
| Albertasse et al. 2010 | Atlantic Forest | *Aloe vera* (L.) Burm.f. | Xanthorrhoeaceae | S | E | E | Dandruff | DSST |
| Albertasse et al. 2010 | Atlantic Forest | *Aloe vera* (L.) Burm.f. | Xanthorrhoeaceae | S | E | E | Hair loss | DSST |
| Albertasse et al. 2010 | Atlantic Forest | *Aloe vera* (L.) Burm.f. | Xanthorrhoeaceae | S | E | E | Hair moisturizer | DSST |
| Albertasse et al. 2010 | Atlantic Forest | *Alternanthera brasiliana* (L.) Kuntze | Amaranthaceae | H | N | N | Antimicrobial | CIPD |
| Albertasse et al. 2010 | Atlantic Forest | *Alternanthera brasiliana* (L.) Kuntze | Amaranthaceae | H | N | N | Dysentry | CIPD |
| Albertasse et al. 2010 | Atlantic Forest | *Alternanthera brasiliana* (L.) Kuntze | Amaranthaceae | H | N | N | Inflammation | SSNEC |
| Albertasse et al. 2010 | Atlantic Forest | *Anacardium occidentale* L. | Anacardiaceae | T | N | N | Diabetes | ENMD |
| Albertasse et al. 2010 | Atlantic Forest | *Anacardium occidentale* L. | Anacardiaceae | T | N | N | Diabetes | ENMD |
| Albertasse et al. 2010 | Atlantic Forest | *Anacardium occidentale* L. | Anacardiaceae | T | N | N | Hemorrhoids | DCS |
| Albertasse et al. 2010 | Atlantic Forest | *Anacardium occidentale* L. | Anacardiaceae | T | N | N | Hemorrhoids | DCS |
| Albertasse et al. 2010 | Atlantic Forest | *Anacardium occidentale* L. | Anacardiaceae | T | N | N | Influenza | DRS |
| Albertasse et al. 2010 | Atlantic Forest | *Anacardium occidentale* L. | Anacardiaceae | T | N | N | Influenza | DRS |
| Albertasse et al. 2010 | Atlantic Forest | *Anacardium occidentale* L. | Anacardiaceae | T | N | N | Uterus cleanser | DGS |
| Albertasse et al. 2010 | Atlantic Forest | *Anacardium occidentale* L. | Anacardiaceae | T | N | N | Uterus cleanser | DGS |
| Albertasse et al. 2010 | Atlantic Forest | *Anacardium occidentale* L. | Anacardiaceae | T | N | N | Vitamin supplement | ENMD |
| Albertasse et al. 2010 | Atlantic Forest | *Anacardium occidentale* L. | Anacardiaceae | T | N | N | Vitamin supplement | ENMD |
| Albertasse et al. 2010 | Atlantic Forest | *Anacardium occidentale* L. | Anacardiaceae | T | N | N | Wound | IPEC |
| Albertasse et al. 2010 | Atlantic Forest | *Anacardium occidentale* L. | Anacardiaceae | T | N | N | Wound | IPEC |
| Albertasse et al. 2010 | Atlantic Forest | *Ananas comosus* (L.) Merril | Bromeliaceae | H | N | N | Bronchitis | DRS |
| Albertasse et al. 2010 | Atlantic Forest | *Anethum graveolens* L. | Apiaceae | H | E | E | Labyrinthitis | DEMP |
| Albertasse et al. 2010 | Atlantic Forest | *Aristolochia cymbifera* Mart. & Zucc. | Aristolochiaceae | S | N | N | Aphrodisiac | DGS |
| Albertasse et al. 2010 | Atlantic Forest | *Aristolochia cymbifera* Mart. & Zucc. | Aristolochiaceae | S | N | N | Aphrodisiac | DGS |
| Albertasse et al. 2010 | Atlantic Forest | *Averrhoa carambola* L. | Oxalidaceae | T | E | E | Diabetes | ENMD |
| Albertasse et al. 2010 | Atlantic Forest | *Averrhoa carambola* L. | Oxalidaceae | T | E | E | High cholesterol | ENMD |
| Albertasse et al. 2010 | Atlantic Forest | *Averrhoa carambola* L. | Oxalidaceae | T | E | E | Kidney problems | DGS |
| Albertasse et al. 2010 | Atlantic Forest | *Baccharis crispa* Spreng. | Asteraceae | H | N | N | Weight reduction | ENMD |
| Albertasse et al. 2010 | Atlantic Forest | *Bidens pilosa* L. | Asteraceae | H | E | N | Jaundice | SSNEC |
| Albertasse et al. 2010 | Atlantic Forest | *Boerhavia diffusa* L. | Nyctaginaceae | H | N | N | Kidney problems | DGS |
| Albertasse et al. 2010 | Atlantic Forest | *Boerhavia diffusa* L. | Nyctaginaceae | H | N | N | Kidney problems | DGS |
| Albertasse et al. 2010 | Atlantic Forest | *Brassica oleracea* L. | Brassicaceae | H | E | E | Improves digestion | DDS |
| Albertasse et al. 2010 | Atlantic Forest | *Brassica oleracea* L. | Brassicaceae | H | E | E | Wound | IPEC |
| Albertasse et al. 2010 | Atlantic Forest | *Bromelia antiacantha* Bertol. | Bromeliaceae | H | N | N | Asthma | DRS |
| Albertasse et al. 2010 | Atlantic Forest | *Bromelia antiacantha* Bertol. | Bromeliaceae | H | N | N | Asthma | DRS |
| Albertasse et al. 2010 | Atlantic Forest | *Bromelia antiacantha* Bertol. | Bromeliaceae | H | N | N | Bronchitis | DRS |
| Albertasse et al. 2010 | Atlantic Forest | *Bromelia antiacantha* Bertol. | Bromeliaceae | H | N | N | Bronchitis | DRS |
| Albertasse et al. 2010 | Atlantic Forest | *Cajanus cajan* (L.) Huth | Fabaceae | S | E | E | Buccal inflammation | DDS |
| Albertasse et al. 2010 | Atlantic Forest | *Cajanus cajan* (L.) Huth | Fabaceae | S | E | E | Sinusitis | DRS |
| Albertasse et al. 2010 | Atlantic Forest | *Chenopodium ambrosioides* L. | Amaranthaceae | H | N | N | Calming | MBD |
| Albertasse et al. 2010 | Atlantic Forest | *Chenopodium ambrosioides* L. | Amaranthaceae | H | N | N | Helminthiasis | CIPD |
| Albertasse et al. 2010 | Atlantic Forest | *Chromolaena maximilianii* (Schrad. ex DC.) R.M.King & H.Rob. | Asteraceae | H | N | N | Depurative | DBBO |
| Albertasse et al. 2010 | Atlantic Forest | *Chromolaena maximilianii* (Schrad. ex DC.) R.M.King & H.Rob. | Asteraceae | H | N | N | Inflammation | SSNEC |
| Albertasse et al. 2010 | Atlantic Forest | *Chromolaena maximilianii* (Schrad. ex DC.) R.M.King & H.Rob. | Asteraceae | H | N | N | Pain | SSNEC |
| Albertasse et al. 2010 | Atlantic Forest | *Chrysobalanus icaco* L. | Chrysobalanaceae | S | N | N | Diabetes | ENMD |
| Albertasse et al. 2010 | Atlantic Forest | *Cissus verticillata* (L.) Nicolson & C.E.Jarvis | Vitaceae | S | N | N | Diabetes | ENMD |
| Albertasse et al. 2010 | Atlantic Forest | *Citrus aurantium* L. | Rutaceae | T | E | E | Anemia | DBBO |
| Albertasse et al. 2010 | Atlantic Forest | *Citrus aurantium* L. | Rutaceae | T | E | E | Influenza | DRS |
| Albertasse et al. 2010 | Atlantic Forest | *Citrus limon* (L.) Osbeck | Rutaceae | T | E | E | Anemia | DBBO |
| Albertasse et al. 2010 | Atlantic Forest | *Citrus limon* (L.) Osbeck | Rutaceae | T | E | E | Influenza | DRS |
| Albertasse et al. 2010 | Atlantic Forest | *Coronopus didymus* (L.) Sm. | Brassicaceae | H | N | N | Helminthiasis | CIPD |
| Albertasse et al. 2010 | Atlantic Forest | *Coronopus didymus* (L.) Sm. | Brassicaceae | H | N | N | Inflammation | SSNEC |
| Albertasse et al. 2010 | Atlantic Forest | *Coronopus didymus* (L.) Sm. | Brassicaceae | H | N | N | Wound | IPEC |
| Albertasse et al. 2010 | Atlantic Forest | *Costus spicatus* (Jacq.) Sw. | Costaceae | S | E | E | Kidney problems | DGS |
| Albertasse et al. 2010 | Atlantic Forest | *Costus spicatus* (Jacq.) Sw. | Costaceae | S | E | E | Urinary problems | DGS |
| Albertasse et al. 2010 | Atlantic Forest | *Cucurbita* pepo L. | Cucurbitaceae | H | E | E | Earache | DEMP |
| Albertasse et al. 2010 | Atlantic Forest | *Cucurbita* pepo L. | Cucurbitaceae | H | E | E | Earache | DEMP |
| Albertasse et al. 2010 | Atlantic Forest | *Cucurbita* pepo L. | Cucurbitaceae | H | E | E | Helminthiasis | CIPD |
| Albertasse et al. 2010 | Atlantic Forest | *Cucurbita* pepo L. | Cucurbitaceae | H | E | E | Helminthiasis | CIPD |
| Albertasse et al. 2010 | Atlantic Forest | *Cymbopogon citratus* (DC.) Stapf | Poaceae | H | E | E | Influenza | DRS |
| Albertasse et al. 2010 | Atlantic Forest | *Cymbopogon citratus* (DC.) Stapf | Poaceae | H | E | E | Influenza | DRS |
| Albertasse et al. 2010 | Atlantic Forest | *Cymbopogon citratus* (DC.) Stapf | Poaceae | H | E | E | Migraine | DNS |
| Albertasse et al. 2010 | Atlantic Forest | *Cymbopogon citratus* (DC.) Stapf | Poaceae | H | E | E | Migraine | DNS |
| Albertasse et al. 2010 | Atlantic Forest | *Cyrtopodium andersonii* (Lamb. ex Andrews) R.Br. | Orchidaceae | H | N | E | Wound | IPEC |
| Albertasse et al. 2010 | Atlantic Forest | *Cyrtopodium andersonii* (Lamb. ex Andrews) R.Br. | Orchidaceae | H | N | E | Wound | IPEC |
| Albertasse et al. 2010 | Atlantic Forest | *Daucus carota* L. | Apiaceae | H | E | E | Anemia | DBBO |
| Albertasse et al. 2010 | Atlantic Forest | *Daucus carota* L. | Apiaceae | H | E | E | Stomach pain/Diarrhea | SSNEC |
| Albertasse et al. 2010 | Atlantic Forest | *Eleusine indica* (L.) Gaertn. | Poaceae | H | E | E | Pneumonia | DRS |
| Albertasse et al. 2010 | Atlantic Forest | *Equisetum hyemale* L. | Equisetaceae | H | E | E | Depurative | DBBO |
| Albertasse et al. 2010 | Atlantic Forest | *Equisetum hyemale* L. | Equisetaceae | H | E | E | Depurative | DBBO |
| Albertasse et al. 2010 | Atlantic Forest | *Eugenia uniflora* L. | Myrtaceae | T | N | N | Boils | DSST |
| Albertasse et al. 2010 | Atlantic Forest | *Eugenia uniflora* L. | Myrtaceae | T | N | N | Boils | DSST |
| Albertasse et al. 2010 | Atlantic Forest | *Eugenia uniflora* L. | Myrtaceae | T | N | N | Diuretic | SSNEC |
| Albertasse et al. 2010 | Atlantic Forest | *Eugenia uniflora* L. | Myrtaceae | T | N | N | Diuretic | SSNEC |
| Albertasse et al. 2010 | Atlantic Forest | *Eugenia uniflora* L. | Myrtaceae | T | N | N | High blood pressure | DCS |
| Albertasse et al. 2010 | Atlantic Forest | *Eugenia uniflora* L. | Myrtaceae | T | N | N | High blood pressure | DCS |
| Albertasse et al. 2010 | Atlantic Forest | *Eugenia uniflora* L. | Myrtaceae | T | N | N | Influenza | DRS |
| Albertasse et al. 2010 | Atlantic Forest | *Eugenia uniflora* L. | Myrtaceae | T | N | N | Influenza | DRS |
| Albertasse et al. 2010 | Atlantic Forest | *Foeniculum vulgare* Mill. | Apiaceae | H | E | E | Calming | MBD |
| Albertasse et al. 2010 | Atlantic Forest | *Foeniculum vulgare* Mill. | Apiaceae | H | E | E | Calming | MBD |
| Albertasse et al. 2010 | Atlantic Forest | *Gossypium hirsutum* L. | Malvaceae | S | E | E | Colic | SSNEC |
| Albertasse et al. 2010 | Atlantic Forest | *Gossypium hirsutum* L. | Malvaceae | S | E | E | Inflammation of the uterus | DGS |
| Albertasse et al. 2010 | Atlantic Forest | *Gossypium hirsutum* L. | Malvaceae | S | E | E | Wound | IPEC |
| Albertasse et al. 2010 | Atlantic Forest | *Hedychium coronarium* J.Koenig | Zingiberaceae | H | E | E | Wound | IPEC |
| Albertasse et al. 2010 | Atlantic Forest | *Hedychium coronarium* J.Koenig | Zingiberaceae | H | E | E | Wound | IPEC |
| Albertasse et al. 2010 | Atlantic Forest | *Hibiscus rosa-sinensis* L. | Malvaceae | S | E | E | Hair care | DSST |
| Albertasse et al. 2010 | Atlantic Forest | *Hibiscus rosa-sinensis* L. | Malvaceae | S | E | E | Hair care | DSST |
| Albertasse et al. 2010 | Atlantic Forest | *Hibiscus rosa-sinensis* L. | Malvaceae | S | E | E | Wound | IPEC |
| Albertasse et al. 2010 | Atlantic Forest | *Hibiscus rosa-sinensis* L. | Malvaceae | S | E | E | Wound | IPEC |
| Albertasse et al. 2010 | Atlantic Forest | *Ipomoea pes-caprae* (L.) R.Br. | Convolvulaceae | H | N | N | Depurative | DBBO |
| Albertasse et al. 2010 | Atlantic Forest | *Jacaranda puberula* Cham. | Bignoniaceae | T | N | N | Erysipelas | CIPD |
| Albertasse et al. 2010 | Atlantic Forest | *Jacaranda puberula* Cham. | Bignoniaceae | T | N | N | Erysipelas | CIPD |
| Albertasse et al. 2010 | Atlantic Forest | *Jacaranda puberula* Cham. | Bignoniaceae | T | N | N | Erysipelas | CIPD |
| Albertasse et al. 2010 | Atlantic Forest | *Jacaranda puberula* Cham. | Bignoniaceae | T | N | N | Scabies | CIPD |
| Albertasse et al. 2010 | Atlantic Forest | *Jacaranda puberula* Cham. | Bignoniaceae | T | N | N | Scabies | CIPD |
| Albertasse et al. 2010 | Atlantic Forest | *Jacaranda puberula* Cham. | Bignoniaceae | T | N | N | Scabies | CIPD |
| Albertasse et al. 2010 | Atlantic Forest | *Joannesia princeps* Vell. | Euphorbiaceae | T | N | N | Depurative | DBBO |
| Albertasse et al. 2010 | Atlantic Forest | *Joannesia princeps* Vell. | Euphorbiaceae | T | N | N | Laxative | DDS |
| Albertasse et al. 2010 | Atlantic Forest | *Kalanchoe crenata* (Andrews) Haw. | Crassulaceae | H | E | E | Cough | SSNEC |
| Albertasse et al. 2010 | Atlantic Forest | *Kalanchoe crenata* (Andrews) Haw. | Crassulaceae | H | E | E | Influenza | DRS |
| Albertasse et al. 2010 | Atlantic Forest | *Kalanchoe crenata* (Andrews) Haw. | Crassulaceae | H | E | E | Wound | IPEC |
| Albertasse et al. 2010 | Atlantic Forest | *Leonurus japonicus* Houtt. | Lamiaceae | H | E | E | Abortifacient | PCP |
| Albertasse et al. 2010 | Atlantic Forest | *Leucas martinicensis* (Jacq.) R.Br. | Lamiaceae | H | E | E | Diabetes | ENMD |
| Albertasse et al. 2010 | Atlantic Forest | *Leucas martinicensis* (Jacq.) R.Br. | Lamiaceae | H | E | E | Stroke | DCS |
| Albertasse et al. 2010 | Atlantic Forest | *Lippia alba* (Mill.) N.E.Br. | Verbenaceae | S | N | N | Calming | MBD |
| Albertasse et al. 2010 | Atlantic Forest | *Lippia alba* (Mill.) N.E.Br. | Verbenaceae | S | N | N | Flatulence | SSNEC |
| Albertasse et al. 2010 | Atlantic Forest | *Lippia alba* (Mill.) N.E.Br. | Verbenaceae | S | N | N | High blood pressure | DCS |
| Albertasse et al. 2010 | Atlantic Forest | *Lippia alba* (Mill.) N.E.Br. | Verbenaceae | S | N | N | Improves digestion | DDS |
| Albertasse et al. 2010 | Atlantic Forest | *Luffa operculata* (L.) Cogn. | Cucurbitaceae | H | N | E | Sinusitis | DRS |
| Albertasse et al. 2010 | Atlantic Forest | *Malpighia glabra* L. | Malpighiaceae | T | E | E | Influenza | DRS |
| Albertasse et al. 2010 | Atlantic Forest | *Malpighia glabra* L. | Malpighiaceae | T | E | E | Influenza | DRS |
| Albertasse et al. 2010 | Atlantic Forest | *Malpighia glabra* L. | Malpighiaceae | T | E | E | Vitamin supplement | ENMD |
| Albertasse et al. 2010 | Atlantic Forest | *Malpighia glabra* L. | Malpighiaceae | T | E | E | Vitamin supplement | ENMD |
| Albertasse et al. 2010 | Atlantic Forest | *Matricaria chamomilla* L. | Asteraceae | H | E | E | Calming | MBD |
| Albertasse et al. 2010 | Atlantic Forest | *Matricaria chamomilla* L. | Asteraceae | H | E | E | Calming | MBD |
| Albertasse et al. 2010 | Atlantic Forest | *Matricaria chamomilla* L. | Asteraceae | H | E | E | Calming | MBD |
| Albertasse et al. 2010 | Atlantic Forest | *Matricaria chamomilla* L. | Asteraceae | H | E | E | Improves digestion | DDS |
| Albertasse et al. 2010 | Atlantic Forest | *Matricaria chamomilla* L. | Asteraceae | H | E | E | Improves digestion | DDS |
| Albertasse et al. 2010 | Atlantic Forest | *Matricaria chamomilla* L. | Asteraceae | H | E | E | Improves digestion | DDS |
| Albertasse et al. 2010 | Atlantic Forest | *Matricaria chamomilla* L. | Asteraceae | H | E | E | Somnific | DNS |
| Albertasse et al. 2010 | Atlantic Forest | *Matricaria chamomilla* L. | Asteraceae | H | E | E | Somnific | DNS |
| Albertasse et al. 2010 | Atlantic Forest | *Matricaria chamomilla* L. | Asteraceae | H | E | E | Somnific | DNS |
| Albertasse et al. 2010 | Atlantic Forest | *Melissa officinalis* L. | Lamiaceae | H | E | E | Calming | MBD |
| Albertasse et al. 2010 | Atlantic Forest | *Melissa officinalis* L. | Lamiaceae | H | E | E | Cardiac problems | DCS |
| Albertasse et al. 2010 | Atlantic Forest | *Mentha × piperita* L. | Lamiaceae | H | E | E | Anemia | DBBO |
| Albertasse et al. 2010 | Atlantic Forest | *Mentha × piperita* L. | Lamiaceae | H | E | E | Helminthiasis | CIPD |
| Albertasse et al. 2010 | Atlantic Forest | *Mentha × piperita* L. | Lamiaceae | H | E | E | Infant colic | SSNEC |
| Albertasse et al. 2010 | Atlantic Forest | *Mentha pulegium* L. | Lamiaceae | H | E | E | Influenza | DRS |
| Albertasse et al. 2010 | Atlantic Forest | *Mentha pulegium* L. | Lamiaceae | H | E | E | Tea for children | SSNEC |
| Albertasse et al. 2010 | Atlantic Forest | *Microgramma vacciniifolia* (Langsd. & Fisch.) Copel. | Polypodiaceae | H | N | N | Hemorrhoids | DCS |
| Albertasse et al. 2010 | Atlantic Forest | *Microgramma vacciniifolia* (Langsd. & Fisch.) Copel. | Polypodiaceae | H | N | N | Uterus cleanser | DGS |
| Albertasse et al. 2010 | Atlantic Forest | *Mikania glomerata* Spreng. | Asteraceae | S | N | N | Epilepsia | DNS |
| Albertasse et al. 2010 | Atlantic Forest | *Mikania glomerata* Spreng. | Asteraceae | S | N | N | Rheumatism | DMS |
| Albertasse et al. 2010 | Atlantic Forest | *Mikania glomerata* Spreng. | Asteraceae | S | N | N | Stroke | DCS |
| Albertasse et al. 2010 | Atlantic Forest | *Morus nigra* L. | Moraceae | T | E | E | High blood pressure | DCS |
| Albertasse et al. 2010 | Atlantic Forest | *Morus nigra* L. | Moraceae | T | E | E | High cholesterol | ENMD |
| Albertasse et al. 2010 | Atlantic Forest | *Morus nigra* L. | Moraceae | T | E | E | Hormone replacement | ENMD |
| Albertasse et al. 2010 | Atlantic Forest | *Morus nigra* L. | Moraceae | T | E | E | Stroke | DCS |
| Albertasse et al. 2010 | Atlantic Forest | *Ocimum basilicum* L. | Lamiaceae | H | E | E | Inflammation | SSNEC |
| Albertasse et al. 2010 | Atlantic Forest | *Ocimum gratissimum* L. | Lamiaceae | H | E | E | Eyewash | DEA |
| Albertasse et al. 2010 | Atlantic Forest | *Ocimum gratissimum* L. | Lamiaceae | H | E | E | Influenza | DRS |
| Albertasse et al. 2010 | Atlantic Forest | *Ocimum gratissimum* L. | Lamiaceae | H | E | E | Insomnia | DNS |
| Albertasse et al. 2010 | Atlantic Forest | *Philodendron bipinnatifidum* Schott | Araceae | S | N | N | Rheumatism | DMS |
| Albertasse et al. 2010 | Atlantic Forest | *Phyllanthus niruri* L. | Phyllanthaceae | H | N | N | Kidney problems | DGS |
| Albertasse et al. 2010 | Atlantic Forest | *Phyllanthus niruri* L. | Phyllanthaceae | H | N | N | Kidney stone | DGS |
| Albertasse et al. 2010 | Atlantic Forest | *Plantago major* L. | Plantaginaceae | H | E | E | Inflammation of the throat | DRS |
| Albertasse et al. 2010 | Atlantic Forest | *Plantago major* L. | Plantaginaceae | H | E | E | Skin ulcers | DSST |
| Albertasse et al. 2010 | Atlantic Forest | *Plectranthus amboinicus* (Lour.) Spreng. | Lamiaceae | H | E | E | Kidney problems | DGS |
| Albertasse et al. 2010 | Atlantic Forest | *Plectranthus amboinicus* (Lour.) Spreng. | Lamiaceae | H | E | E | Liver problems | DDS |
| Albertasse et al. 2010 | Atlantic Forest | *Plectranthus amboinicus* (Lour.) Spreng. | Lamiaceae | H | E | E | Migraine | DNS |
| Albertasse et al. 2010 | Atlantic Forest | *Plectranthus amboinicus* (Lour.) Spreng. | Lamiaceae | H | E | E | Pulmonary problems | DRS |
| Albertasse et al. 2010 | Atlantic Forest | *Plectranthus amboinicus* (Lour.) Spreng. | Lamiaceae | H | E | E | Stomach problems | DDS |
| Albertasse et al. 2010 | Atlantic Forest | *Plectranthus barbatus* Andr. | Lamiaceae | H | E | E | Improves digestion | DDS |
| Albertasse et al. 2010 | Atlantic Forest | *Plectranthus barbatus* Andr. | Lamiaceae | H | E | E | Liver problems | DDS |
| Albertasse et al. 2010 | Atlantic Forest | *Plectranthus ornatus* Codd | Lamiaceae | H | E | E | Improves digestion | DDS |
| Albertasse et al. 2010 | Atlantic Forest | *Protium heptaphyllum* (Aubl.) Marchand | Burseraceae | T | N | N | Arthritis | DMS |
| Albertasse et al. 2010 | Atlantic Forest | *Protium heptaphyllum* (Aubl.) Marchand | Burseraceae | T | N | N | Arthrosis | DMS |
| Albertasse et al. 2010 | Atlantic Forest | *Protium heptaphyllum* (Aubl.) Marchand | Burseraceae | T | N | N | Wound | IPEC |
| Albertasse et al. 2010 | Atlantic Forest | *Psidium guajava* L. | Myrtaceae | S | E | E | Stomach pain/Diarrhea | SSNEC |
| Albertasse et al. 2010 | Atlantic Forest | *Punica granatum* L. | Lythraceae | T | E | E | Dysentry | CIPD |
| Albertasse et al. 2010 | Atlantic Forest | *Punica granatum* L. | Lythraceae | T | E | E | Dysentry | CIPD |
| Albertasse et al. 2010 | Atlantic Forest | *Punica granatum* L. | Lythraceae | T | E | E | Inflammation of the throat | DRS |
| Albertasse et al. 2010 | Atlantic Forest | *Punica granatum* L. | Lythraceae | T | E | E | Inflammation of the throat | DRS |
| Albertasse et al. 2010 | Atlantic Forest | *Ricinus communis* L. | Euphorbiaceae | S | N | N | Erysipelas | CIPD |
| Albertasse et al. 2010 | Atlantic Forest | *Ricinus communis* L. | Euphorbiaceae | S | N | N | Skin ulcers | DSST |
| Albertasse et al. 2010 | Atlantic Forest | *Rosmarinus officinalis* L. | Lamiaceae | S | E | E | Calming | MBD |
| Albertasse et al. 2010 | Atlantic Forest | *Rosmarinus officinalis* L. | Lamiaceae | S | E | E | Cardiac problems | DCS |
| Albertasse et al. 2010 | Atlantic Forest | *Rosmarinus officinalis* L. | Lamiaceae | S | E | E | Improves digestion | DDS |
| Albertasse et al. 2010 | Atlantic Forest | *Ruta graveolens* L. | Rutaceae | H | E | E | Abortifacient | PCP |
| Albertasse et al. 2010 | Atlantic Forest | *Schinus terebinthifolius* Raddi | Anacardiaceae | T | N | N | Cough | SSNEC |
| Albertasse et al. 2010 | Atlantic Forest | *Schinus terebinthifolius* Raddi | Anacardiaceae | T | N | N | Cough | SSNEC |
| Albertasse et al. 2010 | Atlantic Forest | *Schinus terebinthifolius* Raddi | Anacardiaceae | T | N | N | Dandruff | DSST |
| Albertasse et al. 2010 | Atlantic Forest | *Schinus terebinthifolius* Raddi | Anacardiaceae | T | N | N | Dandruff | DSST |
| Albertasse et al. 2010 | Atlantic Forest | *Schinus terebinthifolius* Raddi | Anacardiaceae | T | N | N | For the puerperium | PCP |
| Albertasse et al. 2010 | Atlantic Forest | *Schinus terebinthifolius* Raddi | Anacardiaceae | T | N | N | For the puerperium | PCP |
| Albertasse et al. 2010 | Atlantic Forest | *Schinus terebinthifolius* Raddi | Anacardiaceae | T | N | N | Ulcer | DDS |
| Albertasse et al. 2010 | Atlantic Forest | *Schinus terebinthifolius* Raddi | Anacardiaceae | T | N | N | Ulcer | DDS |
| Albertasse et al. 2010 | Atlantic Forest | *Schinus terebinthifolius* Raddi | Anacardiaceae | T | N | N | Wound | IPEC |
| Albertasse et al. 2010 | Atlantic Forest | *Schinus terebinthifolius* Raddi | Anacardiaceae | T | N | N | Wound | IPEC |
| Albertasse et al. 2010 | Atlantic Forest | *Sechium edule* (Jacq.) Sw. | Cucurbitaceae | H | E | E | Calming | MBD |
| Albertasse et al. 2010 | Atlantic Forest | *Solanum melongena* L. | Solanaceae | H | E | E | Diabetes | ENMD |
| Albertasse et al. 2010 | Atlantic Forest | *Solanum melongena* L. | Solanaceae | H | E | E | High cholesterol | ENMD |
| Albertasse et al. 2010 | Atlantic Forest | *Solanum paniculatum* L. | Solanaceae | S | N | N | Cancer | NEO |
| Albertasse et al. 2010 | Atlantic Forest | *Solanum paniculatum* L. | Solanaceae | S | N | N | Cancer | NEO |
| Albertasse et al. 2010 | Atlantic Forest | *Solanum paniculatum* L. | Solanaceae | S | N | N | Liver problems | DDS |
| Albertasse et al. 2010 | Atlantic Forest | *Solanum paniculatum* L. | Solanaceae | S | N | N | Liver problems | DDS |
| Albertasse et al. 2010 | Atlantic Forest | *Solanum paniculatum* L. | Solanaceae | S | N | N | Stomach problems | DDS |
| Albertasse et al. 2010 | Atlantic Forest | *Solanum paniculatum* L. | Solanaceae | S | N | N | Stomach problems | DDS |
| Albertasse et al. 2010 | Atlantic Forest | *Solanum tuberosum* L. | Solanaceae | H | E | E | Stomach problems | DDS |
| Albertasse et al. 2010 | Atlantic Forest | *Solanum tuberosum* L. | Solanaceae | H | E | E | Ulcer | DDS |
| Albertasse et al. 2010 | Atlantic Forest | *Sparattosperma leucanthum* (Vell.) K.Schum. | Bignoniaceae | T | N | N | Antimicrobial agent for use in childbirth | PCP |
| Albertasse et al. 2010 | Atlantic Forest | *Sparattosperma leucanthum* (Vell.) K.Schum. | Bignoniaceae | T | N | N | Itchiness | DSST |
| Albertasse et al. 2010 | Atlantic Forest | *Sparattosperma leucanthum* (Vell.) K.Schum. | Bignoniaceae | T | N | N | Wound | IPEC |
| Albertasse et al. 2010 | Atlantic Forest | *Spondias mombin* L. | Anacardiaceae | T | N | N | High cholesterol | ENMD |
| Albertasse et al. 2010 | Atlantic Forest | *Spondias mombin* L. | Anacardiaceae | T | N | N | Weight reduction | ENMD |
| Albertasse et al. 2010 | Atlantic Forest | *Stachytarpheta jamaicensis* (L.)Vahl | Verbenaceae | H | N | N | Liver problems | DDS |
| Albertasse et al. 2010 | Atlantic Forest | *Stachytarpheta jamaicensis* (L.)Vahl | Verbenaceae | H | N | N | Stomach problems | DDS |
| Albertasse et al. 2010 | Atlantic Forest | *Struthanthus marginatus* (Desr.) Blume | Loranthaceae | S | N | N | Hemorrhoids | DCS |
| Albertasse et al. 2010 | Atlantic Forest | *Struthanthus marginatus* (Desr.) Blume | Loranthaceae | S | N | N | Tuberculosis | CIPD |
| Albertasse et al. 2010 | Atlantic Forest | *Struthanthus marginatus* (Desr.) Blume | Loranthaceae | S | N | N | Uterus cleanser | DGS |
| Albertasse et al. 2010 | Atlantic Forest | *Struthanthus marginatus* (Desr.) Blume | Loranthaceae | S | N | N | Wound | IPEC |
| Albertasse et al. 2010 | Atlantic Forest | *Syzygium cumini* (L.) Skeels | Myrtaceae | T | E | E | Diabetes | ENMD |
| Albertasse et al. 2010 | Atlantic Forest | *Syzygium cumini* (L.) Skeels | Myrtaceae | T | E | E | Diabetes | ENMD |
| Albertasse et al. 2010 | Atlantic Forest | *Varronia curassavica* Jacq. | Boraginaceae | S | N | N | Dental care | DDS |
| Albertasse et al. 2010 | Atlantic Forest | *Varronia curassavica* Jacq. | Boraginaceae | S | N | N | Strengthen gums | DDS |
| Albertasse et al. 2010 | Atlantic Forest | *Vernonanthura phosphorica* (Vell.) H.Rob. | Asteraceae | S | N | N | Cough | SSNEC |
| Albertasse et al. 2010 | Atlantic Forest | *Vernonanthura phosphorica* (Vell.) H.Rob. | Asteraceae | S | N | N | Influenza | DRS |
| Albertasse et al. 2010 | Atlantic Forest | *Vernonanthura phosphorica* (Vell.) H.Rob. | Asteraceae | S | N | N | Sinusitis | DRS |
| Albertasse et al. 2010 | Atlantic Forest | *Vernonanthura phosphorica* (Vell.) H.Rob. | Asteraceae | S | N | N | Tuberculosis | CIPD |
| Albertasse et al. 2010 | Atlantic Forest | *Zingiber officinale* Roscoe | Zingiberaceae | H | E | E | Hoarseness | DRS |
| Albertasse et al. 2010 | Atlantic Forest | *Zingiber officinale* Roscoe | Zingiberaceae | H | E | E | Hoarseness | DRS |
| Albertasse et al. 2010 | Atlantic Forest | *Zingiber officinale* Roscoe | Zingiberaceae | H | E | E | Influenza | DRS |
| Albertasse et al. 2010 | Atlantic Forest | *Zingiber officinale* Roscoe | Zingiberaceae | H | E | E | Influenza | DRS |
| Albertasse et al. 2010 | Atlantic Forest | *Zingiber officinale* Roscoe | Zingiberaceae | H | E | E | Throat pain | DRS |
| Albertasse et al. 2010 | Atlantic Forest | *Zingiber officinale* Roscoe | Zingiberaceae | H | E | E | Throat pain | DRS |
| Albertasse et al. 2010 | Atlantic Forest | *Zingiber officinale* Roscoe | Zingiberaceae | H | E | E | Voice | SSNEC |
| Albertasse et al. 2010 | Atlantic Forest | *Zingiber officinale* Roscoe | Zingiberaceae | H | E | E | Voice | SSNEC |
| Albuquerque & Andrade 2002a | Caatinga | *Acanthospermum hispidum* DC. | Asteraceae | H | N | E | Asthma | DRS |
| Albuquerque & Andrade 2002a | Caatinga | *Acanthospermum hispidum* DC. | Asteraceae | H | N | E | Cough | SSNEC |
| Albuquerque & Andrade 2002a | Caatinga | *Amburana cearensis* (Allemão) A.C.Sm. | Fabaceae | T | N | N | Cough | SSNEC |
| Albuquerque & Andrade 2002a | Caatinga | *Amburana cearensis* (Allemão) A.C.Sm. | Fabaceae | T | N | N | Influenza | DRS |
| Albuquerque & Andrade 2002a | Caatinga | *Anacardium occidentale* L. | Anacardiaceae | T | N | N | Blow, punch | IPEC |
| Albuquerque & Andrade 2002a | Caatinga | *Anacardium occidentale* L. | Anacardiaceae | T | N | N | Inflammation | SSNEC |
| Albuquerque & Andrade 2002a | Caatinga | *Bauhinia cheilantha* (Bong.) Steud. | Fabaceae | T | N | N | Cough | SSNEC |
| Albuquerque & Andrade 2002a | Caatinga | *Bauhinia cheilantha* (Bong.) Steud. | Fabaceae | T | N | N | Cough | SSNEC |
| Albuquerque & Andrade 2002a | Caatinga | *Bauhinia cheilantha* (Bong.) Steud. | Fabaceae | T | N | N | Diabetes | ENMD |
| Albuquerque & Andrade 2002a | Caatinga | *Bauhinia cheilantha* (Bong.) Steud. | Fabaceae | T | N | N | Expectorant | SSNEC |
| Albuquerque & Andrade 2002a | Caatinga | *Bauhinia cheilantha* (Bong.) Steud. | Fabaceae | T | N | N | Expectorant | SSNEC |
| Albuquerque & Andrade 2002a | Caatinga | *Bauhinia cheilantha* (Bong.) Steud. | Fabaceae | T | N | N | Headache | SSNEC |
| Albuquerque & Andrade 2002a | Caatinga | *Boerhavia diffusa* L. | Nyctaginaceae | H | N | N | Cough | SSNEC |
| Albuquerque & Andrade 2002a | Caatinga | *Boerhavia diffusa* L. | Nyctaginaceae | H | N | N | Inflammation | SSNEC |
| Albuquerque & Andrade 2002a | Caatinga | *Cereus jamacaru* DC. | Cactaceae | T | N | N | Kidney problems | DGS |
| Albuquerque & Andrade 2002a | Caatinga | *Chenopodium ambrosioides* L. | Amaranthaceae | H | N | N | Cough | SSNEC |
| Albuquerque & Andrade 2002a | Caatinga | *Citrus aurantium* L. | Rutaceae | T | E | E | Fever | SSNEC |
| Albuquerque & Andrade 2002a | Caatinga | *Cnidoscolus urens* (L.) Arthur | Euphorbiaceae | S | N | N | Inflammation | SSNEC |
| Albuquerque & Andrade 2002a | Caatinga | *Croton argyrophylloides* Müll.Arg. | Euphorbiaceae | S | N | N | Stomach pain/Diarrhea | SSNEC |
| Albuquerque & Andrade 2002a | Caatinga | *Croton heliotropiifolius* Kunth | Euphorbiaceae | S | N | N | Depurative | DBBO |
| Albuquerque & Andrade 2002a | Caatinga | *Cymbopogon citratus* (DC.) Stapf | Poaceae | H | E | E | Fever | SSNEC |
| Albuquerque & Andrade 2002a | Caatinga | *Cymbopogon citratus* (DC.) Stapf | Poaceae | H | E | E | Headache | SSNEC |
| Albuquerque & Andrade 2002a | Caatinga | *Cymbopogon citratus* (DC.) Stapf | Poaceae | H | E | E | Poor digestion | DDS |
| Albuquerque & Andrade 2002a | Caatinga | *Cymbopogon citratus* (DC.) Stapf | Poaceae | H | E | E | Stomach pain/Diarrhea | SSNEC |
| Albuquerque & Andrade 2002a | Caatinga | *Cynophalla flexuosa* (L.) J.Presl | Capparaceae | T | N | N | Snake bite | IPEC |
| Albuquerque & Andrade 2002a | Caatinga | *Egletes viscosa* (L.) Less. | Asteraceae | H | N | N | Poor digestion | DDS |
| Albuquerque & Andrade 2002a | Caatinga | *Egletes viscosa* (L.) Less. | Asteraceae | H | N | N | Stomach pain/Diarrhea | SSNEC |
| Albuquerque & Andrade 2002a | Caatinga | *Erythrina velutina* Willd. | Fabaceae | T | N | N | Inflammation | SSNEC |
| Albuquerque & Andrade 2002a | Caatinga | *Erythrina velutina* Willd. | Fabaceae | T | N | N | Tranquilizer | MBD |
| Albuquerque & Andrade 2002a | Caatinga | *Gossypium herbaceum* L. | Malvaceae | S | E | E | Burns | IPEC |
| Albuquerque & Andrade 2002a | Caatinga | *Hymenaea courbaril* L. | Fabaceae | T | N | N | Bronchitis | DRS |
| Albuquerque & Andrade 2002a | Caatinga | *Hymenaea courbaril* L. | Fabaceae | T | N | N | Cough | SSNEC |
| Albuquerque & Andrade 2002a | Caatinga | *Hymenaea courbaril* L. | Fabaceae | T | N | N | Weakness | SSNEC |
| Albuquerque & Andrade 2002a | Caatinga | *Hymenaea courbaril* L. | Fabaceae | T | N | N | Weakness | SSNEC |
| Albuquerque & Andrade 2002a | Caatinga | *Jatropha curcas* L. | Euphorbiaceae | S | N | N | Snake bite | IPEC |
| Albuquerque & Andrade 2002a | Caatinga | *Jatropha mollissima* (Pohl) Baill. | Euphorbiaceae | S | N | N | Snake bite | IPEC |
| Albuquerque & Andrade 2002a | Caatinga | *Kalanchoe crenata* (Andrews) Haw. | Crassulaceae | H | E | E | Pain | SSNEC |
| Albuquerque & Andrade 2002a | Caatinga | *Libidibia ferrea* (Mart. ex Tul.) L.P.Queiroz | Fabaceae | T | N | N | Kidney problems | DGS |
| Albuquerque & Andrade 2002a | Caatinga | *Libidibia ferrea* (Mart. ex Tul.) L.P.Queiroz | Fabaceae | T | N | N | Labyrinthitis | DEMP |
| Albuquerque & Andrade 2002a | Caatinga | *Lippia alba* (Mill.) N.E.Br. | Verbenaceae | S | N | N | Fever | SSNEC |
| Albuquerque & Andrade 2002a | Caatinga | *Lippia alba* (Mill.) N.E.Br. | Verbenaceae | S | N | N | Headache | SSNEC |
| Albuquerque & Andrade 2002a | Caatinga | *Lippia alba* (Mill.) N.E.Br. | Verbenaceae | S | N | N | High blood pressure | DCS |
| Albuquerque & Andrade 2002a | Caatinga | *Lippia alba* (Mill.) N.E.Br. | Verbenaceae | S | N | N | Poor digestion | DDS |
| Albuquerque & Andrade 2002a | Caatinga | *Lippia alba* (Mill.) N.E.Br. | Verbenaceae | S | N | N | Stomach pain/Diarrhea | SSNEC |
| Albuquerque & Andrade 2002a | Caatinga | *Maytenus rigida* Mart. | Celastraceae | T | N | N | Cough | SSNEC |
| Albuquerque & Andrade 2002a | Caatinga | *Maytenus rigida* Mart. | Celastraceae | T | N | N | Rheumatism | DMS |
| Albuquerque & Andrade 2002a | Caatinga | *Mimosa tenuiflora* (Willd.) Poir. | Fabaceae | S | N | N | Inflammation | SSNEC |
| Albuquerque & Andrade 2002a | Caatinga | *Mimosa tenuiflora* (Willd.) Poir. | Fabaceae | S | N | N | Toothache | DDS |
| Albuquerque & Andrade 2002a | Caatinga | *Momordica charantia* L. | Cucurbitaceae | H | E | E | Allergy | IPEC |
| Albuquerque & Andrade 2002a | Caatinga | *Momordica charantia* L. | Cucurbitaceae | H | E | E | Skin rash | SSNEC |
| Albuquerque & Andrade 2002a | Caatinga | *Myracrodruon urundeuva* Allemão | Anacardiaceae | T | N | N | Blow, punch | IPEC |
| Albuquerque & Andrade 2002a | Caatinga | *Myracrodruon urundeuva* Allemão | Anacardiaceae | T | N | N | Gastritis | DDS |
| Albuquerque & Andrade 2002a | Caatinga | *Myracrodruon urundeuva* Allemão | Anacardiaceae | T | N | N | Inflammation | SSNEC |
| Albuquerque & Andrade 2002a | Caatinga | *Ocimum campechianum* Mill. | Lamiaceae | H | N | N | Poor digestion | DDS |
| Albuquerque & Andrade 2002a | Caatinga | *Ocimum campechianum* Mill. | Lamiaceae | H | N | N | Stomach pain/Diarrhea | SSNEC |
| Albuquerque & Andrade 2002a | Caatinga | *Passiflora foetida* L. | Passifloraceae | H | N | N | Cough | SSNEC |
| Albuquerque & Andrade 2002a | Caatinga | *Phyllanthus niruri* L. | Phyllanthaceae | H | N | N | Inflammation of the kidney | DGS |
| Albuquerque & Andrade 2002a | Caatinga | *Phyllanthus niruri* L. | Phyllanthaceae | H | N | N | Kidney stone | DGS |
| Albuquerque & Andrade 2002a | Caatinga | *Piptadenia stipulacea* (Benth.) Ducke | Fabaceae | T | N | N | Inflammation | SSNEC |
| Albuquerque & Andrade 2002a | Caatinga | *Poincianella pyramidalis* (Tul.) L.P.Queiroz | Fabaceae | T | N | N | Cough | SSNEC |
| Albuquerque & Andrade 2002a | Caatinga | *Psidium guajava* L. | Myrtaceae | S | E | E | Dysentry | CIPD |
| Albuquerque & Andrade 2002a | Caatinga | *Rosmarinus officinalis* L. | Lamiaceae | S | E | E | Fever | SSNEC |
| Albuquerque & Andrade 2002a | Caatinga | *Ruta graveolens* L. | Rutaceae | H | E | E | Headache | SSNEC |
| Albuquerque & Andrade 2002a | Caatinga | *Sapindus saponaria* L. | Sapindaceae | T | N | E | Mycosis | CIPD |
| Albuquerque & Andrade 2002a | Caatinga | *Schinopsis brasiliensis* Engl. | Anacardiaceae | T | N | N | Cough | SSNEC |
| Albuquerque & Andrade 2002a | Caatinga | *Schinopsis brasiliensis* Engl. | Anacardiaceae | T | N | N | Influenza | DRS |
| Albuquerque & Andrade 2002a | Caatinga | *Senna martiana* (Benth.) H.S.Irwin & Barneby | Fabaceae | S | N | N | Cough | SSNEC |
| Albuquerque & Andrade 2002a | Caatinga | *Serjania comata* Radlk. | Sapindaceae | S | N | E | Rheumatism | DMS |
| Albuquerque & Andrade 2002a | Caatinga | *Sideroxylon obtusifolium* (Roem. & Schult.) T.D.Penn. | Sapotaceae | T | N | N | Blow, punch | IPEC |
| Albuquerque & Andrade 2002a | Caatinga | *Sideroxylon obtusifolium* (Roem. & Schult.) T.D.Penn. | Sapotaceae | T | N | N | Inflammation | SSNEC |
| Albuquerque & Andrade 2002a | Caatinga | *Sideroxylon obtusifolium* (Roem. & Schult.) T.D.Penn. | Sapotaceae | T | N | N | Wound | IPEC |
| Albuquerque & Andrade 2002a | Caatinga | *Solanum paniculatum* L. | Solanaceae | S | N | N | Liver problems | DDS |
| Albuquerque & Andrade 2002a | Caatinga | *Solanum paniculatum* L. | Solanaceae | S | N | N | Liver problems | DDS |
| Albuquerque & Andrade 2002a | Caatinga | *Tarenaya spinosa* (Jacq.) Raf. | Capparaceae | H | N | N | Cough | SSNEC |
| Albuquerque & Andrade 2002a | Caatinga | *Tarenaya spinosa* (Jacq.) Raf. | Capparaceae | H | N | N | Inflammation | SSNEC |
| Albuquerque & Andrade 2002a | Caatinga | *Ziziphus joazeiro* Mart. | Rhamnaceae | T | N | N | Cough | SSNEC |
| Albuquerque & Andrade 2002a | Caatinga | *Ziziphus joazeiro* Mart. | Rhamnaceae | T | N | N | Wound | IPEC |
| Almeida et al. 2009 | Atlantic Forest | *Achyrocline satureioides* (Lam.) DC. | Asteraceae | S | N | N | Poor digestion | DDS |
| Almeida et al. 2009 | Atlantic Forest | *Aloe perfoliata* L. | Xanthorrhoeaceae | S | E | E | Burns | IPEC |
| Almeida et al. 2009 | Atlantic Forest | *Arctium lappa* L. | Asteraceae | H | E | E | Kidney problems | DGS |
| Almeida et al. 2009 | Atlantic Forest | *Baccharis crispa* Spreng. | Asteraceae | H | N | N | Diabetes | ENMD |
| Almeida et al. 2009 | Atlantic Forest | *Baccharis crispa* Spreng. | Asteraceae | H | N | N | Helminthiasis | CIPD |
| Almeida et al. 2009 | Atlantic Forest | *Calendula officinalis* L. | Asteraceae | H | E | E | Antiseptic | CIPD |
| Almeida et al. 2009 | Atlantic Forest | *Calendula officinalis* L. | Asteraceae | H | E | E | Antiseptic | CIPD |
| Almeida et al. 2009 | Atlantic Forest | *Calendula officinalis* L. | Asteraceae | H | E | E | Inflammation | SSNEC |
| Almeida et al. 2009 | Atlantic Forest | *Calendula officinalis* L. | Asteraceae | H | E | E | Inflammation | SSNEC |
| Almeida et al. 2009 | Atlantic Forest | *Calendula officinalis* L. | Asteraceae | H | E | E | Wound | IPEC |
| Almeida et al. 2009 | Atlantic Forest | *Calendula officinalis* L. | Asteraceae | H | E | E | Wound | IPEC |
| Almeida et al. 2009 | Atlantic Forest | *Chenopodium ambrosioides* L. | Amaranthaceae | H | N | N | Helminthiasis | CIPD |
| Almeida et al. 2009 | Atlantic Forest | *Costus spicatus* (Jacq.) Sw. | Costaceae | S | E | E | Depurative | DBBO |
| Almeida et al. 2009 | Atlantic Forest | *Costus spicatus* (Jacq.) Sw. | Costaceae | S | E | E | Diuretic | SSNEC |
| Almeida et al. 2009 | Atlantic Forest | *Cynara cardunculus* L. | Asteraceae | H | E | E | High cholesterol | ENMD |
| Almeida et al. 2009 | Atlantic Forest | *Cynara cardunculus* L. | Asteraceae | H | E | E | Hypoglycemic agent | SSNEC |
| Almeida et al. 2009 | Atlantic Forest | *EchinoPainus macrophyllus (Kunth) Micheli* | Alismataceae | H | N | N | Depurative | DBBO |
| Almeida et al. 2009 | Atlantic Forest | *Foeniculum vulgare* Mill. | Apiaceae | H | E | E | Calming | MBD |
| Almeida et al. 2009 | Atlantic Forest | *Foeniculum vulgare* Mill. | Apiaceae | H | E | E | Flatulence | SSNEC |
| Almeida et al. 2009 | Atlantic Forest | *Leonurus sibiricus* L. | Lamiaceae | H | E | E | Hypertension | DCS |
| Almeida et al. 2009 | Atlantic Forest | *Leonurus sibiricus* L. | Lamiaceae | H | E | E | Stimulates circulation | DCS |
| Almeida et al. 2009 | Atlantic Forest | *Lippia alba* (Mill.) N.E.Br. | Verbenaceae | S | N | N | Calming | MBD |
| Almeida et al. 2009 | Atlantic Forest | *Lippia alba* (Mill.) N.E.Br. | Verbenaceae | S | N | N | Colic | SSNEC |
| Almeida et al. 2009 | Atlantic Forest | *Melissa officinalis* L. | Lamiaceae | H | E | E | Calming | MBD |
| Almeida et al. 2009 | Atlantic Forest | *Melissa officinalis* L. | Lamiaceae | H | E | E | Insomnia | DNS |
| Almeida et al. 2009 | Atlantic Forest | *Melissa officinalis* L. | Lamiaceae | H | E | E | Migraine | DNS |
| Almeida et al. 2009 | Atlantic Forest | *Mikania glomerata* Spreng. | Asteraceae | S | N | N | Asthma | DRS |
| Almeida et al. 2009 | Atlantic Forest | *Mikania glomerata* Spreng. | Asteraceae | S | N | N | Bronchitis | DRS |
| Almeida et al. 2009 | Atlantic Forest | *Mikania glomerata* Spreng. | Asteraceae | S | N | N | Cough | SSNEC |
| Almeida et al. 2009 | Atlantic Forest | *Nasturtium officinale* W. T. Aiton | Brassicaceae | H | E | E | Anemia | DBBO |
| Almeida et al. 2009 | Atlantic Forest | *Nasturtium officinale* W. T. Aiton | Brassicaceae | H | E | E | Liver problems | DDS |
| Almeida et al. 2009 | Atlantic Forest | *Petiveria alliacea* L. | Phytolaccaceae | H | E | E | Colic | SSNEC |
| Almeida et al. 2009 | Atlantic Forest | *Petiveria alliacea* L. | Phytolaccaceae | H | E | E | Diuretic | SSNEC |
| Almeida et al. 2009 | Atlantic Forest | *Petiveria alliacea* L. | Phytolaccaceae | H | E | E | Sudorific | SSNEC |
| Almeida et al. 2009 | Atlantic Forest | *Plectranthus barbatus* Andr. | Lamiaceae | H | E | E | Digestive stimulant | DDS |
| Almeida et al. 2009 | Atlantic Forest | *Plectranthus barbatus* Andr. | Lamiaceae | H | E | E | Heartburn | DGS |
| Almeida et al. 2009 | Atlantic Forest | *Rosmarinus officinalis* L. | Lamiaceae | S | E | E | Flatulence | SSNEC |
| Almeida et al. 2009 | Atlantic Forest | *Rosmarinus officinalis* L. | Lamiaceae | S | E | E | Nervousness | MBD |
| Almeida et al. 2009 | Atlantic Forest | *Ruta graveolens* L. | Rutaceae | H | E | E | Inflammation | SSNEC |
| Almeida et al. 2009 | Atlantic Forest | *Sechium edule* (Jacq.) Sw. | Cucurbitaceae | H | E | E | Diuretic | SSNEC |
| Almeida et al. 2009 | Atlantic Forest | *Sechium edule* (Jacq.) Sw. | Cucurbitaceae | H | E | E | High blood pressure | DCS |
| Almeida et al. 2009 | Atlantic Forest | *Sedum dendroideum* DC. | Crassulaceae | H | E | E | Gastrointestinal inflammation | DDS |
| Almeida et al. 2009 | Atlantic Forest | *Sedum dendroideum* DC. | Crassulaceae | H | E | E | Skin inflammation | DSST |
| Almeida et al. 2009 | Atlantic Forest | *Solanum melongena* L. | Solanaceae | H | E | E | High blood pressure | DCS |
| Almeida et al. 2009 | Atlantic Forest | *Solidago chilensis* Meyen | Asteraceae | H | N | N | Inflammation | SSNEC |
| Almeida et al. 2009 | Atlantic Forest | *Solidago chilensis* Meyen | Asteraceae | H | N | N | Pain | SSNEC |
| Almeida et al. 2009 | Atlantic Forest | *Stryphnodendron aSTDringens (Mart.) Coville* | Fabaceae | T | N | E | Hemorrhoids | DCS |
| Almeida et al. 2009 | Atlantic Forest | *Stryphnodendron aSTDringens (Mart.) Coville* | Fabaceae | T | N | E | Stomach pain/Diarrhea | SSNEC |
| Almeida et al. 2009 | Atlantic Forest | *Stryphnodendron aSTDringens (Mart.) Coville* | Fabaceae | T | N | E | Wound | IPEC |
| Almeida et al. 2009 | Atlantic Forest | *Vernonanthura phosphorica* (Vell.) H.Rob. | Asteraceae | S | N | N | Bronchitis | DRS |
| Almeida et al. 2009 | Atlantic Forest | *Vernonanthura phosphorica* (Vell.) H.Rob. | Asteraceae | S | N | N | Cough | SSNEC |
| Andrade et al.2006 | Caatinga | *Cereus jamacaru* DC. | Cactaceae | T | N | N | Burns | IPEC |
| Andrade et al.2006 | Caatinga | *Cereus jamacaru* DC. | Cactaceae | T | N | N | Influenza | DRS |
| Andrade et al.2006 | Caatinga | *Cereus jamacaru* DC. | Cactaceae | T | N | N | Kidney problems | DGS |
| Andrade et al.2006 | Caatinga | *Cereus jamacaru* DC. | Cactaceae | T | N | N | Problems in the urethra | DGS |
| Andrade et al.2006 | Caatinga | *Cereus jamacaru* DC. | Cactaceae | T | N | N | Spinal problems | DMS |
| Andrade et al.2006 | Caatinga | *Cereus jamacaru* DC. | Cactaceae | T | N | N | Syphilis | CIPD |
| Andrade et al.2006 | Caatinga | *Cereus jamacaru* DC. | Cactaceae | T | N | N | Wound | IPEC |
| Andrade et al.2006 | Caatinga | *Harrisia adscendens* (Gürke) Britton & Rose | Cactaceae | S | N | N | Burns | IPEC |
| Andrade et al.2006 | Caatinga | *Harrisia adscendens* (Gürke) Britton & Rose | Cactaceae | S | N | N | Kidney problems | DGS |
| Andrade et al.2006 | Caatinga | *Harrisia adscendens* (Gürke) Britton & Rose | Cactaceae | S | N | N | Prostate problems | DGS |
| Andrade et al.2006 | Caatinga | *Harrisia adscendens* (Gürke) Britton & Rose | Cactaceae | S | N | N | Toothache | DDS |
| Andrade et al.2006 | Caatinga | *Melocactus zehntneri* (Britton & Rose) Luetzelb. | Cactaceae | S | N | N | Colic | SSNEC |
| Andrade et al.2006 | Caatinga | *Melocactus zehntneri* (Britton & Rose) Luetzelb. | Cactaceae | S | N | N | Intestinal problems | DDS |
| Andrade et al.2006 | Caatinga | *Melocactus zehntneri* (Britton & Rose) Luetzelb. | Cactaceae | S | N | N | Toothache | DDS |
| Andrade et al.2006 | Caatinga | *Opuntia ficus-indica* (L.) Mill. | Cactaceae | S | E | E | Burns | IPEC |
| Andrade et al.2006 | Caatinga | *Opuntia ficus-indica* (L.) Mill. | Cactaceae | S | E | E | Choking | SSNEC |
| Andrade et al.2006 | Caatinga | *Opuntia ficus-indica* (L.) Mill. | Cactaceae | S | E | E | Constipation | DDS |
| Andrade et al.2006 | Caatinga | *Opuntia ficus-indica* (L.) Mill. | Cactaceae | S | E | E | Dysentry | CIPD |
| Andrade et al.2006 | Caatinga | *Opuntia ficus-indica* (L.) Mill. | Cactaceae | S | E | E | Inflammation of the uterus | DGS |
| Andrade et al.2006 | Caatinga | *Opuntia ficus-indica* (L.) Mill. | Cactaceae | S | E | E | Influenza | DRS |
| Andrade et al.2006 | Caatinga | *Opuntia ficus-indica* (L.) Mill. | Cactaceae | S | E | E | Rheumatism | DMS |
| Andrade et al.2006 | Caatinga | *Opuntia ficus-indica* (L.) Mill. | Cactaceae | S | E | E | Stomach pain/Diarrhea | SSNEC |
| Andrade et al.2006 | Caatinga | *Opuntia ficus-indica* (L.) Mill. | Cactaceae | S | E | E | Swollen abdomen | SSNEC |
| Andrade et al.2006 | Caatinga | *Opuntia ficus-indica* (L.) Mill. | Cactaceae | S | E | E | Urinary infection | DGS |
| Andrade et al.2006 | Caatinga | *Opuntia ficus-indica* (L.) Mill. | Cactaceae | S | E | E | Vaginal inflammation | DGS |
| Andrade et al.2006 | Caatinga | *Pilosocereus gounellei* (F.A.C.Weber) Byles & Rowley | Cactaceae | T | N | N | Wound | IPEC |
| Andrade et al.2006 | Caatinga | *Tacinga palmaPaina (Britton & Rose) N.P.Taylor & Stuppy* | Cactaceae | S | N | N | Burns | IPEC |
| Andrade et al.2006 | Caatinga | *Tacinga palmaPaina (Britton & Rose) N.P.Taylor & Stuppy* | Cactaceae | S | N | N | Problems in the urethra | DGS |
| Baldauf et al. 2009 | Pampa | *Achyrocline satureioides* (Lam.) DC. | Asteraceae | S | N | N | Stomach pain/Diarrhea | SSNEC |
| Baldauf et al. 2009 | Pampa | *Aloysia citrioPaina Palau* | Verbenaceae | S | E | E | Calming | MBD |
| Baldauf et al. 2009 | Pampa | *Baccharis articulata* (Lam.) Pers. | Asteraceae | S | N | N | Weight reduction | ENMD |
| Baldauf et al. 2009 | Pampa | *Bromelia antiacantha* Bertol. | Bromeliaceae | H | N | E | Cough | SSNEC |
| Baldauf et al. 2009 | Pampa | *Casearia sylvestris* Sw. | Salicaceae | T | N | N | Lowers cholesterol | DCS |
| Baldauf et al. 2009 | Pampa | *Cirsium vulgare* (Savi) Ten. | Asteraceae | H | E | E | Stomach problems | DDS |
| Baldauf et al. 2009 | Pampa | *Citrus reticulata* Blanco | Rutaceae | T | E | E | Cough | SSNEC |
| Baldauf et al. 2009 | Pampa | *Citrus sinensis* (L.) Osbeck | Rutaceae | T | E | E | Influenza | DRS |
| Baldauf et al. 2009 | Pampa | *Commelina erecta* L. | Commelinaceae | H | N | E | Eye drops | DEA |
| Baldauf et al. 2009 | Pampa | *Coronopus didymus* (L.) Sm. | Brassicaceae | H | N | E | Cough | SSNEC |
| Baldauf et al. 2009 | Pampa | *Coronopus didymus* (L.) Sm. | Brassicaceae | H | N | E | Influenza | DRS |
| Baldauf et al. 2009 | Pampa | *Cunila microcephala* Benth. | Lamiaceae | H | N | N | Cough | SSNEC |
| Baldauf et al. 2009 | Pampa | *Cuphea carthagenensis* (Jacq.) J.Macbr. | Lythraceae | H | N | N | Hemorrhoids | DCS |
| Baldauf et al. 2009 | Pampa | *Cymbopogon citratus* (DC.) Stapf | Poaceae | H | E | E | Calming | MBD |
| Baldauf et al. 2009 | Pampa | *Eugenia uniflora* L. | Myrtaceae | T | N | E | Stomach pain/Diarrhea | SSNEC |
| Baldauf et al. 2009 | Pampa | *Foeniculum vulgare* Mill. | Apiaceae | H | E | E | Flatulence | SSNEC |
| Baldauf et al. 2009 | Pampa | *Luehea divaricata* Mart. & Zucc. | Malvaceae | T | N | E | Cough | SSNEC |
| Baldauf et al. 2009 | Pampa | *Melia azedarach* L | Meliaceae | T | E | E | Wound | IPEC |
| Baldauf et al. 2009 | Pampa | *Mikania laevigata* Sch.Bip. ex Baker | Asteraceae | S | N | E | Cough | SSNEC |
| Baldauf et al. 2009 | Pampa | *Persea americana* Mill. | Lauraceae | T | E | E | Kidney problems | DGS |
| Baldauf et al. 2009 | Pampa | *Persea americana* Mill. | Lauraceae | T | E | E | Urinary Retention | DGS |
| Baldauf et al. 2009 | Pampa | *Plectranthus barbatus* Andr. | Lamiaceae | H | E | E | Liver pain | DDS |
| Baldauf et al. 2009 | Pampa | *Plectranthus barbatus* Andr. | Lamiaceae | H | E | E | Stomach pain/Diarrhea | SSNEC |
| Baldauf et al. 2009 | Pampa | *Polygonum punctatum* Elliott | Polygonaceae | H | N | E | Hemorrhoids | DCS |
| Baldauf et al. 2009 | Pampa | *Psidium guajava* L. | Myrtaceae | S | E | E | Stomach pain/Diarrhea | SSNEC |
| Baldauf et al. 2009 | Pampa | *Punica granatum* L. | Lythraceae | T | E | E | Stomach pain/Diarrhea | SSNEC |
| Baldauf et al. 2009 | Pampa | *Sambucus australis* Cham. & Schltdl. | Adoxaceae | T | N | E | Measles | CIPD |
| Baldauf et al. 2009 | Pampa | *Scoparia dulcis* L. | Plantaginaceae | H | N | N | Apetite stimulant | SSNEC |
| Baldauf et al. 2009 | Pampa | *Sechium edule* (Jacq.) Sw. | Cucurbitaceae | H | E | E | High blood pressure | DCS |
| Baldauf et al. 2009 | Pampa | *Sida rhombifolia* L. | Malvaceae | H | N | N | Stomach pain/Diarrhea | SSNEC |
| Baldauf et al. 2009 | Pampa | *Smilax campestris* Griseb. | Smilacaceae | S | N | N | Depurative | DBBO |
| Baldauf et al. 2009 | Pampa | *Solanum paniculatum* L. | Solanaceae | S | N | E | Liver pain | DDS |
| Baldauf et al. 2009 | Pampa | *Stachytarpheta cayennensis* (Rich.) Vahl | Verbenaceae | S | N | E | Cough | SSNEC |
| Begossi et al. 1993 | Atlantic Forest | *Acalypha poiretii* Spreng. | Euphorbiaceae | H | N | N | Stomach pain/Diarrhea | SSNEC |
| Begossi et al. 1993 | Atlantic Forest | *Ageratum conyzoides* L. | Asteraceae | H | N | N | Torsion | SSNEC |
| Begossi et al. 1993 | Atlantic Forest | *Allium sativum* L. | Alliaceae | H | E | E | Snake bite | IPEC |
| Begossi et al. 1993 | Atlantic Forest | *Aloysia citrioPaina Palau* | Verbenaceae | S | E | E | Cough | SSNEC |
| Begossi et al. 1993 | Atlantic Forest | *Aloysia citrioPaina Palau* | Verbenaceae | S | E | E | High blood pressure | DCS |
| Begossi et al. 1993 | Atlantic Forest | *Aloysia citrioPaina Palau* | Verbenaceae | S | E | E | Influenza | DRS |
| Begossi et al. 1993 | Atlantic Forest | *Aloysia citrioPaina Palau* | Verbenaceae | S | E | E | Menstrual cramps | DGS |
| Begossi et al. 1993 | Atlantic Forest | *Aloysia citrioPaina Palau* | Verbenaceae | S | E | E | Sedative | MBD |
| Begossi et al. 1993 | Atlantic Forest | *Aloysia citrioPaina Palau* | Verbenaceae | S | E | E | Stomach problems | DDS |
| Begossi et al. 1993 | Atlantic Forest | *Anacardium occidentale* L. | Anacardiaceae | T | N | N | Stomach pain/Diarrhea | SSNEC |
| Begossi et al. 1993 | Atlantic Forest | *Artemisia absinthium* L. | Asteraceae | H | E | E | Abortifacient | PCP |
| Begossi et al. 1993 | Atlantic Forest | *Artemisia absinthium* L. | Asteraceae | H | E | E | Helminthiasis | CIPD |
| Begossi et al. 1993 | Atlantic Forest | *Artemisia absinthium* L. | Asteraceae | H | E | E | Stomach pain/Diarrhea | SSNEC |
| Begossi et al. 1993 | Atlantic Forest | *Artemisia absinthium* L. | Asteraceae | H | E | E | Stomach problems | DDS |
| Begossi et al. 1993 | Atlantic Forest | *Baccharis crispa* Spreng. | Asteraceae | H | N | N | High blood pressure | DCS |
| Begossi et al. 1993 | Atlantic Forest | *Baccharis crispa* Spreng. | Asteraceae | H | N | N | Liver problems | DDS |
| Begossi et al. 1993 | Atlantic Forest | *Baccharis crispa* Spreng. | Asteraceae | H | N | N | Stomach pain/Diarrhea | SSNEC |
| Begossi et al. 1993 | Atlantic Forest | *Baccharis crispa* Spreng. | Asteraceae | H | N | N | Stomach problems | DDS |
| Begossi et al. 1993 | Atlantic Forest | *Bidens pilosa* L. | Asteraceae | H | E | N | Hepatitis | CIPD |
| Begossi et al. 1993 | Atlantic Forest | *Bidens pilosa* L. | Asteraceae | H | E | N | Itchiness | DSST |
| Begossi et al. 1993 | Atlantic Forest | *Bidens pilosa* L. | Asteraceae | H | E | N | Lesion | IPEC |
| Begossi et al. 1993 | Atlantic Forest | *Boerhavia diffusa* L. | Nyctaginaceae | H | N | N | Hepatitis | CIPD |
| Begossi et al. 1993 | Atlantic Forest | *Cajanus cajan* (L.) Huth | Fabaceae | S | E | E | Toothache | DDS |
| Begossi et al. 1993 | Atlantic Forest | *Chenopodium ambrosioides* L. | Amaranthaceae | H | N | N | Helminthiasis | CIPD |
| Begossi et al. 1993 | Atlantic Forest | *Chenopodium ambrosioides* L. | Amaranthaceae | H | N | N | Lesion | IPEC |
| Begossi et al. 1993 | Atlantic Forest | *Chenopodium ambrosioides* L. | Amaranthaceae | H | N | N | Stomach pain/Diarrhea | SSNEC |
| Begossi et al. 1993 | Atlantic Forest | *Citrus aurantiifolia* (Christm.) Swingle | Rutaceae | T | E | E | Influenza | DRS |
| Begossi et al. 1993 | Atlantic Forest | *Citrus aurantiifolia* (Christm.) Swingle | Rutaceae | T | E | E | Influenza | DRS |
| Begossi et al. 1993 | Atlantic Forest | *Citrus aurantiifolia* (Christm.) Swingle | Rutaceae | T | E | E | Toothache | DDS |
| Begossi et al. 1993 | Atlantic Forest | *Citrus aurantiifolia* (Christm.) Swingle | Rutaceae | T | E | E | Toothache | DDS |
| Begossi et al. 1993 | Atlantic Forest | *Citrus sinensis* (L.) Osbeck | Rutaceae | T | E | E | Influenza | DRS |
| Begossi et al. 1993 | Atlantic Forest | *Cocos nucifera* L. | Arecaceae | T | N | N | Abortifacient | PCP |
| Begossi et al. 1993 | Atlantic Forest | *Cunila spicata* Benth. | Lamiaceae | H | N | N | Cough | SSNEC |
| Begossi et al. 1993 | Atlantic Forest | *Cunila spicata* Benth. | Lamiaceae | H | N | N | Helminthiasis | CIPD |
| Begossi et al. 1993 | Atlantic Forest | *Cunila spicata* Benth. | Lamiaceae | H | N | N | Influenza | DRS |
| Begossi et al. 1993 | Atlantic Forest | *Cunila spicata* Benth. | Lamiaceae | H | N | N | Stomach pain/Diarrhea | SSNEC |
| Begossi et al. 1993 | Atlantic Forest | *Cymbopogon citratus* (DC.) Stapf | Poaceae | H | E | E | Cough | SSNEC |
| Begossi et al. 1993 | Atlantic Forest | *Cymbopogon citratus* (DC.) Stapf | Poaceae | H | E | E | High blood pressure | DCS |
| Begossi et al. 1993 | Atlantic Forest | *Cymbopogon citratus* (DC.) Stapf | Poaceae | H | E | E | Influenza | DRS |
| Begossi et al. 1993 | Atlantic Forest | *Cymbopogon citratus* (DC.) Stapf | Poaceae | H | E | E | Insomnia | DNS |
| Begossi et al. 1993 | Atlantic Forest | *Cymbopogon citratus* (DC.) Stapf | Poaceae | H | E | E | Sedative | MBD |
| Begossi et al. 1993 | Atlantic Forest | *Cymbopogon citratus* (DC.) Stapf | Poaceae | H | E | E | Stomach problems | DDS |
| Begossi et al. 1993 | Atlantic Forest | *EchinoPainus grandiflorus (Cham. & Schltr.) Micheli* | Alismataceae | H | N | N | Rheumatism | DMS |
| Begossi et al. 1993 | Atlantic Forest | *Eugenia uniflora* L. | Myrtaceae | T | N | N | Urinary pain | DGS |
| Begossi et al. 1993 | Atlantic Forest | *Euphorbia hirta* L. | Euphorbiaceae | H | N | N | Stomach problems | DDS |
| Begossi et al. 1993 | Atlantic Forest | *Foeniculum vulgare* Mill. | Apiaceae | H | E | E | Asthma | DRS |
| Begossi et al. 1993 | Atlantic Forest | *Foeniculum vulgare* Mill. | Apiaceae | H | E | E | Headache | SSNEC |
| Begossi et al. 1993 | Atlantic Forest | *Foeniculum vulgare* Mill. | Apiaceae | H | E | E | Helminthiasis | CIPD |
| Begossi et al. 1993 | Atlantic Forest | *Foeniculum vulgare* Mill. | Apiaceae | H | E | E | Infant colic | SSNEC |
| Begossi et al. 1993 | Atlantic Forest | *Foeniculum vulgare* Mill. | Apiaceae | H | E | E | Influenza | DRS |
| Begossi et al. 1993 | Atlantic Forest | *Foeniculum vulgare* Mill. | Apiaceae | H | E | E | Stomach pain/Diarrhea | SSNEC |
| Begossi et al. 1993 | Atlantic Forest | *Gymnanthemum amygdalinum* (Delile) Sch.Bip. ex Walp. | Asteraceae | S | N | N | Stomach pain/Diarrhea | SSNEC |
| Begossi et al. 1993 | Atlantic Forest | *Hyptis suaveolens* Poit. | Lamiaceae | H | N | N | Lesion | IPEC |
| Begossi et al. 1993 | Atlantic Forest | *Imperata brasiliensis* Trin. | Poaceae | H | N | N | Abortifacient | PCP |
| Begossi et al. 1993 | Atlantic Forest | *Indigofera suffruticosa* Mill. | Fabaceae | S | N | N | Undefined | SSNEC |
| Begossi et al. 1993 | Atlantic Forest | *Laurus nobilis* L. | Lauraceae | T | E | E | Heartburn | DGS |
| Begossi et al. 1993 | Atlantic Forest | *Lepidium virginicum* L. | Brassicaceae | H | E | E | Lesion | IPEC |
| Begossi et al. 1993 | Atlantic Forest | *Lepidium virginicum* L. | Brassicaceae | H | E | E | Pneumonia | DRS |
| Begossi et al. 1993 | Atlantic Forest | *Matricaria chamomilla* L. | Asteraceae | H | E | E | Stomach pain/Diarrhea | SSNEC |
| Begossi et al. 1993 | Atlantic Forest | *Mentha spicata* L. | Lamiaceae | H | E | E | Bronchitis | DRS |
| Begossi et al. 1993 | Atlantic Forest | *Mentha spicata* L. | Lamiaceae | H | E | E | Cough | SSNEC |
| Begossi et al. 1993 | Atlantic Forest | *Mentha spicata* L. | Lamiaceae | H | E | E | Helminthiasis | CIPD |
| Begossi et al. 1993 | Atlantic Forest | *Mentha spicata* L. | Lamiaceae | H | E | E | Stomach pain/Diarrhea | SSNEC |
| Begossi et al. 1993 | Atlantic Forest | *Mikania cordifolia* (L.f.) Willd. | Asteraceae | S | N | N | Snake bite | IPEC |
| Begossi et al. 1993 | Atlantic Forest | *Musa acuminata* Colla | Musaceae | H | E | E | Acne | DSST |
| Begossi et al. 1993 | Atlantic Forest | *Musa acuminata* Colla | Musaceae | H | E | E | Boils | DSST |
| Begossi et al. 1993 | Atlantic Forest | *Nasturtium officinale* W. T. Aiton | Brassicaceae | H | E | E | Influenza | DRS |
| Begossi et al. 1993 | Atlantic Forest | *Passiflora edulis* Sims | Passifloraceae | S | N | N | Cardiac problems | DCS |
| Begossi et al. 1993 | Atlantic Forest | *Passiflora edulis* Sims | Passifloraceae | S | N | N | High blood pressure | DCS |
| Begossi et al. 1993 | Atlantic Forest | *Passiflora edulis* Sims | Passifloraceae | S | N | N | Toothache | DDS |
| Begossi et al. 1993 | Atlantic Forest | *Persea americana* Mill. | Lauraceae | T | E | E | Liver problems | DDS |
| Begossi et al. 1993 | Atlantic Forest | *Persea americana* Mill. | Lauraceae | T | E | E | Urinary problems | DGS |
| Begossi et al. 1993 | Atlantic Forest | *Phyllanthus tenellus* Roxb. | Phyllanthaceae | H | N | N | Urinary pain | DGS |
| Begossi et al. 1993 | Atlantic Forest | *Piper umbellatum* L. | Piperaceae | S | N | N | Kidney problems | DGS |
| Begossi et al. 1993 | Atlantic Forest | *Piper umbellatum* L. | Piperaceae | S | N | N | Liver problems | DDS |
| Begossi et al. 1993 | Atlantic Forest | *Plectranthus barbatus* Andr. | Lamiaceae | H | E | E | Liver problems | DDS |
| Begossi et al. 1993 | Atlantic Forest | *Plectranthus barbatus* Andr. | Lamiaceae | H | E | E | Stomach pain/Diarrhea | SSNEC |
| Begossi et al. 1993 | Atlantic Forest | *Plectranthus barbatus* Andr. | Lamiaceae | H | E | E | Stomach problems | DDS |
| Begossi et al. 1993 | Atlantic Forest | *Porophyllum ruderale* (Jacq.) Cass. | Asteraceae | H | N | E | Stomach pain/Diarrhea | SSNEC |
| Begossi et al. 1993 | Atlantic Forest | *Psidium cattleianum* Sabine | Myrtaceae | S | N | N | Stomach pain/Diarrhea | SSNEC |
| Begossi et al. 1993 | Atlantic Forest | *Psidium guajava* L. | Myrtaceae | S | E | E | Stomach pain/Diarrhea | SSNEC |
| Begossi et al. 1993 | Atlantic Forest | *Pyrostegia venusta* (Ker Gawl.) Miers | Bignoniaceae | S | N | N | Snake bite | IPEC |
| Begossi et al. 1993 | Atlantic Forest | *Ruta graveolens* L. | Rutaceae | H | E | E | Abortifacient | PCP |
| Begossi et al. 1993 | Atlantic Forest | *Sambucus australis* Cham. & Schltdl. | Adoxaceae | T | N | N | Measles | CIPD |
| Begossi et al. 1993 | Atlantic Forest | *Stachytarpheta polyura* Schauer | Verbenaceae | H | N | E | Liver problems | DDS |
| Begossi et al. 1993 | Atlantic Forest | *Zanthoxylum rhoifolium* Lam. | Rutaceae | T | N | N | Hepatitis | CIPD |
| Brandão et al. 1992 | Amazon | *Acanthospermum australe* (Loefl.) Kuntze | Asteraceae | H | N | N | Fever | SSNEC |
| Brandão et al. 1992 | Amazon | *Acanthospermum australe* (Loefl.) Kuntze | Asteraceae | H | N | N | Fever | SSNEC |
| Brandão et al. 1992 | Amazon | *Acanthospermum australe* (Loefl.) Kuntze | Asteraceae | H | N | N | Malaria | CIPD |
| Brandão et al. 1992 | Amazon | *Acanthospermum australe* (Loefl.) Kuntze | Asteraceae | H | N | N | Malaria | CIPD |
| Brandão et al. 1992 | Amazon | *Acmella oleracea* (L.) R.K.Jansen | Asteraceae | H | N | E | Liver problems | DDS |
| Brandão et al. 1992 | Amazon | *Ampelozizyphus amazonicu*s Ducke | Rhamnaceae | S | N | N | Malaria (preventive) | CIPD |
| Brandão et al. 1992 | Amazon | *Bixa orellana* L. | Bixaceae | T | N | N | Malaria | CIPD |
| Brandão et al. 1992 | Amazon | *Boerhavia coccinea* Mill. | Nyctaginaceae | H | E | E | Malaria | CIPD |
| Brandão et al. 1992 | Amazon | *Cassia spruceana* Benth. | Fabaceae | T | N | N | Fever | SSNEC |
| Brandão et al. 1992 | Amazon | *Coutarea hexandra* (Jacq.) K.Schum. | Rubiaceae | T | N | N | Malaria | CIPD |
| Brandão et al. 1992 | Amazon | *Desmodium adscendens* (Sw.) DC. | Fabaceae | H | N | N | Liver problems | DDS |
| Brandão et al. 1992 | Amazon | *Geissospermum sericeum* Miers | Apocynaceae | T | N | N | Malaria | CIPD |
| Brandão et al. 1992 | Amazon | *Gossypium herbaceum* L. | Malvaceae | S | E | E | Liver problems | DDS |
| Brandão et al. 1992 | Amazon | *Gymnanthemum amygdalinum* (Delile) Sch.Bip. ex Walp. | Asteraceae | S | N | N | Liver problems | DDS |
| Brandão et al. 1992 | Amazon | *Leonotis nepetifolia* (L.) R.Br. | Lamiaceae | H | N | N | Liver problems | DDS |
| Brandão et al. 1992 | Amazon | *Phanera rutilans* (Spruce ex Benth.) Vaz | Fabaceae | S | N | N | Liver problems | DDS |
| Brandão et al. 1992 | Amazon | *Physalis brasiliensis* Sendtn. | Solanaceae | H | N | N | Liver problems | DDS |
| Brandão et al. 1992 | Amazon | *Pluchea sagittalis* (Lam.) Cabrera | Asteraceae | H | N | N | Liver problems | DDS |
| Brandão et al. 1992 | Amazon | *Portulaca pilosa* L. | Portulacaceae | H | N | N | Fever | SSNEC |
| Brandão et al. 1992 | Amazon | *Senna alata* (L.) Roxb. | Fabaceae | S | N | N | Fever | SSNEC |
| Brandão et al. 1992 | Amazon | *Senna occidentalis* (L.) Link | Fabaceae | S | N | N | Malaria | CIPD |
| Brandão et al. 1992 | Amazon | *Sida spinosa* L. | Malvaceae | H | N | E | Liver problems | DDS |
| Brandão et al. 2006 | Cerrado and Atlantic Forest | *Achyrocline satureioides* (Lam.) DC. | Asteraceae | S | N | N | Inflammation | SSNEC |
| Brandão et al. 2006 | Cerrado and Atlantic Forest | *Baccharis crispa* Spreng. | Asteraceae | H | N | N | Digestive problems | DDS |
| Brandão et al. 2006 | Cerrado and Atlantic Forest | *Baccharis crispa* Spreng. | Asteraceae | H | N | N | Pain | SSNEC |
| Brandão et al. 2006 | Cerrado and Atlantic Forest | *Cymbopogon citratus* (DC.) Stapf | Poaceae | H | E | E | Nervousness | MBD |
| Brandão et al. 2006 | Cerrado and Atlantic Forest | *Foeniculum vulgare* Mill. | Apiaceae | H | E | E | Flatulence | SSNEC |
| Brandão et al. 2006 | Cerrado and Atlantic Forest | *Gymnanthemum amygdalinum* (Delile) Sch.Bip. ex Walp. | Asteraceae | S | N | N | Liver problems | DDS |
| Brandão et al. 2006 | Cerrado and Atlantic Forest | *Matricaria chamomilla* L. | Asteraceae | H | E | E | Inflammation | SSNEC |
| Brandão et al. 2006 | Cerrado and Atlantic Forest | *Matricaria chamomilla* L. | Asteraceae | H | E | E | Nervousness | MBD |
| Brandão et al. 2006 | Cerrado and Atlantic Forest | *Melissa officinalis* L. | Lamiaceae | H | E | E | Nervousness | MBD |
| Brandão et al. 2006 | Cerrado and Atlantic Forest | *Mentha pulegium* L. | Lamiaceae | H | E | E | Respiratory problems | DRS |
| Brandão et al. 2006 | Cerrado and Atlantic Forest | *Mikania glomerata* Spreng. | Asteraceae | S | N | N | Bronchitis | DRS |
| Brandão et al. 2006 | Cerrado and Atlantic Forest | *Peumus boldus* Molina | Monimiaceae | T | E | E | Liver problems | DDS |
| Brandão et al. 2006 | Cerrado and Atlantic Forest | *Phyllanthus niruri* L. | Phyllanthaceae | H | N | N | Diuretic | SSNEC |
| Brandão et al. 2006 | Cerrado and Atlantic Forest | *Phyllanthus niruri* L. | Phyllanthaceae | H | N | N | Kidney stone | DGS |
| Brandão et al. 2006 | Cerrado and Atlantic Forest | *Phyllanthus tenellus* Roxb. | Phyllanthaceae | H | N | N | Diuretic | SSNEC |
| Brandão et al. 2006 | Cerrado and Atlantic Forest | *Phyllanthus tenellus* Roxb. | Phyllanthaceae | H | N | N | Kidney stone | DGS |
| Brandão et al. 2006 | Cerrado and Atlantic Forest | *Pimpinella anisum* L. | Apiaceae | H | E | E | Flatulence | SSNEC |
| Brandão et al. 2006 | Cerrado and Atlantic Forest | *Plectranthus barbatus* Andr. | Lamiaceae | H | E | E | Liver problems | DDS |
| Brandão et al. 2006 | Cerrado and Atlantic Forest | *Rosmarinus officinalis* L. | Lamiaceae | S | E | E | High blood pressure | DCS |
| Cartaxo et al. 2010 | Caatinga | *Acanthospermum hispidum* DC. | Asteraceae | H | N | E | Inflammation | SSNEC |
| Cartaxo et al. 2010 | Caatinga | *Acanthospermum hispidum* DC. | Asteraceae | H | N | E | Sinusitis | DRS |
| Cartaxo et al. 2010 | Caatinga | *Acanthospermum hispidum* DC. | Asteraceae | H | N | E | Toothache | DDS |
| Cartaxo et al. 2010 | Caatinga | *Achillea millefolium* L. | Asteraceae | H | E | E | Headache | SSNEC |
| Cartaxo et al. 2010 | Caatinga | *Achillea millefolium* L. | Asteraceae | H | E | E | Pain | SSNEC |
| Cartaxo et al. 2010 | Caatinga | *Allium cepa* L. | Alliaceae | H | E | E | Constipation | DDS |
| Cartaxo et al. 2010 | Caatinga | *Allium cepa* L. | Alliaceae | H | E | E | Constipation | DDS |
| Cartaxo et al. 2010 | Caatinga | *Allium cepa* L. | Alliaceae | H | E | E | Flatulence | SSNEC |
| Cartaxo et al. 2010 | Caatinga | *Allium cepa* L. | Alliaceae | H | E | E | Flatulence | SSNEC |
| Cartaxo et al. 2010 | Caatinga | *Allium cepa* L. | Alliaceae | H | E | E | Hypertension | DCS |
| Cartaxo et al. 2010 | Caatinga | *Allium cepa* L. | Alliaceae | H | E | E | Hypertension | DCS |
| Cartaxo et al. 2010 | Caatinga | *Allium cepa* L. | Alliaceae | H | E | E | Infant colic | SSNEC |
| Cartaxo et al. 2010 | Caatinga | *Allium cepa* L. | Alliaceae | H | E | E | Infant colic | SSNEC |
| Cartaxo et al. 2010 | Caatinga | *Allium cepa* L. | Alliaceae | H | E | E | Influenza | DRS |
| Cartaxo et al. 2010 | Caatinga | *Allium cepa* L. | Alliaceae | H | E | E | Influenza | DRS |
| Cartaxo et al. 2010 | Caatinga | *Allium sativum* L. | Alliaceae | H | E | E | Asthma | DRS |
| Cartaxo et al. 2010 | Caatinga | *Allium sativum* L. | Alliaceae | H | E | E | Bronchitis | DRS |
| Cartaxo et al. 2010 | Caatinga | *Allium sativum* L. | Alliaceae | H | E | E | Hypertension | DCS |
| Cartaxo et al. 2010 | Caatinga | *Allium sativum* L. | Alliaceae | H | E | E | Influenza | DRS |
| Cartaxo et al. 2010 | Caatinga | *Allium sativum* L. | Alliaceae | H | E | E | Menstrual cramps | DGS |
| Cartaxo et al. 2010 | Caatinga | *Allium sativum* L. | Alliaceae | H | E | E | Poor digestion | DDS |
| Cartaxo et al. 2010 | Caatinga | *Allium sativum* L. | Alliaceae | H | E | E | Sinusitis | DRS |
| Cartaxo et al. 2010 | Caatinga | *Aloe vera* (L.) Burm.f. | Xanthorrhoeaceae | S | E | E | Cancer | NEO |
| Cartaxo et al. 2010 | Caatinga | *Aloe vera* (L.) Burm.f. | Xanthorrhoeaceae | S | E | E | Hemorrhoids | DCS |
| Cartaxo et al. 2010 | Caatinga | *Aloe vera* (L.) Burm.f. | Xanthorrhoeaceae | S | E | E | Hypertension | DCS |
| Cartaxo et al. 2010 | Caatinga | *Aloe vera* (L.) Burm.f. | Xanthorrhoeaceae | S | E | E | Lack of apetite | SSNEC |
| Cartaxo et al. 2010 | Caatinga | *Aloe vera* (L.) Burm.f. | Xanthorrhoeaceae | S | E | E | Ulcer | DDS |
| Cartaxo et al. 2010 | Caatinga | *Aloe vera* (L.) Burm.f. | Xanthorrhoeaceae | S | E | E | Wound | IPEC |
| Cartaxo et al. 2010 | Caatinga | *Alpinia zerumbet* (Pers.) B.L.Burtt & R.M.Sm. | Zingiberaceae | H | E | E | Hypertension | DCS |
| Cartaxo et al. 2010 | Caatinga | *Amburana cearensis* (Allemão) A.C.Sm. | Fabaceae | T | N | N | Cough | SSNEC |
| Cartaxo et al. 2010 | Caatinga | *Amburana cearensis* (Allemão) A.C.Sm. | Fabaceae | T | N | N | Cough | SSNEC |
| Cartaxo et al. 2010 | Caatinga | *Amburana cearensis* (Allemão) A.C.Sm. | Fabaceae | T | N | N | Cough | SSNEC |
| Cartaxo et al. 2010 | Caatinga | *Amburana cearensis* (Allemão) A.C.Sm. | Fabaceae | T | N | N | Expectorant | SSNEC |
| Cartaxo et al. 2010 | Caatinga | *Amburana cearensis* (Allemão) A.C.Sm. | Fabaceae | T | N | N | Expectorant | SSNEC |
| Cartaxo et al. 2010 | Caatinga | *Amburana cearensis* (Allemão) A.C.Sm. | Fabaceae | T | N | N | Expectorant | SSNEC |
| Cartaxo et al. 2010 | Caatinga | *Amburana cearensis* (Allemão) A.C.Sm. | Fabaceae | T | N | N | Hypertension | DCS |
| Cartaxo et al. 2010 | Caatinga | *Amburana cearensis* (Allemão) A.C.Sm. | Fabaceae | T | N | N | Hypertension | DCS |
| Cartaxo et al. 2010 | Caatinga | *Amburana cearensis* (Allemão) A.C.Sm. | Fabaceae | T | N | N | Hypertension | DCS |
| Cartaxo et al. 2010 | Caatinga | *Amburana cearensis* (Allemão) A.C.Sm. | Fabaceae | T | N | N | Inflammation | SSNEC |
| Cartaxo et al. 2010 | Caatinga | *Amburana cearensis* (Allemão) A.C.Sm. | Fabaceae | T | N | N | Inflammation | SSNEC |
| Cartaxo et al. 2010 | Caatinga | *Amburana cearensis* (Allemão) A.C.Sm. | Fabaceae | T | N | N | Inflammation | SSNEC |
| Cartaxo et al. 2010 | Caatinga | *Amburana cearensis* (Allemão) A.C.Sm. | Fabaceae | T | N | N | Influenza | DRS |
| Cartaxo et al. 2010 | Caatinga | *Amburana cearensis* (Allemão) A.C.Sm. | Fabaceae | T | N | N | Influenza | DRS |
| Cartaxo et al. 2010 | Caatinga | *Amburana cearensis* (Allemão) A.C.Sm. | Fabaceae | T | N | N | Influenza | DRS |
| Cartaxo et al. 2010 | Caatinga | *Amburana cearensis* (Allemão) A.C.Sm. | Fabaceae | T | N | N | Nasal congestion | DRS |
| Cartaxo et al. 2010 | Caatinga | *Amburana cearensis* (Allemão) A.C.Sm. | Fabaceae | T | N | N | Nasal congestion | DRS |
| Cartaxo et al. 2010 | Caatinga | *Amburana cearensis* (Allemão) A.C.Sm. | Fabaceae | T | N | N | Nasal congestion | DRS |
| Cartaxo et al. 2010 | Caatinga | *Amburana cearensis* (Allemão) A.C.Sm. | Fabaceae | T | N | N | Respiratory problems | DRS |
| Cartaxo et al. 2010 | Caatinga | *Amburana cearensis* (Allemão) A.C.Sm. | Fabaceae | T | N | N | Respiratory problems | DRS |
| Cartaxo et al. 2010 | Caatinga | *Amburana cearensis* (Allemão) A.C.Sm. | Fabaceae | T | N | N | Respiratory problems | DRS |
| Cartaxo et al. 2010 | Caatinga | *Amburana cearensis* (Allemão) A.C.Sm. | Fabaceae | T | N | N | Rhinitis | DRS |
| Cartaxo et al. 2010 | Caatinga | *Amburana cearensis* (Allemão) A.C.Sm. | Fabaceae | T | N | N | Rhinitis | DRS |
| Cartaxo et al. 2010 | Caatinga | *Amburana cearensis* (Allemão) A.C.Sm. | Fabaceae | T | N | N | Rhinitis | DRS |
| Cartaxo et al. 2010 | Caatinga | *Amburana cearensis* (Allemão) A.C.Sm. | Fabaceae | T | N | N | Sinusitis | DRS |
| Cartaxo et al. 2010 | Caatinga | *Amburana cearensis* (Allemão) A.C.Sm. | Fabaceae | T | N | N | Sinusitis | DRS |
| Cartaxo et al. 2010 | Caatinga | *Amburana cearensis* (Allemão) A.C.Sm. | Fabaceae | T | N | N | Sinusitis | DRS |
| Cartaxo et al. 2010 | Caatinga | *Amburana cearensis* (Allemão) A.C.Sm. | Fabaceae | T | N | N | Thrombosis | DCS |
| Cartaxo et al. 2010 | Caatinga | *Amburana cearensis* (Allemão) A.C.Sm. | Fabaceae | T | N | N | Thrombosis | DCS |
| Cartaxo et al. 2010 | Caatinga | *Amburana cearensis* (Allemão) A.C.Sm. | Fabaceae | T | N | N | Thrombosis | DCS |
| Cartaxo et al. 2010 | Caatinga | *Amburana cearensis* (Allemão) A.C.Sm. | Fabaceae | T | N | N | Wound | IPEC |
| Cartaxo et al. 2010 | Caatinga | *Amburana cearensis* (Allemão) A.C.Sm. | Fabaceae | T | N | N | Wound | IPEC |
| Cartaxo et al. 2010 | Caatinga | *Amburana cearensis* (Allemão) A.C.Sm. | Fabaceae | T | N | N | Wound | IPEC |
| Cartaxo et al. 2010 | Caatinga | *Anacardium occidentale* L. | Anacardiaceae | T | N | N | Antiseptic | CIPD |
| Cartaxo et al. 2010 | Caatinga | *Anacardium occidentale* L. | Anacardiaceae | T | N | N | Cancer | NEO |
| Cartaxo et al. 2010 | Caatinga | *Anacardium occidentale* L. | Anacardiaceae | T | N | N | Feminine hygiene | DGS |
| Cartaxo et al. 2010 | Caatinga | *Anacardium occidentale* L. | Anacardiaceae | T | N | N | Gengivitis | DDS |
| Cartaxo et al. 2010 | Caatinga | *Anacardium occidentale* L. | Anacardiaceae | T | N | N | Inflammation of internal organs | SSNEC |
| Cartaxo et al. 2010 | Caatinga | *Anacardium occidentale* L. | Anacardiaceae | T | N | N | Throat problems | DRS |
| Cartaxo et al. 2010 | Caatinga | *Anacardium occidentale* L. | Anacardiaceae | T | N | N | Toothache | DDS |
| Cartaxo et al. 2010 | Caatinga | *Anacardium occidentale* L. | Anacardiaceae | T | N | N | Wound | IPEC |
| Cartaxo et al. 2010 | Caatinga | *Anadenanthera colubrina* (Vell.) Brenan | Fabaceae | T | N | N | Antiseptic | CIPD |
| Cartaxo et al. 2010 | Caatinga | *Anadenanthera colubrina* (Vell.) Brenan | Fabaceae | T | N | N | Antiseptic | CIPD |
| Cartaxo et al. 2010 | Caatinga | *Anadenanthera colubrina* (Vell.) Brenan | Fabaceae | T | N | N | Cancer | NEO |
| Cartaxo et al. 2010 | Caatinga | *Anadenanthera colubrina* (Vell.) Brenan | Fabaceae | T | N | N | Cancer | NEO |
| Cartaxo et al. 2010 | Caatinga | *Anadenanthera colubrina* (Vell.) Brenan | Fabaceae | T | N | N | Cough | SSNEC |
| Cartaxo et al. 2010 | Caatinga | *Anadenanthera colubrina* (Vell.) Brenan | Fabaceae | T | N | N | Cough | SSNEC |
| Cartaxo et al. 2010 | Caatinga | *Anadenanthera colubrina* (Vell.) Brenan | Fabaceae | T | N | N | Expectorant | SSNEC |
| Cartaxo et al. 2010 | Caatinga | *Anadenanthera colubrina* (Vell.) Brenan | Fabaceae | T | N | N | Expectorant | SSNEC |
| Cartaxo et al. 2010 | Caatinga | *Anadenanthera colubrina* (Vell.) Brenan | Fabaceae | T | N | N | Infection | CIPD |
| Cartaxo et al. 2010 | Caatinga | *Anadenanthera colubrina* (Vell.) Brenan | Fabaceae | T | N | N | Infection | CIPD |
| Cartaxo et al. 2010 | Caatinga | *Anadenanthera colubrina* (Vell.) Brenan | Fabaceae | T | N | N | Inflammation of the throat | DRS |
| Cartaxo et al. 2010 | Caatinga | *Anadenanthera colubrina* (Vell.) Brenan | Fabaceae | T | N | N | Inflammation of the throat | DRS |
| Cartaxo et al. 2010 | Caatinga | *Anadenanthera colubrina* (Vell.) Brenan | Fabaceae | T | N | N | Influenza | DRS |
| Cartaxo et al. 2010 | Caatinga | *Anadenanthera colubrina* (Vell.) Brenan | Fabaceae | T | N | N | Influenza | DRS |
| Cartaxo et al. 2010 | Caatinga | *Anadenanthera colubrina* (Vell.) Brenan | Fabaceae | T | N | N | Nasal congestion | DRS |
| Cartaxo et al. 2010 | Caatinga | *Anadenanthera colubrina* (Vell.) Brenan | Fabaceae | T | N | N | Nasal congestion | DRS |
| Cartaxo et al. 2010 | Caatinga | *Anadenanthera colubrina* (Vell.) Brenan | Fabaceae | T | N | N | Pulmonary problems | DRS |
| Cartaxo et al. 2010 | Caatinga | *Anadenanthera colubrina* (Vell.) Brenan | Fabaceae | T | N | N | Pulmonary problems | DRS |
| Cartaxo et al. 2010 | Caatinga | *Anadenanthera colubrina* (Vell.) Brenan | Fabaceae | T | N | N | Stomach pain/Diarrhea | SSNEC |
| Cartaxo et al. 2010 | Caatinga | *Anadenanthera colubrina* (Vell.) Brenan | Fabaceae | T | N | N | Stomach pain/Diarrhea | SSNEC |
| Cartaxo et al. 2010 | Caatinga | *Anadenanthera colubrina* (Vell.) Brenan | Fabaceae | T | N | N | Wound | IPEC |
| Cartaxo et al. 2010 | Caatinga | *Anadenanthera colubrina* (Vell.) Brenan | Fabaceae | T | N | N | Wound | IPEC |
| Cartaxo et al. 2010 | Caatinga | *Ananas comosus* (L.) Merril | Bromeliaceae | H | N | N | Expectorant | SSNEC |
| Cartaxo et al. 2010 | Caatinga | *Ananas comosus* (L.) Merril | Bromeliaceae | H | N | N | Influenza | DRS |
| Cartaxo et al. 2010 | Caatinga | *Anethum graveolens* L. | Apiaceae | H | E | E | Calming | MBD |
| Cartaxo et al. 2010 | Caatinga | *Anethum graveolens* L. | Apiaceae | H | E | E | Pain | SSNEC |
| Cartaxo et al. 2010 | Caatinga | *Anethum graveolens* L. | Apiaceae | H | E | E | Poor digestion | DDS |
| Cartaxo et al. 2010 | Caatinga | *Annona crotonifolia* Mart. | Annonaceae | S | N | E | Poor digestion | DDS |
| Cartaxo et al. 2010 | Caatinga | *Annona muricata* L. | Annonaceae | T | E | E | Urinary problems | DGS |
| Cartaxo et al. 2010 | Caatinga | *Artemisia absinthium* L. | Asteraceae | H | E | E | Poor digestion | DDS |
| Cartaxo et al. 2010 | Caatinga | *Astronium fraxinifolium* Schott | Anacardiaceae | T | N | E | Dysentry | CIPD |
| Cartaxo et al. 2010 | Caatinga | *Astronium fraxinifolium* Schott | Anacardiaceae | T | N | E | Fever | SSNEC |
| Cartaxo et al. 2010 | Caatinga | *Bidens pilosa* L. | Asteraceae | H | E | E | Apendicitis | DDS |
| Cartaxo et al. 2010 | Caatinga | *Bidens pilosa* L. | Asteraceae | H | E | E | Apendicitis | DDS |
| Cartaxo et al. 2010 | Caatinga | *Bidens pilosa* L. | Asteraceae | H | E | E | Inflammation | SSNEC |
| Cartaxo et al. 2010 | Caatinga | *Bidens pilosa* L. | Asteraceae | H | E | E | Inflammation | SSNEC |
| Cartaxo et al. 2010 | Caatinga | *Bignonia ramentacea* (Mart. ex DC.) L.G.Lohmann | Bignoniaceae | S | N | N | Cough | SSNEC |
| Cartaxo et al. 2010 | Caatinga | *Bixa orellana* L. | Bixaceae | T | N | E | Bronchitis | DRS |
| Cartaxo et al. 2010 | Caatinga | *Bixa orellana* L. | Bixaceae | T | N | E | Bronchitis | DRS |
| Cartaxo et al. 2010 | Caatinga | *Bixa orellana* L. | Bixaceae | T | N | E | Diabetes | ENMD |
| Cartaxo et al. 2010 | Caatinga | *Bixa orellana* L. | Bixaceae | T | N | E | Diabetes | ENMD |
| Cartaxo et al. 2010 | Caatinga | *Bixa orellana* L. | Bixaceae | T | N | E | Influenza | DRS |
| Cartaxo et al. 2010 | Caatinga | *Bixa orellana* L. | Bixaceae | T | N | E | Influenza | DRS |
| Cartaxo et al. 2010 | Caatinga | *Bixa orellana* L. | Bixaceae | T | N | E | Throat problems | DRS |
| Cartaxo et al. 2010 | Caatinga | *Bixa orellana* L. | Bixaceae | T | N | E | Throat problems | DRS |
| Cartaxo et al. 2010 | Caatinga | *Brassica juncea* (L.) Czern. | Brassicaceae | H | E | E | Headache | SSNEC |
| Cartaxo et al. 2010 | Caatinga | *Brassica juncea* (L.) Czern. | Brassicaceae | H | E | E | Stroke | DCS |
| Cartaxo et al. 2010 | Caatinga | *Brassica juncea* (L.) Czern. | Brassicaceae | H | E | E | Thrombosis | DCS |
| Cartaxo et al. 2010 | Caatinga | *Bredemeyera brevifolia* (Benth.) Klotzsch ex A.W.Benn. | Polygalaceae | S | N | N | Influenza | DRS |
| Cartaxo et al. 2010 | Caatinga | *Carica papaya* L. | Caricaceae | T | E | E | Bronchitis | DRS |
| Cartaxo et al. 2010 | Caatinga | *Carica papaya* L. | Caricaceae | T | E | E | Bronchitis | DRS |
| Cartaxo et al. 2010 | Caatinga | *Carica papaya* L. | Caricaceae | T | E | E | Bronchitis | DRS |
| Cartaxo et al. 2010 | Caatinga | *Carica papaya* L. | Caricaceae | T | E | E | Cough | SSNEC |
| Cartaxo et al. 2010 | Caatinga | *Carica papaya* L. | Caricaceae | T | E | E | Cough | SSNEC |
| Cartaxo et al. 2010 | Caatinga | *Carica papaya* L. | Caricaceae | T | E | E | Cough | SSNEC |
| Cartaxo et al. 2010 | Caatinga | *Carica papaya* L. | Caricaceae | T | E | E | Expectorant | SSNEC |
| Cartaxo et al. 2010 | Caatinga | *Carica papaya* L. | Caricaceae | T | E | E | Expectorant | SSNEC |
| Cartaxo et al. 2010 | Caatinga | *Carica papaya* L. | Caricaceae | T | E | E | Expectorant | SSNEC |
| Cartaxo et al. 2010 | Caatinga | *Carica papaya* L. | Caricaceae | T | E | E | Influenza | DRS |
| Cartaxo et al. 2010 | Caatinga | *Carica papaya* L. | Caricaceae | T | E | E | Influenza | DRS |
| Cartaxo et al. 2010 | Caatinga | *Carica papaya* L. | Caricaceae | T | E | E | Influenza | DRS |
| Cartaxo et al. 2010 | Caatinga | *Carica papaya* L. | Caricaceae | T | E | E | Poor digestion | DDS |
| Cartaxo et al. 2010 | Caatinga | *Carica papaya* L. | Caricaceae | T | E | E | Poor digestion | DDS |
| Cartaxo et al. 2010 | Caatinga | *Carica papaya* L. | Caricaceae | T | E | E | Poor digestion | DDS |
| Cartaxo et al. 2010 | Caatinga | *Carica papaya* L. | Caricaceae | T | E | E | Stomach problems | DDS |
| Cartaxo et al. 2010 | Caatinga | *Carica papaya* L. | Caricaceae | T | E | E | Stomach problems | DDS |
| Cartaxo et al. 2010 | Caatinga | *Carica papaya* L. | Caricaceae | T | E | E | Stomach problems | DDS |
| Cartaxo et al. 2010 | Caatinga | *Ceiba glaziovii* (Kuntze) K.Schum. | Malvaceae | T | N | N | Anemia | DBBO |
| Cartaxo et al. 2010 | Caatinga | *Cenchrus spinosus* Rojas | Poaceae | H | E | E | Diuretic | SSNEC |
| Cartaxo et al. 2010 | Caatinga | *Cereus jamacaru* DC. | Cactaceae | T | N | N | Antiseptic | CIPD |
| Cartaxo et al. 2010 | Caatinga | *Cereus jamacaru* DC. | Cactaceae | T | N | N | Expectorant | SSNEC |
| Cartaxo et al. 2010 | Caatinga | *Cereus jamacaru* DC. | Cactaceae | T | N | N | Inflammation of the uterus | DGS |
| Cartaxo et al. 2010 | Caatinga | *Cereus jamacaru* DC. | Cactaceae | T | N | N | Influenza | DRS |
| Cartaxo et al. 2010 | Caatinga | *Cereus jamacaru* DC. | Cactaceae | T | N | N | Wound | IPEC |
| Cartaxo et al. 2010 | Caatinga | *Chenopodium ambrosioides* L. | Amaranthaceae | H | N | N | Colic | SSNEC |
| Cartaxo et al. 2010 | Caatinga | *Chenopodium ambrosioides* L. | Amaranthaceae | H | N | N | Colic | SSNEC |
| Cartaxo et al. 2010 | Caatinga | *Chenopodium ambrosioides* L. | Amaranthaceae | H | N | N | Colic | SSNEC |
| Cartaxo et al. 2010 | Caatinga | *Chenopodium ambrosioides* L. | Amaranthaceae | H | N | N | Expectorant | SSNEC |
| Cartaxo et al. 2010 | Caatinga | *Chenopodium ambrosioides* L. | Amaranthaceae | H | N | N | Expectorant | SSNEC |
| Cartaxo et al. 2010 | Caatinga | *Chenopodium ambrosioides* L. | Amaranthaceae | H | N | N | Expectorant | SSNEC |
| Cartaxo et al. 2010 | Caatinga | *Chenopodium ambrosioides* L. | Amaranthaceae | H | N | N | Fracture | IPEC |
| Cartaxo et al. 2010 | Caatinga | *Chenopodium ambrosioides* L. | Amaranthaceae | H | N | N | Fracture | IPEC |
| Cartaxo et al. 2010 | Caatinga | *Chenopodium ambrosioides* L. | Amaranthaceae | H | N | N | Fracture | IPEC |
| Cartaxo et al. 2010 | Caatinga | *Chenopodium ambrosioides* L. | Amaranthaceae | H | N | N | Gallbladder problems | DDS |
| Cartaxo et al. 2010 | Caatinga | *Chenopodium ambrosioides* L. | Amaranthaceae | H | N | N | Gallbladder problems | DDS |
| Cartaxo et al. 2010 | Caatinga | *Chenopodium ambrosioides* L. | Amaranthaceae | H | N | N | Gallbladder problems | DDS |
| Cartaxo et al. 2010 | Caatinga | *Chenopodium ambrosioides* L. | Amaranthaceae | H | N | N | Gastritis | DDS |
| Cartaxo et al. 2010 | Caatinga | *Chenopodium ambrosioides* L. | Amaranthaceae | H | N | N | Gastritis | DDS |
| Cartaxo et al. 2010 | Caatinga | *Chenopodium ambrosioides* L. | Amaranthaceae | H | N | N | Gastritis | DDS |
| Cartaxo et al. 2010 | Caatinga | *Chenopodium ambrosioides* L. | Amaranthaceae | H | N | N | Helminthiasis | CIPD |
| Cartaxo et al. 2010 | Caatinga | *Chenopodium ambrosioides* L. | Amaranthaceae | H | N | N | Helminthiasis | CIPD |
| Cartaxo et al. 2010 | Caatinga | *Chenopodium ambrosioides* L. | Amaranthaceae | H | N | N | Helminthiasis | CIPD |
| Cartaxo et al. 2010 | Caatinga | *Chenopodium ambrosioides* L. | Amaranthaceae | H | N | N | Hematoma | SSNEC |
| Cartaxo et al. 2010 | Caatinga | *Chenopodium ambrosioides* L. | Amaranthaceae | H | N | N | Hematoma | SSNEC |
| Cartaxo et al. 2010 | Caatinga | *Chenopodium ambrosioides* L. | Amaranthaceae | H | N | N | Hematoma | SSNEC |
| Cartaxo et al. 2010 | Caatinga | *Chenopodium ambrosioides* L. | Amaranthaceae | H | N | N | Inflammation | SSNEC |
| Cartaxo et al. 2010 | Caatinga | *Chenopodium ambrosioides* L. | Amaranthaceae | H | N | N | Inflammation | SSNEC |
| Cartaxo et al. 2010 | Caatinga | *Chenopodium ambrosioides* L. | Amaranthaceae | H | N | N | Inflammation | SSNEC |
| Cartaxo et al. 2010 | Caatinga | *Chenopodium ambrosioides* L. | Amaranthaceae | H | N | N | Intestinal problems | DDS |
| Cartaxo et al. 2010 | Caatinga | *Chenopodium ambrosioides* L. | Amaranthaceae | H | N | N | Intestinal problems | DDS |
| Cartaxo et al. 2010 | Caatinga | *Chenopodium ambrosioides* L. | Amaranthaceae | H | N | N | Intestinal problems | DDS |
| Cartaxo et al. 2010 | Caatinga | *Chenopodium ambrosioides* L. | Amaranthaceae | H | N | N | Stomach problems | DDS |
| Cartaxo et al. 2010 | Caatinga | *Chenopodium ambrosioides* L. | Amaranthaceae | H | N | N | Stomach problems | DDS |
| Cartaxo et al. 2010 | Caatinga | *Chenopodium ambrosioides* L. | Amaranthaceae | H | N | N | Stomach problems | DDS |
| Cartaxo et al. 2010 | Caatinga | *Chenopodium ambrosioides* L. | Amaranthaceae | H | N | N | Ulcer | DDS |
| Cartaxo et al. 2010 | Caatinga | *Chenopodium ambrosioides* L. | Amaranthaceae | H | N | N | Ulcer | DDS |
| Cartaxo et al. 2010 | Caatinga | *Chenopodium ambrosioides* L. | Amaranthaceae | H | N | N | Ulcer | DDS |
| Cartaxo et al. 2010 | Caatinga | *Chenopodium ambrosioides* L. | Amaranthaceae | H | N | N | Wound | IPEC |
| Cartaxo et al. 2010 | Caatinga | *Chenopodium ambrosioides* L. | Amaranthaceae | H | N | N | Wound | IPEC |
| Cartaxo et al. 2010 | Caatinga | *Chenopodium ambrosioides* L. | Amaranthaceae | H | N | N | Wound | IPEC |
| Cartaxo et al. 2010 | Caatinga | *Chiococca alba* (L.) Hitchc. | Rubiaceae | T | N | N | Rheumatism | DMS |
| Cartaxo et al. 2010 | Caatinga | *Cissampelos glaberrima* A.St.-Hil. | Menispermaceae | H | N | N | Expectorant | SSNEC |
| Cartaxo et al. 2010 | Caatinga | *Cissus simsiana* Schult. & Schult.f. | Vitaceae | S | N | N | Diabetes | ENMD |
| Cartaxo et al. 2010 | Caatinga | *Citrullus vulgaris* Schrad. | Cucurbitaceae | H | E | E | Fever | SSNEC |
| Cartaxo et al. 2010 | Caatinga | *Citrullus vulgaris* Schrad. | Cucurbitaceae | H | E | E | Influenza | DRS |
| Cartaxo et al. 2010 | Caatinga | *Citrus aurantiifolia* (Christm.) Swingle | Rutaceae | T | E | E | Influenza | DRS |
| Cartaxo et al. 2010 | Caatinga | *Citrus aurantiifolia* (Christm.) Swingle | Rutaceae | T | E | E | Stomach pain/Diarrhea | SSNEC |
| Cartaxo et al. 2010 | Caatinga | *Citrus aurantiifolia* (Christm.) Swingle | Rutaceae | T | E | E | Teething in children | DDS |
| Cartaxo et al. 2010 | Caatinga | *Citrus sinensis* (L.) Osbeck | Rutaceae | T | E | E | Calming | MBD |
| Cartaxo et al. 2010 | Caatinga | *Citrus sinensis* (L.) Osbeck | Rutaceae | T | E | E | Calming | MBD |
| Cartaxo et al. 2010 | Caatinga | *Citrus sinensis* (L.) Osbeck | Rutaceae | T | E | E | Fever | SSNEC |
| Cartaxo et al. 2010 | Caatinga | *Citrus sinensis* (L.) Osbeck | Rutaceae | T | E | E | Fever | SSNEC |
| Cartaxo et al. 2010 | Caatinga | *Citrus sinensis* (L.) Osbeck | Rutaceae | T | E | E | Influenza | DRS |
| Cartaxo et al. 2010 | Caatinga | *Citrus sinensis* (L.) Osbeck | Rutaceae | T | E | E | Influenza | DRS |
| Cartaxo et al. 2010 | Caatinga | *Citrus sinensis* (L.) Osbeck | Rutaceae | T | E | E | Insomnia | DNS |
| Cartaxo et al. 2010 | Caatinga | *Citrus sinensis* (L.) Osbeck | Rutaceae | T | E | E | Insomnia | DNS |
| Cartaxo et al. 2010 | Caatinga | *Citrus sinensis* (L.) Osbeck | Rutaceae | T | E | E | Intestinal problems | DDS |
| Cartaxo et al. 2010 | Caatinga | *Citrus sinensis* (L.) Osbeck | Rutaceae | T | E | E | Intestinal problems | DDS |
| Cartaxo et al. 2010 | Caatinga | *Citrus sinensis* (L.) Osbeck | Rutaceae | T | E | E | Lack of apetite | SSNEC |
| Cartaxo et al. 2010 | Caatinga | *Citrus sinensis* (L.) Osbeck | Rutaceae | T | E | E | Lack of apetite | SSNEC |
| Cartaxo et al. 2010 | Caatinga | *Citrus sinensis* (L.) Osbeck | Rutaceae | T | E | E | Poor digestion | DDS |
| Cartaxo et al. 2010 | Caatinga | *Citrus sinensis* (L.) Osbeck | Rutaceae | T | E | E | Poor digestion | DDS |
| Cartaxo et al. 2010 | Caatinga | *Cnidoscolus quercifolius* Pohl | Euphorbiaceae | T | N | N | Antiseptic | CIPD |
| Cartaxo et al. 2010 | Caatinga | *Cnidoscolus quercifolius* Pohl | Euphorbiaceae | T | N | N | Antiseptic | CIPD |
| Cartaxo et al. 2010 | Caatinga | *Cnidoscolus quercifolius* Pohl | Euphorbiaceae | T | N | N | Gastritis | DDS |
| Cartaxo et al. 2010 | Caatinga | *Cnidoscolus quercifolius* Pohl | Euphorbiaceae | T | N | N | Gastritis | DDS |
| Cartaxo et al. 2010 | Caatinga | *Cnidoscolus quercifolius* Pohl | Euphorbiaceae | T | N | N | Poor digestion | DDS |
| Cartaxo et al. 2010 | Caatinga | *Cnidoscolus quercifolius* Pohl | Euphorbiaceae | T | N | N | Poor digestion | DDS |
| Cartaxo et al. 2010 | Caatinga | *Cnidoscolus quercifolius* Pohl | Euphorbiaceae | T | N | N | Toothache | DDS |
| Cartaxo et al. 2010 | Caatinga | *Cnidoscolus quercifolius* Pohl | Euphorbiaceae | T | N | N | Toothache | DDS |
| Cartaxo et al. 2010 | Caatinga | *Cnidoscolus quercifolius* Pohl | Euphorbiaceae | T | N | N | Ulcer | DDS |
| Cartaxo et al. 2010 | Caatinga | *Cnidoscolus quercifolius* Pohl | Euphorbiaceae | T | N | N | Ulcer | DDS |
| Cartaxo et al. 2010 | Caatinga | *Coffea arabica* L. | Rubiaceae | S | E | E | Fever | SSNEC |
| Cartaxo et al. 2010 | Caatinga | *Commiphora leptophloeos* (Mart.) J.B.Gillett | Burseraceae | T | N | N | Influenza | DRS |
| Cartaxo et al. 2010 | Caatinga | *Commiphora leptophloeos* (Mart.) J.B.Gillett | Burseraceae | T | N | N | Stomach pain/Diarrhea | SSNEC |
| Cartaxo et al. 2010 | Caatinga | *Commiphora leptophloeos* (Mart.) J.B.Gillett | Burseraceae | T | N | N | Wound | IPEC |
| Cartaxo et al. 2010 | Caatinga | *Cordia trichotoma* (Vell.) Arráb. ex Steud. | Boraginaceae | S | N | N | Anticoagulant | DCS |
| Cartaxo et al. 2010 | Caatinga | *Cordia trichotoma* (Vell.) Arráb. ex Steud. | Boraginaceae | S | N | N | Stomach pain/Diarrhea | SSNEC |
| Cartaxo et al. 2010 | Caatinga | *Cordia trichotoma* (Vell.) Arráb. ex Steud. | Boraginaceae | S | N | N | Ulcer | DDS |
| Cartaxo et al. 2010 | Caatinga | *Coriandrum sativum* L. | Apiaceae | H | E | E | Infant colic | SSNEC |
| Cartaxo et al. 2010 | Caatinga | *Coriandrum sativum* L. | Apiaceae | H | E | E | Menstrual cramps | DGS |
| Cartaxo et al. 2010 | Caatinga | *Coutarea hexandra* (Jacq.) K.Schum. | Rubiaceae | T | N | N | Expectorant | SSNEC |
| Cartaxo et al. 2010 | Caatinga | *Coutarea hexandra* (Jacq.) K.Schum. | Rubiaceae | T | N | N | Expectorant | SSNEC |
| Cartaxo et al. 2010 | Caatinga | *Coutarea hexandra* (Jacq.) K.Schum. | Rubiaceae | T | N | N | Headache | SSNEC |
| Cartaxo et al. 2010 | Caatinga | *Coutarea hexandra* (Jacq.) K.Schum. | Rubiaceae | T | N | N | Headache | SSNEC |
| Cartaxo et al. 2010 | Caatinga | *Coutarea hexandra* (Jacq.) K.Schum. | Rubiaceae | T | N | N | Influenza | DRS |
| Cartaxo et al. 2010 | Caatinga | *Coutarea hexandra* (Jacq.) K.Schum. | Rubiaceae | T | N | N | Influenza | DRS |
| Cartaxo et al. 2010 | Caatinga | *Coutarea hexandra* (Jacq.) K.Schum. | Rubiaceae | T | N | N | Pulmonary problems | DRS |
| Cartaxo et al. 2010 | Caatinga | *Coutarea hexandra* (Jacq.) K.Schum. | Rubiaceae | T | N | N | Pulmonary problems | DRS |
| Cartaxo et al. 2010 | Caatinga | *Croton blanchetianus* Baill. | Euphorbiaceae | S | N | N | Depurative | DBBO |
| Cartaxo et al. 2010 | Caatinga | *Croton blanchetianus* Baill. | Euphorbiaceae | S | N | N | Depurative | DBBO |
| Cartaxo et al. 2010 | Caatinga | *Croton blanchetianus* Baill. | Euphorbiaceae | S | N | N | Dysentry | CIPD |
| Cartaxo et al. 2010 | Caatinga | *Croton blanchetianus* Baill. | Euphorbiaceae | S | N | N | Dysentry | CIPD |
| Cartaxo et al. 2010 | Caatinga | *Croton blanchetianus* Baill. | Euphorbiaceae | S | N | N | Poor digestion | DDS |
| Cartaxo et al. 2010 | Caatinga | *Croton blanchetianus* Baill. | Euphorbiaceae | S | N | N | Poor digestion | DDS |
| Cartaxo et al. 2010 | Caatinga | *Croton blanchetianus* Baill. | Euphorbiaceae | S | N | N | Stomach pain/Diarrhea | SSNEC |
| Cartaxo et al. 2010 | Caatinga | *Croton blanchetianus* Baill. | Euphorbiaceae | S | N | N | Stomach pain/Diarrhea | SSNEC |
| Cartaxo et al. 2010 | Caatinga | *Croton blanchetianus* Baill. | Euphorbiaceae | S | N | N | Stomach pain/Diarrhea | SSNEC |
| Cartaxo et al. 2010 | Caatinga | *Croton blanchetianus* Baill. | Euphorbiaceae | S | N | N | Stomach pain/Diarrhea | SSNEC |
| Cartaxo et al. 2010 | Caatinga | *Croton blanchetianus* Baill. | Euphorbiaceae | S | N | N | Stomach problems | DDS |
| Cartaxo et al. 2010 | Caatinga | *Croton blanchetianus* Baill. | Euphorbiaceae | S | N | N | Stomach problems | DDS |
| Cartaxo et al. 2010 | Caatinga | *Croton heliotropiifolius* Kunth | Euphorbiaceae | S | N | N | Headache | SSNEC |
| Cartaxo et al. 2010 | Caatinga | *Croton heliotropiifolius* Kunth | Euphorbiaceae | S | N | N | Headache | SSNEC |
| Cartaxo et al. 2010 | Caatinga | *Croton heliotropiifolius* Kunth | Euphorbiaceae | S | N | N | Influenza | DRS |
| Cartaxo et al. 2010 | Caatinga | *Croton heliotropiifolius* Kunth | Euphorbiaceae | S | N | N | Influenza | DRS |
| Cartaxo et al. 2010 | Caatinga | *Croton heliotropiifolius* Kunth | Euphorbiaceae | S | N | N | Poor digestion | DDS |
| Cartaxo et al. 2010 | Caatinga | *Croton heliotropiifolius* Kunth | Euphorbiaceae | S | N | N | Poor digestion | DDS |
| Cartaxo et al. 2010 | Caatinga | *Croton heliotropiifolius* Kunth | Euphorbiaceae | S | N | N | Stomach pain/Diarrhea | SSNEC |
| Cartaxo et al. 2010 | Caatinga | *Croton heliotropiifolius* Kunth | Euphorbiaceae | S | N | N | Stomach pain/Diarrhea | SSNEC |
| Cartaxo et al. 2010 | Caatinga | *Croton heliotropiifolius* Kunth | Euphorbiaceae | S | N | N | Stomach problems | DDS |
| Cartaxo et al. 2010 | Caatinga | *Croton heliotropiifolius* Kunth | Euphorbiaceae | S | N | N | Stomach problems | DDS |
| Cartaxo et al. 2010 | Caatinga | *Curcuma longa* L. | Zingiberaceae | H | E | E | Throat problems | DRS |
| Cartaxo et al. 2010 | Caatinga | *Curcuma longa* L. | Zingiberaceae | H | E | E | Throat problems | DRS |
| Cartaxo et al. 2010 | Caatinga | *Cymbopogon citratus* (DC.) Stapf | Poaceae | H | E | E | Calming | MBD |
| Cartaxo et al. 2010 | Caatinga | *Cymbopogon citratus* (DC.) Stapf | Poaceae | H | E | E | Fever | SSNEC |
| Cartaxo et al. 2010 | Caatinga | *Cymbopogon citratus* (DC.) Stapf | Poaceae | H | E | E | Headache | SSNEC |
| Cartaxo et al. 2010 | Caatinga | *Cymbopogon citratus* (DC.) Stapf | Poaceae | H | E | E | Hypertension | DCS |
| Cartaxo et al. 2010 | Caatinga | *Cymbopogon citratus* (DC.) Stapf | Poaceae | H | E | E | Influenza | DRS |
| Cartaxo et al. 2010 | Caatinga | *Cymbopogon citratus* (DC.) Stapf | Poaceae | H | E | E | Lack of apetite | SSNEC |
| Cartaxo et al. 2010 | Caatinga | *Cymbopogon citratus* (DC.) Stapf | Poaceae | H | E | E | Poor digestion | DDS |
| Cartaxo et al. 2010 | Caatinga | *Cymbopogon citratus* (DC.) Stapf | Poaceae | H | E | E | Stomach pain/Diarrhea | SSNEC |
| Cartaxo et al. 2010 | Caatinga | *Echtrus mexicanus* (L.) Nieuwl. | Papaveraceae | H | E | E | Cough | SSNEC |
| Cartaxo et al. 2010 | Caatinga | *Echtrus mexicanus* (L.) Nieuwl. | Papaveraceae | H | E | E | Expectorant | SSNEC |
| Cartaxo et al. 2010 | Caatinga | *Egletes viscosa* (L.) Less. | Asteraceae | H | N | N | Dysentry | CIPD |
| Cartaxo et al. 2010 | Caatinga | *Egletes viscosa* (L.) Less. | Asteraceae | H | N | N | Dysentry | CIPD |
| Cartaxo et al. 2010 | Caatinga | *Egletes viscosa* (L.) Less. | Asteraceae | H | N | N | Flatulence | SSNEC |
| Cartaxo et al. 2010 | Caatinga | *Egletes viscosa* (L.) Less. | Asteraceae | H | N | N | Flatulence | SSNEC |
| Cartaxo et al. 2010 | Caatinga | *Egletes viscosa* (L.) Less. | Asteraceae | H | N | N | Intestinal problems | DDS |
| Cartaxo et al. 2010 | Caatinga | *Egletes viscosa* (L.) Less. | Asteraceae | H | N | N | Intestinal problems | DDS |
| Cartaxo et al. 2010 | Caatinga | *Egletes viscosa* (L.) Less. | Asteraceae | H | N | N | Poor digestion | DDS |
| Cartaxo et al. 2010 | Caatinga | *Egletes viscosa* (L.) Less. | Asteraceae | H | N | N | Poor digestion | DDS |
| Cartaxo et al. 2010 | Caatinga | *Egletes viscosa* (L.) Less. | Asteraceae | H | N | N | Stomach pain/Diarrhea | SSNEC |
| Cartaxo et al. 2010 | Caatinga | *Egletes viscosa* (L.) Less. | Asteraceae | H | N | N | Stomach pain/Diarrhea | SSNEC |
| Cartaxo et al. 2010 | Caatinga | *Egletes viscosa* (L.) Less. | Asteraceae | H | N | N | Stomach problems | DDS |
| Cartaxo et al. 2010 | Caatinga | *Egletes viscosa* (L.) Less. | Asteraceae | H | N | N | Stomach problems | DDS |
| Cartaxo et al. 2010 | Caatinga | *Egletes viscosa* (L.) Less. | Asteraceae | H | N | N | Ulcer | DDS |
| Cartaxo et al. 2010 | Caatinga | *Egletes viscosa* (L.) Less. | Asteraceae | H | N | N | Ulcer | DDS |
| Cartaxo et al. 2010 | Caatinga | *Enterolobium contortisiliquum* (Vell.) Morong | Fabaceae | T | N | N | Inflammation | SSNEC |
| Cartaxo et al. 2010 | Caatinga | *Enterolobium contortisiliquum* (Vell.) Morong | Fabaceae | T | N | N | Inflammation | SSNEC |
| Cartaxo et al. 2010 | Caatinga | *Enterolobium contortisiliquum* (Vell.) Morong | Fabaceae | T | N | N | Prostate problems | DGS |
| Cartaxo et al. 2010 | Caatinga | *Enterolobium contortisiliquum* (Vell.) Morong | Fabaceae | T | N | N | Prostate problems | DGS |
| Cartaxo et al. 2010 | Caatinga | *Enterolobium contortisiliquum* (Vell.) Morong | Fabaceae | T | N | N | Respiratory problems | DRS |
| Cartaxo et al. 2010 | Caatinga | *Enterolobium contortisiliquum* (Vell.) Morong | Fabaceae | T | N | N | Respiratory problems | DRS |
| Cartaxo et al. 2010 | Caatinga | *Enterolobium contortisiliquum* (Vell.) Morong | Fabaceae | T | N | N | Rhinitis | DRS |
| Cartaxo et al. 2010 | Caatinga | *Enterolobium contortisiliquum* (Vell.) Morong | Fabaceae | T | N | N | Rhinitis | DRS |
| Cartaxo et al. 2010 | Caatinga | *Enterolobium contortisiliquum* (Vell.) Morong | Fabaceae | T | N | N | Sinusitis | DRS |
| Cartaxo et al. 2010 | Caatinga | *Enterolobium contortisiliquum* (Vell.) Morong | Fabaceae | T | N | N | Sinusitis | DRS |
| Cartaxo et al. 2010 | Caatinga | *Eucalyptus globulus* Labill. | Myrtaceae | T | E | E | Asthma | DRS |
| Cartaxo et al. 2010 | Caatinga | *Eucalyptus globulus* Labill. | Myrtaceae | T | E | E | Bad breath | SSNEC |
| Cartaxo et al. 2010 | Caatinga | *Eucalyptus globulus* Labill. | Myrtaceae | T | E | E | Fever | SSNEC |
| Cartaxo et al. 2010 | Caatinga | *Eucalyptus globulus* Labill. | Myrtaceae | T | E | E | Headache | SSNEC |
| Cartaxo et al. 2010 | Caatinga | *Eucalyptus globulus* Labill. | Myrtaceae | T | E | E | Influenza | DRS |
| Cartaxo et al. 2010 | Caatinga | *Eucalyptus globulus* Labill. | Myrtaceae | T | E | E | Nasal congestion | DRS |
| Cartaxo et al. 2010 | Caatinga | *Eucalyptus globulus* Labill. | Myrtaceae | T | E | E | Respiratory problems | DRS |
| Cartaxo et al. 2010 | Caatinga | *Eucalyptus globulus* Labill. | Myrtaceae | T | E | E | Sinusitis | DRS |
| Cartaxo et al. 2010 | Caatinga | *Euphorbia phosphorea* Mart. | Euphorbiaceae | S | N | N | Anticoagulant | DCS |
| Cartaxo et al. 2010 | Caatinga | *Euphorbia phosphorea* Mart. | Euphorbiaceae | S | N | N | Inflammation | SSNEC |
| Cartaxo et al. 2010 | Caatinga | *Genipa americana* L. | Rubiaceae | T | N | N | Fracture | IPEC |
| Cartaxo et al. 2010 | Caatinga | *Genipa americana* L. | Rubiaceae | T | N | N | Fracture | IPEC |
| Cartaxo et al. 2010 | Caatinga | *Genipa americana* L. | Rubiaceae | T | N | N | Hematoma | SSNEC |
| Cartaxo et al. 2010 | Caatinga | *Genipa americana* L. | Rubiaceae | T | N | N | Hematoma | SSNEC |
| Cartaxo et al. 2010 | Caatinga | *Genipa americana* L. | Rubiaceae | T | N | N | Torsion | SSNEC |
| Cartaxo et al. 2010 | Caatinga | *Genipa americana* L. | Rubiaceae | T | N | N | Torsion | SSNEC |
| Cartaxo et al. 2010 | Caatinga | *Genipa americana* L. | Rubiaceae | T | N | N | Wound | IPEC |
| Cartaxo et al. 2010 | Caatinga | *Genipa americana* L. | Rubiaceae | T | N | N | Wound | IPEC |
| Cartaxo et al. 2010 | Caatinga | *Gossypium hirsutum* L. | Malvaceae | S | E | E | Inflammation | SSNEC |
| Cartaxo et al. 2010 | Caatinga | *Gossypium hirsutum* L. | Malvaceae | S | E | E | Inflammation | SSNEC |
| Cartaxo et al. 2010 | Caatinga | *Gossypium hirsutum* L. | Malvaceae | S | E | E | Inflammation of the uterus | DGS |
| Cartaxo et al. 2010 | Caatinga | *Gossypium hirsutum* L. | Malvaceae | S | E | E | Inflammation of the uterus | DGS |
| Cartaxo et al. 2010 | Caatinga | *Guatteria australis* A.St.-Hil. | Annonaceae | T | N | E | Pain | SSNEC |
| Cartaxo et al. 2010 | Caatinga | *Guatteria australis* A.St.-Hil. | Annonaceae | T | N | E | Stroke | DCS |
| Cartaxo et al. 2010 | Caatinga | *Handroanthus impetiginosus* Mattos | Bignoniaceae | T | N | N | Anticoagulant | DCS |
| Cartaxo et al. 2010 | Caatinga | *Handroanthus impetiginosus* Mattos | Bignoniaceae | T | N | N | Conjunctivitis | DEA |
| Cartaxo et al. 2010 | Caatinga | *Handroanthus impetiginosus* Mattos | Bignoniaceae | T | N | N | Influenza | DRS |
| Cartaxo et al. 2010 | Caatinga | *Handroanthus impetiginosus* Mattos | Bignoniaceae | T | N | N | Nasal congestion | DRS |
| Cartaxo et al. 2010 | Caatinga | *Handroanthus impetiginosus* Mattos | Bignoniaceae | T | N | N | Pain | SSNEC |
| Cartaxo et al. 2010 | Caatinga | *Handroanthus impetiginosus* Mattos | Bignoniaceae | T | N | N | Stomach problems | DDS |
| Cartaxo et al. 2010 | Caatinga | *Handroanthus impetiginosus* Mattos | Bignoniaceae | T | N | N | Wound | IPEC |
| Cartaxo et al. 2010 | Caatinga | *Helianthus annuus* L. | Asteraceae | H | E | E | Anticoagulant | DCS |
| Cartaxo et al. 2010 | Caatinga | *Helianthus annuus* L. | Asteraceae | H | E | E | Anticoagulant | DCS |
| Cartaxo et al. 2010 | Caatinga | *Helianthus annuus* L. | Asteraceae | H | E | E | Headache | SSNEC |
| Cartaxo et al. 2010 | Caatinga | *Helianthus annuus* L. | Asteraceae | H | E | E | Headache | SSNEC |
| Cartaxo et al. 2010 | Caatinga | *Helianthus annuus* L. | Asteraceae | H | E | E | Stroke | DCS |
| Cartaxo et al. 2010 | Caatinga | *Helianthus annuus* L. | Asteraceae | H | E | E | Stroke | DCS |
| Cartaxo et al. 2010 | Caatinga | *Helianthus annuus* L. | Asteraceae | H | E | E | Thrombosis | DCS |
| Cartaxo et al. 2010 | Caatinga | *Helianthus annuus* L. | Asteraceae | H | E | E | Thrombosis | DCS |
| Cartaxo et al. 2010 | Caatinga | *Hybanthus calceolaria* (L.) Oken | Violaceae | H | N | N | Teething in children | DDS |
| Cartaxo et al. 2010 | Caatinga | *Hymenaea courbaril* L. | Fabaceae | T | N | N | Anemia | DBBO |
| Cartaxo et al. 2010 | Caatinga | *Hymenaea courbaril* L. | Fabaceae | T | N | N | Anemia | DBBO |
| Cartaxo et al. 2010 | Caatinga | *Hymenaea courbaril* L. | Fabaceae | T | N | N | Anemia | DBBO |
| Cartaxo et al. 2010 | Caatinga | *Hymenaea courbaril* L. | Fabaceae | T | N | N | Anticoagulant | DCS |
| Cartaxo et al. 2010 | Caatinga | *Hymenaea courbaril* L. | Fabaceae | T | N | N | Anticoagulant | DCS |
| Cartaxo et al. 2010 | Caatinga | *Hymenaea courbaril* L. | Fabaceae | T | N | N | Anticoagulant | DCS |
| Cartaxo et al. 2010 | Caatinga | *Hymenaea courbaril* L. | Fabaceae | T | N | N | Bronchitis | DRS |
| Cartaxo et al. 2010 | Caatinga | *Hymenaea courbaril* L. | Fabaceae | T | N | N | Bronchitis | DRS |
| Cartaxo et al. 2010 | Caatinga | *Hymenaea courbaril* L. | Fabaceae | T | N | N | Bronchitis | DRS |
| Cartaxo et al. 2010 | Caatinga | *Hymenaea courbaril* L. | Fabaceae | T | N | N | Expectorant | SSNEC |
| Cartaxo et al. 2010 | Caatinga | *Hymenaea courbaril* L. | Fabaceae | T | N | N | Expectorant | SSNEC |
| Cartaxo et al. 2010 | Caatinga | *Hymenaea courbaril* L. | Fabaceae | T | N | N | Expectorant | SSNEC |
| Cartaxo et al. 2010 | Caatinga | *Hymenaea courbaril* L. | Fabaceae | T | N | N | Herpes labialis | CIPD |
| Cartaxo et al. 2010 | Caatinga | *Hymenaea courbaril* L. | Fabaceae | T | N | N | Herpes labialis | CIPD |
| Cartaxo et al. 2010 | Caatinga | *Hymenaea courbaril* L. | Fabaceae | T | N | N | Herpes labialis (cold sores) | CIPD |
| Cartaxo et al. 2010 | Caatinga | *Hymenaea courbaril* L. | Fabaceae | T | N | N | Influenza | DRS |
| Cartaxo et al. 2010 | Caatinga | *Hymenaea courbaril* L. | Fabaceae | T | N | N | Influenza | DRS |
| Cartaxo et al. 2010 | Caatinga | *Hymenaea courbaril* L. | Fabaceae | T | N | N | Influenza | DRS |
| Cartaxo et al. 2010 | Caatinga | *Hymenaea courbaril* L. | Fabaceae | T | N | N | Kidney problems | DGS |
| Cartaxo et al. 2010 | Caatinga | *Hymenaea courbaril* L. | Fabaceae | T | N | N | Kidney problems | DGS |
| Cartaxo et al. 2010 | Caatinga | *Hymenaea courbaril* L. | Fabaceae | T | N | N | Kidney problems | DGS |
| Cartaxo et al. 2010 | Caatinga | *Hymenaea courbaril* L. | Fabaceae | T | N | N | Leukemia | NEO |
| Cartaxo et al. 2010 | Caatinga | *Hymenaea courbaril* L. | Fabaceae | T | N | N | Leukemia | NEO |
| Cartaxo et al. 2010 | Caatinga | *Hymenaea courbaril* L. | Fabaceae | T | N | N | Leukemia | NEO |
| Cartaxo et al. 2010 | Caatinga | *Hymenaea courbaril* L. | Fabaceae | T | N | N | Lice | CIPD |
| Cartaxo et al. 2010 | Caatinga | *Hymenaea courbaril* L. | Fabaceae | T | N | N | Lice | CIPD |
| Cartaxo et al. 2010 | Caatinga | *Hymenaea courbaril* L. | Fabaceae | T | N | N | Lice | CIPD |
| Cartaxo et al. 2010 | Caatinga | *Hymenaea courbaril* L. | Fabaceae | T | N | N | Prostate problems | DGS |
| Cartaxo et al. 2010 | Caatinga | *Hymenaea courbaril* L. | Fabaceae | T | N | N | Prostate problems | DGS |
| Cartaxo et al. 2010 | Caatinga | *Hymenaea courbaril* L. | Fabaceae | T | N | N | Prostate problems | DGS |
| Cartaxo et al. 2010 | Caatinga | *Hymenaea courbaril* L. | Fabaceae | T | N | N | Pulmonary problems | DRS |
| Cartaxo et al. 2010 | Caatinga | *Hymenaea courbaril* L. | Fabaceae | T | N | N | Pulmonary problems | DRS |
| Cartaxo et al. 2010 | Caatinga | *Hymenaea courbaril* L. | Fabaceae | T | N | N | Pulmonary problems | DRS |
| Cartaxo et al. 2010 | Caatinga | *Hymenaea courbaril* L. | Fabaceae | T | N | N | Stomach problems | DDS |
| Cartaxo et al. 2010 | Caatinga | *Hymenaea courbaril* L. | Fabaceae | T | N | N | Stomach problems | DDS |
| Cartaxo et al. 2010 | Caatinga | *Hymenaea courbaril* L. | Fabaceae | T | N | N | Stomach problems | DDS |
| Cartaxo et al. 2010 | Caatinga | *Hymenaea courbaril* L. | Fabaceae | T | N | N | Throat problems | DRS |
| Cartaxo et al. 2010 | Caatinga | *Hymenaea courbaril* L. | Fabaceae | T | N | N | Throat problems | DRS |
| Cartaxo et al. 2010 | Caatinga | *Hymenaea courbaril* L. | Fabaceae | T | N | N | Throat problems | DRS |
| Cartaxo et al. 2010 | Caatinga | *Ipomoea batatas* (L.) Lam. | Convolvulaceae | H | E | E | Diabetes | ENMD |
| Cartaxo et al. 2010 | Caatinga | *Ipomoea batatas* (L.) Lam. | Convolvulaceae | H | E | E | High cholesterol | ENMD |
| Cartaxo et al. 2010 | Caatinga | *Kalanchoe crenata* (Andrews) Haw. | Crassulaceae | H | E | E | Anticoagulant | DCS |
| Cartaxo et al. 2010 | Caatinga | *Kalanchoe crenata* (Andrews) Haw. | Crassulaceae | H | E | E | Anticoagulant | DCS |
| Cartaxo et al. 2010 | Caatinga | *Kalanchoe crenata* (Andrews) Haw. | Crassulaceae | H | E | E | Cough | SSNEC |
| Cartaxo et al. 2010 | Caatinga | *Kalanchoe crenata* (Andrews) Haw. | Crassulaceae | H | E | E | Cough | SSNEC |
| Cartaxo et al. 2010 | Caatinga | *Kalanchoe crenata* (Andrews) Haw. | Crassulaceae | H | E | E | Depurative | DBBO |
| Cartaxo et al. 2010 | Caatinga | *Kalanchoe crenata* (Andrews) Haw. | Crassulaceae | H | E | E | Depurative | DBBO |
| Cartaxo et al. 2010 | Caatinga | *Kalanchoe crenata* (Andrews) Haw. | Crassulaceae | H | E | E | Expectorant | SSNEC |
| Cartaxo et al. 2010 | Caatinga | *Kalanchoe crenata* (Andrews) Haw. | Crassulaceae | H | E | E | Expectorant | SSNEC |
| Cartaxo et al. 2010 | Caatinga | *Kalanchoe crenata* (Andrews) Haw. | Crassulaceae | H | E | E | Inflammation | SSNEC |
| Cartaxo et al. 2010 | Caatinga | *Kalanchoe crenata* (Andrews) Haw. | Crassulaceae | H | E | E | Inflammation | SSNEC |
| Cartaxo et al. 2010 | Caatinga | *Kalanchoe crenata* (Andrews) Haw. | Crassulaceae | H | E | E | Inflammation of the uterus | DGS |
| Cartaxo et al. 2010 | Caatinga | *Kalanchoe crenata* (Andrews) Haw. | Crassulaceae | H | E | E | Inflammation of the uterus | DGS |
| Cartaxo et al. 2010 | Caatinga | *Kalanchoe crenata* (Andrews) Haw. | Crassulaceae | H | E | E | Influenza | DRS |
| Cartaxo et al. 2010 | Caatinga | *Kalanchoe crenata* (Andrews) Haw. | Crassulaceae | H | E | E | Influenza | DRS |
| Cartaxo et al. 2010 | Caatinga | *Kalanchoe crenata* (Andrews) Haw. | Crassulaceae | H | E | E | Pain | SSNEC |
| Cartaxo et al. 2010 | Caatinga | *Kalanchoe crenata* (Andrews) Haw. | Crassulaceae | H | E | E | Pain | SSNEC |
| Cartaxo et al. 2010 | Caatinga | *Kalanchoe crenata* (Andrews) Haw. | Crassulaceae | H | E | E | Wound | IPEC |
| Cartaxo et al. 2010 | Caatinga | *Kalanchoe crenata* (Andrews) Haw. | Crassulaceae | H | E | E | Wound | IPEC |
| Cartaxo et al. 2010 | Caatinga | *Leonotis nepetifolia* (L.) R.Br. | Lamiaceae | H | N | N | Poor digestion | DDS |
| Cartaxo et al. 2010 | Caatinga | *Licania rigida* Benth. | Chrysobalanaceae | T | N | N | Dysentry | CIPD |
| Cartaxo et al. 2010 | Caatinga | *Licania rigida* Benth. | Chrysobalanaceae | T | N | N | Stomach pain/Diarrhea | SSNEC |
| Cartaxo et al. 2010 | Caatinga | *Licania rigida* Benth. | Chrysobalanaceae | T | N | N | Stomach pain/Diarrhea | SSNEC |
| Cartaxo et al. 2010 | Caatinga | *Lippia alba* (Mill.) N.E.Br. | Verbenaceae | S | N | N | Calming | MBD |
| Cartaxo et al. 2010 | Caatinga | *Lippia alba* (Mill.) N.E.Br. | Verbenaceae | S | N | N | Calming | MBD |
| Cartaxo et al. 2010 | Caatinga | *Lippia alba* (Mill.) N.E.Br. | Verbenaceae | S | N | N | Intestinal problems | DDS |
| Cartaxo et al. 2010 | Caatinga | *Lippia alba* (Mill.) N.E.Br. | Verbenaceae | S | N | N | Intestinal problems | DDS |
| Cartaxo et al. 2010 | Caatinga | *Lippia alba* (Mill.) N.E.Br. | Verbenaceae | S | N | N | Lack of apetite | SSNEC |
| Cartaxo et al. 2010 | Caatinga | *Lippia alba* (Mill.) N.E.Br. | Verbenaceae | S | N | N | Lack of apetite | SSNEC |
| Cartaxo et al. 2010 | Caatinga | *Lippia alba* (Mill.) N.E.Br. | Verbenaceae | S | N | N | Poor digestion | DDS |
| Cartaxo et al. 2010 | Caatinga | *Lippia alba* (Mill.) N.E.Br. | Verbenaceae | S | N | N | Poor digestion | DDS |
| Cartaxo et al. 2010 | Caatinga | *Lippia alba* (Mill.) N.E.Br. | Verbenaceae | S | N | N | Stomach pain/Diarrhea | SSNEC |
| Cartaxo et al. 2010 | Caatinga | *Lippia alba* (Mill.) N.E.Br. | Verbenaceae | S | N | N | Stomach pain/Diarrhea | SSNEC |
| Cartaxo et al. 2010 | Caatinga | *Lippia alba* (Mill.) N.E.Br. | Verbenaceae | S | N | N | Stomach pain/Diarrhea | SSNEC |
| Cartaxo et al. 2010 | Caatinga | *Lippia alba* (Mill.) N.E.Br. | Verbenaceae | S | N | N | Stomach pain/Diarrhea | SSNEC |
| Cartaxo et al. 2010 | Caatinga | *Malpighia glabra* L. | Malpighiaceae | T | E | E | Dengue fever | CIPD |
| Cartaxo et al. 2010 | Caatinga | *Mangifera indica* L. | Anacardiaceae | T | E | E | Fever | SSNEC |
| Cartaxo et al. 2010 | Caatinga | *Manihot esculenta* Crantz | Euphorbiaceae | S | N | E | Inflammation | SSNEC |
| Cartaxo et al. 2010 | Caatinga | *Matricaria chamomilla* L. | Asteraceae | H | E | E | Calming | MBD |
| Cartaxo et al. 2010 | Caatinga | *Matricaria chamomilla* L. | Asteraceae | H | E | E | Calming | MBD |
| Cartaxo et al. 2010 | Caatinga | *Mauritia flexuosa* L.f. | Arecaceae | T | N | N | Apendicitis | DDS |
| Cartaxo et al. 2010 | Caatinga | *Maytenus rigida* Mart. | Celastraceae | T | N | N | Kidney problems | DGS |
| Cartaxo et al. 2010 | Caatinga | *Maytenus rigida* Mart. | Celastraceae | T | N | N | Kidney problems | DGS |
| Cartaxo et al. 2010 | Caatinga | *Mentha × piperita* L. | Lamiaceae | H | E | E | Pain | SSNEC |
| Cartaxo et al. 2010 | Caatinga | *Mentha × villosa* Huds. | Lamiaceae | H | E | E | Earache | DEMP |
| Cartaxo et al. 2010 | Caatinga | *Mentha × villosa* Huds. | Lamiaceae | H | E | E | Fever | SSNEC |
| Cartaxo et al. 2010 | Caatinga | *Mentha × villosa* Huds. | Lamiaceae | H | E | E | Headache | SSNEC |
| Cartaxo et al. 2010 | Caatinga | *Mentha × villosa* Huds. | Lamiaceae | H | E | E | Helminthiasis | CIPD |
| Cartaxo et al. 2010 | Caatinga | *Mentha × villosa* Huds. | Lamiaceae | H | E | E | Inflammation | SSNEC |
| Cartaxo et al. 2010 | Caatinga | *Mentha × villosa* Huds. | Lamiaceae | H | E | E | Influenza | DRS |
| Cartaxo et al. 2010 | Caatinga | *Mentha × villosa* Huds. | Lamiaceae | H | E | E | Intestinal infection | CIPD |
| Cartaxo et al. 2010 | Caatinga | *Mentha × villosa* Huds. | Lamiaceae | H | E | E | Menstrual cramps | DGS |
| Cartaxo et al. 2010 | Caatinga | *Mentha × villosa* Huds. | Lamiaceae | H | E | E | Migraine | DNS |
| Cartaxo et al. 2010 | Caatinga | *Mentha × villosa* Huds. | Lamiaceae | H | E | E | Ophthalmological problems | DEA |
| Cartaxo et al. 2010 | Caatinga | *Mentha × villosa* Huds. | Lamiaceae | H | E | E | Pain | SSNEC |
| Cartaxo et al. 2010 | Caatinga | *Mentha × villosa* Huds. | Lamiaceae | H | E | E | Poor digestion | DDS |
| Cartaxo et al. 2010 | Caatinga | *Mentha × villosa* Huds. | Lamiaceae | H | E | E | Sinusitis | DRS |
| Cartaxo et al. 2010 | Caatinga | *Mentha × villosa* Huds. | Lamiaceae | H | E | E | Stroke | DCS |
| Cartaxo et al. 2010 | Caatinga | *Mimosa caesalpiniifolia* Benth. | Fabaceae | T | N | N | Inflammation | SSNEC |
| Cartaxo et al. 2010 | Caatinga | *Mimosa tenuiflora* (Willd.) Poir. | Fabaceae | S | N | N | Antiseptic | CIPD |
| Cartaxo et al. 2010 | Caatinga | *Mimosa tenuiflora* (Willd.) Poir. | Fabaceae | S | N | N | Inflammation | SSNEC |
| Cartaxo et al. 2010 | Caatinga | *Mimosa tenuiflora* (Willd.) Poir. | Fabaceae | S | N | N | Tooth inflammation | DDS |
| Cartaxo et al. 2010 | Caatinga | *Mimosa tenuiflora* (Willd.) Poir. | Fabaceae | S | N | N | Toothache | DDS |
| Cartaxo et al. 2010 | Caatinga | *Mimosa tenuiflora* (Willd.) Poir. | Fabaceae | S | N | N | Wound | IPEC |
| Cartaxo et al. 2010 | Caatinga | *Musa paradisiaca* L. | Musaceae | H | E | E | Asthma | DRS |
| Cartaxo et al. 2010 | Caatinga | *Musa paradisiaca* L. | Musaceae | H | E | E | Asthma | DRS |
| Cartaxo et al. 2010 | Caatinga | *Musa paradisiaca* L. | Musaceae | H | E | E | Influenza | DRS |
| Cartaxo et al. 2010 | Caatinga | *Musa paradisiaca* L. | Musaceae | H | E | E | Influenza | DRS |
| Cartaxo et al. 2010 | Caatinga | *Musa paradisiaca* L. | Musaceae | H | E | E | Urinary problems | DGS |
| Cartaxo et al. 2010 | Caatinga | *Musa paradisiaca* L. | Musaceae | H | E | E | Urinary problems | DGS |
| Cartaxo et al. 2010 | Caatinga | *Myracrodruon urundeuva* Allemão | Anacardiaceae | T | N | N | Antiseptic | CIPD |
| Cartaxo et al. 2010 | Caatinga | *Myracrodruon urundeuva* Allemão | Anacardiaceae | T | N | N | Antiseptic | CIPD |
| Cartaxo et al. 2010 | Caatinga | *Myracrodruon urundeuva* Allemão | Anacardiaceae | T | N | N | Cancer | NEO |
| Cartaxo et al. 2010 | Caatinga | *Myracrodruon urundeuva* Allemão | Anacardiaceae | T | N | N | Cancer | NEO |
| Cartaxo et al. 2010 | Caatinga | *Myracrodruon urundeuva* Allemão | Anacardiaceae | T | N | N | Expectorant | SSNEC |
| Cartaxo et al. 2010 | Caatinga | *Myracrodruon urundeuva* Allemão | Anacardiaceae | T | N | N | Expectorant | SSNEC |
| Cartaxo et al. 2010 | Caatinga | *Myracrodruon urundeuva* Allemão | Anacardiaceae | T | N | N | Headache | SSNEC |
| Cartaxo et al. 2010 | Caatinga | *Myracrodruon urundeuva* Allemão | Anacardiaceae | T | N | N | Headache | SSNEC |
| Cartaxo et al. 2010 | Caatinga | *Myracrodruon urundeuva* Allemão | Anacardiaceae | T | N | N | Infection | CIPD |
| Cartaxo et al. 2010 | Caatinga | *Myracrodruon urundeuva* Allemão | Anacardiaceae | T | N | N | Infection | CIPD |
| Cartaxo et al. 2010 | Caatinga | *Myracrodruon urundeuva* Allemão | Anacardiaceae | T | N | N | Inflammation | SSNEC |
| Cartaxo et al. 2010 | Caatinga | *Myracrodruon urundeuva* Allemão | Anacardiaceae | T | N | N | Inflammation | SSNEC |
| Cartaxo et al. 2010 | Caatinga | *Myracrodruon urundeuva* Allemão | Anacardiaceae | T | N | N | Inflammation of external organs | SSNEC |
| Cartaxo et al. 2010 | Caatinga | *Myracrodruon urundeuva* Allemão | Anacardiaceae | T | N | N | Inflammation of external organs | SSNEC |
| Cartaxo et al. 2010 | Caatinga | *Myracrodruon urundeuva* Allemão | Anacardiaceae | T | N | N | Inflammation of internal organs | SSNEC |
| Cartaxo et al. 2010 | Caatinga | *Myracrodruon urundeuva* Allemão | Anacardiaceae | T | N | N | Inflammation of internal organs | SSNEC |
| Cartaxo et al. 2010 | Caatinga | *Myracrodruon urundeuva* Allemão | Anacardiaceae | T | N | N | Inflammation of internal organs | SSNEC |
| Cartaxo et al. 2010 | Caatinga | *Myracrodruon urundeuva* Allemão | Anacardiaceae | T | N | N | Inflammation of internal organs | SSNEC |
| Cartaxo et al. 2010 | Caatinga | *Myracrodruon urundeuva* Allemão | Anacardiaceae | T | N | N | Inflammation of the female organs | DGS |
| Cartaxo et al. 2010 | Caatinga | *Myracrodruon urundeuva* Allemão | Anacardiaceae | T | N | N | Inflammation of the female organs | DGS |
| Cartaxo et al. 2010 | Caatinga | *Myracrodruon urundeuva* Allemão | Anacardiaceae | T | N | N | Inflammation of the ovaries | DGS |
| Cartaxo et al. 2010 | Caatinga | *Myracrodruon urundeuva* Allemão | Anacardiaceae | T | N | N | Inflammation of the ovaries | DGS |
| Cartaxo et al. 2010 | Caatinga | *Myracrodruon urundeuva* Allemão | Anacardiaceae | T | N | N | Influenza | DRS |
| Cartaxo et al. 2010 | Caatinga | *Myracrodruon urundeuva* Allemão | Anacardiaceae | T | N | N | Influenza | DRS |
| Cartaxo et al. 2010 | Caatinga | *Myracrodruon urundeuva* Allemão | Anacardiaceae | T | N | N | Intestinal problems | DDS |
| Cartaxo et al. 2010 | Caatinga | *Myracrodruon urundeuva* Allemão | Anacardiaceae | T | N | N | Intestinal problems | DDS |
| Cartaxo et al. 2010 | Caatinga | *Myracrodruon urundeuva* Allemão | Anacardiaceae | T | N | N | Kidney problems | DGS |
| Cartaxo et al. 2010 | Caatinga | *Myracrodruon urundeuva* Allemão | Anacardiaceae | T | N | N | Kidney problems | DGS |
| Cartaxo et al. 2010 | Caatinga | *Myracrodruon urundeuva* Allemão | Anacardiaceae | T | N | N | Liver problems | DDS |
| Cartaxo et al. 2010 | Caatinga | *Myracrodruon urundeuva* Allemão | Anacardiaceae | T | N | N | Liver problems | DDS |
| Cartaxo et al. 2010 | Caatinga | *Myracrodruon urundeuva* Allemão | Anacardiaceae | T | N | N | Toothache | DDS |
| Cartaxo et al. 2010 | Caatinga | *Myracrodruon urundeuva* Allemão | Anacardiaceae | T | N | N | Toothache | DDS |
| Cartaxo et al. 2010 | Caatinga | *Myracrodruon urundeuva* Allemão | Anacardiaceae | T | N | N | Wound | IPEC |
| Cartaxo et al. 2010 | Caatinga | *Myracrodruon urundeuva* Allemão | Anacardiaceae | T | N | N | Wound | IPEC |
| Cartaxo et al. 2010 | Caatinga | *Myristica fragrans* Houtt. | Myristicaceae | T | E | E | Calming | MBD |
| Cartaxo et al. 2010 | Caatinga | *Myristica fragrans* Houtt. | Myristicaceae | T | E | E | Headache | SSNEC |
| Cartaxo et al. 2010 | Caatinga | *Myristica fragrans* Houtt. | Myristicaceae | T | E | E | Respiratory problems | DRS |
| Cartaxo et al. 2010 | Caatinga | *Myristica fragrans* Houtt. | Myristicaceae | T | E | E | Stroke | DCS |
| Cartaxo et al. 2010 | Caatinga | *Myroxylon peruiferum* L.f. | Fabaceae | T | N | E | Antiseptic | CIPD |
| Cartaxo et al. 2010 | Caatinga | *Nasturtium officinale* W. T. Aiton | Brassicaceae | H | E | E | Throat problems | DRS |
| Cartaxo et al. 2010 | Caatinga | *Ocimum campechianum* Mill. | Lamiaceae | H | N | N | Antiseptic | CIPD |
| Cartaxo et al. 2010 | Caatinga | *Ocimum campechianum* Mill. | Lamiaceae | H | N | N | Antiseptic | CIPD |
| Cartaxo et al. 2010 | Caatinga | *Ocimum campechianum* Mill. | Lamiaceae | H | N | N | Influenza | DRS |
| Cartaxo et al. 2010 | Caatinga | *Ocimum campechianum* Mill. | Lamiaceae | H | N | N | Influenza | DRS |
| Cartaxo et al. 2010 | Caatinga | *Ocimum campechianum* Mill. | Lamiaceae | H | N | N | Sinusitis | DRS |
| Cartaxo et al. 2010 | Caatinga | *Ocimum campechianum* Mill. | Lamiaceae | H | N | N | Sinusitis | DRS |
| Cartaxo et al. 2010 | Caatinga | *Operculina macrocarpa* (L.) Urb. | Convolvulaceae | H | N | N | Anticoagulant | DCS |
| Cartaxo et al. 2010 | Caatinga | *Operculina macrocarpa* (L.) Urb. | Convolvulaceae | H | N | N | Helminthiasis | CIPD |
| Cartaxo et al. 2010 | Caatinga | *Operculina macrocarpa* (L.) Urb. | Convolvulaceae | H | N | N | Inflammation | SSNEC |
| Cartaxo et al. 2010 | Caatinga | *Passiflora cincinnata* Mast. | Passifloraceae | S | N | N | Calming | MBD |
| Cartaxo et al. 2010 | Caatinga | *Passiflora edulis* Sims | Passifloraceae | S | N | N | Calming | MBD |
| Cartaxo et al. 2010 | Caatinga | *Passiflora edulis* Sims | Passifloraceae | S | N | N | Hypertension | DCS |
| Cartaxo et al. 2010 | Caatinga | *Persea americana* Mill. | Lauraceae | T | E | E | Kidney pain | DGS |
| Cartaxo et al. 2010 | Caatinga | *Phoradendron mucronatum* (DC.) Krug & Urb. | Santalaceae | S | N | N | Rheumatism | DMS |
| Cartaxo et al. 2010 | Caatinga | *Phyllanthus amarus* Schumach. | Phyllanthaceae | H | N | N | Kidney problems | DGS |
| Cartaxo et al. 2010 | Caatinga | *Pimpinella anisum* L. | Apiaceae | H | E | E | Calming | MBD |
| Cartaxo et al. 2010 | Caatinga | *Pimpinella anisum* L. | Apiaceae | H | E | E | Calming | MBD |
| Cartaxo et al. 2010 | Caatinga | *Pimpinella anisum* L. | Apiaceae | H | E | E | Constipation | DDS |
| Cartaxo et al. 2010 | Caatinga | *Pimpinella anisum* L. | Apiaceae | H | E | E | Constipation | DDS |
| Cartaxo et al. 2010 | Caatinga | *Pimpinella anisum* L. | Apiaceae | H | E | E | Infant colic | SSNEC |
| Cartaxo et al. 2010 | Caatinga | *Pimpinella anisum* L. | Apiaceae | H | E | E | Infant colic | SSNEC |
| Cartaxo et al. 2010 | Caatinga | *Pimpinella anisum* L. | Apiaceae | H | E | E | Poor digestion | DDS |
| Cartaxo et al. 2010 | Caatinga | *Pimpinella anisum* L. | Apiaceae | H | E | E | Poor digestion | DDS |
| Cartaxo et al. 2010 | Caatinga | *Pimpinella anisum* L. | Apiaceae | H | E | E | Stomach problems | DDS |
| Cartaxo et al. 2010 | Caatinga | *Pimpinella anisum* L. | Apiaceae | H | E | E | Stomach problems | DDS |
| Cartaxo et al. 2010 | Caatinga | *Piper aduncum* L. | Piperaceae | S | N | E | Headache | SSNEC |
| Cartaxo et al. 2010 | Caatinga | *Piper aduncum* L. | Piperaceae | S | N | E | Menstrual cramps | DGS |
| Cartaxo et al. 2010 | Caatinga | *Plectranthus amboinicus* (Lour.) Spreng. | Lamiaceae | H | E | E | Anticoagulant | DCS |
| Cartaxo et al. 2010 | Caatinga | *Plectranthus amboinicus* (Lour.) Spreng. | Lamiaceae | H | E | E | Bronchitis | DRS |
| Cartaxo et al. 2010 | Caatinga | *Plectranthus amboinicus* (Lour.) Spreng. | Lamiaceae | H | E | E | Cough | SSNEC |
| Cartaxo et al. 2010 | Caatinga | *Plectranthus amboinicus* (Lour.) Spreng. | Lamiaceae | H | E | E | Expectorant | SSNEC |
| Cartaxo et al. 2010 | Caatinga | *Plectranthus amboinicus* (Lour.) Spreng. | Lamiaceae | H | E | E | Headache | SSNEC |
| Cartaxo et al. 2010 | Caatinga | *Plectranthus amboinicus* (Lour.) Spreng. | Lamiaceae | H | E | E | Inflammation | SSNEC |
| Cartaxo et al. 2010 | Caatinga | *Plectranthus amboinicus* (Lour.) Spreng. | Lamiaceae | H | E | E | Inflammation of internal organs | SSNEC |
| Cartaxo et al. 2010 | Caatinga | *Plectranthus amboinicus* (Lour.) Spreng. | Lamiaceae | H | E | E | Inflammation of the uterus | DGS |
| Cartaxo et al. 2010 | Caatinga | *Plectranthus amboinicus* (Lour.) Spreng. | Lamiaceae | H | E | E | Influenza | DRS |
| Cartaxo et al. 2010 | Caatinga | *Plectranthus amboinicus* (Lour.) Spreng. | Lamiaceae | H | E | E | Stomach problems | DDS |
| Cartaxo et al. 2010 | Caatinga | *Plectranthus amboinicus* (Lour.) Spreng. | Lamiaceae | H | E | E | Throat problems | DRS |
| Cartaxo et al. 2010 | Caatinga | *Poincianella pyramidalis* (Tul.) L.P.Queiroz | Fabaceae | T | N | N | Expectorant | SSNEC |
| Cartaxo et al. 2010 | Caatinga | *Poincianella pyramidalis* (Tul.) L.P.Queiroz | Fabaceae | T | N | N | Expectorant | SSNEC |
| Cartaxo et al. 2010 | Caatinga | *Poincianella pyramidalis* (Tul.) L.P.Queiroz | Fabaceae | T | N | N | Hemorrhage | SSNEC |
| Cartaxo et al. 2010 | Caatinga | *Poincianella pyramidalis* (Tul.) L.P.Queiroz | Fabaceae | T | N | N | Hemorrhage | SSNEC |
| Cartaxo et al. 2010 | Caatinga | *Poincianella pyramidalis* (Tul.) L.P.Queiroz | Fabaceae | T | N | N | Influenza | DRS |
| Cartaxo et al. 2010 | Caatinga | *Poincianella pyramidalis* (Tul.) L.P.Queiroz | Fabaceae | T | N | N | Influenza | DRS |
| Cartaxo et al. 2010 | Caatinga | *Poincianella pyramidalis* (Tul.) L.P.Queiroz | Fabaceae | T | N | N | Poor digestion | DDS |
| Cartaxo et al. 2010 | Caatinga | *Poincianella pyramidalis* (Tul.) L.P.Queiroz | Fabaceae | T | N | N | Poor digestion | DDS |
| Cartaxo et al. 2010 | Caatinga | *Poincianella pyramidalis* (Tul.) L.P.Queiroz | Fabaceae | T | N | N | Stomach pain/Diarrhea | SSNEC |
| Cartaxo et al. 2010 | Caatinga | *Poincianella pyramidalis* (Tul.) L.P.Queiroz | Fabaceae | T | N | N | Stomach pain/Diarrhea | SSNEC |
| Cartaxo et al. 2010 | Caatinga | *Poincianella pyramidalis* (Tul.) L.P.Queiroz | Fabaceae | T | N | N | Stomach pain/Diarrhea | SSNEC |
| Cartaxo et al. 2010 | Caatinga | *Poincianella pyramidalis* (Tul.) L.P.Queiroz | Fabaceae | T | N | N | Stomach pain/Diarrhea | SSNEC |
| Cartaxo et al. 2010 | Caatinga | *Poincianella pyramidalis* (Tul.) L.P.Queiroz | Fabaceae | T | N | N | Stomach problems | DDS |
| Cartaxo et al. 2010 | Caatinga | *Poincianella pyramidalis* (Tul.) L.P.Queiroz | Fabaceae | T | N | N | Stomach problems | DDS |
| Cartaxo et al. 2010 | Caatinga | *Poincianella pyramidalis* (Tul.) L.P.Queiroz | Fabaceae | T | N | N | Wound | IPEC |
| Cartaxo et al. 2010 | Caatinga | *Poincianella pyramidalis* (Tul.) L.P.Queiroz | Fabaceae | T | N | N | Wound | IPEC |
| Cartaxo et al. 2010 | Caatinga | *Psidium guajava* L. | Myrtaceae | S | E | E | Dysentry | CIPD |
| Cartaxo et al. 2010 | Caatinga | *Psidium guajava* L. | Myrtaceae | S | E | E | Intestinal problems | DDS |
| Cartaxo et al. 2010 | Caatinga | *Psidium guajava* L. | Myrtaceae | S | E | E | Stomach pain/Diarrhea | SSNEC |
| Cartaxo et al. 2010 | Caatinga | *Psidium guajava* L. | Myrtaceae | S | E | E | Stomach pain/Diarrhea | SSNEC |
| Cartaxo et al. 2010 | Caatinga | *Punica granatum* L. | Lythraceae | T | E | E | Gastritis | DDS |
| Cartaxo et al. 2010 | Caatinga | *Punica granatum* L. | Lythraceae | T | E | E | Gastritis | DDS |
| Cartaxo et al. 2010 | Caatinga | *Punica granatum* L. | Lythraceae | T | E | E | Gastritis | DDS |
| Cartaxo et al. 2010 | Caatinga | *Punica granatum* L. | Lythraceae | T | E | E | Inflammation of the throat | DRS |
| Cartaxo et al. 2010 | Caatinga | *Punica granatum* L. | Lythraceae | T | E | E | Inflammation of the throat | DRS |
| Cartaxo et al. 2010 | Caatinga | *Punica granatum* L. | Lythraceae | T | E | E | Inflammation of the throat | DRS |
| Cartaxo et al. 2010 | Caatinga | *Punica granatum* L. | Lythraceae | T | E | E | Influenza | DRS |
| Cartaxo et al. 2010 | Caatinga | *Punica granatum* L. | Lythraceae | T | E | E | Influenza | DRS |
| Cartaxo et al. 2010 | Caatinga | *Punica granatum* L. | Lythraceae | T | E | E | Influenza | DRS |
| Cartaxo et al. 2010 | Caatinga | *Punica granatum* L. | Lythraceae | T | E | E | Throat problems | DRS |
| Cartaxo et al. 2010 | Caatinga | *Punica granatum* L. | Lythraceae | T | E | E | Throat problems | DRS |
| Cartaxo et al. 2010 | Caatinga | *Punica granatum* L. | Lythraceae | T | E | E | Throat problems | DRS |
| Cartaxo et al. 2010 | Caatinga | *Rosmarinus officinalis* L. | Lamiaceae | S | E | E | Headache | SSNEC |
| Cartaxo et al. 2010 | Caatinga | *Rosmarinus officinalis* L. | Lamiaceae | S | E | E | Headache | SSNEC |
| Cartaxo et al. 2010 | Caatinga | *Rosmarinus officinalis* L. | Lamiaceae | S | E | E | Pain | SSNEC |
| Cartaxo et al. 2010 | Caatinga | *Rosmarinus officinalis* L. | Lamiaceae | S | E | E | Pain | SSNEC |
| Cartaxo et al. 2010 | Caatinga | *Rosmarinus officinalis* L. | Lamiaceae | S | E | E | Poor digestion | DDS |
| Cartaxo et al. 2010 | Caatinga | *Rosmarinus officinalis* L. | Lamiaceae | S | E | E | Poor digestion | DDS |
| Cartaxo et al. 2010 | Caatinga | *Rosmarinus officinalis* L. | Lamiaceae | S | E | E | Sinusitis | DRS |
| Cartaxo et al. 2010 | Caatinga | *Rosmarinus officinalis* L. | Lamiaceae | S | E | E | Sinusitis | DRS |
| Cartaxo et al. 2010 | Caatinga | *Ruta graveolens* L. | Rutaceae | H | E | E | Amenorrhea | DGS |
| Cartaxo et al. 2010 | Caatinga | *Ruta graveolens* L. | Rutaceae | H | E | E | Antiseptic | CIPD |
| Cartaxo et al. 2010 | Caatinga | *Ruta graveolens* L. | Rutaceae | H | E | E | Colic | SSNEC |
| Cartaxo et al. 2010 | Caatinga | *Ruta graveolens* L. | Rutaceae | H | E | E | Earache | DEMP |
| Cartaxo et al. 2010 | Caatinga | *Ruta graveolens* L. | Rutaceae | H | E | E | Fever | SSNEC |
| Cartaxo et al. 2010 | Caatinga | *Ruta graveolens* L. | Rutaceae | H | E | E | Headache | SSNEC |
| Cartaxo et al. 2010 | Caatinga | *Ruta graveolens* L. | Rutaceae | H | E | E | Headache | SSNEC |
| Cartaxo et al. 2010 | Caatinga | *Ruta graveolens* L. | Rutaceae | H | E | E | Hematoma | SSNEC |
| Cartaxo et al. 2010 | Caatinga | *Ruta graveolens* L. | Rutaceae | H | E | E | Intestinal infection | CIPD |
| Cartaxo et al. 2010 | Caatinga | *Ruta graveolens* L. | Rutaceae | H | E | E | Intestinal problems | DDS |
| Cartaxo et al. 2010 | Caatinga | *Ruta graveolens* L. | Rutaceae | H | E | E | Kidney problems | DGS |
| Cartaxo et al. 2010 | Caatinga | *Ruta graveolens* L. | Rutaceae | H | E | E | Menstrual cramps | DGS |
| Cartaxo et al. 2010 | Caatinga | *Ruta graveolens* L. | Rutaceae | H | E | E | Poor digestion | DDS |
| Cartaxo et al. 2010 | Caatinga | *Ruta graveolens* L. | Rutaceae | H | E | E | Tetanus | CIPD |
| Cartaxo et al. 2010 | Caatinga | *Ruta graveolens* L. | Rutaceae | H | E | E | Thrombosis | DCS |
| Cartaxo et al. 2010 | Caatinga | *Ruta graveolens* L. | Rutaceae | H | E | E | Wound | IPEC |
| Cartaxo et al. 2010 | Caatinga | *Saccharum officinarum* L. | Poaceae | H | E | E | Hypertension | DCS |
| Cartaxo et al. 2010 | Caatinga | *Sambucus australis* Cham. & Schltdl. | Adoxaceae | T | N | E | Wound | IPEC |
| Cartaxo et al. 2010 | Caatinga | *Scoparia dulcis* L. | Plantaginaceae | H | N | N | Cough | SSNEC |
| Cartaxo et al. 2010 | Caatinga | *Scoparia dulcis* L. | Plantaginaceae | H | N | N | Diuretic | SSNEC |
| Cartaxo et al. 2010 | Caatinga | *Scoparia dulcis* L. | Plantaginaceae | H | N | N | Influenza | DRS |
| Cartaxo et al. 2010 | Caatinga | *Scoparia dulcis* L. | Plantaginaceae | H | N | N | Kidney problems | DGS |
| Cartaxo et al. 2010 | Caatinga | *Scoparia dulcis* L. | Plantaginaceae | H | N | N | Teething in children | DDS |
| Cartaxo et al. 2010 | Caatinga | *Scoparia dulcis* L. | Plantaginaceae | H | N | N | Urinary problems | DGS |
| Cartaxo et al. 2010 | Caatinga | *Senegalia tenuifolia* (L.) Britton & Rose | Fabaceae | S | N | N | Influenza | DRS |
| Cartaxo et al. 2010 | Caatinga | *Senegalia tenuifolia* (L.) Britton & Rose | Fabaceae | S | N | N | Influenza | DRS |
| Cartaxo et al. 2010 | Caatinga | *Senegalia tenuifolia* (L.) Britton & Rose | Fabaceae | S | N | N | Pain in spinal column | DMS |
| Cartaxo et al. 2010 | Caatinga | *Senegalia tenuifolia* (L.) Britton & Rose | Fabaceae | S | N | N | Pain in spinal column | DMS |
| Cartaxo et al. 2010 | Caatinga | *Senegalia tenuifolia* (L.) Britton & Rose | Fabaceae | S | N | N | Wound | IPEC |
| Cartaxo et al. 2010 | Caatinga | *Senegalia tenuifolia* (L.) Britton & Rose | Fabaceae | S | N | N | Wound | IPEC |
| Cartaxo et al. 2010 | Caatinga | *Senna alexandrina* Mill. | Fabaceae | S | N | N | Amenorrhea | DGS |
| Cartaxo et al. 2010 | Caatinga | *Senna alexandrina* Mill. | Fabaceae | S | N | N | Influenza | DRS |
| Cartaxo et al. 2010 | Caatinga | *Senna spectabilis* (DC.) H.S.Irwin & Barneby | Fabaceae | T | N | N | Wound | IPEC |
| Cartaxo et al. 2010 | Caatinga | *Sesamum indicum* L. | Pedaliaceae | H | E | E | Allergy | IPEC |
| Cartaxo et al. 2010 | Caatinga | *Sesamum indicum* L. | Pedaliaceae | H | E | E | Eye inflammation | DEA |
| Cartaxo et al. 2010 | Caatinga | *Sesamum indicum* L. | Pedaliaceae | H | E | E | Fever | SSNEC |
| Cartaxo et al. 2010 | Caatinga | *Sesamum indicum* L. | Pedaliaceae | H | E | E | Headache | SSNEC |
| Cartaxo et al. 2010 | Caatinga | *Sesamum indicum* L. | Pedaliaceae | H | E | E | Stroke | DCS |
| Cartaxo et al. 2010 | Caatinga | *Solanum ambrosiacum* Vell. | Solanaceae | H | N | N | Kidney problems | DGS |
| Cartaxo et al. 2010 | Caatinga | *Spondias dulcis* Parkinson | Anacardiaceae | T | E | E | Antiseptic | CIPD |
| Cartaxo et al. 2010 | Caatinga | *Spondias dulcis* Parkinson | Anacardiaceae | T | E | E | Antiseptic | CIPD |
| Cartaxo et al. 2010 | Caatinga | *Spondias dulcis* Parkinson | Anacardiaceae | T | E | E | Antiseptic | CIPD |
| Cartaxo et al. 2010 | Caatinga | *Spondias dulcis* Parkinson | Anacardiaceae | T | E | E | Bronchitis | DRS |
| Cartaxo et al. 2010 | Caatinga | *Spondias dulcis* Parkinson | Anacardiaceae | T | E | E | Bronchitis | DRS |
| Cartaxo et al. 2010 | Caatinga | *Spondias dulcis* Parkinson | Anacardiaceae | T | E | E | Bronchitis | DRS |
| Cartaxo et al. 2010 | Caatinga | *Spondias dulcis* Parkinson | Anacardiaceae | T | E | E | Influenza | DRS |
| Cartaxo et al. 2010 | Caatinga | *Spondias dulcis* Parkinson | Anacardiaceae | T | E | E | Influenza | DRS |
| Cartaxo et al. 2010 | Caatinga | *Spondias dulcis* Parkinson | Anacardiaceae | T | E | E | Influenza | DRS |
| Cartaxo et al. 2010 | Caatinga | *Spondias dulcis* Parkinson | Anacardiaceae | T | E | E | Stomach pain/Diarrhea | SSNEC |
| Cartaxo et al. 2010 | Caatinga | *Spondias dulcis* Parkinson | Anacardiaceae | T | E | E | Stomach pain/Diarrhea | SSNEC |
| Cartaxo et al. 2010 | Caatinga | *Spondias dulcis* Parkinson | Anacardiaceae | T | E | E | Stomach pain/Diarrhea | SSNEC |
| Cartaxo et al. 2010 | Caatinga | *Spondias dulcis* Parkinson | Anacardiaceae | T | E | E | Stomach pain/Diarrhea | SSNEC |
| Cartaxo et al. 2010 | Caatinga | *Spondias dulcis* Parkinson | Anacardiaceae | T | E | E | Stomach pain/Diarrhea | SSNEC |
| Cartaxo et al. 2010 | Caatinga | *Spondias dulcis* Parkinson | Anacardiaceae | T | E | E | Stomach pain/Diarrhea | SSNEC |
| Cartaxo et al. 2010 | Caatinga | *Spondias dulcis* Parkinson | Anacardiaceae | T | E | E | Wound | IPEC |
| Cartaxo et al. 2010 | Caatinga | *Spondias dulcis* Parkinson | Anacardiaceae | T | E | E | Wound | IPEC |
| Cartaxo et al. 2010 | Caatinga | *Spondias dulcis* Parkinson | Anacardiaceae | T | E | E | Wound | IPEC |
| Cartaxo et al. 2010 | Caatinga | *Spondias mombin* L. | Anacardiaceae | T | N | N | Anemia | DBBO |
| Cartaxo et al. 2010 | Caatinga | *Spondias purpurea* L. | Anacardiaceae | T | E | E | Stomach pain/Diarrhea | SSNEC |
| Cartaxo et al. 2010 | Caatinga | *Syzygium aromaticum* (L.) Merr. & L. M. Perry | Myrtaceae | T | E | E | Bad breath | SSNEC |
| Cartaxo et al. 2010 | Caatinga | *Tamarindus indica* L. | Fabaceae | T | E | E | Cough | SSNEC |
| Cartaxo et al. 2010 | Caatinga | *Tamarindus indica* L. | Fabaceae | T | E | E | Influenza | DRS |
| Cartaxo et al. 2010 | Caatinga | *Tarenaya spinosa* (Jacq.) Raf. | Capparaceae | H | N | N | Influenza | DRS |
| Cartaxo et al. 2010 | Caatinga | *Tarenaya spinosa* (Jacq.) Raf. | Capparaceae | H | N | N | Influenza | DRS |
| Cartaxo et al. 2010 | Caatinga | *Tarenaya spinosa* (Jacq.) Raf. | Capparaceae | H | N | N | Influenza | DRS |
| Cartaxo et al. 2010 | Caatinga | *Tarenaya spinosa* (Jacq.) Raf. | Capparaceae | H | N | N | Tuberculosis | CIPD |
| Cartaxo et al. 2010 | Caatinga | *Tarenaya spinosa* (Jacq.) Raf. | Capparaceae | H | N | N | Tuberculosis | CIPD |
| Cartaxo et al. 2010 | Caatinga | *Tarenaya spinosa* (Jacq.) Raf. | Capparaceae | H | N | N | Tuberculosis | CIPD |
| Cartaxo et al. 2010 | Caatinga | *Tarenaya spinosa* (Jacq.) Raf. | Capparaceae | H | N | N | Wound | IPEC |
| Cartaxo et al. 2010 | Caatinga | *Tarenaya spinosa* (Jacq.) Raf. | Capparaceae | H | N | N | Wound | IPEC |
| Cartaxo et al. 2010 | Caatinga | *Tarenaya spinosa* (Jacq.) Raf. | Capparaceae | H | N | N | Wound | IPEC |
| Cartaxo et al. 2010 | Caatinga | *Terminalia catappa* L. | Combretaceae | T | E | E | Kidney problems | DGS |
| Cartaxo et al. 2010 | Caatinga | *Triplaris gardneriana* Wedd. | Polygonaceae | T | N | N | Inflammation of internal organs | SSNEC |
| Cartaxo et al. 2010 | Caatinga | *Turnera ulmifolia* L. | Passifloraceae | H | E | E | Inflammation of the female organs | DGS |
| Cartaxo et al. 2010 | Caatinga | *Turnera ulmifolia* L. | Passifloraceae | H | E | E | Inflammation of the uterus | DGS |
| Cartaxo et al. 2010 | Caatinga | *Turnera ulmifolia* L. | Passifloraceae | H | E | E | Influenza | DRS |
| Cartaxo et al. 2010 | Caatinga | *Ximenia americana* L. | Ximeniaceae | T | N | N | Antiseptic | CIPD |
| Cartaxo et al. 2010 | Caatinga | *Ximenia americana* L. | Ximeniaceae | T | N | N | Antiseptic | CIPD |
| Cartaxo et al. 2010 | Caatinga | *Ximenia americana* L. | Ximeniaceae | T | N | N | Inflammation | SSNEC |
| Cartaxo et al. 2010 | Caatinga | *Ximenia americana* L. | Ximeniaceae | T | N | N | Inflammation | SSNEC |
| Cartaxo et al. 2010 | Caatinga | *Ximenia americana* L. | Ximeniaceae | T | N | N | Inflammation of internal organs | SSNEC |
| Cartaxo et al. 2010 | Caatinga | *Ximenia americana* L. | Ximeniaceae | T | N | N | Inflammation of internal organs | SSNEC |
| Cartaxo et al. 2010 | Caatinga | *Ximenia americana* L. | Ximeniaceae | T | N | N | Menstrual cramps | DGS |
| Cartaxo et al. 2010 | Caatinga | *Ximenia americana* L. | Ximeniaceae | T | N | N | Menstrual cramps | DGS |
| Cartaxo et al. 2010 | Caatinga | *Ximenia americana* L. | Ximeniaceae | T | N | N | Toothache | DDS |
| Cartaxo et al. 2010 | Caatinga | *Ximenia americana* L. | Ximeniaceae | T | N | N | Toothache | DDS |
| Cartaxo et al. 2010 | Caatinga | *Ximenia americana* L. | Ximeniaceae | T | N | N | Wound | IPEC |
| Cartaxo et al. 2010 | Caatinga | *Ximenia americana* L. | Ximeniaceae | T | N | N | Wound | IPEC |
| Cartaxo et al. 2010 | Caatinga | *Zea mays* L. | Poaceae | H | E | E | Hypertension | DCS |
| Cartaxo et al. 2010 | Caatinga | *Ziziphus joazeiro* Mart. | Rhamnaceae | T | N | N | Antiseptic | CIPD |
| Cartaxo et al. 2010 | Caatinga | *Ziziphus joazeiro* Mart. | Rhamnaceae | T | N | N | Antiseptic | CIPD |
| Cartaxo et al. 2010 | Caatinga | *Ziziphus joazeiro* Mart. | Rhamnaceae | T | N | N | Antiseptic | CIPD |
| Cartaxo et al. 2010 | Caatinga | *Ziziphus joazeiro* Mart. | Rhamnaceae | T | N | N | Dandruff | DSST |
| Cartaxo et al. 2010 | Caatinga | *Ziziphus joazeiro* Mart. | Rhamnaceae | T | N | N | Dandruff | DSST |
| Cartaxo et al. 2010 | Caatinga | *Ziziphus joazeiro* Mart. | Rhamnaceae | T | N | N | Dandruff | DSST |
| Cartaxo et al. 2010 | Caatinga | *Ziziphus joazeiro* Mart. | Rhamnaceae | T | N | N | Dental cleaning | DDS |
| Cartaxo et al. 2010 | Caatinga | *Ziziphus joazeiro* Mart. | Rhamnaceae | T | N | N | Dental cleaning | DDS |
| Cartaxo et al. 2010 | Caatinga | *Ziziphus joazeiro* Mart. | Rhamnaceae | T | N | N | Dental cleaning | DDS |
| Cartaxo et al. 2010 | Caatinga | *Ziziphus joazeiro* Mart. | Rhamnaceae | T | N | N | Fever | SSNEC |
| Cartaxo et al. 2010 | Caatinga | *Ziziphus joazeiro* Mart. | Rhamnaceae | T | N | N | Fever | SSNEC |
| Cartaxo et al. 2010 | Caatinga | *Ziziphus joazeiro* Mart. | Rhamnaceae | T | N | N | Fever | SSNEC |
| Cartaxo et al. 2010 | Caatinga | *Ziziphus joazeiro* Mart. | Rhamnaceae | T | N | N | Hair strengthener | DSST |
| Cartaxo et al. 2010 | Caatinga | *Ziziphus joazeiro* Mart. | Rhamnaceae | T | N | N | Hair strengthener | DSST |
| Cartaxo et al. 2010 | Caatinga | *Ziziphus joazeiro* Mart. | Rhamnaceae | T | N | N | Hair strengthener | DSST |
| Cartaxo et al. 2010 | Caatinga | *Ziziphus joazeiro* Mart. | Rhamnaceae | T | N | N | Heartburn | DGS |
| Cartaxo et al. 2010 | Caatinga | *Ziziphus joazeiro* Mart. | Rhamnaceae | T | N | N | Heartburn | DGS |
| Cartaxo et al. 2010 | Caatinga | *Ziziphus joazeiro* Mart. | Rhamnaceae | T | N | N | Heartburn | DGS |
| Cartaxo et al. 2010 | Caatinga | *Ziziphus joazeiro* Mart. | Rhamnaceae | T | N | N | Influenza | DRS |
| Cartaxo et al. 2010 | Caatinga | *Ziziphus joazeiro* Mart. | Rhamnaceae | T | N | N | Influenza | DRS |
| Cartaxo et al. 2010 | Caatinga | *Ziziphus joazeiro* Mart. | Rhamnaceae | T | N | N | Influenza | DRS |
| Cartaxo et al. 2010 | Caatinga | *Ziziphus joazeiro* Mart. | Rhamnaceae | T | N | N | Poor digestion | DDS |
| Cartaxo et al. 2010 | Caatinga | *Ziziphus joazeiro* Mart. | Rhamnaceae | T | N | N | Poor digestion | DDS |
| Cartaxo et al. 2010 | Caatinga | *Ziziphus joazeiro* Mart. | Rhamnaceae | T | N | N | Poor digestion | DDS |
| Cartaxo et al. 2010 | Caatinga | *Ziziphus joazeiro* Mart. | Rhamnaceae | T | N | N | Rheumatism | DMS |
| Cartaxo et al. 2010 | Caatinga | *Ziziphus joazeiro* Mart. | Rhamnaceae | T | N | N | Rheumatism | DMS |
| Cartaxo et al. 2010 | Caatinga | *Ziziphus joazeiro* Mart. | Rhamnaceae | T | N | N | Rheumatism | DMS |
| Cartaxo et al. 2010 | Caatinga | *Ziziphus joazeiro* Mart. | Rhamnaceae | T | N | N | Stomach problems | DDS |
| Cartaxo et al. 2010 | Caatinga | *Ziziphus joazeiro* Mart. | Rhamnaceae | T | N | N | Stomach problems | DDS |
| Cartaxo et al. 2010 | Caatinga | *Ziziphus joazeiro* Mart. | Rhamnaceae | T | N | N | Stomach problems | DDS |
| Cartaxo et al. 2010 | Caatinga | *Ziziphus joazeiro* Mart. | Rhamnaceae | T | N | N | Wound | IPEC |
| Cartaxo et al. 2010 | Caatinga | *Ziziphus joazeiro* Mart. | Rhamnaceae | T | N | N | Wound | IPEC |
| Cartaxo et al. 2010 | Caatinga | *Ziziphus joazeiro* Mart. | Rhamnaceae | T | N | N | Wound | IPEC |
| Cruz-Silva et al. 2009 | Atlantic Forest | *Achyrocline satureioides* (Lam.) DC. | Asteraceae | S | N | N | Bladder pain | SSNEC |
| Cruz-Silva et al. 2009 | Atlantic Forest | *Achyrocline satureioides* (Lam.) DC. | Asteraceae | S | N | N | Calming | MBD |
| Cruz-Silva et al. 2009 | Atlantic Forest | *Achyrocline satureioides* (Lam.) DC. | Asteraceae | S | N | N | Colic | SSNEC |
| Cruz-Silva et al. 2009 | Atlantic Forest | *Achyrocline satureioides* (Lam.) DC. | Asteraceae | S | N | N | Improves digestion | DDS |
| Cruz-Silva et al. 2009 | Atlantic Forest | *Achyrocline satureioides* (Lam.) DC. | Asteraceae | S | N | N | Influenza | DRS |
| Cruz-Silva et al. 2009 | Atlantic Forest | *Achyrocline satureioides* (Lam.) DC. | Asteraceae | S | N | N | Insomnia | DNS |
| Cruz-Silva et al. 2009 | Atlantic Forest | *Achyrocline satureioides* (Lam.) DC. | Asteraceae | S | N | N | Liver problems | DDS |
| Cruz-Silva et al. 2009 | Atlantic Forest | *Achyrocline satureioides* (Lam.) DC. | Asteraceae | S | N | N | Stomach pain/Diarrhea | SSNEC |
| Cruz-Silva et al. 2009 | Atlantic Forest | *Achyrocline satureioides* (Lam.) DC. | Asteraceae | S | N | N | Stomach problems | DDS |
| Cruz-Silva et al. 2009 | Atlantic Forest | *Citrus aurantium* L. | Rutaceae | T | E | E | Calming | MBD |
| Cruz-Silva et al. 2009 | Atlantic Forest | *Citrus aurantium* L. | Rutaceae | T | E | E | Calming | MBD |
| Cruz-Silva et al. 2009 | Atlantic Forest | *Citrus aurantium* L. | Rutaceae | T | E | E | Cough | SSNEC |
| Cruz-Silva et al. 2009 | Atlantic Forest | *Citrus aurantium* L. | Rutaceae | T | E | E | Cough | SSNEC |
| Cruz-Silva et al. 2009 | Atlantic Forest | *Citrus aurantium* L. | Rutaceae | T | E | E | Fever | SSNEC |
| Cruz-Silva et al. 2009 | Atlantic Forest | *Citrus aurantium* L. | Rutaceae | T | E | E | Fever | SSNEC |
| Cruz-Silva et al. 2009 | Atlantic Forest | *Citrus aurantium* L. | Rutaceae | T | E | E | Headache | SSNEC |
| Cruz-Silva et al. 2009 | Atlantic Forest | *Citrus aurantium* L. | Rutaceae | T | E | E | Headache | SSNEC |
| Cruz-Silva et al. 2009 | Atlantic Forest | *Citrus aurantium* L. | Rutaceae | T | E | E | Influenza | DRS |
| Cruz-Silva et al. 2009 | Atlantic Forest | *Citrus aurantium* L. | Rutaceae | T | E | E | Influenza | DRS |
| Cruz-Silva et al. 2009 | Atlantic Forest | *Cymbopogon citratus* (DC.) Stapf | Poaceae | H | E | E | Calming | MBD |
| Cruz-Silva et al. 2009 | Atlantic Forest | *Cymbopogon citratus* (DC.) Stapf | Poaceae | H | E | E | Cough | SSNEC |
| Cruz-Silva et al. 2009 | Atlantic Forest | *Cymbopogon citratus* (DC.) Stapf | Poaceae | H | E | E | Fever | SSNEC |
| Cruz-Silva et al. 2009 | Atlantic Forest | *Cymbopogon citratus* (DC.) Stapf | Poaceae | H | E | E | Headache | SSNEC |
| Cruz-Silva et al. 2009 | Atlantic Forest | *Cymbopogon citratus* (DC.) Stapf | Poaceae | H | E | E | Hypertension | DCS |
| Cruz-Silva et al. 2009 | Atlantic Forest | *Cymbopogon citratus* (DC.) Stapf | Poaceae | H | E | E | Influenza | DRS |
| Cruz-Silva et al. 2009 | Atlantic Forest | *Cymbopogon citratus* (DC.) Stapf | Poaceae | H | E | E | Insomnia | DNS |
| Cruz-Silva et al. 2009 | Atlantic Forest | *Cymbopogon citratus* (DC.) Stapf | Poaceae | H | E | E | Stomach problems | DDS |
| Cruz-Silva et al. 2009 | Atlantic Forest | *Foeniculum vulgare* Mill. | Apiaceae | H | E | E | Bladder pain | SSNEC |
| Cruz-Silva et al. 2009 | Atlantic Forest | *Foeniculum vulgare* Mill. | Apiaceae | H | E | E | Bladder pain | SSNEC |
| Cruz-Silva et al. 2009 | Atlantic Forest | *Foeniculum vulgare* Mill. | Apiaceae | H | E | E | Bladder pain | SSNEC |
| Cruz-Silva et al. 2009 | Atlantic Forest | *Foeniculum vulgare* Mill. | Apiaceae | H | E | E | Bladder pain | SSNEC |
| Cruz-Silva et al. 2009 | Atlantic Forest | *Foeniculum vulgare* Mill. | Apiaceae | H | E | E | Breast milk production | PCP |
| Cruz-Silva et al. 2009 | Atlantic Forest | *Foeniculum vulgare* Mill. | Apiaceae | H | E | E | Breast milk production | PCP |
| Cruz-Silva et al. 2009 | Atlantic Forest | *Foeniculum vulgare* Mill. | Apiaceae | H | E | E | Breast milk production | PCP |
| Cruz-Silva et al. 2009 | Atlantic Forest | *Foeniculum vulgare* Mill. | Apiaceae | H | E | E | Breast milk production | PCP |
| Cruz-Silva et al. 2009 | Atlantic Forest | *Foeniculum vulgare* Mill. | Apiaceae | H | E | E | Calming | MBD |
| Cruz-Silva et al. 2009 | Atlantic Forest | *Foeniculum vulgare* Mill. | Apiaceae | H | E | E | Calming | MBD |
| Cruz-Silva et al. 2009 | Atlantic Forest | *Foeniculum vulgare* Mill. | Apiaceae | H | E | E | Calming | MBD |
| Cruz-Silva et al. 2009 | Atlantic Forest | *Foeniculum vulgare* Mill. | Apiaceae | H | E | E | Calming | MBD |
| Cruz-Silva et al. 2009 | Atlantic Forest | *Foeniculum vulgare* Mill. | Apiaceae | H | E | E | Colic | SSNEC |
| Cruz-Silva et al. 2009 | Atlantic Forest | *Foeniculum vulgare* Mill. | Apiaceae | H | E | E | Colic | SSNEC |
| Cruz-Silva et al. 2009 | Atlantic Forest | *Foeniculum vulgare* Mill. | Apiaceae | H | E | E | Colic | SSNEC |
| Cruz-Silva et al. 2009 | Atlantic Forest | *Foeniculum vulgare* Mill. | Apiaceae | H | E | E | Colic | SSNEC |
| Cruz-Silva et al. 2009 | Atlantic Forest | *Foeniculum vulgare* Mill. | Apiaceae | H | E | E | Flatulence | SSNEC |
| Cruz-Silva et al. 2009 | Atlantic Forest | *Foeniculum vulgare* Mill. | Apiaceae | H | E | E | Flatulence | SSNEC |
| Cruz-Silva et al. 2009 | Atlantic Forest | *Foeniculum vulgare* Mill. | Apiaceae | H | E | E | Flatulence | SSNEC |
| Cruz-Silva et al. 2009 | Atlantic Forest | *Foeniculum vulgare* Mill. | Apiaceae | H | E | E | Flatulence | SSNEC |
| Cruz-Silva et al. 2009 | Atlantic Forest | *Foeniculum vulgare* Mill. | Apiaceae | H | E | E | Headache | SSNEC |
| Cruz-Silva et al. 2009 | Atlantic Forest | *Foeniculum vulgare* Mill. | Apiaceae | H | E | E | Headache | SSNEC |
| Cruz-Silva et al. 2009 | Atlantic Forest | *Foeniculum vulgare* Mill. | Apiaceae | H | E | E | Headache | SSNEC |
| Cruz-Silva et al. 2009 | Atlantic Forest | *Foeniculum vulgare* Mill. | Apiaceae | H | E | E | Headache | SSNEC |
| Cruz-Silva et al. 2009 | Atlantic Forest | *Foeniculum vulgare* Mill. | Apiaceae | H | E | E | High blood pressure | DCS |
| Cruz-Silva et al. 2009 | Atlantic Forest | *Foeniculum vulgare* Mill. | Apiaceae | H | E | E | High blood pressure | DCS |
| Cruz-Silva et al. 2009 | Atlantic Forest | *Foeniculum vulgare* Mill. | Apiaceae | H | E | E | High blood pressure | DCS |
| Cruz-Silva et al. 2009 | Atlantic Forest | *Foeniculum vulgare* Mill. | Apiaceae | H | E | E | High blood pressure | DCS |
| Cruz-Silva et al. 2009 | Atlantic Forest | *Foeniculum vulgare* Mill. | Apiaceae | H | E | E | Improves digestion | DDS |
| Cruz-Silva et al. 2009 | Atlantic Forest | *Foeniculum vulgare* Mill. | Apiaceae | H | E | E | Improves digestion | DDS |
| Cruz-Silva et al. 2009 | Atlantic Forest | *Foeniculum vulgare* Mill. | Apiaceae | H | E | E | Improves digestion | DDS |
| Cruz-Silva et al. 2009 | Atlantic Forest | *Foeniculum vulgare* Mill. | Apiaceae | H | E | E | Improves digestion | DDS |
| Cruz-Silva et al. 2009 | Atlantic Forest | *Foeniculum vulgare* Mill. | Apiaceae | H | E | E | Influenza | DRS |
| Cruz-Silva et al. 2009 | Atlantic Forest | *Foeniculum vulgare* Mill. | Apiaceae | H | E | E | Influenza | DRS |
| Cruz-Silva et al. 2009 | Atlantic Forest | *Foeniculum vulgare* Mill. | Apiaceae | H | E | E | Influenza | DRS |
| Cruz-Silva et al. 2009 | Atlantic Forest | *Foeniculum vulgare* Mill. | Apiaceae | H | E | E | Influenza | DRS |
| Cruz-Silva et al. 2009 | Atlantic Forest | *Foeniculum vulgare* Mill. | Apiaceae | H | E | E | Intestinal problems | DDS |
| Cruz-Silva et al. 2009 | Atlantic Forest | *Foeniculum vulgare* Mill. | Apiaceae | H | E | E | Intestinal problems | DDS |
| Cruz-Silva et al. 2009 | Atlantic Forest | *Foeniculum vulgare* Mill. | Apiaceae | H | E | E | Intestinal problems | DDS |
| Cruz-Silva et al. 2009 | Atlantic Forest | *Foeniculum vulgare* Mill. | Apiaceae | H | E | E | Intestinal problems | DDS |
| Cruz-Silva et al. 2009 | Atlantic Forest | *Foeniculum vulgare* Mill. | Apiaceae | H | E | E | Stomach pain/Diarrhea | SSNEC |
| Cruz-Silva et al. 2009 | Atlantic Forest | *Foeniculum vulgare* Mill. | Apiaceae | H | E | E | Stomach pain/Diarrhea | SSNEC |
| Cruz-Silva et al. 2009 | Atlantic Forest | *Foeniculum vulgare* Mill. | Apiaceae | H | E | E | Stomach pain/Diarrhea | SSNEC |
| Cruz-Silva et al. 2009 | Atlantic Forest | *Foeniculum vulgare* Mill. | Apiaceae | H | E | E | Stomach pain/Diarrhea | SSNEC |
| Cruz-Silva et al. 2009 | Atlantic Forest | *Foeniculum vulgare* Mill. | Apiaceae | H | E | E | Stomach problems | DDS |
| Cruz-Silva et al. 2009 | Atlantic Forest | *Foeniculum vulgare* Mill. | Apiaceae | H | E | E | Stomach problems | DDS |
| Cruz-Silva et al. 2009 | Atlantic Forest | *Foeniculum vulgare* Mill. | Apiaceae | H | E | E | Stomach problems | DDS |
| Cruz-Silva et al. 2009 | Atlantic Forest | *Foeniculum vulgare* Mill. | Apiaceae | H | E | E | Stomach problems | DDS |
| Cruz-Silva et al. 2009 | Atlantic Forest | *Matricaria chamomilla* L. | Asteraceae | H | E | E | Bronchitis | DRS |
| Cruz-Silva et al. 2009 | Atlantic Forest | *Matricaria chamomilla* L. | Asteraceae | H | E | E | Calming | MBD |
| Cruz-Silva et al. 2009 | Atlantic Forest | *Matricaria chamomilla* L. | Asteraceae | H | E | E | Colic | SSNEC |
| Cruz-Silva et al. 2009 | Atlantic Forest | *Matricaria chamomilla* L. | Asteraceae | H | E | E | Fever | SSNEC |
| Cruz-Silva et al. 2009 | Atlantic Forest | *Matricaria chamomilla* L. | Asteraceae | H | E | E | High blood pressure | DCS |
| Cruz-Silva et al. 2009 | Atlantic Forest | *Matricaria chamomilla* L. | Asteraceae | H | E | E | Improves digestion | DDS |
| Cruz-Silva et al. 2009 | Atlantic Forest | *Matricaria chamomilla* L. | Asteraceae | H | E | E | Infection | CIPD |
| Cruz-Silva et al. 2009 | Atlantic Forest | *Matricaria chamomilla* L. | Asteraceae | H | E | E | Intestinal problems | DDS |
| Cruz-Silva et al. 2009 | Atlantic Forest | *Matricaria chamomilla* L. | Asteraceae | H | E | E | Laxative | DDS |
| Cruz-Silva et al. 2009 | Atlantic Forest | *Matricaria chamomilla* L. | Asteraceae | H | E | E | Stomach pain/Diarrhea | SSNEC |
| Cruz-Silva et al. 2009 | Atlantic Forest | *Matricaria chamomilla* L. | Asteraceae | H | E | E | Stomach problems | DDS |
| Cruz-Silva et al. 2009 | Atlantic Forest | *Mentha pulegium* L. | Lamiaceae | H | E | E | Allergy | IPEC |
| Cruz-Silva et al. 2009 | Atlantic Forest | *Mentha pulegium* L. | Lamiaceae | H | E | E | Allergy | IPEC |
| Cruz-Silva et al. 2009 | Atlantic Forest | *Mentha pulegium* L. | Lamiaceae | H | E | E | Calming | MBD |
| Cruz-Silva et al. 2009 | Atlantic Forest | *Mentha pulegium* L. | Lamiaceae | H | E | E | Calming | MBD |
| Cruz-Silva et al. 2009 | Atlantic Forest | *Mentha pulegium* L. | Lamiaceae | H | E | E | Colic | SSNEC |
| Cruz-Silva et al. 2009 | Atlantic Forest | *Mentha pulegium* L. | Lamiaceae | H | E | E | Colic | SSNEC |
| Cruz-Silva et al. 2009 | Atlantic Forest | *Mentha pulegium* L. | Lamiaceae | H | E | E | Cough | SSNEC |
| Cruz-Silva et al. 2009 | Atlantic Forest | *Mentha pulegium* L. | Lamiaceae | H | E | E | Cough | SSNEC |
| Cruz-Silva et al. 2009 | Atlantic Forest | *Mentha pulegium* L. | Lamiaceae | H | E | E | Fever | SSNEC |
| Cruz-Silva et al. 2009 | Atlantic Forest | *Mentha pulegium* L. | Lamiaceae | H | E | E | Fever | SSNEC |
| Cruz-Silva et al. 2009 | Atlantic Forest | *Mentha pulegium* L. | Lamiaceae | H | E | E | Flatulence | SSNEC |
| Cruz-Silva et al. 2009 | Atlantic Forest | *Mentha pulegium* L. | Lamiaceae | H | E | E | Flatulence | SSNEC |
| Cruz-Silva et al. 2009 | Atlantic Forest | *Mentha pulegium* L. | Lamiaceae | H | E | E | Infection | CIPD |
| Cruz-Silva et al. 2009 | Atlantic Forest | *Mentha pulegium* L. | Lamiaceae | H | E | E | Infection | CIPD |
| Cruz-Silva et al. 2009 | Atlantic Forest | *Mentha pulegium* L. | Lamiaceae | H | E | E | Influenza | DRS |
| Cruz-Silva et al. 2009 | Atlantic Forest | *Mentha pulegium* L. | Lamiaceae | H | E | E | Influenza | DRS |
| Cruz-Silva et al. 2009 | Atlantic Forest | *Mentha pulegium* L. | Lamiaceae | H | E | E | Insomnia | DNS |
| Cruz-Silva et al. 2009 | Atlantic Forest | *Mentha pulegium* L. | Lamiaceae | H | E | E | Insomnia | DNS |
| Cruz-Silva et al. 2009 | Atlantic Forest | *Mentha pulegium* L. | Lamiaceae | H | E | E | Stomach pain/Diarrhea | SSNEC |
| Cruz-Silva et al. 2009 | Atlantic Forest | *Mentha pulegium* L. | Lamiaceae | H | E | E | Stomach pain/Diarrhea | SSNEC |
| Cruz-Silva et al. 2009 | Atlantic Forest | *Mentha pulegium* L. | Lamiaceae | H | E | E | Stomach problems | DDS |
| Cruz-Silva et al. 2009 | Atlantic Forest | *Mentha pulegium* L. | Lamiaceae | H | E | E | Stomach problems | DDS |
| Cruz-Silva et al. 2009 | Atlantic Forest | *Origanum majorana* L. | Lamiaceae | H | E | E | Calming | MBD |
| Cruz-Silva et al. 2009 | Atlantic Forest | *Origanum majorana* L. | Lamiaceae | H | E | E | Calming | MBD |
| Cruz-Silva et al. 2009 | Atlantic Forest | *Origanum majorana* L. | Lamiaceae | H | E | E | Colic | SSNEC |
| Cruz-Silva et al. 2009 | Atlantic Forest | *Origanum majorana* L. | Lamiaceae | H | E | E | Colic | SSNEC |
| Cruz-Silva et al. 2009 | Atlantic Forest | *Origanum majorana* L. | Lamiaceae | H | E | E | High blood pressure | DCS |
| Cruz-Silva et al. 2009 | Atlantic Forest | *Origanum majorana* L. | Lamiaceae | H | E | E | High blood pressure | DCS |
| Cruz-Silva et al. 2009 | Atlantic Forest | *Origanum majorana* L. | Lamiaceae | H | E | E | Influenza | DRS |
| Cruz-Silva et al. 2009 | Atlantic Forest | *Origanum majorana* L. | Lamiaceae | H | E | E | Influenza | DRS |
| Cruz-Silva et al. 2009 | Atlantic Forest | *Origanum majorana* L. | Lamiaceae | H | E | E | Intestinal problems | DDS |
| Cruz-Silva et al. 2009 | Atlantic Forest | *Origanum majorana* L. | Lamiaceae | H | E | E | Intestinal problems | DDS |
| Cruz-Silva et al. 2009 | Atlantic Forest | *Origanum majorana* L. | Lamiaceae | H | E | E | Kidney problems | DGS |
| Cruz-Silva et al. 2009 | Atlantic Forest | *Origanum majorana* L. | Lamiaceae | H | E | E | Kidney problems | DGS |
| Cruz-Silva et al. 2009 | Atlantic Forest | *Origanum majorana* L. | Lamiaceae | H | E | E | Stomach pain/Diarrhea | SSNEC |
| Cruz-Silva et al. 2009 | Atlantic Forest | *Origanum majorana* L. | Lamiaceae | H | E | E | Stomach pain/Diarrhea | SSNEC |
| Cruz-Silva et al. 2009 | Atlantic Forest | *Plectranthus barbatus* Andr. | Lamiaceae | H | E | E | Calming | MBD |
| Cruz-Silva et al. 2009 | Atlantic Forest | *Plectranthus barbatus* Andr. | Lamiaceae | H | E | E | Headache | SSNEC |
| Cruz-Silva et al. 2009 | Atlantic Forest | *Plectranthus barbatus* Andr. | Lamiaceae | H | E | E | Liver problems | DDS |
| Cruz-Silva et al. 2009 | Atlantic Forest | *Plectranthus barbatus* Andr. | Lamiaceae | H | E | E | Stomach pain/Diarrhea | SSNEC |
| Cruz-Silva et al. 2009 | Atlantic Forest | *Plectranthus barbatus* Andr. | Lamiaceae | H | E | E | Stomach problems | DDS |
| Franco & Barros 2006 | Cerrado and Mata dos Cocais | *Allium ascalonicum* L. | Alliaceae | H | E | E | Influenza | DRS |
| Franco & Barros 2006 | Cerrado and Mata dos Cocais | *Allium sativum* L. | Alliaceae | H | E | E | Influenza | DRS |
| Franco & Barros 2006 | Cerrado and Mata dos Cocais | *Anacardium occidentale* L. | Anacardiaceae | T | N | N | Influenza | DRS |
| Franco & Barros 2006 | Cerrado and Mata dos Cocais | *Anacardium occidentale* L. | Anacardiaceae | T | N | N | Influenza | DRS |
| Franco & Barros 2006 | Cerrado and Mata dos Cocais | *Anacardium occidentale* L. | Anacardiaceae | T | N | N | Influenza | DRS |
| Franco & Barros 2006 | Cerrado and Mata dos Cocais | *Anacardium occidentale* L. | Anacardiaceae | T | N | N | Stomach pain/Diarrhea | SSNEC |
| Franco & Barros 2006 | Cerrado and Mata dos Cocais | *Anacardium occidentale* L. | Anacardiaceae | T | N | N | Stomach pain/Diarrhea | SSNEC |
| Franco & Barros 2006 | Cerrado and Mata dos Cocais | *Anacardium occidentale* L. | Anacardiaceae | T | N | N | Stomach pain/Diarrhea | SSNEC |
| Franco & Barros 2006 | Cerrado and Mata dos Cocais | *Anacardium occidentale* L. | Anacardiaceae | T | N | N | Stroke | DCS |
| Franco & Barros 2006 | Cerrado and Mata dos Cocais | *Anacardium occidentale* L. | Anacardiaceae | T | N | N | Stroke | DCS |
| Franco & Barros 2006 | Cerrado and Mata dos Cocais | *Anacardium occidentale* L. | Anacardiaceae | T | N | N | Stroke | DCS |
| Franco & Barros 2006 | Cerrado and Mata dos Cocais | *Anadenanthera colubrina* (Vell.) Brenan | Fabaceae | T | N | N | Back pain | DMS |
| Franco & Barros 2006 | Cerrado and Mata dos Cocais | *Anadenanthera colubrina* (Vell.) Brenan | Fabaceae | T | N | N | Inflammation | SSNEC |
| Franco & Barros 2006 | Cerrado and Mata dos Cocais | *Anadenanthera colubrina* (Vell.) Brenan | Fabaceae | T | N | N | Influenza | DRS |
| Franco & Barros 2006 | Cerrado and Mata dos Cocais | *Aspidosperma pyrifolium* Mart. | Apocynaceae | T | N | N | Fever | SSNEC |
| Franco & Barros 2006 | Cerrado and Mata dos Cocais | *Attalea speciosa* Mart. ex Spreng. | Arecaceae | T | N | N | Stomach pain/Diarrhea | SSNEC |
| Franco & Barros 2006 | Cerrado and Mata dos Cocais | *Attalea speciosa* Mart. ex Spreng. | Arecaceae | T | N | N | Wound | IPEC |
| Franco & Barros 2006 | Cerrado and Mata dos Cocais | *Bowdichia virgilioides* Kunth | Fabaceae | T | N | N | Headache | SSNEC |
| Franco & Barros 2006 | Cerrado and Mata dos Cocais | *Bowdichia virgilioides* Kunth | Fabaceae | T | N | N | Inflammation | SSNEC |
| Franco & Barros 2006 | Cerrado and Mata dos Cocais | *Bowdichia virgilioides* Kunth | Fabaceae | T | N | N | Influenza | DRS |
| Franco & Barros 2006 | Cerrado and Mata dos Cocais | *Bromelia karatas* L. | Bromeliaceae | H | N | N | Influenza | DRS |
| Franco & Barros 2006 | Cerrado and Mata dos Cocais | *Bromelia karatas* L. | Bromeliaceae | H | N | N | Whooping Cough | CIPD |
| Franco & Barros 2006 | Cerrado and Mata dos Cocais | *Campomanesia aromatica* (Aubl.) Griseb. | Myrtaceae | S | N | E | Cardiac problems | DCS |
| Franco & Barros 2006 | Cerrado and Mata dos Cocais | *Carica papaya* L. | Caricaceae | T | E | E | Constipation | DDS |
| Franco & Barros 2006 | Cerrado and Mata dos Cocais | *Caryocar coriaceum* Wittm. | Caryocaraceae | T | N | N | Influenza | DRS |
| Franco & Barros 2006 | Cerrado and Mata dos Cocais | *Cecropia glaziovii* Snethl. | Urticaceae | T | N | E | Influenza | DRS |
| Franco & Barros 2006 | Cerrado and Mata dos Cocais | *Cecropia glaziovii* Snethl. | Urticaceae | T | N | E | Influenza | DRS |
| Franco & Barros 2006 | Cerrado and Mata dos Cocais | *Cecropia glaziovii* Snethl. | Urticaceae | T | N | E | Swelling | SSNEC |
| Franco & Barros 2006 | Cerrado and Mata dos Cocais | *Cecropia glaziovii* Snethl. | Urticaceae | T | N | E | Swelling | SSNEC |
| Franco & Barros 2006 | Cerrado and Mata dos Cocais | *Chenopodium ambrosioides* L. | Amaranthaceae | H | N | N | Influenza | DRS |
| Franco & Barros 2006 | Cerrado and Mata dos Cocais | *Citrus aurantium* L. | Rutaceae | T | E | E | Constipation | DDS |
| Franco & Barros 2006 | Cerrado and Mata dos Cocais | *Citrus aurantium* L. | Rutaceae | T | E | E | Fever | SSNEC |
| Franco & Barros 2006 | Cerrado and Mata dos Cocais | *Citrus aurantium* L. | Rutaceae | T | E | E | Influenza | DRS |
| Franco & Barros 2006 | Cerrado and Mata dos Cocais | *Citrus limon* (L.) Osbeck | Rutaceae | T | E | E | Hypertension | DCS |
| Franco & Barros 2006 | Cerrado and Mata dos Cocais | *Citrus limon* (L.) Osbeck | Rutaceae | T | E | E | Influenza | DRS |
| Franco & Barros 2006 | Cerrado and Mata dos Cocais | *Citrus sinensis* (L.) Osbeck | Rutaceae | T | E | E | Calming | MBD |
| Franco & Barros 2006 | Cerrado and Mata dos Cocais | *Citrus sinensis* (L.) Osbeck | Rutaceae | T | E | E | Calming | MBD |
| Franco & Barros 2006 | Cerrado and Mata dos Cocais | *Citrus sinensis* (L.) Osbeck | Rutaceae | T | E | E | Constipation | DDS |
| Franco & Barros 2006 | Cerrado and Mata dos Cocais | *Citrus sinensis* (L.) Osbeck | Rutaceae | T | E | E | Constipation | DDS |
| Franco & Barros 2006 | Cerrado and Mata dos Cocais | *Cocos nucifera* L. | Arecaceae | T | N | N | Hemorrhage | SSNEC |
| Franco & Barros 2006 | Cerrado and Mata dos Cocais | *Combretum leprosum* Mart. | Combretaceae | T | N | N | Hemorrhage | SSNEC |
| Franco & Barros 2006 | Cerrado and Mata dos Cocais | *Combretum leprosum* Mart. | Combretaceae | T | N | N | Stomach pain/Diarrhea | SSNEC |
| Franco & Barros 2006 | Cerrado and Mata dos Cocais | *Croton campestris* A.St.-Hil. | Euphorbiaceae | S | N | N | Constipation | DDS |
| Franco & Barros 2006 | Cerrado and Mata dos Cocais | *Croton campestris* A.St.-Hil. | Euphorbiaceae | S | N | N | Constipation | DDS |
| Franco & Barros 2006 | Cerrado and Mata dos Cocais | *Croton campestris* A.St.-Hil. | Euphorbiaceae | S | N | N | Influenza | DRS |
| Franco & Barros 2006 | Cerrado and Mata dos Cocais | *Croton campestris* A.St.-Hil. | Euphorbiaceae | S | N | N | Influenza | DRS |
| Franco & Barros 2006 | Cerrado and Mata dos Cocais | *Croton campestris* A.St.-Hil. | Euphorbiaceae | S | N | N | Pain | SSNEC |
| Franco & Barros 2006 | Cerrado and Mata dos Cocais | *Croton campestris* A.St.-Hil. | Euphorbiaceae | S | N | N | Pain | SSNEC |
| Franco & Barros 2006 | Cerrado and Mata dos Cocais | *Croton sonderianus* Müll.Arg. | Euphorbiaceae | S | N | E | Headache | SSNEC |
| Franco & Barros 2006 | Cerrado and Mata dos Cocais | *Croton sonderianus* Müll.Arg. | Euphorbiaceae | S | N | E | Swelling | SSNEC |
| Franco & Barros 2006 | Cerrado and Mata dos Cocais | *Cucurbita* pepo L. | Cucurbitaceae | H | E | E | Earache | DEMP |
| Franco & Barros 2006 | Cerrado and Mata dos Cocais | *Curatella americana* L. | Dilleniaceae | T | N | N | Inflammation | SSNEC |
| Franco & Barros 2006 | Cerrado and Mata dos Cocais | *Cymbopogon citratus* (DC.) Stapf | Poaceae | H | E | E | Calming | MBD |
| Franco & Barros 2006 | Cerrado and Mata dos Cocais | *Cymbopogon citratus* (DC.) Stapf | Poaceae | H | E | E | Hypertension | DCS |
| Franco & Barros 2006 | Cerrado and Mata dos Cocais | *Dioclea violacea* Mart. ex Benth. | Fabaceae | S | N | E | Influenza | DRS |
| Franco & Barros 2006 | Cerrado and Mata dos Cocais | *Eucalyptus globulus* Labill. | Myrtaceae | T | E | E | Influenza | DRS |
| Franco & Barros 2006 | Cerrado and Mata dos Cocais | *Euphorbia prostrata* Aiton | Euphorbiaceae | H | N | N | Stomach pain/Diarrhea | SSNEC |
| Franco & Barros 2006 | Cerrado and Mata dos Cocais | *Euphorbia prostrata* Aiton | Euphorbiaceae | H | N | N | Stomach pain/Diarrhea | SSNEC |
| Franco & Barros 2006 | Cerrado and Mata dos Cocais | *Gossypium herbaceum* L. | Malvaceae | S | E | E | Asthma | DRS |
| Franco & Barros 2006 | Cerrado and Mata dos Cocais | *Gossypium herbaceum* L. | Malvaceae | S | E | E | Asthma | DRS |
| Franco & Barros 2006 | Cerrado and Mata dos Cocais | *Gossypium herbaceum* L. | Malvaceae | S | E | E | Inflammation | SSNEC |
| Franco & Barros 2006 | Cerrado and Mata dos Cocais | *Gossypium herbaceum* L. | Malvaceae | S | E | E | Inflammation | SSNEC |
| Franco & Barros 2006 | Cerrado and Mata dos Cocais | *Gossypium herbaceum* L. | Malvaceae | S | E | E | Influenza | DRS |
| Franco & Barros 2006 | Cerrado and Mata dos Cocais | *Gossypium herbaceum* L. | Malvaceae | S | E | E | Influenza | DRS |
| Franco & Barros 2006 | Cerrado and Mata dos Cocais | *Handroanthus impetiginosus* Mattos | Bignoniaceae | T | N | N | Anemia | DBBO |
| Franco & Barros 2006 | Cerrado and Mata dos Cocais | *Heliotropium elongatum* (Lehm.) I.M.Johnst. | Boraginaceae | H | N | N | Influenza | DRS |
| Franco & Barros 2006 | Cerrado and Mata dos Cocais | *Heliotropium lanceolatum* Ruiz & Pav. | Boraginaceae | H | E | E | Depurative | DBBO |
| Franco & Barros 2006 | Cerrado and Mata dos Cocais | *Himatanthus sucuuba* (Spruce ex Müll.Arg.) Woodson | Apocynaceae | T | N | N | Cough | SSNEC |
| Franco & Barros 2006 | Cerrado and Mata dos Cocais | *Himatanthus sucuuba* (Spruce ex Müll.Arg.) Woodson | Apocynaceae | T | N | N | Depurative | DBBO |
| Franco & Barros 2006 | Cerrado and Mata dos Cocais | *Himatanthus sucuuba* (Spruce ex Müll.Arg.) Woodson | Apocynaceae | T | N | N | Inflammation | SSNEC |
| Franco & Barros 2006 | Cerrado and Mata dos Cocais | *Himatanthus sucuuba* (Spruce ex Müll.Arg.) Woodson | Apocynaceae | T | N | N | Inflammation of the throat | DRS |
| Franco & Barros 2006 | Cerrado and Mata dos Cocais | *Himatanthus sucuuba* (Spruce ex Müll.Arg.) Woodson | Apocynaceae | T | N | N | Influenza | DRS |
| Franco & Barros 2006 | Cerrado and Mata dos Cocais | *Holocalyx balansae* Micheli | Fabaceae | T | N | N | Headache | SSNEC |
| Franco & Barros 2006 | Cerrado and Mata dos Cocais | *Holocalyx balansae* Micheli | Fabaceae | T | N | N | Influenza | DRS |
| Franco & Barros 2006 | Cerrado and Mata dos Cocais | *Hymenaea stigonocarpa* Mart. ex Hayne | Fabaceae | T | N | N | Inflammation | SSNEC |
| Franco & Barros 2006 | Cerrado and Mata dos Cocais | *Hymenaea stigonocarpa* Mart. ex Hayne | Fabaceae | T | N | N | Influenza | DRS |
| Franco & Barros 2006 | Cerrado and Mata dos Cocais | *Ipomoea asarifolia* (Desr.) Roem. & Schult. | Convolvulaceae | H | N | E | Chickenpox | CIPD |
| Franco & Barros 2006 | Cerrado and Mata dos Cocais | *Jatropha curcas* L. | Euphorbiaceae | S | N | N | Inflammation | SSNEC |
| Franco & Barros 2006 | Cerrado and Mata dos Cocais | *Justicia pectoralis* Jacq. | Acanthaceae | H | N | N | Headache | SSNEC |
| Franco & Barros 2006 | Cerrado and Mata dos Cocais | *Lecythis pisonis* Cambess. | Lecythidaceae | T | N | N | Itchiness | DSST |
| Franco & Barros 2006 | Cerrado and Mata dos Cocais | *Libidibia ferrea* (Mart. ex Tul.) L.P.Queiroz | Fabaceae | T | N | N | Inflammation | SSNEC |
| Franco & Barros 2006 | Cerrado and Mata dos Cocais | *Libidibia ferrea* (Mart. ex Tul.) L.P.Queiroz | Fabaceae | T | N | N | Inflammation | SSNEC |
| Franco & Barros 2006 | Cerrado and Mata dos Cocais | *Libidibia ferrea* (Mart. ex Tul.) L.P.Queiroz | Fabaceae | T | N | N | Kidney pain | DGS |
| Franco & Barros 2006 | Cerrado and Mata dos Cocais | *Libidibia ferrea* (Mart. ex Tul.) L.P.Queiroz | Fabaceae | T | N | N | Kidney pain | DGS |
| Franco & Barros 2006 | Cerrado and Mata dos Cocais | *Libidibia ferrea* (Mart. ex Tul.) L.P.Queiroz | Fabaceae | T | N | N | Pain in spinal column | DMS |
| Franco & Barros 2006 | Cerrado and Mata dos Cocais | *Libidibia ferrea* (Mart. ex Tul.) L.P.Queiroz | Fabaceae | T | N | N | Pain in spinal column | DMS |
| Franco & Barros 2006 | Cerrado and Mata dos Cocais | *Lippia alba* (Mill.) N.E.Br. | Verbenaceae | S | N | N | Hoarseness | DRS |
| Franco & Barros 2006 | Cerrado and Mata dos Cocais | *Lippia alba* (Mill.) N.E.Br. | Verbenaceae | S | N | N | Influenza | DRS |
| Franco & Barros 2006 | Cerrado and Mata dos Cocais | *Luehea divaricata* Mart. & Zucc. | Malvaceae | T | N | N | Anemia | DBBO |
| Franco & Barros 2006 | Cerrado and Mata dos Cocais | *Luehea divaricata* Mart. & Zucc. | Malvaceae | T | N | N | Depurative | DBBO |
| Franco & Barros 2006 | Cerrado and Mata dos Cocais | *Luehea divaricata* Mart. & Zucc. | Malvaceae | T | N | N | Hepatitis | CIPD |
| Franco & Barros 2006 | Cerrado and Mata dos Cocais | *Luehea divaricata* Mart. & Zucc. | Malvaceae | T | N | N | Inflammation | SSNEC |
| Franco & Barros 2006 | Cerrado and Mata dos Cocais | *Luehea divaricata* Mart. & Zucc. | Malvaceae | T | N | N | Influenza | DRS |
| Franco & Barros 2006 | Cerrado and Mata dos Cocais | *Malpighia glabra* L. | Malpighiaceae | T | E | E | Influenza | DRS |
| Franco & Barros 2006 | Cerrado and Mata dos Cocais | *Malva sylvestris* L. | Malvaceae | H | E | E | Hoarseness | DRS |
| Franco & Barros 2006 | Cerrado and Mata dos Cocais | *Malva sylvestris* L. | Malvaceae | H | E | E | Influenza | DRS |
| Franco & Barros 2006 | Cerrado and Mata dos Cocais | *Momordica charantia* L. | Cucurbitaceae | H | E | E | Itchiness | DSST |
| Franco & Barros 2006 | Cerrado and Mata dos Cocais | *Momordica charantia* L. | Cucurbitaceae | H | E | E | Lice | CIPD |
| Franco & Barros 2006 | Cerrado and Mata dos Cocais | *Myracrodruon urundeuva* Allemão | Anacardiaceae | T | N | N | Gastritis | DDS |
| Franco & Barros 2006 | Cerrado and Mata dos Cocais | *Myracrodruon urundeuva* Allemão | Anacardiaceae | T | N | N | Inflammation | SSNEC |
| Franco & Barros 2006 | Cerrado and Mata dos Cocais | *Ocimum americanum* L. | Lamiaceae | H | E | E | Influenza | DRS |
| Franco & Barros 2006 | Cerrado and Mata dos Cocais | *Ocimum gratissimum* L. | Lamiaceae | H | E | E | Influenza | DRS |
| Franco & Barros 2006 | Cerrado and Mata dos Cocais | *Operculina macrocarpa* (L.) Urb. | Convolvulaceae | H | N | N | Depurative | DBBO |
| Franco & Barros 2006 | Cerrado and Mata dos Cocais | *Operculina macrocarpa* (L.) Urb. | Convolvulaceae | H | N | N | Laxative | DDS |
| Franco & Barros 2006 | Cerrado and Mata dos Cocais | *Persea americana* Mill. | Lauraceae | T | E | E | Kidney pain | DGS |
| Franco & Barros 2006 | Cerrado and Mata dos Cocais | *Phanera flexuosa* (Moric.) L.P.Queiroz | Fabaceae | S | N | E | Fever | SSNEC |
| Franco & Barros 2006 | Cerrado and Mata dos Cocais | *Phanera flexuosa* (Moric.) L.P.Queiroz | Fabaceae | S | N | E | Kidney pain | DGS |
| Franco & Barros 2006 | Cerrado and Mata dos Cocais | *Phanera flexuosa* (Moric.) L.P.Queiroz | Fabaceae | S | N | E | Stomach pain/Diarrhea | SSNEC |
| Franco & Barros 2006 | Cerrado and Mata dos Cocais | *Phyllanthus niruri* L. | Phyllanthaceae | H | N | N | Inflammation of the liver | DDS |
| Franco & Barros 2006 | Cerrado and Mata dos Cocais | *Phyllanthus niruri* L. | Phyllanthaceae | H | N | N | Influenza | DRS |
| Franco & Barros 2006 | Cerrado and Mata dos Cocais | *Pimpinella anisum* L. | Apiaceae | H | E | E | Fever | SSNEC |
| Franco & Barros 2006 | Cerrado and Mata dos Cocais | *Platonia insignis* Mart. | Clusiaceae | T | N | N | Stomach pain/Diarrhea | SSNEC |
| Franco & Barros 2006 | Cerrado and Mata dos Cocais | *Plectranthus barbatus* Andr. | Lamiaceae | H | E | E | Hangover | MBD |
| Franco & Barros 2006 | Cerrado and Mata dos Cocais | *Plectranthus barbatus* Andr. | Lamiaceae | H | E | E | Inflammation of the liver | DDS |
| Franco & Barros 2006 | Cerrado and Mata dos Cocais | *Plectranthus barbatus* Andr. | Lamiaceae | H | E | E | Intestinal pain | SSNEC |
| Franco & Barros 2006 | Cerrado and Mata dos Cocais | *Plumbago scandens* L. | Plumbaginaceae | H | N | E | Itchiness | DSST |
| Franco & Barros 2006 | Cerrado and Mata dos Cocais | *Psidium guajava* L. | Myrtaceae | S | E | E | Stomach pain/Diarrhea | SSNEC |
| Franco & Barros 2006 | Cerrado and Mata dos Cocais | *Punica granatum* L. | Lythraceae | T | E | E | Influenza | DRS |
| Franco & Barros 2006 | Cerrado and Mata dos Cocais | *Ricinus communis* L. | Euphorbiaceae | S | N | E | Headache | SSNEC |
| Franco & Barros 2006 | Cerrado and Mata dos Cocais | *Ricinus communis* L. | Euphorbiaceae | S | N | E | Headache | SSNEC |
| Franco & Barros 2006 | Cerrado and Mata dos Cocais | *Ricinus communis* L. | Euphorbiaceae | S | N | E | Helminthiasis | CIPD |
| Franco & Barros 2006 | Cerrado and Mata dos Cocais | *Ricinus communis* L. | Euphorbiaceae | S | N | E | Helminthiasis | CIPD |
| Franco & Barros 2006 | Cerrado and Mata dos Cocais | *Ricinus communis* L. | Euphorbiaceae | S | N | E | Laxative | DDS |
| Franco & Barros 2006 | Cerrado and Mata dos Cocais | *Ricinus communis* L. | Euphorbiaceae | S | N | E | Laxative | DDS |
| Franco & Barros 2006 | Cerrado and Mata dos Cocais | *Ruta graveolens* L. | Rutaceae | H | E | E | Toothache | DDS |
| Franco & Barros 2006 | Cerrado and Mata dos Cocais | *Scoparia dulcis* L. | Plantaginaceae | H | N | N | Inflammation | SSNEC |
| Franco & Barros 2006 | Cerrado and Mata dos Cocais | *Scoparia dulcis* L. | Plantaginaceae | H | N | N | Influenza | DRS |
| Franco & Barros 2006 | Cerrado and Mata dos Cocais | *Senegalia langsPainffii (Benth.) Seigler & Ebinger* | Fabaceae | S | N | E | Influenza | DRS |
| Franco & Barros 2006 | Cerrado and Mata dos Cocais | *Senegalia langsPainffii (Benth.) Seigler & Ebinger* | Fabaceae | S | N | E | Influenza | DRS |
| Franco & Barros 2006 | Cerrado and Mata dos Cocais | *Senegalia langsPainffii (Benth.) Seigler & Ebinger* | Fabaceae | S | N | E | Pain in spinal column | DMS |
| Franco & Barros 2006 | Cerrado and Mata dos Cocais | *Senegalia langsPainffii (Benth.) Seigler & Ebinger* | Fabaceae | S | N | E | Pain in spinal column | DMS |
| Franco & Barros 2006 | Cerrado and Mata dos Cocais | *Senegalia langsPainffii (Benth.) Seigler & Ebinger* | Fabaceae | S | N | E | Wound | IPEC |
| Franco & Barros 2006 | Cerrado and Mata dos Cocais | *Senegalia langsPainffii (Benth.) Seigler & Ebinger* | Fabaceae | S | N | E | Wound | IPEC |
| Franco & Barros 2006 | Cerrado and Mata dos Cocais | *Senna alata* (L.) Roxb. | Fabaceae | S | N | N | Influenza | DRS |
| Franco & Barros 2006 | Cerrado and Mata dos Cocais | *Senna obtusifolia* (L.) H.S.Irwin & Barneby | Fabaceae | S | N | E | Influenza | DRS |
| Franco & Barros 2006 | Cerrado and Mata dos Cocais | *Senna obtusifolia* (L.) H.S.Irwin & Barneby | Fabaceae | S | N | E | Influenza | DRS |
| Franco & Barros 2006 | Cerrado and Mata dos Cocais | *Senna obtusifolia* (L.) H.S.Irwin & Barneby | Fabaceae | S | N | E | Influenza | DRS |
| Franco & Barros 2006 | Cerrado and Mata dos Cocais | *Senna obtusifolia* (L.) H.S.Irwin & Barneby | Fabaceae | S | N | E | Laxative | DDS |
| Franco & Barros 2006 | Cerrado and Mata dos Cocais | *Senna obtusifolia* (L.) H.S.Irwin & Barneby | Fabaceae | S | N | E | Laxative | DDS |
| Franco & Barros 2006 | Cerrado and Mata dos Cocais | *Senna obtusifolia* (L.) H.S.Irwin & Barneby | Fabaceae | S | N | E | Laxative | DDS |
| Franco & Barros 2006 | Cerrado and Mata dos Cocais | *Senna occidentalis* (L.) Link | Fabaceae | S | N | N | Dermatophytosis | CIPD |
| Franco & Barros 2006 | Cerrado and Mata dos Cocais | *Senna occidentalis* (L.) Link | Fabaceae | S | N | N | Dermatophytosis | CIPD |
| Franco & Barros 2006 | Cerrado and Mata dos Cocais | *Senna occidentalis* (L.) Link | Fabaceae | S | N | N | Helminthiasis | CIPD |
| Franco & Barros 2006 | Cerrado and Mata dos Cocais | *Senna occidentalis* (L.) Link | Fabaceae | S | N | N | Helminthiasis | CIPD |
| Franco & Barros 2006 | Cerrado and Mata dos Cocais | *Senna occidentalis* (L.) Link | Fabaceae | S | N | N | Influenza | DRS |
| Franco & Barros 2006 | Cerrado and Mata dos Cocais | *Senna occidentalis* (L.) Link | Fabaceae | S | N | N | Influenza | DRS |
| Franco & Barros 2006 | Cerrado and Mata dos Cocais | *Simaba maiana* Casar. | Simaroubaceae | S | N | E | Depurative | DBBO |
| Franco & Barros 2006 | Cerrado and Mata dos Cocais | *Solanum paniculatum* L. | Solanaceae | S | N | N | Wound | IPEC |
| Franco & Barros 2006 | Cerrado and Mata dos Cocais | *Spondias purpurea* L. | Anacardiaceae | T | E | E | Stomach pain/Diarrhea | SSNEC |
| Franco & Barros 2006 | Cerrado and Mata dos Cocais | *Tachigali vulgaris* L.G.Silva & H.C.Lima | Fabaceae | T | N | N | Wound | IPEC |
| Franco & Barros 2006 | Cerrado and Mata dos Cocais | *Tamarindus indica* L. | Fabaceae | T | E | E | Helminthiasis | CIPD |
| Franco & Barros 2006 | Cerrado and Mata dos Cocais | *Tarenaya spinosa* (Jacq.) Raf. | Capparaceae | H | N | N | Cough | SSNEC |
| Franco & Barros 2006 | Cerrado and Mata dos Cocais | *Tarenaya spinosa* (Jacq.) Raf. | Capparaceae | H | N | N | Cough | SSNEC |
| Franco & Barros 2006 | Cerrado and Mata dos Cocais | *Tarenaya spinosa* (Jacq.) Raf. | Capparaceae | H | N | N | Inflammation of the throat | DRS |
| Franco & Barros 2006 | Cerrado and Mata dos Cocais | *Tarenaya spinosa* (Jacq.) Raf. | Capparaceae | H | N | N | Inflammation of the throat | DRS |
| Franco & Barros 2006 | Cerrado and Mata dos Cocais | *Tarenaya spinosa* (Jacq.) Raf. | Capparaceae | H | N | N | Influenza | DRS |
| Franco & Barros 2006 | Cerrado and Mata dos Cocais | *Tarenaya spinosa* (Jacq.) Raf. | Capparaceae | H | N | N | Influenza | DRS |
| Franco & Barros 2006 | Cerrado and Mata dos Cocais | *Turnera ulmifolia* L. | Passifloraceae | H | E | E | Wound | IPEC |
| Franco & Barros 2006 | Cerrado and Mata dos Cocais | *Vachellia farnesiana* (L.) Wight & Arn. | Fabaceae | S | N | E | Fever | SSNEC |
| Franco & Barros 2006 | Cerrado and Mata dos Cocais | *Vachellia farnesiana* (L.) Wight & Arn. | Fabaceae | S | N | E | Fever | SSNEC |
| Franco & Barros 2006 | Cerrado and Mata dos Cocais | *Vachellia farnesiana* (L.) Wight & Arn. | Fabaceae | S | N | E | Headache | SSNEC |
| Franco & Barros 2006 | Cerrado and Mata dos Cocais | *Vachellia farnesiana* (L.) Wight & Arn. | Fabaceae | S | N | E | Headache | SSNEC |
| Franco & Barros 2006 | Cerrado and Mata dos Cocais | *Vachellia farnesiana* (L.) Wight & Arn. | Fabaceae | S | N | E | Hoarseness | DRS |
| Franco & Barros 2006 | Cerrado and Mata dos Cocais | *Vachellia farnesiana* (L.) Wight & Arn. | Fabaceae | S | N | E | Hoarseness | DRS |
| Franco & Barros 2006 | Cerrado and Mata dos Cocais | *Vachellia farnesiana* (L.) Wight & Arn. | Fabaceae | S | N | E | Inflammation of the throat | DRS |
| Franco & Barros 2006 | Cerrado and Mata dos Cocais | *Vachellia farnesiana* (L.) Wight & Arn. | Fabaceae | S | N | E | Inflammation of the throat | DRS |
| Franco & Barros 2006 | Cerrado and Mata dos Cocais | *Vachellia farnesiana* (L.) Wight & Arn. | Fabaceae | S | N | E | Influenza | DRS |
| Franco & Barros 2006 | Cerrado and Mata dos Cocais | *Vachellia farnesiana* (L.) Wight & Arn. | Fabaceae | S | N | E | Influenza | DRS |
| Franco & Barros 2006 | Cerrado and Mata dos Cocais | *Vachellia farnesiana* (L.) Wight & Arn. | Fabaceae | S | N | E | Stomach pain/Diarrhea | SSNEC |
| Franco & Barros 2006 | Cerrado and Mata dos Cocais | *Vachellia farnesiana* (L.) Wight & Arn. | Fabaceae | S | N | E | Stomach pain/Diarrhea | SSNEC |
| Franco & Barros 2006 | Cerrado and Mata dos Cocais | *Vachellia farnesiana* (L.) Wight & Arn. | Fabaceae | S | N | E | Stroke | DCS |
| Franco & Barros 2006 | Cerrado and Mata dos Cocais | *Vachellia farnesiana* (L.) Wight & Arn. | Fabaceae | S | N | E | Stroke | DCS |
| Franco & Barros 2006 | Cerrado and Mata dos Cocais | *Vitex triflora* Vahl | Lamiaceae | T | N | N | Warts | CIPD |
| Franco & Barros 2006 | Cerrado and Mata dos Cocais | *Wilbrandia verticillata* (Vell.) Cogn. | Cucurbitaceae | S | N | E | Snake bite | IPEC |
| Franco & Barros 2006 | Cerrado and Mata dos Cocais | *Ximenia americana* L. | Ximeniaceae | T | N | N | Dyspnea | SSNEC |
| Franco & Barros 2006 | Cerrado and Mata dos Cocais | *Ximenia americana* L. | Ximeniaceae | T | N | N | Inflammation | SSNEC |
| Franco & Barros 2006 | Cerrado and Mata dos Cocais | *Ximenia americana* L. | Ximeniaceae | T | N | N | Wound | IPEC |
| Freitas & Fernandes 2006 | Amazon | *Abuta grandifolia* (Mart.) Sandwith | Menispermaceae | S | N | N | Blow, punch | IPEC |
| Freitas & Fernandes 2006 | Amazon | *Abuta grandifolia* (Mart.) Sandwith | Menispermaceae | S | N | N | Blow, punch | IPEC |
| Freitas & Fernandes 2006 | Amazon | *Abuta grandifolia* (Mart.) Sandwith | Menispermaceae | S | N | N | Conjunctivitis | DEA |
| Freitas & Fernandes 2006 | Amazon | *Abuta grandifolia* (Mart.) Sandwith | Menispermaceae | S | N | N | Inflammation | SSNEC |
| Freitas & Fernandes 2006 | Amazon | *Abuta grandifolia* (Mart.) Sandwith | Menispermaceae | S | N | N | Torsion | SSNEC |
| Freitas & Fernandes 2006 | Amazon | *Abuta grandifolia* (Mart.) Sandwith | Menispermaceae | S | N | N | Torsion | SSNEC |
| Freitas & Fernandes 2006 | Amazon | *Borreria verticillata* (L.) G.Mey. | Rubiaceae | H | N | N | Hemorrhoids | DCS |
| Freitas & Fernandes 2006 | Amazon | *Borreria verticillata* (L.) G.Mey. | Rubiaceae | H | N | N | Influenza | DRS |
| Freitas & Fernandes 2006 | Amazon | *Brosimum parinarioides* Ducke | Moraceae | T | N | N | Hemorrhoids | DCS |
| Freitas & Fernandes 2006 | Amazon | *Brosimum parinarioides* Ducke | Moraceae | T | N | N | Inflammation | SSNEC |
| Freitas & Fernandes 2006 | Amazon | *Brosimum parinarioides* Ducke | Moraceae | T | N | N | Malnutrition | ENMD |
| Freitas & Fernandes 2006 | Amazon | *Brosimum parinarioides* Ducke | Moraceae | T | N | N | Stomach pain/Diarrhea | SSNEC |
| Freitas & Fernandes 2006 | Amazon | *Brosimum parinarioides* Ducke | Moraceae | T | N | N | Weakness | SSNEC |
| Freitas & Fernandes 2006 | Amazon | *Cecropia obtusa* Trécul | Urticaceae | T | N | N | Albumin | SSNEC |
| Freitas & Fernandes 2006 | Amazon | *Cecropia obtusa* Trécul | Urticaceae | T | N | N | Inflammation | SSNEC |
| Freitas & Fernandes 2006 | Amazon | *Cecropia obtusa* Trécul | Urticaceae | T | N | N | Swelling | SSNEC |
| Freitas & Fernandes 2006 | Amazon | *Cecropia palmata* Willd. | Urticaceae | T | N | N | Anemia | DBBO |
| Freitas & Fernandes 2006 | Amazon | *Cecropia palmata* Willd. | Urticaceae | T | N | N | Swelling | SSNEC |
| Freitas & Fernandes 2006 | Amazon | *Cinnamomum verum* J. Presl | Lauraceae | T | E | E | Poor digestion | DDS |
| Freitas & Fernandes 2006 | Amazon | *Cinnamomum verum* J. Presl | Lauraceae | T | E | E | Weakness | SSNEC |
| Freitas & Fernandes 2006 | Amazon | *Clavija lancifolia* Desf. | Theophrastaceae | T | N | N | Rheumatism | DMS |
| Freitas & Fernandes 2006 | Amazon | *Clavija lancifolia* Desf. | Theophrastaceae | T | N | N | Rheumatism | DMS |
| Freitas & Fernandes 2006 | Amazon | *Clavija lancifolia* Desf. | Theophrastaceae | T | N | N | Sexual impotence | DGS |
| Freitas & Fernandes 2006 | Amazon | *Clavija lancifolia* Desf. | Theophrastaceae | T | N | N | Sexual impotence | DGS |
| Freitas & Fernandes 2006 | Amazon | *Clusia insignis* Mart. | Clusiaceae | T | N | N | Inflammation | SSNEC |
| Freitas & Fernandes 2006 | Amazon | *Clusia insignis* Mart. | Clusiaceae | T | N | N | Torsion | SSNEC |
| Freitas & Fernandes 2006 | Amazon | *Dalbergia monetaria* L.f. | Fabaceae | S | N | N | Anti-emetic | SSNEC |
| Freitas & Fernandes 2006 | Amazon | *Dalbergia monetaria* L.f. | Fabaceae | S | N | N | Inflammation | SSNEC |
| Freitas & Fernandes 2006 | Amazon | *Dalbergia monetaria* L.f. | Fabaceae | S | N | N | Wound | IPEC |
| Freitas & Fernandes 2006 | Amazon | *Davilla rugosa* Poir. | Dilleniaceae | S | N | N | Dysentry | CIPD |
| Freitas & Fernandes 2006 | Amazon | *Davilla rugosa* Poir. | Dilleniaceae | S | N | N | Inflammation | SSNEC |
| Freitas & Fernandes 2006 | Amazon | *Davilla rugosa* Poir. | Dilleniaceae | S | N | N | Warts | CIPD |
| Freitas & Fernandes 2006 | Amazon | *Dimorphandra mollis* Benth. | Fabaceae | T | N | N | Inflammation | SSNEC |
| Freitas & Fernandes 2006 | Amazon | *Geissospermum sericeum* Miers | Apocynaceae | T | N | N | Itchiness | DSST |
| Freitas & Fernandes 2006 | Amazon | *Geissospermum sericeum* Miers | Apocynaceae | T | N | N | Itchiness | DSST |
| Freitas & Fernandes 2006 | Amazon | *Geissospermum sericeum* Miers | Apocynaceae | T | N | N | Swollen legs | DMS |
| Freitas & Fernandes 2006 | Amazon | *Geissospermum sericeum* Miers | Apocynaceae | T | N | N | Swollen legs | DMS |
| Freitas & Fernandes 2006 | Amazon | *Geissospermum sericeum* Miers | Apocynaceae | T | N | N | Urinary infection | DGS |
| Freitas & Fernandes 2006 | Amazon | *Geissospermum sericeum* Miers | Apocynaceae | T | N | N | Urinary infection | DGS |
| Freitas & Fernandes 2006 | Amazon | *Himatanthus sucuuba* (Spruce ex Müll.Arg.) Woodson | Apocynaceae | T | N | N | Amebiasis | CIPD |
| Freitas & Fernandes 2006 | Amazon | *Himatanthus sucuuba* (Spruce ex Müll.Arg.) Woodson | Apocynaceae | T | N | N | Inflammation | SSNEC |
| Freitas & Fernandes 2006 | Amazon | *Himatanthus sucuuba* (Spruce ex Müll.Arg.) Woodson | Apocynaceae | T | N | N | Mycosis | CIPD |
| Freitas & Fernandes 2006 | Amazon | *Himatanthus sucuuba* (Spruce ex Müll.Arg.) Woodson | Apocynaceae | T | N | N | Stomach pain/Diarrhea | SSNEC |
| Freitas & Fernandes 2006 | Amazon | *Himatanthus sucuuba* (Spruce ex Müll.Arg.) Woodson | Apocynaceae | T | N | N | Wound | IPEC |
| Freitas & Fernandes 2006 | Amazon | *Hyperbaena hassleri* Diels | Menispermaceae | S | N | E | Blow, punch | IPEC |
| Freitas & Fernandes 2006 | Amazon | *Hyperbaena hassleri* Diels | Menispermaceae | S | N | E | Blow, punch | IPEC |
| Freitas & Fernandes 2006 | Amazon | *Hyperbaena hassleri* Diels | Menispermaceae | S | N | E | Torn muscle | IPEC |
| Freitas & Fernandes 2006 | Amazon | *Hyperbaena hassleri* Diels | Menispermaceae | S | N | E | Torn muscle | IPEC |
| Freitas & Fernandes 2006 | Amazon | *Luehea divaricata* Mart. & Zucc. | Malvaceae | T | N | E | Diabetes | ENMD |
| Freitas & Fernandes 2006 | Amazon | *Luehea divaricata* Mart. & Zucc. | Malvaceae | T | N | E | Inflammation | SSNEC |
| Freitas & Fernandes 2006 | Amazon | *Luehea divaricata* Mart. & Zucc. | Malvaceae | T | N | E | Rheumatism | DMS |
| Freitas & Fernandes 2006 | Amazon | *Luehea divaricata* Mart. & Zucc. | Malvaceae | T | N | E | Rheumatism | DMS |
| Freitas & Fernandes 2006 | Amazon | *Parahancornia fasciculata* (Poir.) Benoist | Apocynaceae | T | N | N | Amebiasis | CIPD |
| Freitas & Fernandes 2006 | Amazon | *Parahancornia fasciculata* (Poir.) Benoist | Apocynaceae | T | N | N | Gastritis | DDS |
| Freitas & Fernandes 2006 | Amazon | *Parahancornia fasciculata* (Poir.) Benoist | Apocynaceae | T | N | N | Influenza | DRS |
| Freitas & Fernandes 2006 | Amazon | *Parahancornia fasciculata* (Poir.) Benoist | Apocynaceae | T | N | N | Intestinal problems | DDS |
| Freitas & Fernandes 2006 | Amazon | *Parahancornia fasciculata* (Poir.) Benoist | Apocynaceae | T | N | N | Respiratory problems | DRS |
| Freitas & Fernandes 2006 | Amazon | *Parahancornia fasciculata* (Poir.) Benoist | Apocynaceae | T | N | N | Stomach pain/Diarrhea | SSNEC |
| Freitas & Fernandes 2006 | Amazon | *Parahancornia fasciculata* (Poir.) Benoist | Apocynaceae | T | N | N | Weakness | SSNEC |
| Freitas & Fernandes 2006 | Amazon | *Pilocarpus carajaensis* Skorupa | Rutaceae | T | N | N | Hair loss | DSST |
| Freitas & Fernandes 2006 | Amazon | *Pilocarpus carajaensis* Skorupa | Rutaceae | T | N | N | Hair loss | DSST |
| Freitas & Fernandes 2006 | Amazon | *Pilocarpus carajaensis* Skorupa | Rutaceae | T | N | N | Inflammation | SSNEC |
| Freitas & Fernandes 2006 | Amazon | *Pilocarpus carajaensis* Skorupa | Rutaceae | T | N | N | Inflammation | SSNEC |
| Freitas & Fernandes 2006 | Amazon | *Pilocarpus carajaensis* Skorupa | Rutaceae | T | N | N | Rheumatism | DMS |
| Freitas & Fernandes 2006 | Amazon | *Pilocarpus carajaensis* Skorupa | Rutaceae | T | N | N | Rheumatism | DMS |
| Freitas & Fernandes 2006 | Amazon | *Pilocarpus carajaensis* Skorupa | Rutaceae | T | N | N | Toothache | DDS |
| Freitas & Fernandes 2006 | Amazon | *Pilocarpus carajaensis* Skorupa | Rutaceae | T | N | N | Toothache | DDS |
| Freitas & Fernandes 2006 | Amazon | *Piper ottonoides* Yunck. | Piperaceae | S | N | N | Toothache | DDS |
| Freitas & Fernandes 2006 | Amazon | *Piper peltatum* L. | Piperaceae | S | N | N | Cough | SSNEC |
| Freitas & Fernandes 2006 | Amazon | *Piper peltatum* L. | Piperaceae | S | N | N | Swelling | SSNEC |
| Freitas & Fernandes 2006 | Amazon | *Polygala spectabilis* DC. | Polygalaceae | H | N | N | Hemorrhoids | DCS |
| Freitas & Fernandes 2006 | Amazon | *Polygala spectabilis* DC. | Polygalaceae | H | N | N | Stomach pain/Diarrhea | SSNEC |
| Freitas & Fernandes 2006 | Amazon | *Psychotria colorata* (Willd. ex Schult.) Müll.Arg. | Rubiaceae | H | N | N | Colic | SSNEC |
| Freitas & Fernandes 2006 | Amazon | *Schnella guianensis* (Aubl.) Wunderlin | Fabaceae | S | N | N | Hemorrhoids | DCS |
| Freitas & Fernandes 2006 | Amazon | *Schnella guianensis* (Aubl.) Wunderlin | Fabaceae | S | N | N | Hemorrhoids | DCS |
| Freitas & Fernandes 2006 | Amazon | *Schnella guianensis* (Aubl.) Wunderlin | Fabaceae | S | N | N | High blood pressure | DCS |
| Freitas & Fernandes 2006 | Amazon | *Schnella guianensis* (Aubl.) Wunderlin | Fabaceae | S | N | N | High blood pressure | DCS |
| Freitas & Fernandes 2006 | Amazon | *Schnella guianensis* (Aubl.) Wunderlin | Fabaceae | S | N | N | Rheumatism | DMS |
| Freitas & Fernandes 2006 | Amazon | *Schnella guianensis* (Aubl.) Wunderlin | Fabaceae | S | N | N | Rheumatism | DMS |
| Freitas & Fernandes 2006 | Amazon | *Schnella guianensis* (Aubl.) Wunderlin | Fabaceae | S | N | N | Stomach pain/Diarrhea | SSNEC |
| Freitas & Fernandes 2006 | Amazon | *Schnella guianensis* (Aubl.) Wunderlin | Fabaceae | S | N | N | Stomach pain/Diarrhea | SSNEC |
| Freitas & Fernandes 2006 | Amazon | *Scoparia dulcis* L. | Plantaginaceae | H | N | N | Itchiness | DSST |
| Freitas & Fernandes 2006 | Amazon | *Scoparia dulcis* L. | Plantaginaceae | H | N | N | Toothache | DDS |
| Freitas & Fernandes 2006 | Amazon | *Senna fruticosa* (Mill.) H.S.Irwin & Barneby | Fabaceae | S | N | N | Cough | SSNEC |
| Freitas & Fernandes 2006 | Amazon | *Senna fruticosa* (Mill.) H.S.Irwin & Barneby | Fabaceae | S | N | N | Wound | IPEC |
| Freitas & Fernandes 2006 | Amazon | *Siparuna decipiens* (Tul.) A.DC. | Siparunaceae | T | N | N | Headache | SSNEC |
| Freitas & Fernandes 2006 | Amazon | *Siparuna decipiens* (Tul.) A.DC. | Siparunaceae | T | N | N | Weakness | SSNEC |
| Freitas & Fernandes 2006 | Amazon | *Stryphnodendron aSTDringens (Mart.) Coville* | Fabaceae | T | N | E | Amebiasis | CIPD |
| Freitas & Fernandes 2006 | Amazon | *Stryphnodendron aSTDringens (Mart.) Coville* | Fabaceae | T | N | E | Inflammation | SSNEC |
| Freitas & Fernandes 2006 | Amazon | *Stryphnodendron aSTDringens (Mart.) Coville* | Fabaceae | T | N | E | Stomach pain/Diarrhea | SSNEC |
| Freitas & Fernandes 2006 | Amazon | *Stryphnodendron aSTDringens (Mart.) Coville* | Fabaceae | T | N | E | Ulcer | DDS |
| Freitas & Fernandes 2006 | Amazon | *Stryphnodendron aSTDringens (Mart.) Coville* | Fabaceae | T | N | E | Vaginal discharge | DGS |
| Freitas & Fernandes 2006 | Amazon | *Stryphnodendron aSTDringens (Mart.) Coville* | Fabaceae | T | N | E | Wound | IPEC |
| Freitas & Fernandes 2006 | Amazon | *Stryphnodendron aSTDringens (Mart.) Coville* | Fabaceae | T | N | E | Wound | IPEC |
| Freitas & Fernandes 2006 | Amazon | *Uncaria guianensis* (Aubl.) J.F.Gmel. | Rubiaceae | S | N | N | Diabetes | ENMD |
| Freitas & Fernandes 2006 | Amazon | *Uncaria guianensis* (Aubl.) J.F.Gmel. | Rubiaceae | S | N | N | Diabetes | ENMD |
| Freitas & Fernandes 2006 | Amazon | *Uncaria guianensis* (Aubl.) J.F.Gmel. | Rubiaceae | S | N | N | Inflammation | SSNEC |
| Freitas & Fernandes 2006 | Amazon | *Uncaria guianensis* (Aubl.) J.F.Gmel. | Rubiaceae | S | N | N | Inflammation | SSNEC |
| Freitas & Fernandes 2006 | Amazon | *Uncaria guianensis* (Aubl.) J.F.Gmel. | Rubiaceae | S | N | N | Toothache | DDS |
| Freitas & Fernandes 2006 | Amazon | *Uncaria guianensis* (Aubl.) J.F.Gmel. | Rubiaceae | S | N | N | Toothache | DDS |
| Garlet & Irgang 2001 | Pampa and Atlantic Forest | *Acanthospermum australe* (Loefl.) Kuntze | Asteraceae | H | N | N | Antimicrobial | CIPD |
| Garlet & Irgang 2001 | Pampa and Atlantic Forest | *Acanthospermum australe* (Loefl.) Kuntze | Asteraceae | H | N | N | Bladder problems | DGS |
| Garlet & Irgang 2001 | Pampa and Atlantic Forest | *Acanthospermum australe* (Loefl.) Kuntze | Asteraceae | H | N | N | Improves digestion | DDS |
| Garlet & Irgang 2001 | Pampa and Atlantic Forest | *Acanthospermum australe* (Loefl.) Kuntze | Asteraceae | H | N | N | Kidney problems | DGS |
| Garlet & Irgang 2001 | Pampa and Atlantic Forest | *Acanthospermum australe* (Loefl.) Kuntze | Asteraceae | H | N | N | Liver problems | DDS |
| Garlet & Irgang 2001 | Pampa and Atlantic Forest | *Acanthospermum australe* (Loefl.) Kuntze | Asteraceae | H | N | N | Stomach problems | DDS |
| Garlet & Irgang 2001 | Pampa and Atlantic Forest | *Achillea millefolium* L. | Asteraceae | H | E | E | Infant colic | SSNEC |
| Garlet & Irgang 2001 | Pampa and Atlantic Forest | *Achillea millefolium* L. | Asteraceae | H | E | E | Infant colic | SSNEC |
| Garlet & Irgang 2001 | Pampa and Atlantic Forest | *Achillea millefolium* L. | Asteraceae | H | E | E | Inflammation | SSNEC |
| Garlet & Irgang 2001 | Pampa and Atlantic Forest | *Achillea millefolium* L. | Asteraceae | H | E | E | Inflammation | SSNEC |
| Garlet & Irgang 2001 | Pampa and Atlantic Forest | *Achillea millefolium* L. | Asteraceae | H | E | E | Influenza | DRS |
| Garlet & Irgang 2001 | Pampa and Atlantic Forest | *Achillea millefolium* L. | Asteraceae | H | E | E | Influenza | DRS |
| Garlet & Irgang 2001 | Pampa and Atlantic Forest | *Achillea millefolium* L. | Asteraceae | H | E | E | Undefined | SSNEC |
| Garlet & Irgang 2001 | Pampa and Atlantic Forest | *Achillea millefolium* L. | Asteraceae | H | E | E | Undefined | SSNEC |
| Garlet & Irgang 2001 | Pampa and Atlantic Forest | *Achyrocline satureioides* (Lam.) DC. | Asteraceae | S | N | N | Anti-emetic | SSNEC |
| Garlet & Irgang 2001 | Pampa and Atlantic Forest | *Achyrocline satureioides* (Lam.) DC. | Asteraceae | S | N | N | Cold sore | DDS |
| Garlet & Irgang 2001 | Pampa and Atlantic Forest | *Achyrocline satureioides* (Lam.) DC. | Asteraceae | S | N | N | Expectorant | SSNEC |
| Garlet & Irgang 2001 | Pampa and Atlantic Forest | *Achyrocline satureioides* (Lam.) DC. | Asteraceae | S | N | N | Headache | SSNEC |
| Garlet & Irgang 2001 | Pampa and Atlantic Forest | *Achyrocline satureioides* (Lam.) DC. | Asteraceae | S | N | N | High blood pressure | DCS |
| Garlet & Irgang 2001 | Pampa and Atlantic Forest | *Achyrocline satureioides* (Lam.) DC. | Asteraceae | S | N | N | Influenza | DRS |
| Garlet & Irgang 2001 | Pampa and Atlantic Forest | *Achyrocline satureioides* (Lam.) DC. | Asteraceae | S | N | N | Laxative | DDS |
| Garlet & Irgang 2001 | Pampa and Atlantic Forest | *Achyrocline satureioides* (Lam.) DC. | Asteraceae | S | N | N | Poor digestion | DDS |
| Garlet & Irgang 2001 | Pampa and Atlantic Forest | *Achyrocline satureioides* (Lam.) DC. | Asteraceae | S | N | N | Stomach problems | DDS |
| Garlet & Irgang 2001 | Pampa and Atlantic Forest | *Achyrocline satureioides* (Lam.) DC. | Asteraceae | S | N | N | Stomatitis | DDS |
| Garlet & Irgang 2001 | Pampa and Atlantic Forest | *Achyrocline satureioides* (Lam.) DC. | Asteraceae | S | N | N | To prevent belly fat | ENMD |
| Garlet & Irgang 2001 | Pampa and Atlantic Forest | *Achyrocline satureioides* (Lam.) DC. | Asteraceae | S | N | N | Wound | IPEC |
| Garlet & Irgang 2001 | Pampa and Atlantic Forest | *Adiantopsis chlorophylla* (Sw.) Fée | Pteridaceae | H | N | N | Joint pain | DMS |
| Garlet & Irgang 2001 | Pampa and Atlantic Forest | *Adiantopsis chlorophylla* (Sw.) Fée | Pteridaceae | H | N | N | Rheumatism | DMS |
| Garlet & Irgang 2001 | Pampa and Atlantic Forest | *Adiantum pseudotinctum* Hieron. | Pteridaceae | H | N | N | Cough | SSNEC |
| Garlet & Irgang 2001 | Pampa and Atlantic Forest | *Adiantum pseudotinctum* Hieron. | Pteridaceae | H | N | N | Influenza | DRS |
| Garlet & Irgang 2001 | Pampa and Atlantic Forest | *Allium ampeloprasum* L. | Alliaceae | H | E | E | Fever | SSNEC |
| Garlet & Irgang 2001 | Pampa and Atlantic Forest | *Allium ampeloprasum* L. | Alliaceae | H | E | E | Influenza | DRS |
| Garlet & Irgang 2001 | Pampa and Atlantic Forest | *Allium cepa* L. | Alliaceae | H | E | E | Nervousness | MBD |
| Garlet & Irgang 2001 | Pampa and Atlantic Forest | *Allium cepa* L. | Alliaceae | H | E | E | Osteoporosis | DMS |
| Garlet & Irgang 2001 | Pampa and Atlantic Forest | *Allium sativum* L. | Alliaceae | H | E | E | Infection | CIPD |
| Garlet & Irgang 2001 | Pampa and Atlantic Forest | *Allium sativum* L. | Alliaceae | H | E | E | Influenza | DRS |
| Garlet & Irgang 2001 | Pampa and Atlantic Forest | *Allium sativum* L. | Alliaceae | H | E | E | Spider bite | IPEC |
| Garlet & Irgang 2001 | Pampa and Atlantic Forest | *Aloe arborescens* Mill. | Xanthorrhoeaceae | S | E | E | Burns | IPEC |
| Garlet & Irgang 2001 | Pampa and Atlantic Forest | *Aloe arborescens* Mill. | Xanthorrhoeaceae | S | E | E | Cancer | NEO |
| Garlet & Irgang 2001 | Pampa and Atlantic Forest | *Aloe arborescens* Mill. | Xanthorrhoeaceae | S | E | E | Herpes labialis | CIPD |
| Garlet & Irgang 2001 | Pampa and Atlantic Forest | *Aloe arborescens* Mill. | Xanthorrhoeaceae | S | E | E | Liver problems | DDS |
| Garlet & Irgang 2001 | Pampa and Atlantic Forest | *Aloe arborescens* Mill. | Xanthorrhoeaceae | S | E | E | Ovarian infection | DGS |
| Garlet & Irgang 2001 | Pampa and Atlantic Forest | *Aloe arborescens* Mill. | Xanthorrhoeaceae | S | E | E | Prostate problems | DGS |
| Garlet & Irgang 2001 | Pampa and Atlantic Forest | *Aloe arborescens* Mill. | Xanthorrhoeaceae | S | E | E | Stomach problems | DDS |
| Garlet & Irgang 2001 | Pampa and Atlantic Forest | *Aloe arborescens* Mill. | Xanthorrhoeaceae | S | E | E | Undefined | SSNEC |
| Garlet & Irgang 2001 | Pampa and Atlantic Forest | *Aloe arborescens* Mill. | Xanthorrhoeaceae | S | E | E | Wound | IPEC |
| Garlet & Irgang 2001 | Pampa and Atlantic Forest | *Aloe vera* (L.) Burm.f. | Xanthorrhoeaceae | S | E | E | Nausea | SSNEC |
| Garlet & Irgang 2001 | Pampa and Atlantic Forest | *Aloe vera* (L.) Burm.f. | Xanthorrhoeaceae | S | E | E | Stomach pain/Diarrhea | SSNEC |
| Garlet & Irgang 2001 | Pampa and Atlantic Forest | *Aloysia citrioPaina Palau* | Verbenaceae | S | E | E | Calming | MBD |
| Garlet & Irgang 2001 | Pampa and Atlantic Forest | *Aloysia citrioPaina Palau* | Verbenaceae | S | E | E | Fever | SSNEC |
| Garlet & Irgang 2001 | Pampa and Atlantic Forest | *Aloysia citrioPaina Palau* | Verbenaceae | S | E | E | Hemorrhoids | DCS |
| Garlet & Irgang 2001 | Pampa and Atlantic Forest | *Aloysia citrioPaina Palau* | Verbenaceae | S | E | E | High blood pressure | DCS |
| Garlet & Irgang 2001 | Pampa and Atlantic Forest | *Aloysia gratissima* (Gillies & Hook. ex Hook.) Tronc. | Verbenaceae | S | N | N | Bronchitis | DRS |
| Garlet & Irgang 2001 | Pampa and Atlantic Forest | *Aloysia gratissima* (Gillies & Hook. ex Hook.) Tronc. | Verbenaceae | S | N | N | Fever | SSNEC |
| Garlet & Irgang 2001 | Pampa and Atlantic Forest | *Aloysia gratissima* (Gillies & Hook. ex Hook.) Tronc. | Verbenaceae | S | N | N | Headache | SSNEC |
| Garlet & Irgang 2001 | Pampa and Atlantic Forest | *Aloysia gratissima* (Gillies & Hook. ex Hook.) Tronc. | Verbenaceae | S | N | N | Helminthiasis | CIPD |
| Garlet & Irgang 2001 | Pampa and Atlantic Forest | *Aloysia gratissima* (Gillies & Hook. ex Hook.) Tronc. | Verbenaceae | S | N | N | Influenza | DRS |
| Garlet & Irgang 2001 | Pampa and Atlantic Forest | *Aloysia gratissima* (Gillies & Hook. ex Hook.) Tronc. | Verbenaceae | S | N | N | Pneumonia | DRS |
| Garlet & Irgang 2001 | Pampa and Atlantic Forest | *Aloysia gratissima* (Gillies & Hook. ex Hook.) Tronc. | Verbenaceae | S | N | N | Pulmonary pain | DRS |
| Garlet & Irgang 2001 | Pampa and Atlantic Forest | *Alternanthera brasiliana* (L.) Kuntze | Amaranthaceae | H | N | N | Antimicrobial | CIPD |
| Garlet & Irgang 2001 | Pampa and Atlantic Forest | *Alternanthera brasiliana* (L.) Kuntze | Amaranthaceae | H | N | N | Headache | SSNEC |
| Garlet & Irgang 2001 | Pampa and Atlantic Forest | *Alternanthera brasiliana* (L.) Kuntze | Amaranthaceae | H | N | N | Infection | CIPD |
| Garlet & Irgang 2001 | Pampa and Atlantic Forest | *Alternanthera brasiliana* (L.) Kuntze | Amaranthaceae | H | N | N | Pain | SSNEC |
| Garlet & Irgang 2001 | Pampa and Atlantic Forest | *Alternanthera brasiliana* (L.) Kuntze | Amaranthaceae | H | N | N | Stomach problems | DDS |
| Garlet & Irgang 2001 | Pampa and Atlantic Forest | *Alternanthera brasiliana* (L.) Kuntze | Amaranthaceae | H | N | N | Throat infection | DRS |
| Garlet & Irgang 2001 | Pampa and Atlantic Forest | *Alternanthera paronychioides* A.St.-Hil. | Amaranthaceae | H | N | N | Headache | SSNEC |
| Garlet & Irgang 2001 | Pampa and Atlantic Forest | *Alternanthera paronychioides* A.St.-Hil. | Amaranthaceae | H | N | N | Headache | SSNEC |
| Garlet & Irgang 2001 | Pampa and Atlantic Forest | *Alternanthera paronychioides* A.St.-Hil. | Amaranthaceae | H | N | N | Pain | SSNEC |
| Garlet & Irgang 2001 | Pampa and Atlantic Forest | *Alternanthera paronychioides* A.St.-Hil. | Amaranthaceae | H | N | N | Pain | SSNEC |
| Garlet & Irgang 2001 | Pampa and Atlantic Forest | *Anethum graveolens* L. | Apiaceae | H | E | E | Stomach problems | DDS |
| Garlet & Irgang 2001 | Pampa and Atlantic Forest | *Annona rugulosa* (Schltdl.) H.Rainer | Annonaceae | T | N | N | Kidney problems | DGS |
| Garlet & Irgang 2001 | Pampa and Atlantic Forest | *Annona rugulosa* (Schltdl.) H.Rainer | Annonaceae | T | N | N | Throat infection | DRS |
| Garlet & Irgang 2001 | Pampa and Atlantic Forest | *Arctium lappa* L. | Asteraceae | H | E | E | Infection | CIPD |
| Garlet & Irgang 2001 | Pampa and Atlantic Forest | *Arctium lappa* L. | Asteraceae | H | E | E | Infection | CIPD |
| Garlet & Irgang 2001 | Pampa and Atlantic Forest | *Arctium lappa* L. | Asteraceae | H | E | E | Inflammation of the ovaries | DGS |
| Garlet & Irgang 2001 | Pampa and Atlantic Forest | *Arctium lappa* L. | Asteraceae | H | E | E | Inflammation of the ovaries | DGS |
| Garlet & Irgang 2001 | Pampa and Atlantic Forest | *Arctium lappa* L. | Asteraceae | H | E | E | Mumps | CIPD |
| Garlet & Irgang 2001 | Pampa and Atlantic Forest | *Arctium lappa* L. | Asteraceae | H | E | E | Mumps | CIPD |
| Garlet & Irgang 2001 | Pampa and Atlantic Forest | *Arctium lappa* L. | Asteraceae | H | E | E | Throat problems | DRS |
| Garlet & Irgang 2001 | Pampa and Atlantic Forest | *Arctium lappa* L. | Asteraceae | H | E | E | Throat problems | DRS |
| Garlet & Irgang 2001 | Pampa and Atlantic Forest | *Aristida jubata* (Arechav.) Herter | Poaceae | H | N | N | Fever | SSNEC |
| Garlet & Irgang 2001 | Pampa and Atlantic Forest | *Aristolochia triangularis* Cham. & Schltdl. | Aristolochiaceae | S | N | N | Amenorrhea | DGS |
| Garlet & Irgang 2001 | Pampa and Atlantic Forest | *Aristolochia triangularis* Cham. & Schltdl. | Aristolochiaceae | S | N | N | Kidney problems | DGS |
| Garlet & Irgang 2001 | Pampa and Atlantic Forest | *Aristolochia triangularis* Cham. & Schltdl. | Aristolochiaceae | S | N | N | Stomach problems | DDS |
| Garlet & Irgang 2001 | Pampa and Atlantic Forest | *Artemisia absinthium* L. | Asteraceae | H | E | E | Colic | SSNEC |
| Garlet & Irgang 2001 | Pampa and Atlantic Forest | *Artemisia absinthium* L. | Asteraceae | H | E | E | Digestive problems | DDS |
| Garlet & Irgang 2001 | Pampa and Atlantic Forest | *Artemisia absinthium* L. | Asteraceae | H | E | E | Hangover | MBD |
| Garlet & Irgang 2001 | Pampa and Atlantic Forest | *Artemisia absinthium* L. | Asteraceae | H | E | E | Liver problems | DDS |
| Garlet & Irgang 2001 | Pampa and Atlantic Forest | *Artemisia absinthium* L. | Asteraceae | H | E | E | Poor digestion | DDS |
| Garlet & Irgang 2001 | Pampa and Atlantic Forest | *Artemisia absinthium* L. | Asteraceae | H | E | E | Stomach problems | DDS |
| Garlet & Irgang 2001 | Pampa and Atlantic Forest | *Artemisia alba* Turra | Asteraceae | S | E | E | Headache | SSNEC |
| Garlet & Irgang 2001 | Pampa and Atlantic Forest | *Artemisia alba* Turra | Asteraceae | S | E | E | Muscle pain | DMS |
| Garlet & Irgang 2001 | Pampa and Atlantic Forest | *Artemisia alba* Turra | Asteraceae | S | E | E | Poor digestion | DDS |
| Garlet & Irgang 2001 | Pampa and Atlantic Forest | *Artemisia alba* Turra | Asteraceae | S | E | E | Rheumatism | DMS |
| Garlet & Irgang 2001 | Pampa and Atlantic Forest | *Artemisia alba* Turra | Asteraceae | S | E | E | Sinusitis | DRS |
| Garlet & Irgang 2001 | Pampa and Atlantic Forest | *Artemisia alba* Turra | Asteraceae | S | E | E | Toothache | DDS |
| Garlet & Irgang 2001 | Pampa and Atlantic Forest | *Artemisia verlotorum* Lamotte | Asteraceae | H | E | E | Liver problems | DDS |
| Garlet & Irgang 2001 | Pampa and Atlantic Forest | *Artemisia verlotorum* Lamotte | Asteraceae | H | E | E | Stomach problems | DDS |
| Garlet & Irgang 2001 | Pampa and Atlantic Forest | *Asparagus setaceus* (Kunth) Jessop | Asparagaceae | H | E | E | Kidney problems | DGS |
| Garlet & Irgang 2001 | Pampa and Atlantic Forest | *Aspilia montevidensis* (Spreng.) Kuntze | Asteraceae | H | N | N | Diabetes | ENMD |
| Garlet & Irgang 2001 | Pampa and Atlantic Forest | *Baccharis anomala* DC. | Asteraceae | S | N | N | Infection | CIPD |
| Garlet & Irgang 2001 | Pampa and Atlantic Forest | *Baccharis anomala* DC. | Asteraceae | S | N | N | Laxative | DDS |
| Garlet & Irgang 2001 | Pampa and Atlantic Forest | *Baccharis anomala* DC. | Asteraceae | S | N | N | Wound | IPEC |
| Garlet & Irgang 2001 | Pampa and Atlantic Forest | *Baccharis articulata* (Lam.) Pers. | Asteraceae | S | N | N | Liver problems | DDS |
| Garlet & Irgang 2001 | Pampa and Atlantic Forest | *Baccharis articulata* (Lam.) Pers. | Asteraceae | S | N | N | Problems with spinal column | DMS |
| Garlet & Irgang 2001 | Pampa and Atlantic Forest | *Baccharis articulata* (Lam.) Pers. | Asteraceae | S | N | N | Stomach problems | DDS |
| Garlet & Irgang 2001 | Pampa and Atlantic Forest | *Baccharis crispa* Spreng. | Asteraceae | H | N | N | Liver problems | DDS |
| Garlet & Irgang 2001 | Pampa and Atlantic Forest | *Baccharis dracunculifolia* DC. | Asteraceae | S | N | N | Menstrual cramps | DGS |
| Garlet & Irgang 2001 | Pampa and Atlantic Forest | *Baccharis gaudichaudiana* DC. | Asteraceae | S | N | N | Diabetes | ENMD |
| Garlet & Irgang 2001 | Pampa and Atlantic Forest | *Baccharis gaudichaudiana* DC. | Asteraceae | S | N | N | Liver problems | DDS |
| Garlet & Irgang 2001 | Pampa and Atlantic Forest | *Baccharis gaudichaudiana* DC. | Asteraceae | S | N | N | Stomach problems | DDS |
| Garlet & Irgang 2001 | Pampa and Atlantic Forest | *Baccharis gaudichaudiana* DC. | Asteraceae | S | N | N | Weight reduction | ENMD |
| Garlet & Irgang 2001 | Pampa and Atlantic Forest | *Banara parviflora* (A.Gray) Benth. | Salicaceae | T | N | N | Ulcer | DDS |
| Garlet & Irgang 2001 | Pampa and Atlantic Forest | *Bauhinia forficata* Link | Fabaceae | T | N | N | Bladder infection | DGS |
| Garlet & Irgang 2001 | Pampa and Atlantic Forest | *Bauhinia forficata* Link | Fabaceae | T | N | N | Diabetes | ENMD |
| Garlet & Irgang 2001 | Pampa and Atlantic Forest | *Bauhinia forficata* Link | Fabaceae | T | N | N | Diuretic | SSNEC |
| Garlet & Irgang 2001 | Pampa and Atlantic Forest | *Bauhinia forficata* Link | Fabaceae | T | N | N | Menopause | ENMD |
| Garlet & Irgang 2001 | Pampa and Atlantic Forest | *Bauhinia forficata* Link | Fabaceae | T | N | N | Renal infection | DGS |
| Garlet & Irgang 2001 | Pampa and Atlantic Forest | *Bidens pilosa* L. | Asteraceae | H | E | N | Anemia | DBBO |
| Garlet & Irgang 2001 | Pampa and Atlantic Forest | *Bidens pilosa* L. | Asteraceae | H | E | N | Anemia | DBBO |
| Garlet & Irgang 2001 | Pampa and Atlantic Forest | *Bidens pilosa* L. | Asteraceae | H | E | N | Anemia | DBBO |
| Garlet & Irgang 2001 | Pampa and Atlantic Forest | *Bidens pilosa* L. | Asteraceae | H | E | N | Diabetes | ENMD |
| Garlet & Irgang 2001 | Pampa and Atlantic Forest | *Bidens pilosa* L. | Asteraceae | H | E | N | Diabetes | ENMD |
| Garlet & Irgang 2001 | Pampa and Atlantic Forest | *Bidens pilosa* L. | Asteraceae | H | E | N | Diabetes | ENMD |
| Garlet & Irgang 2001 | Pampa and Atlantic Forest | *Bidens pilosa* L. | Asteraceae | H | E | N | Inflammation of the kidney | DGS |
| Garlet & Irgang 2001 | Pampa and Atlantic Forest | *Bidens pilosa* L. | Asteraceae | H | E | N | Inflammation of the kidney | DGS |
| Garlet & Irgang 2001 | Pampa and Atlantic Forest | *Bidens pilosa* L. | Asteraceae | H | E | N | Inflammation of the kidney | DGS |
| Garlet & Irgang 2001 | Pampa and Atlantic Forest | *Bidens pilosa* L. | Asteraceae | H | E | N | Inflammation of the throat | DRS |
| Garlet & Irgang 2001 | Pampa and Atlantic Forest | *Bidens pilosa* L. | Asteraceae | H | E | N | Inflammation of the throat | DRS |
| Garlet & Irgang 2001 | Pampa and Atlantic Forest | *Bidens pilosa* L. | Asteraceae | H | E | N | Inflammation of the throat | DRS |
| Garlet & Irgang 2001 | Pampa and Atlantic Forest | *Bidens pilosa* L. | Asteraceae | H | E | N | Kidney stone | DGS |
| Garlet & Irgang 2001 | Pampa and Atlantic Forest | *Bidens pilosa* L. | Asteraceae | H | E | N | Kidney stone | DGS |
| Garlet & Irgang 2001 | Pampa and Atlantic Forest | *Bidens pilosa* L. | Asteraceae | H | E | N | Kidney stone | DGS |
| Garlet & Irgang 2001 | Pampa and Atlantic Forest | *Bidens pilosa* L. | Asteraceae | H | E | N | Wound | IPEC |
| Garlet & Irgang 2001 | Pampa and Atlantic Forest | *Bidens pilosa* L. | Asteraceae | H | E | N | Wound | IPEC |
| Garlet & Irgang 2001 | Pampa and Atlantic Forest | *Bidens pilosa* L. | Asteraceae | H | E | N | Wound | IPEC |
| Garlet & Irgang 2001 | Pampa and Atlantic Forest | *Borreria dasycephala* (Cham. & Schltdl.) Bacigalupo & E.L.Cabral | Rubiaceae | H | N | N | Stomach pain/Diarrhea | SSNEC |
| Garlet & Irgang 2001 | Pampa and Atlantic Forest | *Borreria verticillata* (L.) G.Mey. | Rubiaceae | H | N | N | Colic | SSNEC |
| Garlet & Irgang 2001 | Pampa and Atlantic Forest | *Borreria verticillata* (L.) G.Mey. | Rubiaceae | H | N | N | Stomach pain/Diarrhea | SSNEC |
| Garlet & Irgang 2001 | Pampa and Atlantic Forest | *Borreria verticillata* (L.) G.Mey. | Rubiaceae | H | N | N | Undefined | SSNEC |
| Garlet & Irgang 2001 | Pampa and Atlantic Forest | *Bromelia antiacantha* Bertol. | Bromeliaceae | H | N | N | Chest problems | SSNEC |
| Garlet & Irgang 2001 | Pampa and Atlantic Forest | *Calea serrata* Less. | Asteraceae | S | N | N | Liver problems | DDS |
| Garlet & Irgang 2001 | Pampa and Atlantic Forest | *Calendula officinalis* L. | Asteraceae | H | E | E | Cancer | NEO |
| Garlet & Irgang 2001 | Pampa and Atlantic Forest | *Calyptocarpus biaristatus* (DC.) H.Rob. | Asteraceae | H | E | E | Anemia | DBBO |
| Garlet & Irgang 2001 | Pampa and Atlantic Forest | *Calyptocarpus biaristatus* (DC.) H.Rob. | Asteraceae | H | E | E | Anemia | DBBO |
| Garlet & Irgang 2001 | Pampa and Atlantic Forest | *Calyptocarpus biaristatus* (DC.) H.Rob. | Asteraceae | H | E | E | Calming (for children) | MBD |
| Garlet & Irgang 2001 | Pampa and Atlantic Forest | *Calyptocarpus biaristatus* (DC.) H.Rob. | Asteraceae | H | E | E | Calming (for children) | MBD |
| Garlet & Irgang 2001 | Pampa and Atlantic Forest | *Campomanesia guazumifolia* (Cambess.) O.Berg | Myrtaceae | T | N | N | Chest problems | SSNEC |
| Garlet & Irgang 2001 | Pampa and Atlantic Forest | *Campomanesia guazumifolia* (Cambess.) O.Berg | Myrtaceae | T | N | N | Cough | SSNEC |
| Garlet & Irgang 2001 | Pampa and Atlantic Forest | *Campomanesia guazumifolia* (Cambess.) O.Berg | Myrtaceae | T | N | N | Fortifier | SSNEC |
| Garlet & Irgang 2001 | Pampa and Atlantic Forest | *Campomanesia guazumifolia* (Cambess.) O.Berg | Myrtaceae | T | N | N | Fortifier | SSNEC |
| Garlet & Irgang 2001 | Pampa and Atlantic Forest | *Campomanesia guazumifolia* (Cambess.) O.Berg | Myrtaceae | T | N | N | Inflammation of the bladder | DGS |
| Garlet & Irgang 2001 | Pampa and Atlantic Forest | *Campomanesia guazumifolia* (Cambess.) O.Berg | Myrtaceae | T | N | N | Inflammation of the kidney | DGS |
| Garlet & Irgang 2001 | Pampa and Atlantic Forest | *Campomanesia guazumifolia* (Cambess.) O.Berg | Myrtaceae | T | N | N | Influenza | DRS |
| Garlet & Irgang 2001 | Pampa and Atlantic Forest | *Campomanesia xanthocarpa* (Mart.) O.Berg | Myrtaceae | T | N | N | Eyewash | DEA |
| Garlet & Irgang 2001 | Pampa and Atlantic Forest | *Campomanesia xanthocarpa* (Mart.) O.Berg | Myrtaceae | T | N | N | High blood pressure | DCS |
| Garlet & Irgang 2001 | Pampa and Atlantic Forest | *Campomanesia xanthocarpa* (Mart.) O.Berg | Myrtaceae | T | N | N | High cholesterol | ENMD |
| Garlet & Irgang 2001 | Pampa and Atlantic Forest | *Campomanesia xanthocarpa* (Mart.) O.Berg | Myrtaceae | T | N | N | Weight reduction | ENMD |
| Garlet & Irgang 2001 | Pampa and Atlantic Forest | *Carica papaya* L. | Caricaceae | T | E | E | Cough | SSNEC |
| Garlet & Irgang 2001 | Pampa and Atlantic Forest | *Carya illinoinensis* (Wangenh.) K. Koch | Juglandaceae | T | E | E | Depurative | DBBO |
| Garlet & Irgang 2001 | Pampa and Atlantic Forest | *Carya illinoinensis* (Wangenh.) K. Koch | Juglandaceae | T | E | E | Diabetes | ENMD |
| Garlet & Irgang 2001 | Pampa and Atlantic Forest | *Carya illinoinensis* (Wangenh.) K. Koch | Juglandaceae | T | E | E | Expectorant | SSNEC |
| Garlet & Irgang 2001 | Pampa and Atlantic Forest | *Carya illinoinensis* (Wangenh.) K. Koch | Juglandaceae | T | E | E | High cholesterol | ENMD |
| Garlet & Irgang 2001 | Pampa and Atlantic Forest | *Carya illinoinensis* (Wangenh.) K. Koch | Juglandaceae | T | E | E | Influenza | DRS |
| Garlet & Irgang 2001 | Pampa and Atlantic Forest | *Casearia decandra* Jacq. | Salicaceae | T | N | N | Fortifier | SSNEC |
| Garlet & Irgang 2001 | Pampa and Atlantic Forest | *Casearia decandra* Jacq. | Salicaceae | T | N | N | Fortifier | SSNEC |
| Garlet & Irgang 2001 | Pampa and Atlantic Forest | *Casearia sylvestris* Sw. | Salicaceae | T | N | N | Snake bite | IPEC |
| Garlet & Irgang 2001 | Pampa and Atlantic Forest | *Chaptalia nutans* (L.) Pol. | Asteraceae | H | N | N | Liver problems | DDS |
| Garlet & Irgang 2001 | Pampa and Atlantic Forest | *Chelidonium majus* L. | Papaveraceae | H | E | E | Warts | CIPD |
| Garlet & Irgang 2001 | Pampa and Atlantic Forest | *Chenopodium ambrosioides* L. | Amaranthaceae | H | N | N | Anemia | DBBO |
| Garlet & Irgang 2001 | Pampa and Atlantic Forest | *Chenopodium ambrosioides* L. | Amaranthaceae | H | N | N | Helminthiasis | CIPD |
| Garlet & Irgang 2001 | Pampa and Atlantic Forest | *Chenopodium ambrosioides* L. | Amaranthaceae | H | N | N | Stomach pain/Diarrhea | SSNEC |
| Garlet & Irgang 2001 | Pampa and Atlantic Forest | *Cichorium intybus* L. | Asteraceae | H | E | E | Gastritis | DDS |
| Garlet & Irgang 2001 | Pampa and Atlantic Forest | *Citrus aurantiifolia* (Christm.) Swingle | Rutaceae | T | E | E | Calming | MBD |
| Garlet & Irgang 2001 | Pampa and Atlantic Forest | *Citrus aurantiifolia* (Christm.) Swingle | Rutaceae | T | E | E | Gastritis | DDS |
| Garlet & Irgang 2001 | Pampa and Atlantic Forest | *Citrus aurantiifolia* (Christm.) Swingle | Rutaceae | T | E | E | High blood pressure | DCS |
| Garlet & Irgang 2001 | Pampa and Atlantic Forest | *Citrus aurantiifolia* (Christm.) Swingle | Rutaceae | T | E | E | Vaginal discharge | DGS |
| Garlet & Irgang 2001 | Pampa and Atlantic Forest | *Citrus limon* (L.) Osbeck | Rutaceae | T | E | E | Cough | SSNEC |
| Garlet & Irgang 2001 | Pampa and Atlantic Forest | *Citrus limon* (L.) Osbeck | Rutaceae | T | E | E | Influenza | DRS |
| Garlet & Irgang 2001 | Pampa and Atlantic Forest | *Citrus limon* (L.) Osbeck | Rutaceae | T | E | E | Throat pain | DRS |
| Garlet & Irgang 2001 | Pampa and Atlantic Forest | *Citrus reticulata* Blanco | Rutaceae | T | E | E | Calming | MBD |
| Garlet & Irgang 2001 | Pampa and Atlantic Forest | *Citrus reticulata* Blanco | Rutaceae | T | E | E | Fever | SSNEC |
| Garlet & Irgang 2001 | Pampa and Atlantic Forest | *Citrus reticulata* Blanco | Rutaceae | T | E | E | Influenza | DRS |
| Garlet & Irgang 2001 | Pampa and Atlantic Forest | *Commelina erecta* L. | Commelinaceae | H | N | N | Infection | CIPD |
| Garlet & Irgang 2001 | Pampa and Atlantic Forest | *Commelina erecta* L. | Commelinaceae | H | N | N | Infection | CIPD |
| Garlet & Irgang 2001 | Pampa and Atlantic Forest | *Cordia americana* (L.) Gottschling & J.S.Mill. | Boraginaceae | T | N | N | Cough | SSNEC |
| Garlet & Irgang 2001 | Pampa and Atlantic Forest | *Cordia americana* (L.) Gottschling & J.S.Mill. | Boraginaceae | T | N | N | High cholesterol | ENMD |
| Garlet & Irgang 2001 | Pampa and Atlantic Forest | *Coronopus didymus* (L.) Sm. | Brassicaceae | H | N | N | Abortifacient | PCP |
| Garlet & Irgang 2001 | Pampa and Atlantic Forest | *Coronopus didymus* (L.) Sm. | Brassicaceae | H | N | N | Cough | SSNEC |
| Garlet & Irgang 2001 | Pampa and Atlantic Forest | *Coronopus didymus* (L.) Sm. | Brassicaceae | H | N | N | Expectorant | SSNEC |
| Garlet & Irgang 2001 | Pampa and Atlantic Forest | *Coronopus didymus* (L.) Sm. | Brassicaceae | H | N | N | Fracture | IPEC |
| Garlet & Irgang 2001 | Pampa and Atlantic Forest | *Coronopus didymus* (L.) Sm. | Brassicaceae | H | N | N | Influenza | DRS |
| Garlet & Irgang 2001 | Pampa and Atlantic Forest | *Coronopus didymus* (L.) Sm. | Brassicaceae | H | N | N | Injury | IPEC |
| Garlet & Irgang 2001 | Pampa and Atlantic Forest | *Coronopus didymus* (L.) Sm. | Brassicaceae | H | N | N | Internal wounds | IPEC |
| Garlet & Irgang 2001 | Pampa and Atlantic Forest | *Coronopus didymus* (L.) Sm. | Brassicaceae | H | N | N | Lung fortifier | DRS |
| Garlet & Irgang 2001 | Pampa and Atlantic Forest | *Croton didrichsenii* G.L.Webster | Euphorbiaceae | H | N | N | Problems with the blood | DBBO |
| Garlet & Irgang 2001 | Pampa and Atlantic Forest | *Cucurbita* *maxima* Duchesne ex Lam. | Cucurbitaceae | H | N | E | Earache | DEMP |
| Garlet & Irgang 2001 | Pampa and Atlantic Forest | *Cucurbita* *maxima* Duchesne ex Lam. | Cucurbitaceae | H | N | E | Helminthiasis | CIPD |
| Garlet & Irgang 2001 | Pampa and Atlantic Forest | *Cunila microcephala* Benth. | Lamiaceae | H | N | N | Chest problems | SSNEC |
| Garlet & Irgang 2001 | Pampa and Atlantic Forest | *Cunila microcephala* Benth. | Lamiaceae | H | N | N | Chest problems | SSNEC |
| Garlet & Irgang 2001 | Pampa and Atlantic Forest | *Cunila microcephala* Benth. | Lamiaceae | H | N | N | Cough | SSNEC |
| Garlet & Irgang 2001 | Pampa and Atlantic Forest | *Cunila microcephala* Benth. | Lamiaceae | H | N | N | Cough | SSNEC |
| Garlet & Irgang 2001 | Pampa and Atlantic Forest | *Cunila microcephala* Benth. | Lamiaceae | H | N | N | Infant colic | SSNEC |
| Garlet & Irgang 2001 | Pampa and Atlantic Forest | *Cunila microcephala* Benth. | Lamiaceae | H | N | N | Infant colic | SSNEC |
| Garlet & Irgang 2001 | Pampa and Atlantic Forest | *Cunila microcephala* Benth. | Lamiaceae | H | N | N | Influenza | DRS |
| Garlet & Irgang 2001 | Pampa and Atlantic Forest | *Cunila microcephala* Benth. | Lamiaceae | H | N | N | Influenza | DRS |
| Garlet & Irgang 2001 | Pampa and Atlantic Forest | *Cunila microcephala* Benth. | Lamiaceae | H | N | N | Stomach problems | DDS |
| Garlet & Irgang 2001 | Pampa and Atlantic Forest | *Cunila microcephala* Benth. | Lamiaceae | H | N | N | Stomach problems | DDS |
| Garlet & Irgang 2001 | Pampa and Atlantic Forest | *Cupania vernalis* Cambess | Sapindaceae | T | N | N | Bronchitis | DRS |
| Garlet & Irgang 2001 | Pampa and Atlantic Forest | *Cuphea ingrata* Cham. & Schltdl. | Lythraceae | S | N | N | Bladder problems | DGS |
| Garlet & Irgang 2001 | Pampa and Atlantic Forest | *Cuphea ingrata* Cham. & Schltdl. | Lythraceae | S | N | N | Depurative | DBBO |
| Garlet & Irgang 2001 | Pampa and Atlantic Forest | *Cuphea ingrata* Cham. & Schltdl. | Lythraceae | S | N | N | Diuretic | SSNEC |
| Garlet & Irgang 2001 | Pampa and Atlantic Forest | *Cuphea ingrata* Cham. & Schltdl. | Lythraceae | S | N | N | Kidney problems | DGS |
| Garlet & Irgang 2001 | Pampa and Atlantic Forest | *Cuphea ingrata* Cham. & Schltdl. | Lythraceae | S | N | N | Rejuvenation | SSNEC |
| Garlet & Irgang 2001 | Pampa and Atlantic Forest | *Cupressus arizonica* Greene | Cupressaceae | T | E | E | Sinusitis | DRS |
| Garlet & Irgang 2001 | Pampa and Atlantic Forest | *Cymbopogon citratus* (DC.) Stapf | Poaceae | H | E | E | Calming | MBD |
| Garlet & Irgang 2001 | Pampa and Atlantic Forest | *Cymbopogon citratus* (DC.) Stapf | Poaceae | H | E | E | Fever | SSNEC |
| Garlet & Irgang 2001 | Pampa and Atlantic Forest | *Cymbopogon citratus* (DC.) Stapf | Poaceae | H | E | E | High blood pressure | DCS |
| Garlet & Irgang 2001 | Pampa and Atlantic Forest | *Cymbopogon citratus* (DC.) Stapf | Poaceae | H | E | E | Influenza | DRS |
| Garlet & Irgang 2001 | Pampa and Atlantic Forest | *Cynara cardunculus* L. | Asteraceae | H | E | E | Digestive problems | DDS |
| Garlet & Irgang 2001 | Pampa and Atlantic Forest | *Cynara cardunculus* L. | Asteraceae | H | E | E | High blood pressure | DCS |
| Garlet & Irgang 2001 | Pampa and Atlantic Forest | *Cynara cardunculus* L. | Asteraceae | H | E | E | High cholesterol | ENMD |
| Garlet & Irgang 2001 | Pampa and Atlantic Forest | *Cynara cardunculus* L. | Asteraceae | H | E | E | Liver problems | DDS |
| Garlet & Irgang 2001 | Pampa and Atlantic Forest | *Cynara cardunculus* L. | Asteraceae | H | E | E | STD | CIPD |
| Garlet & Irgang 2001 | Pampa and Atlantic Forest | *Cynara cardunculus* L. | Asteraceae | H | E | E | Weight reduction | ENMD |
| Garlet & Irgang 2001 | Pampa and Atlantic Forest | *Desmodium incanum* DC | Fabaceae | H | N | N | Kidney problems | DGS |
| Garlet & Irgang 2001 | Pampa and Atlantic Forest | *Desmodium incanum* DC | Fabaceae | H | N | N | Liver problems | DDS |
| Garlet & Irgang 2001 | Pampa and Atlantic Forest | *Desmodium incanum* DC | Fabaceae | H | N | N | Problems with the blood | DBBO |
| Garlet & Irgang 2001 | Pampa and Atlantic Forest | *Desmodium incanum* DC | Fabaceae | H | N | N | Prostate problems | DGS |
| Garlet & Irgang 2001 | Pampa and Atlantic Forest | *Desmodium incanum* DC | Fabaceae | H | N | N | Urinary incontinence | DGS |
| Garlet & Irgang 2001 | Pampa and Atlantic Forest | *Dichondra sericea* Sw. | Convolvulaceae | H | N | N | Kidney problems | DGS |
| Garlet & Irgang 2001 | Pampa and Atlantic Forest | *Dichorisandra thyrsiflora* J.C.Mikan | Commelinaceae | H | N | N | Diuretic | SSNEC |
| Garlet & Irgang 2001 | Pampa and Atlantic Forest | *Dichorisandra thyrsiflora* J.C.Mikan | Commelinaceae | H | N | N | High blood pressure | DCS |
| Garlet & Irgang 2001 | Pampa and Atlantic Forest | *Diospyros kaki* Thunb. | Ebenaceae | T | E | E | Influenza | DRS |
| Garlet & Irgang 2001 | Pampa and Atlantic Forest | *EchinoPainus grandiflorus (Cham. & Schltr.) Micheli* | Alismataceae | H | N | N | Depurative | DBBO |
| Garlet & Irgang 2001 | Pampa and Atlantic Forest | *EchinoPainus grandiflorus (Cham. & Schltr.) Micheli* | Alismataceae | H | N | N | Diuretic | SSNEC |
| Garlet & Irgang 2001 | Pampa and Atlantic Forest | *EchinoPainus grandiflorus (Cham. & Schltr.) Micheli* | Alismataceae | H | N | N | Inflammation | SSNEC |
| Garlet & Irgang 2001 | Pampa and Atlantic Forest | *EchinoPainus grandiflorus (Cham. & Schltr.) Micheli* | Alismataceae | H | N | N | Liver problems | DDS |
| Garlet & Irgang 2001 | Pampa and Atlantic Forest | *EchinoPainus grandiflorus (Cham. & Schltr.) Micheli* | Alismataceae | H | N | N | Mucus in the bladder | DGS |
| Garlet & Irgang 2001 | Pampa and Atlantic Forest | *EchinoPainus grandiflorus (Cham. & Schltr.) Micheli* | Alismataceae | H | N | N | Rheumatism | DMS |
| Garlet & Irgang 2001 | Pampa and Atlantic Forest | *EchinoPainus grandiflorus (Cham. & Schltr.) Micheli* | Alismataceae | H | N | N | Skin cleanser | DSST |
| Garlet & Irgang 2001 | Pampa and Atlantic Forest | *EchinoPainus grandiflorus (Cham. & Schltr.) Micheli* | Alismataceae | H | N | N | Weight reduction | ENMD |
| Garlet & Irgang 2001 | Pampa and Atlantic Forest | *Elephantopus mollis* Kunth | Asteraceae | H | E | E | Chest problems | SSNEC |
| Garlet & Irgang 2001 | Pampa and Atlantic Forest | *Elephantopus mollis* Kunth | Asteraceae | H | E | E | Chest problems | SSNEC |
| Garlet & Irgang 2001 | Pampa and Atlantic Forest | *Elephantopus mollis* Kunth | Asteraceae | H | E | E | Colic | SSNEC |
| Garlet & Irgang 2001 | Pampa and Atlantic Forest | *Elephantopus mollis* Kunth | Asteraceae | H | E | E | Colic | SSNEC |
| Garlet & Irgang 2001 | Pampa and Atlantic Forest | *Elephantopus mollis* Kunth | Asteraceae | H | E | E | Colitis | DDS |
| Garlet & Irgang 2001 | Pampa and Atlantic Forest | *Elephantopus mollis* Kunth | Asteraceae | H | E | E | Colitis | DDS |
| Garlet & Irgang 2001 | Pampa and Atlantic Forest | *Elephantopus mollis* Kunth | Asteraceae | H | E | E | Menopause | ENMD |
| Garlet & Irgang 2001 | Pampa and Atlantic Forest | *Elephantopus mollis* Kunth | Asteraceae | H | E | E | Menopause | ENMD |
| Garlet & Irgang 2001 | Pampa and Atlantic Forest | *Eriobotrya japonica* (Thunb.) Lindl. | Rosaceae | T | E | E | Chest problems | SSNEC |
| Garlet & Irgang 2001 | Pampa and Atlantic Forest | *Eriobotrya japonica* (Thunb.) Lindl. | Rosaceae | T | E | E | Cough | SSNEC |
| Garlet & Irgang 2001 | Pampa and Atlantic Forest | *Eriobotrya japonica* (Thunb.) Lindl. | Rosaceae | T | E | E | High blood pressure | DCS |
| Garlet & Irgang 2001 | Pampa and Atlantic Forest | *Eriobotrya japonica* (Thunb.) Lindl. | Rosaceae | T | E | E | Influenza | DRS |
| Garlet & Irgang 2001 | Pampa and Atlantic Forest | *Eriobotrya japonica* (Thunb.) Lindl. | Rosaceae | T | E | E | Throat irritation | DDS |
| Garlet & Irgang 2001 | Pampa and Atlantic Forest | *Eryngium horridum* Malme | Apiaceae | H | N | N | Cough | SSNEC |
| Garlet & Irgang 2001 | Pampa and Atlantic Forest | *Erythrina crista-galli* L. | Fabaceae | T | N | N | Gastritis | DDS |
| Garlet & Irgang 2001 | Pampa and Atlantic Forest | *Erythrina crista-galli* L. | Fabaceae | T | N | N | Influenza | DRS |
| Garlet & Irgang 2001 | Pampa and Atlantic Forest | *Erythrina crista-galli* L. | Fabaceae | T | N | N | Problems with spinal column | DMS |
| Garlet & Irgang 2001 | Pampa and Atlantic Forest | *Erythrina crista-galli* L. | Fabaceae | T | N | N | Throat pain | DRS |
| Garlet & Irgang 2001 | Pampa and Atlantic Forest | *Erythrina crista-galli* L. | Fabaceae | T | N | N | Ulcer | DDS |
| Garlet & Irgang 2001 | Pampa and Atlantic Forest | *Eugenia involucrata* DC. | Myrtaceae | T | N | N | Cough | SSNEC |
| Garlet & Irgang 2001 | Pampa and Atlantic Forest | *Eugenia involucrata* DC. | Myrtaceae | T | N | N | Eyewash | DEA |
| Garlet & Irgang 2001 | Pampa and Atlantic Forest | *Eugenia uniflora* L. | Myrtaceae | T | N | N | Diabetes | ENMD |
| Garlet & Irgang 2001 | Pampa and Atlantic Forest | *Eugenia uniflora* L. | Myrtaceae | T | N | N | Fortifier | SSNEC |
| Garlet & Irgang 2001 | Pampa and Atlantic Forest | *Eugenia uniflora* L. | Myrtaceae | T | N | N | High cholesterol | ENMD |
| Garlet & Irgang 2001 | Pampa and Atlantic Forest | *Eugenia uniflora* L. | Myrtaceae | T | N | N | Menstrual cramps | DGS |
| Garlet & Irgang 2001 | Pampa and Atlantic Forest | *Eugenia uniflora* L. | Myrtaceae | T | N | N | Stomach pain/Diarrhea | SSNEC |
| Garlet & Irgang 2001 | Pampa and Atlantic Forest | *Eugenia uniflora* L. | Myrtaceae | T | N | N | Stomach problems | DDS |
| Garlet & Irgang 2001 | Pampa and Atlantic Forest | *Euphorbia prostrata* Aiton | Euphorbiaceae | H | N | N | Inflammation of the kidney | DGS |
| Garlet & Irgang 2001 | Pampa and Atlantic Forest | *Euphorbia prostrata* Aiton | Euphorbiaceae | H | N | N | Kidney stone | DGS |
| Garlet & Irgang 2001 | Pampa and Atlantic Forest | *Euphorbia prostrata* Aiton | Euphorbiaceae | H | N | N | Throat infection | DRS |
| Garlet & Irgang 2001 | Pampa and Atlantic Forest | *Euphorbia serpens* Kunth | Euphorbiaceae | H | N | N | Bladder problems | DGS |
| Garlet & Irgang 2001 | Pampa and Atlantic Forest | *Euphorbia serpens* Kunth | Euphorbiaceae | H | N | N | Diuretic | SSNEC |
| Garlet & Irgang 2001 | Pampa and Atlantic Forest | *Euphorbia serpens* Kunth | Euphorbiaceae | H | N | N | Inflammation of the kidney | DGS |
| Garlet & Irgang 2001 | Pampa and Atlantic Forest | *Euphorbia serpens* Kunth | Euphorbiaceae | H | N | N | Kidney stone | DGS |
| Garlet & Irgang 2001 | Pampa and Atlantic Forest | *Euphorbia tirucalli* L. | Euphorbiaceae | S | E | E | Bronchitis | DRS |
| Garlet & Irgang 2001 | Pampa and Atlantic Forest | *Euphorbia tirucalli* L. | Euphorbiaceae | S | E | E | Cancer | NEO |
| Garlet & Irgang 2001 | Pampa and Atlantic Forest | *Ficus carica* L. | Moraceae | S | E | E | Influenza | DRS |
| Garlet & Irgang 2001 | Pampa and Atlantic Forest | *Foeniculum vulgare* Mill. | Apiaceae | H | E | E | Breast milk production | PCP |
| Garlet & Irgang 2001 | Pampa and Atlantic Forest | *Foeniculum vulgare* Mill. | Apiaceae | H | E | E | Constipation | DDS |
| Garlet & Irgang 2001 | Pampa and Atlantic Forest | *Foeniculum vulgare* Mill. | Apiaceae | H | E | E | Influenza | DRS |
| Garlet & Irgang 2001 | Pampa and Atlantic Forest | *Fragaria vesca* L. | Rosaceae | H | E | E | Calming | MBD |
| Garlet & Irgang 2001 | Pampa and Atlantic Forest | *Fragaria vesca* L. | Rosaceae | H | E | E | Cough | SSNEC |
| Garlet & Irgang 2001 | Pampa and Atlantic Forest | *Fragaria vesca* L. | Rosaceae | H | E | E | Depurative | DBBO |
| Garlet & Irgang 2001 | Pampa and Atlantic Forest | *Fragaria vesca* L. | Rosaceae | H | E | E | Throat problems | DRS |
| Garlet & Irgang 2001 | Pampa and Atlantic Forest | *Gochnatia polymorpha (Less.) Cabrera* | Asteraceae | T | N | E | Bronchitis | DRS |
| Garlet & Irgang 2001 | Pampa and Atlantic Forest | *Gochnatia polymorpha (Less.) Cabrera* | Asteraceae | T | N | E | Bronchitis | DRS |
| Garlet & Irgang 2001 | Pampa and Atlantic Forest | *Gochnatia polymorpha (Less.) Cabrera* | Asteraceae | T | N | E | Cough | SSNEC |
| Garlet & Irgang 2001 | Pampa and Atlantic Forest | *Gochnatia polymorpha (Less.) Cabrera* | Asteraceae | T | N | E | Cough | SSNEC |
| Garlet & Irgang 2001 | Pampa and Atlantic Forest | *Gochnatia polymorpha (Less.) Cabrera* | Asteraceae | T | N | E | Influenza | DRS |
| Garlet & Irgang 2001 | Pampa and Atlantic Forest | *Gochnatia polymorpha (Less.) Cabrera* | Asteraceae | T | N | E | Influenza | DRS |
| Garlet & Irgang 2001 | Pampa and Atlantic Forest | *Gymnanthemum amygdalinum* (Delile) Sch.Bip. ex Walp. | Asteraceae | S | N | N | Headache | SSNEC |
| Garlet & Irgang 2001 | Pampa and Atlantic Forest | *Gymnanthemum amygdalinum* (Delile) Sch.Bip. ex Walp. | Asteraceae | S | N | N | Stomach problems | DDS |
| Garlet & Irgang 2001 | Pampa and Atlantic Forest | *Hypericum connatum* Lam. | Hypericaceae | H | N | N | Chest problems | SSNEC |
| Garlet & Irgang 2001 | Pampa and Atlantic Forest | *Hypericum connatum* Lam. | Hypericaceae | H | N | N | Cough | SSNEC |
| Garlet & Irgang 2001 | Pampa and Atlantic Forest | *Hypericum connatum* Lam. | Hypericaceae | H | N | N | Hemorrhoids | DCS |
| Garlet & Irgang 2001 | Pampa and Atlantic Forest | *Impatiens walleriana* Hook. f. | Balsaminaceae | H | E | E | Calming | MBD |
| Garlet & Irgang 2001 | Pampa and Atlantic Forest | *Ipomoea batatas* (L.) Lam. | Convolvulaceae | H | E | E | Erysipelas | CIPD |
| Garlet & Irgang 2001 | Pampa and Atlantic Forest | *Ipomoea batatas* (L.) Lam. | Convolvulaceae | H | E | E | Inflammation of the bladder | DGS |
| Garlet & Irgang 2001 | Pampa and Atlantic Forest | *Ipomoea batatas* (L.) Lam. | Convolvulaceae | H | E | E | Rejuvenation | SSNEC |
| Garlet & Irgang 2001 | Pampa and Atlantic Forest | *Ipomoea batatas* (L.) Lam. | Convolvulaceae | H | E | E | Wound | IPEC |
| Garlet & Irgang 2001 | Pampa and Atlantic Forest | *Iresine herbstii* Hook. | Amaranthaceae | H | E | E | Cancer | NEO |
| Garlet & Irgang 2001 | Pampa and Atlantic Forest | *Juncus capillaceus* Lam. | Juncaceae | H | N | N | Bladder problems | DGS |
| Garlet & Irgang 2001 | Pampa and Atlantic Forest | *Juncus capillaceus* Lam. | Juncaceae | H | N | N | Kidney problems | DGS |
| Garlet & Irgang 2001 | Pampa and Atlantic Forest | *Juncus capillaceus* Lam. | Juncaceae | H | N | N | Kidney stone | DGS |
| Garlet & Irgang 2001 | Pampa and Atlantic Forest | *Juncus capillaceus* Lam. | Juncaceae | H | N | N | Urinary problems | DGS |
| Garlet & Irgang 2001 | Pampa and Atlantic Forest | *Kalanchoe delagoensis* Eckl. & Zeyh. | Crassulaceae | H | E | E | Earache | DEMP |
| Garlet & Irgang 2001 | Pampa and Atlantic Forest | *Lactuca sativa* L. | Asteraceae | H | E | E | Calming | MBD |
| Garlet & Irgang 2001 | Pampa and Atlantic Forest | *Lactuca sativa* L. | Asteraceae | H | E | E | Influenza | DRS |
| Garlet & Irgang 2001 | Pampa and Atlantic Forest | *Laurus nobilis* L. | Lauraceae | T | E | E | Menopause | ENMD |
| Garlet & Irgang 2001 | Pampa and Atlantic Forest | *Laurus nobilis* L. | Lauraceae | T | E | E | Menstrual cramps | DGS |
| Garlet & Irgang 2001 | Pampa and Atlantic Forest | *Lavandula angustifolia* Mill. | Lamiaceae | H | E | E | Cold sore | DDS |
| Garlet & Irgang 2001 | Pampa and Atlantic Forest | *Lippia alba* (Mill.) N.E.Br. | Verbenaceae | S | N | N | Colds | DRS |
| Garlet & Irgang 2001 | Pampa and Atlantic Forest | *Lippia alba* (Mill.) N.E.Br. | Verbenaceae | S | N | N | Cough | SSNEC |
| Garlet & Irgang 2001 | Pampa and Atlantic Forest | *Lippia alba* (Mill.) N.E.Br. | Verbenaceae | S | N | N | Fever | SSNEC |
| Garlet & Irgang 2001 | Pampa and Atlantic Forest | *Lippia alba* (Mill.) N.E.Br. | Verbenaceae | S | N | N | Influenza | DRS |
| Garlet & Irgang 2001 | Pampa and Atlantic Forest | *Lippia alba* (Mill.) N.E.Br. | Verbenaceae | S | N | N | Menopause | ENMD |
| Garlet & Irgang 2001 | Pampa and Atlantic Forest | *Lippia alba* (Mill.) N.E.Br. | Verbenaceae | S | N | N | Rhinitis | DRS |
| Garlet & Irgang 2001 | Pampa and Atlantic Forest | *Lippia alba* (Mill.) N.E.Br. | Verbenaceae | S | N | N | Throat problems | DRS |
| Garlet & Irgang 2001 | Pampa and Atlantic Forest | *Luehea divaricata* Mart. & Zucc. | Malvaceae | T | N | N | Aids memory | SSNEC |
| Garlet & Irgang 2001 | Pampa and Atlantic Forest | *Luehea divaricata* Mart. & Zucc. | Malvaceae | T | N | N | Chest problems | SSNEC |
| Garlet & Irgang 2001 | Pampa and Atlantic Forest | *Luehea divaricata* Mart. & Zucc. | Malvaceae | T | N | N | Chest problems | SSNEC |
| Garlet & Irgang 2001 | Pampa and Atlantic Forest | *Luehea divaricata* Mart. & Zucc. | Malvaceae | T | N | N | Chest problems | SSNEC |
| Garlet & Irgang 2001 | Pampa and Atlantic Forest | *Luehea divaricata* Mart. & Zucc. | Malvaceae | T | N | N | Cough | SSNEC |
| Garlet & Irgang 2001 | Pampa and Atlantic Forest | *Luehea divaricata* Mart. & Zucc. | Malvaceae | T | N | N | Cough | SSNEC |
| Garlet & Irgang 2001 | Pampa and Atlantic Forest | *Luehea divaricata* Mart. & Zucc. | Malvaceae | T | N | N | Cough | SSNEC |
| Garlet & Irgang 2001 | Pampa and Atlantic Forest | *Malus domestica* Borkh. | Rosaceae | T | E | E | Breast milk production | PCP |
| Garlet & Irgang 2001 | Pampa and Atlantic Forest | *Malus domestica* Borkh. | Rosaceae | T | E | E | Diuretic | SSNEC |
| Garlet & Irgang 2001 | Pampa and Atlantic Forest | *Malus domestica* Borkh. | Rosaceae | T | E | E | Tonic | SSNEC |
| Garlet & Irgang 2001 | Pampa and Atlantic Forest | *Malva parviflora* L. | Malvaceae | H | E | E | Antimicrobial | CIPD |
| Garlet & Irgang 2001 | Pampa and Atlantic Forest | *Malva parviflora* L. | Malvaceae | H | E | E | Antimicrobial | CIPD |
| Garlet & Irgang 2001 | Pampa and Atlantic Forest | *Malva parviflora* L. | Malvaceae | H | E | E | Expectorant | SSNEC |
| Garlet & Irgang 2001 | Pampa and Atlantic Forest | *Malva parviflora* L. | Malvaceae | H | E | E | Expectorant | SSNEC |
| Garlet & Irgang 2001 | Pampa and Atlantic Forest | *Malva parviflora* L. | Malvaceae | H | E | E | Inflammation | SSNEC |
| Garlet & Irgang 2001 | Pampa and Atlantic Forest | *Malva parviflora* L. | Malvaceae | H | E | E | Inflammation | SSNEC |
| Garlet & Irgang 2001 | Pampa and Atlantic Forest | *Malva parviflora* L. | Malvaceae | H | E | E | Inflammation of the ovaries | DGS |
| Garlet & Irgang 2001 | Pampa and Atlantic Forest | *Malva parviflora* L. | Malvaceae | H | E | E | Inflammation of the ovaries | DGS |
| Garlet & Irgang 2001 | Pampa and Atlantic Forest | *Malva parviflora* L. | Malvaceae | H | E | E | Joint pain | DMS |
| Garlet & Irgang 2001 | Pampa and Atlantic Forest | *Malva parviflora* L. | Malvaceae | H | E | E | Joint pain | DMS |
| Garlet & Irgang 2001 | Pampa and Atlantic Forest | *Malva parviflora* L. | Malvaceae | H | E | E | Menstrual cramps | DGS |
| Garlet & Irgang 2001 | Pampa and Atlantic Forest | *Malva parviflora* L. | Malvaceae | H | E | E | Menstrual cramps | DGS |
| Garlet & Irgang 2001 | Pampa and Atlantic Forest | *Malva parviflora* L. | Malvaceae | H | E | E | Pain in the fingers | DMS |
| Garlet & Irgang 2001 | Pampa and Atlantic Forest | *Malva parviflora* L. | Malvaceae | H | E | E | Pain in the fingers | DMS |
| Garlet & Irgang 2001 | Pampa and Atlantic Forest | *Malva parviflora* L. | Malvaceae | H | E | E | Throat pain | DRS |
| Garlet & Irgang 2001 | Pampa and Atlantic Forest | *Malva parviflora* L. | Malvaceae | H | E | E | Throat pain | DRS |
| Garlet & Irgang 2001 | Pampa and Atlantic Forest | *Malva parviflora* L. | Malvaceae | H | E | E | Toothache | DDS |
| Garlet & Irgang 2001 | Pampa and Atlantic Forest | *Malva parviflora* L. | Malvaceae | H | E | E | Toothache | DDS |
| Garlet & Irgang 2001 | Pampa and Atlantic Forest | *Malva parviflora* L. | Malvaceae | H | E | E | Wound | IPEC |
| Garlet & Irgang 2001 | Pampa and Atlantic Forest | *Malva parviflora* L. | Malvaceae | H | E | E | Wound | IPEC |
| Garlet & Irgang 2001 | Pampa and Atlantic Forest | *Mandevilla longiflora* (Desf.) Pichon | Apocynaceae | H | N | N | Allergy | IPEC |
| Garlet & Irgang 2001 | Pampa and Atlantic Forest | *Mandevilla longiflora* (Desf.) Pichon | Apocynaceae | H | N | N | Wound | IPEC |
| Garlet & Irgang 2001 | Pampa and Atlantic Forest | *Matricaria chamomilla* L. | Asteraceae | H | E | E | Calming | MBD |
| Garlet & Irgang 2001 | Pampa and Atlantic Forest | *Matricaria chamomilla* L. | Asteraceae | H | E | E | Constipation | DDS |
| Garlet & Irgang 2001 | Pampa and Atlantic Forest | *Matricaria chamomilla* L. | Asteraceae | H | E | E | Infection | CIPD |
| Garlet & Irgang 2001 | Pampa and Atlantic Forest | *Matricaria chamomilla* L. | Asteraceae | H | E | E | Weak bladder | DGS |
| Garlet & Irgang 2001 | Pampa and Atlantic Forest | *Matricaria chamomilla* L. | Asteraceae | H | E | E | Wound | IPEC |
| Garlet & Irgang 2001 | Pampa and Atlantic Forest | *Maytenus ilicifolia* Mart. ex Reissek | Celastraceae | S | N | N | Depurative | DBBO |
| Garlet & Irgang 2001 | Pampa and Atlantic Forest | *Maytenus ilicifolia* Mart. ex Reissek | Celastraceae | S | N | N | External wounds | IPEC |
| Garlet & Irgang 2001 | Pampa and Atlantic Forest | *Maytenus ilicifolia* Mart. ex Reissek | Celastraceae | S | N | N | Inflammation of the kidney | DGS |
| Garlet & Irgang 2001 | Pampa and Atlantic Forest | *Maytenus ilicifolia* Mart. ex Reissek | Celastraceae | S | N | N | Stomach pain/Diarrhea | SSNEC |
| Garlet & Irgang 2001 | Pampa and Atlantic Forest | *Maytenus ilicifolia* Mart. ex Reissek | Celastraceae | S | N | N | Ulcer | DDS |
| Garlet & Irgang 2001 | Pampa and Atlantic Forest | *Melia azedarach* L | Meliaceae | T | E | E | Throat pain | DRS |
| Garlet & Irgang 2001 | Pampa and Atlantic Forest | *Melissa officinalis* L. | Lamiaceae | H | E | E | Calming | MBD |
| Garlet & Irgang 2001 | Pampa and Atlantic Forest | *Melissa officinalis* L. | Lamiaceae | H | E | E | High blood pressure | DCS |
| Garlet & Irgang 2001 | Pampa and Atlantic Forest | *Mentha × rotundifolia* (L.) Huds. | Lamiaceae | H | E | E | Calming | MBD |
| Garlet & Irgang 2001 | Pampa and Atlantic Forest | *Mentha × rotundifolia* (L.) Huds. | Lamiaceae | H | E | E | Cough | SSNEC |
| Garlet & Irgang 2001 | Pampa and Atlantic Forest | *Mentha × rotundifolia* (L.) Huds. | Lamiaceae | H | E | E | Helminthiasis | CIPD |
| Garlet & Irgang 2001 | Pampa and Atlantic Forest | *Mentha × rotundifolia* (L.) Huds. | Lamiaceae | H | E | E | Influenza | DRS |
| Garlet & Irgang 2001 | Pampa and Atlantic Forest | *Mentha × rotundifolia* (L.) Huds. | Lamiaceae | H | E | E | Weakness | SSNEC |
| Garlet & Irgang 2001 | Pampa and Atlantic Forest | *Microgramma squamulosa* (Kaulf.) de la Sota | Polypodiaceae | H | N | N | Diabetes | ENMD |
| Garlet & Irgang 2001 | Pampa and Atlantic Forest | *Microgramma squamulosa* (Kaulf.) de la Sota | Polypodiaceae | H | N | N | Kidney problems | DGS |
| Garlet & Irgang 2001 | Pampa and Atlantic Forest | *Mikania glomerata* Spreng. | Asteraceae | S | N | N | High blood pressure | DCS |
| Garlet & Irgang 2001 | Pampa and Atlantic Forest | *Mikania glomerata* Spreng. | Asteraceae | S | N | N | Phlegm | DRS |
| Garlet & Irgang 2001 | Pampa and Atlantic Forest | *Mikania laevigata* Sch.Bip. ex Baker | Asteraceae | S | N | N | Cough | SSNEC |
| Garlet & Irgang 2001 | Pampa and Atlantic Forest | *Mikania laevigata* Sch.Bip. ex Baker | Asteraceae | S | N | N | Pulmonary problems | DRS |
| Garlet & Irgang 2001 | Pampa and Atlantic Forest | *Mirabilis jalapa* L. | Nyctaginaceae | H | E | E | Earache | DEMP |
| Garlet & Irgang 2001 | Pampa and Atlantic Forest | *Muehlenbeckia sagittifolia* (Ortega) Meisn. | Polygonaceae | S | N | N | Allergy | IPEC |
| Garlet & Irgang 2001 | Pampa and Atlantic Forest | *Muehlenbeckia sagittifolia* (Ortega) Meisn. | Polygonaceae | S | N | N | Depurative | DBBO |
| Garlet & Irgang 2001 | Pampa and Atlantic Forest | *Muehlenbeckia sagittifolia* (Ortega) Meisn. | Polygonaceae | S | N | N | Detoxifying agent | SSNEC |
| Garlet & Irgang 2001 | Pampa and Atlantic Forest | *Muehlenbeckia sagittifolia* (Ortega) Meisn. | Polygonaceae | S | N | N | Diabetes | ENMD |
| Garlet & Irgang 2001 | Pampa and Atlantic Forest | *Muehlenbeckia sagittifolia* (Ortega) Meisn. | Polygonaceae | S | N | N | Hemorrhoids | DCS |
| Garlet & Irgang 2001 | Pampa and Atlantic Forest | *Muehlenbeckia sagittifolia* (Ortega) Meisn. | Polygonaceae | S | N | N | Scabies | CIPD |
| Garlet & Irgang 2001 | Pampa and Atlantic Forest | *Musa paradisiaca* L. | Musaceae | H | E | E | Bronchitis | DRS |
| Garlet & Irgang 2001 | Pampa and Atlantic Forest | *Musa paradisiaca* L. | Musaceae | H | E | E | Cardiac problems | DCS |
| Garlet & Irgang 2001 | Pampa and Atlantic Forest | *Nasturtium officinale* W. T. Aiton | Brassicaceae | H | E | E | Bronchitis | DRS |
| Garlet & Irgang 2001 | Pampa and Atlantic Forest | *Nasturtium officinale* W. T. Aiton | Brassicaceae | H | E | E | Cardiac problems | DCS |
| Garlet & Irgang 2001 | Pampa and Atlantic Forest | *Nasturtium officinale* W. T. Aiton | Brassicaceae | H | E | E | Cough | SSNEC |
| Garlet & Irgang 2001 | Pampa and Atlantic Forest | *Nasturtium officinale* W. T. Aiton | Brassicaceae | H | E | E | Hair loss | DSST |
| Garlet & Irgang 2001 | Pampa and Atlantic Forest | *Nasturtium officinale* W. T. Aiton | Brassicaceae | H | E | E | Influenza | DRS |
| Garlet & Irgang 2001 | Pampa and Atlantic Forest | *Nasturtium officinale* W. T. Aiton | Brassicaceae | H | E | E | Lung fortifier | DRS |
| Garlet & Irgang 2001 | Pampa and Atlantic Forest | *Nasturtium officinale* W. T. Aiton | Brassicaceae | H | E | E | Thyroid problems | ENMD |
| Garlet & Irgang 2001 | Pampa and Atlantic Forest | *Ocimum carnosum* (Spreng.) Link & Otto ex Benth. | Lamiaceae | H | N | N | Constipation | DDS |
| Garlet & Irgang 2001 | Pampa and Atlantic Forest | *Ocimum carnosum* (Spreng.) Link & Otto ex Benth. | Lamiaceae | H | N | N | Headache | SSNEC |
| Garlet & Irgang 2001 | Pampa and Atlantic Forest | *Ocimum carnosum* (Spreng.) Link & Otto ex Benth. | Lamiaceae | H | N | N | Improves digestion | DDS |
| Garlet & Irgang 2001 | Pampa and Atlantic Forest | *Ocimum carnosum* (Spreng.) Link & Otto ex Benth. | Lamiaceae | H | N | N | Menstrual cramps | DGS |
| Garlet & Irgang 2001 | Pampa and Atlantic Forest | *Ocimum carnosum* (Spreng.) Link & Otto ex Benth. | Lamiaceae | H | N | N | Rheumatism | DMS |
| Garlet & Irgang 2001 | Pampa and Atlantic Forest | *Ocimum carnosum* (Spreng.) Link & Otto ex Benth. | Lamiaceae | H | N | N | Stomach problems | DDS |
| Garlet & Irgang 2001 | Pampa and Atlantic Forest | *Odontocarya acuparata* Miers | Menispermaceae | S | N | N | Intoxication | IPEC |
| Garlet & Irgang 2001 | Pampa and Atlantic Forest | *Odontocarya acuparata* Miers | Menispermaceae | S | N | N | Liver problems | DDS |
| Garlet & Irgang 2001 | Pampa and Atlantic Forest | *Odontocarya acuparata* Miers | Menispermaceae | S | N | N | Stomach problems | DDS |
| Garlet & Irgang 2001 | Pampa and Atlantic Forest | *Origanum × majoricum* Cambess. | Lamiaceae | H | E | E | Bladder pain | SSNEC |
| Garlet & Irgang 2001 | Pampa and Atlantic Forest | *Origanum × majoricum* Cambess. | Lamiaceae | H | E | E | Bladder pain | SSNEC |
| Garlet & Irgang 2001 | Pampa and Atlantic Forest | *Origanum × majoricum* Cambess. | Lamiaceae | H | E | E | Calming | MBD |
| Garlet & Irgang 2001 | Pampa and Atlantic Forest | *Origanum × majoricum* Cambess. | Lamiaceae | H | E | E | Calming | MBD |
| Garlet & Irgang 2001 | Pampa and Atlantic Forest | *Origanum × majoricum* Cambess. | Lamiaceae | H | E | E | Chest problems | SSNEC |
| Garlet & Irgang 2001 | Pampa and Atlantic Forest | *Origanum × majoricum* Cambess. | Lamiaceae | H | E | E | Chest problems | SSNEC |
| Garlet & Irgang 2001 | Pampa and Atlantic Forest | *Origanum × majoricum* Cambess. | Lamiaceae | H | E | E | Cough | SSNEC |
| Garlet & Irgang 2001 | Pampa and Atlantic Forest | *Origanum × majoricum* Cambess. | Lamiaceae | H | E | E | Cough | SSNEC |
| Garlet & Irgang 2001 | Pampa and Atlantic Forest | *Origanum × majoricum* Cambess. | Lamiaceae | H | E | E | Earache | DEMP |
| Garlet & Irgang 2001 | Pampa and Atlantic Forest | *Origanum × majoricum* Cambess. | Lamiaceae | H | E | E | Earache | DEMP |
| Garlet & Irgang 2001 | Pampa and Atlantic Forest | *Origanum × majoricum* Cambess. | Lamiaceae | H | E | E | Infant colic | SSNEC |
| Garlet & Irgang 2001 | Pampa and Atlantic Forest | *Origanum × majoricum* Cambess. | Lamiaceae | H | E | E | Infant colic | SSNEC |
| Garlet & Irgang 2001 | Pampa and Atlantic Forest | *Origanum × majoricum* Cambess. | Lamiaceae | H | E | E | Stomach pain/Diarrhea | SSNEC |
| Garlet & Irgang 2001 | Pampa and Atlantic Forest | *Origanum × majoricum* Cambess. | Lamiaceae | H | E | E | Stomach pain/Diarrhea | SSNEC |
| Garlet & Irgang 2001 | Pampa and Atlantic Forest | *Parapiptadenia rigida* (Benth.) Brenan | Fabaceae | T | N | N | Bronchitis | DRS |
| Garlet & Irgang 2001 | Pampa and Atlantic Forest | *Parapiptadenia rigida* (Benth.) Brenan | Fabaceae | T | N | N | Cough | SSNEC |
| Garlet & Irgang 2001 | Pampa and Atlantic Forest | *Parapiptadenia rigida* (Benth.) Brenan | Fabaceae | T | N | N | Influenza | DRS |
| Garlet & Irgang 2001 | Pampa and Atlantic Forest | *Paspalum repens* P.J.Bergius | Poaceae | H | N | N | Anemia | DBBO |
| Garlet & Irgang 2001 | Pampa and Atlantic Forest | *Passiflora alata* Curtis | Passifloraceae | S | N | N | Calming | MBD |
| Garlet & Irgang 2001 | Pampa and Atlantic Forest | *Passiflora alata* Curtis | Passifloraceae | S | N | N | High blood pressure | DCS |
| Garlet & Irgang 2001 | Pampa and Atlantic Forest | *Passiflora edulis* Sims | Passifloraceae | S | N | N | Aphrodisiac | DGS |
| Garlet & Irgang 2001 | Pampa and Atlantic Forest | *Passiflora edulis* Sims | Passifloraceae | S | N | N | Calming | MBD |
| Garlet & Irgang 2001 | Pampa and Atlantic Forest | *Passiflora edulis* Sims | Passifloraceae | S | N | N | High blood pressure | DCS |
| Garlet & Irgang 2001 | Pampa and Atlantic Forest | *Passiflora edulis* Sims | Passifloraceae | S | N | N | Insomnia | DNS |
| Garlet & Irgang 2001 | Pampa and Atlantic Forest | *Persea americana* Mill. | Lauraceae | T | E | E | High blood pressure | DCS |
| Garlet & Irgang 2001 | Pampa and Atlantic Forest | *Petiveria alliacea* L. | Phytolaccaceae | H | E | E | Infection | CIPD |
| Garlet & Irgang 2001 | Pampa and Atlantic Forest | *Petiveria alliacea* L. | Phytolaccaceae | H | E | E | Toothache | DDS |
| Garlet & Irgang 2001 | Pampa and Atlantic Forest | *Petroselinum crispum* (Mill.) Fuss | Apiaceae | H | E | E | Aids memory | SSNEC |
| Garlet & Irgang 2001 | Pampa and Atlantic Forest | *Petroselinum crispum* (Mill.) Fuss | Apiaceae | H | E | E | Bladder problems | DGS |
| Garlet & Irgang 2001 | Pampa and Atlantic Forest | *Petroselinum crispum* (Mill.) Fuss | Apiaceae | H | E | E | Diuretic | SSNEC |
| Garlet & Irgang 2001 | Pampa and Atlantic Forest | *Petroselinum crispum* (Mill.) Fuss | Apiaceae | H | E | E | Hookworm infection | CIPD |
| Garlet & Irgang 2001 | Pampa and Atlantic Forest | *Phoradendron crassifolium* (Pohl ex DC.) Eichler | Santalaceae | S | N | N | Kidney problems | DGS |
| Garlet & Irgang 2001 | Pampa and Atlantic Forest | *Phyllanthus niruri* L. | Phyllanthaceae | H | N | N | Bladder problems | DGS |
| Garlet & Irgang 2001 | Pampa and Atlantic Forest | *Phyllanthus niruri* L. | Phyllanthaceae | H | N | N | Kidney problems | DGS |
| Garlet & Irgang 2001 | Pampa and Atlantic Forest | *Phyllanthus tenellus* Roxb. | Phyllanthaceae | H | N | N | Bladder problems | DGS |
| Garlet & Irgang 2001 | Pampa and Atlantic Forest | *Phyllanthus tenellus* Roxb. | Phyllanthaceae | H | N | N | Bladder problems | DGS |
| Garlet & Irgang 2001 | Pampa and Atlantic Forest | *Phyllanthus tenellus* Roxb. | Phyllanthaceae | H | N | N | Kidney problems | DGS |
| Garlet & Irgang 2001 | Pampa and Atlantic Forest | *Phyllanthus tenellus* Roxb. | Phyllanthaceae | H | N | N | Kidney problems | DGS |
| Garlet & Irgang 2001 | Pampa and Atlantic Forest | *Piper mikanianum* (Kunth) Steud. | Piperaceae | S | N | N | Abortifacient | PCP |
| Garlet & Irgang 2001 | Pampa and Atlantic Forest | *Piper mikanianum* (Kunth) Steud. | Piperaceae | S | N | N | Antimicrobial | CIPD |
| Garlet & Irgang 2001 | Pampa and Atlantic Forest | *Piper mikanianum* (Kunth) Steud. | Piperaceae | S | N | N | Infection | CIPD |
| Garlet & Irgang 2001 | Pampa and Atlantic Forest | *Piper mikanianum* (Kunth) Steud. | Piperaceae | S | N | N | Intestinal problems | DDS |
| Garlet & Irgang 2001 | Pampa and Atlantic Forest | *Piper mikanianum* (Kunth) Steud. | Piperaceae | S | N | N | Rheumatism | DMS |
| Garlet & Irgang 2001 | Pampa and Atlantic Forest | *Piptochaetium montevidense* (Spreng.) Parodi | Poaceae | H | N | N | Kidney problems | DGS |
| Garlet & Irgang 2001 | Pampa and Atlantic Forest | *Plantago australis* Lam. | Plantaginaceae | H | N | N | Constipation | DDS |
| Garlet & Irgang 2001 | Pampa and Atlantic Forest | *Plantago australis* Lam. | Plantaginaceae | H | N | N | Constipation | DDS |
| Garlet & Irgang 2001 | Pampa and Atlantic Forest | *Plantago australis* Lam. | Plantaginaceae | H | N | N | Dental care | DDS |
| Garlet & Irgang 2001 | Pampa and Atlantic Forest | *Plantago australis* Lam. | Plantaginaceae | H | N | N | Dental care | DDS |
| Garlet & Irgang 2001 | Pampa and Atlantic Forest | *Plantago australis* Lam. | Plantaginaceae | H | N | N | Hemorrhoids | DCS |
| Garlet & Irgang 2001 | Pampa and Atlantic Forest | *Plantago australis* Lam. | Plantaginaceae | H | N | N | Hemorrhoids | DCS |
| Garlet & Irgang 2001 | Pampa and Atlantic Forest | *Plantago australis* Lam. | Plantaginaceae | H | N | N | High blood pressure | DCS |
| Garlet & Irgang 2001 | Pampa and Atlantic Forest | *Plantago australis* Lam. | Plantaginaceae | H | N | N | High blood pressure | DCS |
| Garlet & Irgang 2001 | Pampa and Atlantic Forest | *Plantago australis* Lam. | Plantaginaceae | H | N | N | Inflammation | SSNEC |
| Garlet & Irgang 2001 | Pampa and Atlantic Forest | *Plantago australis* Lam. | Plantaginaceae | H | N | N | Inflammation | SSNEC |
| Garlet & Irgang 2001 | Pampa and Atlantic Forest | *Plantago australis* Lam. | Plantaginaceae | H | N | N | Inflammation of the ovaries | DGS |
| Garlet & Irgang 2001 | Pampa and Atlantic Forest | *Plantago australis* Lam. | Plantaginaceae | H | N | N | Inflammation of the ovaries | DGS |
| Garlet & Irgang 2001 | Pampa and Atlantic Forest | *Plantago australis* Lam. | Plantaginaceae | H | N | N | Problems with the uterus | DGS |
| Garlet & Irgang 2001 | Pampa and Atlantic Forest | *Plantago australis* Lam. | Plantaginaceae | H | N | N | Problems with the uterus | DGS |
| Garlet & Irgang 2001 | Pampa and Atlantic Forest | *Plantago australis* Lam. | Plantaginaceae | H | N | N | Wound | IPEC |
| Garlet & Irgang 2001 | Pampa and Atlantic Forest | *Plantago australis* Lam. | Plantaginaceae | H | N | N | Wound | IPEC |
| Garlet & Irgang 2001 | Pampa and Atlantic Forest | *Plantago major* L. | Plantaginaceae | H | E | E | Constipation | DDS |
| Garlet & Irgang 2001 | Pampa and Atlantic Forest | *Plantago major* L. | Plantaginaceae | H | E | E | Constipation | DDS |
| Garlet & Irgang 2001 | Pampa and Atlantic Forest | *Plantago major* L. | Plantaginaceae | H | E | E | Expectorant | SSNEC |
| Garlet & Irgang 2001 | Pampa and Atlantic Forest | *Plantago major* L. | Plantaginaceae | H | E | E | Expectorant | SSNEC |
| Garlet & Irgang 2001 | Pampa and Atlantic Forest | *Plantago major* L. | Plantaginaceae | H | E | E | Hemorrhoids | DCS |
| Garlet & Irgang 2001 | Pampa and Atlantic Forest | *Plantago major* L. | Plantaginaceae | H | E | E | Hemorrhoids | DCS |
| Garlet & Irgang 2001 | Pampa and Atlantic Forest | *Plantago major* L. | Plantaginaceae | H | E | E | Inflammation of the throat | DRS |
| Garlet & Irgang 2001 | Pampa and Atlantic Forest | *Plantago major* L. | Plantaginaceae | H | E | E | Inflammation of the throat | DRS |
| Garlet & Irgang 2001 | Pampa and Atlantic Forest | *Plantago major* L. | Plantaginaceae | H | E | E | Menstrual cramps | DGS |
| Garlet & Irgang 2001 | Pampa and Atlantic Forest | *Plantago major* L. | Plantaginaceae | H | E | E | Menstrual cramps | DGS |
| Garlet & Irgang 2001 | Pampa and Atlantic Forest | *Plantago major* L. | Plantaginaceae | H | E | E | Throat pain | DRS |
| Garlet & Irgang 2001 | Pampa and Atlantic Forest | *Plantago major* L. | Plantaginaceae | H | E | E | Throat pain | DRS |
| Garlet & Irgang 2001 | Pampa and Atlantic Forest | *Plantago major* L. | Plantaginaceae | H | E | E | Wound | IPEC |
| Garlet & Irgang 2001 | Pampa and Atlantic Forest | *Plantago major* L. | Plantaginaceae | H | E | E | Wound | IPEC |
| Garlet & Irgang 2001 | Pampa and Atlantic Forest | *Plectranthus barbatus* Andr. | Lamiaceae | H | E | E | Hangover | MBD |
| Garlet & Irgang 2001 | Pampa and Atlantic Forest | *Plectranthus barbatus* Andr. | Lamiaceae | H | E | E | High blood pressure | DCS |
| Garlet & Irgang 2001 | Pampa and Atlantic Forest | *Plectranthus barbatus* Andr. | Lamiaceae | H | E | E | Liver problems | DDS |
| Garlet & Irgang 2001 | Pampa and Atlantic Forest | *Plectranthus barbatus* Andr. | Lamiaceae | H | E | E | Nasal congestion | DRS |
| Garlet & Irgang 2001 | Pampa and Atlantic Forest | *Plectranthus barbatus* Andr. | Lamiaceae | H | E | E | Stomach problems | DDS |
| Garlet & Irgang 2001 | Pampa and Atlantic Forest | *Pluchea sagittalis* (Lam.) Cabrera | Asteraceae | H | N | N | Injury | IPEC |
| Garlet & Irgang 2001 | Pampa and Atlantic Forest | *Pluchea sagittalis* (Lam.) Cabrera | Asteraceae | H | N | N | Stomach pain/Diarrhea | SSNEC |
| Garlet & Irgang 2001 | Pampa and Atlantic Forest | *Polygonum punctatum* Elliott | Polygonaceae | H | N | N | Helminthiasis | CIPD |
| Garlet & Irgang 2001 | Pampa and Atlantic Forest | *Polygonum punctatum* Elliott | Polygonaceae | H | N | N | Helminthiasis | CIPD |
| Garlet & Irgang 2001 | Pampa and Atlantic Forest | *Polygonum punctatum* Elliott | Polygonaceae | H | N | N | Hemorrhoids | DCS |
| Garlet & Irgang 2001 | Pampa and Atlantic Forest | *Polygonum punctatum* Elliott | Polygonaceae | H | N | N | Hemorrhoids | DCS |
| Garlet & Irgang 2001 | Pampa and Atlantic Forest | *Polygonum punctatum* Elliott | Polygonaceae | H | N | N | Injury | IPEC |
| Garlet & Irgang 2001 | Pampa and Atlantic Forest | *Polygonum punctatum* Elliott | Polygonaceae | H | N | N | Injury | IPEC |
| Garlet & Irgang 2001 | Pampa and Atlantic Forest | *Polygonum punctatum* Elliott | Polygonaceae | H | N | N | Itchiness | DSST |
| Garlet & Irgang 2001 | Pampa and Atlantic Forest | *Polygonum punctatum* Elliott | Polygonaceae | H | N | N | Itchiness | DSST |
| Garlet & Irgang 2001 | Pampa and Atlantic Forest | *Polygonum punctatum* Elliott | Polygonaceae | H | N | N | Stomach pain/Diarrhea | SSNEC |
| Garlet & Irgang 2001 | Pampa and Atlantic Forest | *Polygonum punctatum* Elliott | Polygonaceae | H | N | N | Stomach pain/Diarrhea | SSNEC |
| Garlet & Irgang 2001 | Pampa and Atlantic Forest | *Porophyllum ruderale* (Jacq.) Cass. | Asteraceae | H | N | E | Inflammation of the ovaries | DGS |
| Garlet & Irgang 2001 | Pampa and Atlantic Forest | *Psidium grandifolium* Mart. ex DC. | Myrtaceae | S | N | N | Prostate problems | DGS |
| Garlet & Irgang 2001 | Pampa and Atlantic Forest | *Pteridium aquilinum* (L.) Kuhn | Dennstaedtiaceae | H | E | E | Problems with spinal column | DMS |
| Garlet & Irgang 2001 | Pampa and Atlantic Forest | *Pteridium aquilinum* (L.) Kuhn | Dennstaedtiaceae | H | E | E | Rheumatism | DMS |
| Garlet & Irgang 2001 | Pampa and Atlantic Forest | *Pterocaulon polystachyum* DC. | Asteraceae | H | N | N | Abortifacient | PCP |
| Garlet & Irgang 2001 | Pampa and Atlantic Forest | *Pterocaulon polystachyum* DC. | Asteraceae | H | N | N | Amenorrhea | DGS |
| Garlet & Irgang 2001 | Pampa and Atlantic Forest | *Pterocaulon polystachyum* DC. | Asteraceae | H | N | N | Liver problems | DDS |
| Garlet & Irgang 2001 | Pampa and Atlantic Forest | *Punica granatum* L. | Lythraceae | T | E | E | Stomach pain/Diarrhea | SSNEC |
| Garlet & Irgang 2001 | Pampa and Atlantic Forest | *Punica granatum* L. | Lythraceae | T | E | E | Stomach pain/Diarrhea | SSNEC |
| Garlet & Irgang 2001 | Pampa and Atlantic Forest | *Randia armata* (Sw.) DC. | Rubiaceae | S | N | N | Cough | SSNEC |
| Garlet & Irgang 2001 | Pampa and Atlantic Forest | *Randia armata* (Sw.) DC. | Rubiaceae | S | N | N | Problems with the blood | DBBO |
| Garlet & Irgang 2001 | Pampa and Atlantic Forest | *Rosmarinus officinalis* L. | Lamiaceae | S | E | E | Anemia | DBBO |
| Garlet & Irgang 2001 | Pampa and Atlantic Forest | *Rosmarinus officinalis* L. | Lamiaceae | S | E | E | Expectorant | SSNEC |
| Garlet & Irgang 2001 | Pampa and Atlantic Forest | *Rosmarinus officinalis* L. | Lamiaceae | S | E | E | Heart fortifier | DCS |
| Garlet & Irgang 2001 | Pampa and Atlantic Forest | *Rosmarinus officinalis* L. | Lamiaceae | S | E | E | Problems with the nervous system | DNS |
| Garlet & Irgang 2001 | Pampa and Atlantic Forest | *Rosmarinus officinalis* L. | Lamiaceae | S | E | E | Pulmonary problems | DRS |
| Garlet & Irgang 2001 | Pampa and Atlantic Forest | *Rosmarinus officinalis* L. | Lamiaceae | S | E | E | Stomach problems | DDS |
| Garlet & Irgang 2001 | Pampa and Atlantic Forest | *Rosmarinus officinalis* L. | Lamiaceae | S | E | E | Weak blood | DBBO |
| Garlet & Irgang 2001 | Pampa and Atlantic Forest | *Ruellia sanguinea* Griseb. | Acanthaceae | H | E | E | Depurative | DBBO |
| Garlet & Irgang 2001 | Pampa and Atlantic Forest | *Ruellia sanguinea* Griseb. | Acanthaceae | H | E | E | Depurative | DBBO |
| Garlet & Irgang 2001 | Pampa and Atlantic Forest | *Ruellia sanguinea* Griseb. | Acanthaceae | H | E | E | Rheumatism | DMS |
| Garlet & Irgang 2001 | Pampa and Atlantic Forest | *Ruellia sanguinea* Griseb. | Acanthaceae | H | E | E | Rheumatism | DMS |
| Garlet & Irgang 2001 | Pampa and Atlantic Forest | *Ruta chalepensis* L. | Rutaceae | H | E | E | Headache | SSNEC |
| Garlet & Irgang 2001 | Pampa and Atlantic Forest | *Ruta chalepensis* L. | Rutaceae | H | E | E | Helminthiasis | CIPD |
| Garlet & Irgang 2001 | Pampa and Atlantic Forest | *Ruta chalepensis* L. | Rutaceae | H | E | E | Pain | SSNEC |
| Garlet & Irgang 2001 | Pampa and Atlantic Forest | *Ruta graveolens* L. | Rutaceae | H | E | E | Amenorrhea | DGS |
| Garlet & Irgang 2001 | Pampa and Atlantic Forest | *Saccharum officinarum* L. | Poaceae | H | E | E | High blood pressure | DCS |
| Garlet & Irgang 2001 | Pampa and Atlantic Forest | *Salvia microphylla* Kunth | Lamiaceae | S | E | E | Fever | SSNEC |
| Garlet & Irgang 2001 | Pampa and Atlantic Forest | *Salvia microphylla* Kunth | Lamiaceae | S | E | E | Headache | SSNEC |
| Garlet & Irgang 2001 | Pampa and Atlantic Forest | *Salvia microphylla* Kunth | Lamiaceae | S | E | E | Influenza | DRS |
| Garlet & Irgang 2001 | Pampa and Atlantic Forest | *Salvia officinalis* L. | Lamiaceae | S | E | E | Bronchitis | DRS |
| Garlet & Irgang 2001 | Pampa and Atlantic Forest | *Salvia officinalis* L. | Lamiaceae | S | E | E | Stomach problems | DDS |
| Garlet & Irgang 2001 | Pampa and Atlantic Forest | *Salvia officinalis* L. | Lamiaceae | S | E | E | Thrush infection on the tongue | CIPD |
| Garlet & Irgang 2001 | Pampa and Atlantic Forest | *Sambucus australis* Cham. & Schltdl. | Adoxaceae | T | N | N | Bronchitis | DRS |
| Garlet & Irgang 2001 | Pampa and Atlantic Forest | *Sambucus australis* Cham. & Schltdl. | Adoxaceae | T | N | N | Bronchitis | DRS |
| Garlet & Irgang 2001 | Pampa and Atlantic Forest | *Sambucus australis* Cham. & Schltdl. | Adoxaceae | T | N | N | Diabetes | ENMD |
| Garlet & Irgang 2001 | Pampa and Atlantic Forest | *Sambucus australis* Cham. & Schltdl. | Adoxaceae | T | N | N | Diabetes | ENMD |
| Garlet & Irgang 2001 | Pampa and Atlantic Forest | *Sambucus australis* Cham. & Schltdl. | Adoxaceae | T | N | N | Fever | SSNEC |
| Garlet & Irgang 2001 | Pampa and Atlantic Forest | *Sambucus australis* Cham. & Schltdl. | Adoxaceae | T | N | N | Fever | SSNEC |
| Garlet & Irgang 2001 | Pampa and Atlantic Forest | *Sambucus australis* Cham. & Schltdl. | Adoxaceae | T | N | N | Influenza | DRS |
| Garlet & Irgang 2001 | Pampa and Atlantic Forest | *Sambucus australis* Cham. & Schltdl. | Adoxaceae | T | N | N | Influenza | DRS |
| Garlet & Irgang 2001 | Pampa and Atlantic Forest | *Sambucus australis* Cham. & Schltdl. | Adoxaceae | T | N | N | Measles | CIPD |
| Garlet & Irgang 2001 | Pampa and Atlantic Forest | *Sambucus australis* Cham. & Schltdl. | Adoxaceae | T | N | N | Measles | CIPD |
| Garlet & Irgang 2001 | Pampa and Atlantic Forest | *Sambucus australis* Cham. & Schltdl. | Adoxaceae | T | N | N | Undefined | SSNEC |
| Garlet & Irgang 2001 | Pampa and Atlantic Forest | *Sambucus australis* Cham. & Schltdl. | Adoxaceae | T | N | N | Undefined | SSNEC |
| Garlet & Irgang 2001 | Pampa and Atlantic Forest | *Scoparia dulcis* L. | Plantaginaceae | H | N | N | Colic | SSNEC |
| Garlet & Irgang 2001 | Pampa and Atlantic Forest | *Scoparia dulcis* L. | Plantaginaceae | H | N | N | Colic | SSNEC |
| Garlet & Irgang 2001 | Pampa and Atlantic Forest | *Scoparia dulcis* L. | Plantaginaceae | H | N | N | Inflammation of the bladder | DGS |
| Garlet & Irgang 2001 | Pampa and Atlantic Forest | *Scoparia dulcis* L. | Plantaginaceae | H | N | N | Inflammation of the bladder | DGS |
| Garlet & Irgang 2001 | Pampa and Atlantic Forest | *Scoparia dulcis* L. | Plantaginaceae | H | N | N | Inflammation of the kidney | DGS |
| Garlet & Irgang 2001 | Pampa and Atlantic Forest | *Scoparia dulcis* L. | Plantaginaceae | H | N | N | Inflammation of the kidney | DGS |
| Garlet & Irgang 2001 | Pampa and Atlantic Forest | *Scutia buxifolia* Reissek | Rhamnaceae | S | N | N | Diuretic | SSNEC |
| Garlet & Irgang 2001 | Pampa and Atlantic Forest | *Scutia buxifolia* Reissek | Rhamnaceae | S | N | N | Diuretic | SSNEC |
| Garlet & Irgang 2001 | Pampa and Atlantic Forest | *Scutia buxifolia* Reissek | Rhamnaceae | S | N | N | High blood pressure | DCS |
| Garlet & Irgang 2001 | Pampa and Atlantic Forest | *Scutia buxifolia* Reissek | Rhamnaceae | S | N | N | High blood pressure | DCS |
| Garlet & Irgang 2001 | Pampa and Atlantic Forest | *Sechium edule* (Jacq.) Sw. | Cucurbitaceae | H | E | E | High blood pressure | DCS |
| Garlet & Irgang 2001 | Pampa and Atlantic Forest | *Sedum dendroideum* DC. | Crassulaceae | H | E | E | Earache | DEMP |
| Garlet & Irgang 2001 | Pampa and Atlantic Forest | *Sedum dendroideum* DC. | Crassulaceae | H | E | E | Hemorrhoids | DCS |
| Garlet & Irgang 2001 | Pampa and Atlantic Forest | *Sida rhombifolia* L. | Malvaceae | H | N | N | High blood pressure | DCS |
| Garlet & Irgang 2001 | Pampa and Atlantic Forest | *Sida rhombifolia* L. | Malvaceae | H | N | N | High blood pressure | DCS |
| Garlet & Irgang 2001 | Pampa and Atlantic Forest | *Sida rhombifolia* L. | Malvaceae | H | N | N | Infection | CIPD |
| Garlet & Irgang 2001 | Pampa and Atlantic Forest | *Sida rhombifolia* L. | Malvaceae | H | N | N | Infection | CIPD |
| Garlet & Irgang 2001 | Pampa and Atlantic Forest | *Sida rhombifolia* L. | Malvaceae | H | N | N | Stomach pain/Diarrhea | SSNEC |
| Garlet & Irgang 2001 | Pampa and Atlantic Forest | *Sida rhombifolia* L. | Malvaceae | H | N | N | Stomach pain/Diarrhea | SSNEC |
| Garlet & Irgang 2001 | Pampa and Atlantic Forest | *Sida rhombifolia* L. | Malvaceae | H | N | N | Urinary Retention | DGS |
| Garlet & Irgang 2001 | Pampa and Atlantic Forest | *Sida rhombifolia* L. | Malvaceae | H | N | N | Urinary Retention | DGS |
| Garlet & Irgang 2001 | Pampa and Atlantic Forest | *Sida rhombifolia* L. | Malvaceae | H | N | N | Wound | IPEC |
| Garlet & Irgang 2001 | Pampa and Atlantic Forest | *Sida rhombifolia* L. | Malvaceae | H | N | N | Wound | IPEC |
| Garlet & Irgang 2001 | Pampa and Atlantic Forest | *Sida spinosa* L. | Malvaceae | H | N | N | Reduce triglycerides | ENMD |
| Garlet & Irgang 2001 | Pampa and Atlantic Forest | *Sida tuberculata* R.E.Fr. | Malvaceae | H | N | N | Reduce triglycerides | ENMD |
| Garlet & Irgang 2001 | Pampa and Atlantic Forest | *Sisyrinchium vaginatum* Spreng. | Iridaceae | H | N | N | Bladder problems | DGS |
| Garlet & Irgang 2001 | Pampa and Atlantic Forest | *Sisyrinchium vaginatum* Spreng. | Iridaceae | H | N | N | Kidney problems | DGS |
| Garlet & Irgang 2001 | Pampa and Atlantic Forest | *Sisyrinchium vaginatum* Spreng. | Iridaceae | H | N | N | Urinary problems | DGS |
| Garlet & Irgang 2001 | Pampa and Atlantic Forest | *Smilax campestris* Griseb. | Smilacaceae | S | N | N | Colic | SSNEC |
| Garlet & Irgang 2001 | Pampa and Atlantic Forest | *Smilax campestris* Griseb. | Smilacaceae | S | N | N | Stomach pain/Diarrhea | SSNEC |
| Garlet & Irgang 2001 | Pampa and Atlantic Forest | *Smilax cognata* Kunth | Smilacaceae | S | N | N | Stomach pain/Diarrhea | SSNEC |
| Garlet & Irgang 2001 | Pampa and Atlantic Forest | *Solanum guaraniticum* A.St.-Hil. | Solanaceae | S | N | N | Kidney problems | DGS |
| Garlet & Irgang 2001 | Pampa and Atlantic Forest | *Solanum mauritianum* Scop. | Solanaceae | S | N | N | Antimicrobial | CIPD |
| Garlet & Irgang 2001 | Pampa and Atlantic Forest | *Solanum mauritianum* Scop. | Solanaceae | S | N | N | Antimicrobial | CIPD |
| Garlet & Irgang 2001 | Pampa and Atlantic Forest | *Solanum mauritianum* Scop. | Solanaceae | S | N | N | Expectorant | SSNEC |
| Garlet & Irgang 2001 | Pampa and Atlantic Forest | *Solanum mauritianum* Scop. | Solanaceae | S | N | N | Expectorant | SSNEC |
| Garlet & Irgang 2001 | Pampa and Atlantic Forest | *Solanum mauritianum* Scop. | Solanaceae | S | N | N | Fever | SSNEC |
| Garlet & Irgang 2001 | Pampa and Atlantic Forest | *Solanum mauritianum* Scop. | Solanaceae | S | N | N | Fever | SSNEC |
| Garlet & Irgang 2001 | Pampa and Atlantic Forest | *Solanum mauritianum* Scop. | Solanaceae | S | N | N | Influenza | DRS |
| Garlet & Irgang 2001 | Pampa and Atlantic Forest | *Solanum mauritianum* Scop. | Solanaceae | S | N | N | Influenza | DRS |
| Garlet & Irgang 2001 | Pampa and Atlantic Forest | *Solanum mauritianum* Scop. | Solanaceae | S | N | N | Pulmonary pain | DRS |
| Garlet & Irgang 2001 | Pampa and Atlantic Forest | *Solanum mauritianum* Scop. | Solanaceae | S | N | N | Pulmonary pain | DRS |
| Garlet & Irgang 2001 | Pampa and Atlantic Forest | *Solanum paniculatum* L. | Solanaceae | S | N | N | Flatulence | SSNEC |
| Garlet & Irgang 2001 | Pampa and Atlantic Forest | *Solanum paniculatum* L. | Solanaceae | S | N | N | Intestinal cleansing | DDS |
| Garlet & Irgang 2001 | Pampa and Atlantic Forest | *Solanum granulosoleprosum* Dunal | Solanaceae | S | N | N | Influenza | DRS |
| Garlet & Irgang 2001 | Pampa and Atlantic Forest | *Sphagneticola trilobata* (L.) Pruski | Asteraceae | H | N | N | Diabetes | ENMD |
| Garlet & Irgang 2001 | Pampa and Atlantic Forest | *Stachys germanica* L. | Lamiaceae | H | E | E | Expectorant | SSNEC |
| Garlet & Irgang 2001 | Pampa and Atlantic Forest | *Stachys germanica* L. | Lamiaceae | H | E | E | Inflammation | SSNEC |
| Garlet & Irgang 2001 | Pampa and Atlantic Forest | *Stachys germanica* L. | Lamiaceae | H | E | E | Pulmonary problems | DRS |
| Garlet & Irgang 2001 | Pampa and Atlantic Forest | *Stachytarpheta cayennensis* (Rich.) Vahl | Verbenaceae | S | N | N | Gall stone | DDS |
| Garlet & Irgang 2001 | Pampa and Atlantic Forest | *Stachytarpheta cayennensis* (Rich.) Vahl | Verbenaceae | S | N | N | High cholesterol | ENMD |
| Garlet & Irgang 2001 | Pampa and Atlantic Forest | *Stachytarpheta cayennensis* (Rich.) Vahl | Verbenaceae | S | N | N | Improves digestion | DDS |
| Garlet & Irgang 2001 | Pampa and Atlantic Forest | *Stachytarpheta cayennensis* (Rich.) Vahl | Verbenaceae | S | N | N | Stomach problems | DDS |
| Garlet & Irgang 2001 | Pampa and Atlantic Forest | *Stenachaenium campestre* Baker | Asteraceae | H | N | N | Depurative | DBBO |
| Garlet & Irgang 2001 | Pampa and Atlantic Forest | *Syagrus romanzoffiana* (Cham.) Glassman | Arecaceae | T | N | N | Expectorant | SSNEC |
| Garlet & Irgang 2001 | Pampa and Atlantic Forest | *Syagrus romanzoffiana* (Cham.) Glassman | Arecaceae | T | N | N | Sinusitis | DRS |
| Garlet & Irgang 2001 | Pampa and Atlantic Forest | *Symphyotrichum squamatum* (Spreng.) G.L.Nesom | Asteraceae | H | N | N | Cancer | NEO |
| Garlet & Irgang 2001 | Pampa and Atlantic Forest | *Symphytum* officinale L. | Boraginaceae | H | E | E | Wound | IPEC |
| Garlet & Irgang 2001 | Pampa and Atlantic Forest | *Symphytum* officinale L. | Boraginaceae | H | E | E | Wound | IPEC |
| Garlet & Irgang 2001 | Pampa and Atlantic Forest | *Symplocos uniflora* (Pohl) Benth. | Symplocaceae | T | N | N | Allergy | IPEC |
| Garlet & Irgang 2001 | Pampa and Atlantic Forest | *Symplocos uniflora* (Pohl) Benth. | Symplocaceae | T | N | N | High cholesterol | ENMD |
| Garlet & Irgang 2001 | Pampa and Atlantic Forest | *Symplocos uniflora* (Pohl) Benth. | Symplocaceae | T | N | N | Itchiness | DSST |
| Garlet & Irgang 2001 | Pampa and Atlantic Forest | *Symplocos uniflora* (Pohl) Benth. | Symplocaceae | T | N | N | Rheumatism | DMS |
| Garlet & Irgang 2001 | Pampa and Atlantic Forest | *Syzygium cumini* (L.) Skeels | Myrtaceae | T | E | E | Diabetes | ENMD |
| Garlet & Irgang 2001 | Pampa and Atlantic Forest | *Tabernaemontana catharinensis* A.DC. | Apocynaceae | T | N | N | Headache | SSNEC |
| Garlet & Irgang 2001 | Pampa and Atlantic Forest | *Tabernaemontana catharinensis* A.DC. | Apocynaceae | T | N | N | Headache | SSNEC |
| Garlet & Irgang 2001 | Pampa and Atlantic Forest | *Tabernaemontana catharinensis* A.DC. | Apocynaceae | T | N | N | Insect bite | IPEC |
| Garlet & Irgang 2001 | Pampa and Atlantic Forest | *Tabernaemontana catharinensis* A.DC. | Apocynaceae | T | N | N | Poisoning by pesticides | IPEC |
| Garlet & Irgang 2001 | Pampa and Atlantic Forest | *Tabernaemontana catharinensis* A.DC. | Apocynaceae | T | N | N | Snake bite | IPEC |
| Garlet & Irgang 2001 | Pampa and Atlantic Forest | *Tanacetum parthenium* (L.) Sch. Bip. | Asteraceae | H | E | E | Abortifacient | PCP |
| Garlet & Irgang 2001 | Pampa and Atlantic Forest | *Tanacetum parthenium* (L.) Sch. Bip. | Asteraceae | H | E | E | Menstrual cramps | DGS |
| Garlet & Irgang 2001 | Pampa and Atlantic Forest | *Tanacetum vulgare* L. | Asteraceae | H | E | E | Amenorrhea | DGS |
| Garlet & Irgang 2001 | Pampa and Atlantic Forest | *Tanacetum vulgare* L. | Asteraceae | H | E | E | Diabetes | ENMD |
| Garlet & Irgang 2001 | Pampa and Atlantic Forest | *Tanacetum vulgare* L. | Asteraceae | H | E | E | Hangover | MBD |
| Garlet & Irgang 2001 | Pampa and Atlantic Forest | *Tanacetum vulgare* L. | Asteraceae | H | E | E | Pain | SSNEC |
| Garlet & Irgang 2001 | Pampa and Atlantic Forest | *Taraxacum officinale* F. H. Wigg. | Asteraceae | H | E | E | Diabetes | ENMD |
| Garlet & Irgang 2001 | Pampa and Atlantic Forest | *Tradescantia zebrina* Bosse | *Commelinaceae* | H | E | E | Kidney pain | DGS |
| Garlet & Irgang 2001 | Pampa and Atlantic Forest | *Tradescantia zebrina* Bosse | *Commelinaceae* | H | E | E | Kidney stone | DGS |
| Garlet & Irgang 2001 | Pampa and Atlantic Forest | *Tradescantia zebrina* Bosse | *Commelinaceae* | H | E | E | Skin blemishes | DSST |
| Garlet & Irgang 2001 | Pampa and Atlantic Forest | *Tradescantia zebrina* Bosse | *Commelinaceae* | H | E | E | Vitiligo | DSST |
| Garlet & Irgang 2001 | Pampa and Atlantic Forest | *Trichocline catharinensis* Cabrera | Asteraceae | H | N | N | Wound | IPEC |
| Garlet & Irgang 2001 | Pampa and Atlantic Forest | *Urera baccifera* (L.) Gaudich. ex Wedd. | Urticaceae | S | N | N | Chilblains | CIPD |
| Garlet & Irgang 2001 | Pampa and Atlantic Forest | *Urera baccifera* (L.) Gaudich. ex Wedd. | Urticaceae | S | N | N | Diuretic | SSNEC |
| Garlet & Irgang 2001 | Pampa and Atlantic Forest | *Urera baccifera* (L.) Gaudich. ex Wedd. | Urticaceae | S | N | N | Kidney stone | DGS |
| Garlet & Irgang 2001 | Pampa and Atlantic Forest | *Urera baccifera* (L.) Gaudich. ex Wedd. | Urticaceae | S | N | N | Wound | IPEC |
| Garlet & Irgang 2001 | Pampa and Atlantic Forest | *Verbena litoralis* Kunth | Verbenaceae | H | N | N | Bladder problems | DGS |
| Garlet & Irgang 2001 | Pampa and Atlantic Forest | *Verbena litoralis* Kunth | Verbenaceae | H | N | N | Kidney problems | DGS |
| Garlet & Irgang 2001 | Pampa and Atlantic Forest | *Verbena litoralis* Kunth | Verbenaceae | H | N | N | Problems with spinal column | DMS |
| Garlet & Irgang 2001 | Pampa and Atlantic Forest | *Verbena litoralis* Kunth | Verbenaceae | H | N | N | Stomach pain/Diarrhea | SSNEC |
| Garlet & Irgang 2001 | Pampa and Atlantic Forest | *Verbena litoralis* Kunth | Verbenaceae | H | N | N | Stomach problems | DDS |
| Garlet & Irgang 2001 | Pampa and Atlantic Forest | *Vernonanthura tweediana* (Baker) H.Rob. | Asteraceae | S | N | N | Chest problems | SSNEC |
| Garlet & Irgang 2001 | Pampa and Atlantic Forest | *Vernonanthura tweediana* (Baker) H.Rob. | Asteraceae | S | N | N | Cough | SSNEC |
| Garlet & Irgang 2001 | Pampa and Atlantic Forest | *Viola oPainata L.* | Violaceae | H | E | E | Bronchitis | DRS |
| Garlet & Irgang 2001 | Pampa and Atlantic Forest | *Viola oPainata L.* | Violaceae | H | E | E | Chest problems | SSNEC |
| Garlet & Irgang 2001 | Pampa and Atlantic Forest | *Viola oPainata L.* | Violaceae | H | E | E | Inflammation of the throat | DRS |
| Garlet & Irgang 2001 | Pampa and Atlantic Forest | *Viola oPainata L.* | Violaceae | H | E | E | Influenza | DRS |
| Garlet & Irgang 2001 | Pampa and Atlantic Forest | *Viola oPainata L.* | Violaceae | H | E | E | Malaise | SSNEC |
| Garlet & Irgang 2001 | Pampa and Atlantic Forest | *Viola oPainata L.* | Violaceae | H | E | E | Pain | SSNEC |
| Garlet & Irgang 2001 | Pampa and Atlantic Forest | *Visnaga daucoides* Gaertn. | Apiaceae | H | E | E | Constipation | DDS |
| Garlet & Irgang 2001 | Pampa and Atlantic Forest | *Visnaga daucoides* Gaertn. | Apiaceae | H | E | E | Fever | SSNEC |
| Garlet & Irgang 2001 | Pampa and Atlantic Forest | *Visnaga daucoides* Gaertn. | Apiaceae | H | E | E | Flatulence | SSNEC |
| Garlet & Irgang 2001 | Pampa and Atlantic Forest | *Visnaga daucoides* Gaertn. | Apiaceae | H | E | E | Kidney problems | DGS |
| Garlet & Irgang 2001 | Pampa and Atlantic Forest | *Vitex megapotamica* (Spreng.) Moldenke | Lamiaceae | T | N | N | High cholesterol | ENMD |
| Garlet & Irgang 2001 | Pampa and Atlantic Forest | *Vitis vinifera* L. | Vitaceae | S | E | E | Menopause | ENMD |
| Garlet & Irgang 2001 | Pampa and Atlantic Forest | *Waltheria americana* L. | Malvaceae | H | N | N | Depurative | DBBO |
| Garlet & Irgang 2001 | Pampa and Atlantic Forest | *Waltheria americana* L. | Malvaceae | H | N | N | Depurative | DBBO |
| Garlet & Irgang 2001 | Pampa and Atlantic Forest | *Waltheria communis* A.St.-Hil. | Malvaceae | H | N | N | Depurative | DBBO |
| Garlet & Irgang 2001 | Pampa and Atlantic Forest | *Waltheria communis* A.St.-Hil. | Malvaceae | H | N | N | High blood pressure | DCS |
| Garlet & Irgang 2001 | Pampa and Atlantic Forest | *Xanthium strumarium* L. | Asteraceae | H | E | E | Abortifacient | PCP |
| Garlet & Irgang 2001 | Pampa and Atlantic Forest | *Xanthium strumarium* L. | Asteraceae | H | E | E | Rheumatism | DMS |
| Garlet & Irgang 2001 | Pampa and Atlantic Forest | *Zea mays* L. | Poaceae | H | E | E | Bladder problems | DGS |
| Garlet & Irgang 2001 | Pampa and Atlantic Forest | *Zinnia elegans* Jacq. | Asteraceae | H | E | E | Menstrual cramps | DGS |
| Hanazaki et al. 1996 | Atlantic Forest | *Baccharis crispa* Spreng. | Asteraceae | H | N | N | Calming | MBD |
| Hanazaki et al. 1996 | Atlantic Forest | *Baccharis crispa* Spreng. | Asteraceae | H | N | N | Circulatory problems | DCS |
| Hanazaki et al. 1996 | Atlantic Forest | *Baccharis crispa* Spreng. | Asteraceae | H | N | N | Hemorrhoids | DCS |
| Hanazaki et al. 1996 | Atlantic Forest | *Baccharis crispa* Spreng. | Asteraceae | H | N | N | High blood pressure | DCS |
| Hanazaki et al. 1996 | Atlantic Forest | *Baccharis crispa* Spreng. | Asteraceae | H | N | N | Liver problems | DDS |
| Hanazaki et al. 1996 | Atlantic Forest | *Baccharis crispa* Spreng. | Asteraceae | H | N | N | Stomach problems | DDS |
| Hanazaki et al. 1996 | Atlantic Forest | *Baccharis crispa* Spreng. | Asteraceae | H | N | N | Uterine wound | DGS |
| Hanazaki et al. 1996 | Atlantic Forest | *Cecropia glaziovii* Snethl. | Urticaceae | T | N | N | Bronchitis | DRS |
| Hanazaki et al. 1996 | Atlantic Forest | *Cecropia glaziovii* Snethl. | Urticaceae | T | N | N | Bronchitis | DRS |
| Hanazaki et al. 1996 | Atlantic Forest | *Cecropia glaziovii* Snethl. | Urticaceae | T | N | N | Influenza | DRS |
| Hanazaki et al. 1996 | Atlantic Forest | *Cecropia glaziovii* Snethl. | Urticaceae | T | N | N | Influenza | DRS |
| Hanazaki et al. 1996 | Atlantic Forest | *Citrus limon* (L.) Osbeck | Rutaceae | T | E | E | Bronchitis | DRS |
| Hanazaki et al. 1996 | Atlantic Forest | *Citrus limon* (L.) Osbeck | Rutaceae | T | E | E | Bronchitis | DRS |
| Hanazaki et al. 1996 | Atlantic Forest | *Citrus limon* (L.) Osbeck | Rutaceae | T | E | E | Cough | SSNEC |
| Hanazaki et al. 1996 | Atlantic Forest | *Citrus limon* (L.) Osbeck | Rutaceae | T | E | E | Cough | SSNEC |
| Hanazaki et al. 1996 | Atlantic Forest | *Citrus limon* (L.) Osbeck | Rutaceae | T | E | E | Influenza | DRS |
| Hanazaki et al. 1996 | Atlantic Forest | *Citrus limon* (L.) Osbeck | Rutaceae | T | E | E | Influenza | DRS |
| Hanazaki et al. 1996 | Atlantic Forest | *Citrus sinensis* (L.) Osbeck | Rutaceae | T | E | E | Calming | MBD |
| Hanazaki et al. 1996 | Atlantic Forest | *Citrus sinensis* (L.) Osbeck | Rutaceae | T | E | E | Calming | MBD |
| Hanazaki et al. 1996 | Atlantic Forest | *Citrus sinensis* (L.) Osbeck | Rutaceae | T | E | E | Dizziness | SSNEC |
| Hanazaki et al. 1996 | Atlantic Forest | *Citrus sinensis* (L.) Osbeck | Rutaceae | T | E | E | Dizziness | SSNEC |
| Hanazaki et al. 1996 | Atlantic Forest | *Citrus sinensis* (L.) Osbeck | Rutaceae | T | E | E | Headache | SSNEC |
| Hanazaki et al. 1996 | Atlantic Forest | *Citrus sinensis* (L.) Osbeck | Rutaceae | T | E | E | Headache | SSNEC |
| Hanazaki et al. 1996 | Atlantic Forest | *Citrus sinensis* (L.) Osbeck | Rutaceae | T | E | E | Influenza | DRS |
| Hanazaki et al. 1996 | Atlantic Forest | *Citrus sinensis* (L.) Osbeck | Rutaceae | T | E | E | Influenza | DRS |
| Hanazaki et al. 1996 | Atlantic Forest | *Cymbopogon citratus* (DC.) Stapf | Poaceae | H | E | E | Anti-emetic | SSNEC |
| Hanazaki et al. 1996 | Atlantic Forest | *Cymbopogon citratus* (DC.) Stapf | Poaceae | H | E | E | Calming | MBD |
| Hanazaki et al. 1996 | Atlantic Forest | *Cymbopogon citratus* (DC.) Stapf | Poaceae | H | E | E | Calming | MBD |
| Hanazaki et al. 1996 | Atlantic Forest | *Cymbopogon citratus* (DC.) Stapf | Poaceae | H | E | E | Colds | DRS |
| Hanazaki et al. 1996 | Atlantic Forest | *Cymbopogon citratus* (DC.) Stapf | Poaceae | H | E | E | Dizziness | SSNEC |
| Hanazaki et al. 1996 | Atlantic Forest | *Cymbopogon citratus* (DC.) Stapf | Poaceae | H | E | E | High blood pressure | DCS |
| Hanazaki et al. 1996 | Atlantic Forest | *Cymbopogon citratus* (DC.) Stapf | Poaceae | H | E | E | Influenza | DRS |
| Hanazaki et al. 1996 | Atlantic Forest | *Cymbopogon citratus* (DC.) Stapf | Poaceae | H | E | E | Liver problems | DDS |
| Hanazaki et al. 1996 | Atlantic Forest | *Cymbopogon citratus* (DC.) Stapf | Poaceae | H | E | E | Poor digestion | DDS |
| Hanazaki et al. 1996 | Atlantic Forest | *Cymbopogon citratus* (DC.) Stapf | Poaceae | H | E | E | Poor digestion | DDS |
| Hanazaki et al. 1996 | Atlantic Forest | *Eugenia uniflora* L. | Myrtaceae | T | N | N | Colds | DRS |
| Hanazaki et al. 1996 | Atlantic Forest | *Eugenia uniflora* L. | Myrtaceae | T | N | N | Colds | DRS |
| Hanazaki et al. 1996 | Atlantic Forest | *Eugenia uniflora* L. | Myrtaceae | T | N | N | Cough | SSNEC |
| Hanazaki et al. 1996 | Atlantic Forest | *Eugenia uniflora* L. | Myrtaceae | T | N | N | Cough | SSNEC |
| Hanazaki et al. 1996 | Atlantic Forest | *Eugenia uniflora* L. | Myrtaceae | T | N | N | Throat pain | DRS |
| Hanazaki et al. 1996 | Atlantic Forest | *Eugenia uniflora* L. | Myrtaceae | T | N | N | Throat pain | DRS |
| Hanazaki et al. 1996 | Atlantic Forest | *Foeniculum vulgare* Mill. | Apiaceae | H | E | E | Anti-emetic | SSNEC |
| Hanazaki et al. 1996 | Atlantic Forest | *Foeniculum vulgare* Mill. | Apiaceae | H | E | E | Childhood illnesses | SSNEC |
| Hanazaki et al. 1996 | Atlantic Forest | *Foeniculum vulgare* Mill. | Apiaceae | H | E | E | Infant colic | SSNEC |
| Hanazaki et al. 1996 | Atlantic Forest | *Foeniculum vulgare* Mill. | Apiaceae | H | E | E | Insomnia | DNS |
| Hanazaki et al. 1996 | Atlantic Forest | *Foeniculum vulgare* Mill. | Apiaceae | H | E | E | Intoxication | IPEC |
| Hanazaki et al. 1996 | Atlantic Forest | *Foeniculum vulgare* Mill. | Apiaceae | H | E | E | Stomach problems | DDS |
| Hanazaki et al. 1996 | Atlantic Forest | *Matricaria chamomilla* L. | Asteraceae | H | E | E | Childhood illnesses | SSNEC |
| Hanazaki et al. 1996 | Atlantic Forest | *Matricaria chamomilla* L. | Asteraceae | H | E | E | Colds | DRS |
| Hanazaki et al. 1996 | Atlantic Forest | *Matricaria chamomilla* L. | Asteraceae | H | E | E | Fever | SSNEC |
| Hanazaki et al. 1996 | Atlantic Forest | *Matricaria chamomilla* L. | Asteraceae | H | E | E | Helminthiasis | CIPD |
| Hanazaki et al. 1996 | Atlantic Forest | *Matricaria chamomilla* L. | Asteraceae | H | E | E | Influenza | DRS |
| Hanazaki et al. 1996 | Atlantic Forest | *Matricaria chamomilla* L. | Asteraceae | H | E | E | Pain | SSNEC |
| Hanazaki et al. 1996 | Atlantic Forest | *Melissa officinalis* L. | Lamiaceae | H | E | E | Calming | MBD |
| Hanazaki et al. 1996 | Atlantic Forest | *Melissa officinalis* L. | Lamiaceae | H | E | E | Colds | DRS |
| Hanazaki et al. 1996 | Atlantic Forest | *Melissa officinalis* L. | Lamiaceae | H | E | E | Infection | CIPD |
| Hanazaki et al. 1996 | Atlantic Forest | *Melissa officinalis* L. | Lamiaceae | H | E | E | Influenza | DRS |
| Hanazaki et al. 1996 | Atlantic Forest | *Melissa officinalis* L. | Lamiaceae | H | E | E | Poor digestion | DDS |
| Hanazaki et al. 1996 | Atlantic Forest | *Mentha × piperita* L. | Lamiaceae | H | E | E | Calming | MBD |
| Hanazaki et al. 1996 | Atlantic Forest | *Mentha × piperita* L. | Lamiaceae | H | E | E | Childhood illnesses | SSNEC |
| Hanazaki et al. 1996 | Atlantic Forest | *Mentha × piperita* L. | Lamiaceae | H | E | E | Colic | SSNEC |
| Hanazaki et al. 1996 | Atlantic Forest | *Mentha × piperita* L. | Lamiaceae | H | E | E | Fever | SSNEC |
| Hanazaki et al. 1996 | Atlantic Forest | *Mentha × piperita* L. | Lamiaceae | H | E | E | Helminthiasis | CIPD |
| Hanazaki et al. 1996 | Atlantic Forest | *Persea americana* Mill. | Lauraceae | T | E | E | Liver problems | DDS |
| Hanazaki et al. 1996 | Atlantic Forest | *Persea americana* Mill. | Lauraceae | T | E | E | Liver problems | DDS |
| Hanazaki et al. 1996 | Atlantic Forest | *Persea americana* Mill. | Lauraceae | T | E | E | Stomach problems | DDS |
| Hanazaki et al. 1996 | Atlantic Forest | *Persea americana* Mill. | Lauraceae | T | E | E | Stomach problems | DDS |
| Hanazaki et al. 1996 | Atlantic Forest | *Plectranthus barbatus* Andr. | Lamiaceae | H | E | E | Colic | SSNEC |
| Hanazaki et al. 1996 | Atlantic Forest | *Plectranthus barbatus* Andr. | Lamiaceae | H | E | E | Liver problems | DDS |
| Hanazaki et al. 1996 | Atlantic Forest | *Plectranthus barbatus* Andr. | Lamiaceae | H | E | E | Stomach problems | DDS |
| Hanazaki et al. 1996 | Atlantic Forest | *Psidium guajava* L. | Myrtaceae | S | E | E | Bronchitis | DRS |
| Hanazaki et al. 1996 | Atlantic Forest | *Psidium guajava* L. | Myrtaceae | S | E | E | Bronchitis | DRS |
| Hanazaki et al. 1996 | Atlantic Forest | *Psidium guajava* L. | Myrtaceae | S | E | E | Stomach pain/Diarrhea | SSNEC |
| Hanazaki et al. 1996 | Atlantic Forest | *Psidium guajava* L. | Myrtaceae | S | E | E | Stomach pain/Diarrhea | SSNEC |
| Hanazaki et al. 1996 | Atlantic Forest | *Psidium guajava* L. | Myrtaceae | S | E | E | Stomach pain/Diarrhea | SSNEC |
| Hanazaki et al. 1996 | Atlantic Forest | *Psidium guajava* L. | Myrtaceae | S | E | E | Stomach pain/Diarrhea | SSNEC |
| Lima et al. 2007 | Atlantic Forest | *Achillea millefolium* L. | Asteraceae | H | E | E | Headache | SSNEC |
| Lima et al. 2007 | Atlantic Forest | *Aloe arborescens* Mill. | Xanthorrhoeaceae | S | E | E | Burns | IPEC |
| Lima et al. 2007 | Atlantic Forest | *Aloe arborescens* Mill. | Xanthorrhoeaceae | S | E | E | Hair care | DSST |
| Lima et al. 2007 | Atlantic Forest | *Aloe arborescens* Mill. | Xanthorrhoeaceae | S | E | E | Ulcer | DDS |
| Lima et al. 2007 | Atlantic Forest | *Aloe arborescens* Mill. | Xanthorrhoeaceae | S | E | E | Wound | IPEC |
| Lima et al. 2007 | Atlantic Forest | *Aloe vera* (L.) Burm.f. | Xanthorrhoeaceae | S | E | E | Burns | IPEC |
| Lima et al. 2007 | Atlantic Forest | *Aloe vera* (L.) Burm.f. | Xanthorrhoeaceae | S | E | E | Hair care | DSST |
| Lima et al. 2007 | Atlantic Forest | *Alpinia zerumbet* (Pers.) B.L.Burtt & R.M.Sm. | Zingiberaceae | H | E | E | Diabetes | ENMD |
| Lima et al. 2007 | Atlantic Forest | *Alpinia zerumbet* (Pers.) B.L.Burtt & R.M.Sm. | Zingiberaceae | H | E | E | High blood pressure | DCS |
| Lima et al. 2007 | Atlantic Forest | *Alpinia zerumbet* (Pers.) B.L.Burtt & R.M.Sm. | Zingiberaceae | H | E | E | Stomach problems | DDS |
| Lima et al. 2007 | Atlantic Forest | *Alternanthera dentata* (Moench) Stuchlík ex R.E.Fr. | Amaranthaceae | H | N | N | Feminine hygiene | DGS |
| Lima et al. 2007 | Atlantic Forest | *Alternanthera dentata* (Moench) Stuchlík ex R.E.Fr. | Amaranthaceae | H | N | N | Pain | SSNEC |
| Lima et al. 2007 | Atlantic Forest | *Alternanthera dentata* (Moench) Stuchlík ex R.E.Fr. | Amaranthaceae | H | N | N | Regulation of female reproductive system | DGS |
| Lima et al. 2007 | Atlantic Forest | *Artemisia absinthium* L. | Asteraceae | H | E | E | Stomach pain/Diarrhea | SSNEC |
| Lima et al. 2007 | Atlantic Forest | *Artemisia vulgaris* L | Asteraceae | H | E | E | Pain | SSNEC |
| Lima et al. 2007 | Atlantic Forest | *Artemisia vulgaris* L | Asteraceae | H | E | E | Stomach problems | DDS |
| Lima et al. 2007 | Atlantic Forest | *Averrhoa carambola* L. | Oxalidaceae | T | E | E | Circulatory problems | DCS |
| Lima et al. 2007 | Atlantic Forest | *Averrhoa carambola* L. | Oxalidaceae | T | E | E | Diabetes | ENMD |
| Lima et al. 2007 | Atlantic Forest | *Baccharis crispa* Spreng. | Asteraceae | H | N | N | Diabetes | ENMD |
| Lima et al. 2007 | Atlantic Forest | *Baccharis crispa* Spreng. | Asteraceae | H | N | N | Hematoma | SSNEC |
| Lima et al. 2007 | Atlantic Forest | *Baccharis crispa* Spreng. | Asteraceae | H | N | N | Stomach problems | DDS |
| Lima et al. 2007 | Atlantic Forest | *Brassica oleracea* L. | Brassicaceae | H | E | E | Stomach problems | DDS |
| Lima et al. 2007 | Atlantic Forest | *Cissus sulcicaulis* (Baker) Planch. | Vitaceae | S | N | N | Kidney problems | DGS |
| Lima et al. 2007 | Atlantic Forest | *Cissus verticillata* (L.) Nicolson & C.E.Jarvis | Vitaceae | S | N | N | Diabetes | ENMD |
| Lima et al. 2007 | Atlantic Forest | *Coronopus didymus* (L.) Sm. | Brassicaceae | H | N | N | Inflammation | SSNEC |
| Lima et al. 2007 | Atlantic Forest | *Costus spicatus* (Jacq.) Sw. | Costaceae | S | E | E | Kidney problems | DGS |
| Lima et al. 2007 | Atlantic Forest | *Costus spicatus* (Jacq.) Sw. | Costaceae | S | E | E | Kidney problems | DGS |
| Lima et al. 2007 | Atlantic Forest | *Crassula argentea* Thunb. | Crassulaceae | S | E | E | Earache | DEMP |
| Lima et al. 2007 | Atlantic Forest | *Crassula argentea* Thunb. | Crassulaceae | S | E | E | Stomach problems | DDS |
| Lima et al. 2007 | Atlantic Forest | *Cuphea carthagenensis* (Jacq.) J.Macbr. | Lythraceae | H | N | N | Diabetes | ENMD |
| Lima et al. 2007 | Atlantic Forest | *Cuphea carthagenensis* (Jacq.) J.Macbr. | Lythraceae | H | N | N | High blood pressure | DCS |
| Lima et al. 2007 | Atlantic Forest | *Cymbopogon citratus* (DC.) Stapf | Poaceae | H | E | E | Calming | MBD |
| Lima et al. 2007 | Atlantic Forest | *Cymbopogon citratus* (DC.) Stapf | Poaceae | H | E | E | Colds | DRS |
| Lima et al. 2007 | Atlantic Forest | *Cymbopogon citratus* (DC.) Stapf | Poaceae | H | E | E | Headache | SSNEC |
| Lima et al. 2007 | Atlantic Forest | *Cymbopogon citratus* (DC.) Stapf | Poaceae | H | E | E | High blood pressure | DCS |
| Lima et al. 2007 | Atlantic Forest | *Cymbopogon citratus* (DC.) Stapf | Poaceae | H | E | E | Influenza | DRS |
| Lima et al. 2007 | Atlantic Forest | *Cymbopogon citratus* (DC.) Stapf | Poaceae | H | E | E | Insomnia | DNS |
| Lima et al. 2007 | Atlantic Forest | *Foeniculum vulgare* Mill. | Apiaceae | H | E | E | Calming | MBD |
| Lima et al. 2007 | Atlantic Forest | *Foeniculum vulgare* Mill. | Apiaceae | H | E | E | High blood pressure | DCS |
| Lima et al. 2007 | Atlantic Forest | *Fragaria vesca* L. | Rosaceae | H | E | E | Kidney problems | DGS |
| Lima et al. 2007 | Atlantic Forest | *Gymnanthemum amygdalinum* (Delile) Sch.Bip. ex Walp. | Asteraceae | S | N | N | Stomach problems | DDS |
| Lima et al. 2007 | Atlantic Forest | *Jatropha multifida* L. | Euphorbiaceae | S | E | E | Wound | IPEC |
| Lima et al. 2007 | Atlantic Forest | *Leonurus sibiricus* L. | Lamiaceae | H | E | E | Wound | IPEC |
| Lima et al. 2007 | Atlantic Forest | *Lippia alba* (Mill.) N.E.Br. | Verbenaceae | S | N | N | High blood pressure | DCS |
| Lima et al. 2007 | Atlantic Forest | *Lippia alba* (Mill.) N.E.Br. | Verbenaceae | S | N | N | High blood pressure | DCS |
| Lima et al. 2007 | Atlantic Forest | *Lippia alba* (Mill.) N.E.Br. | Verbenaceae | S | N | N | Influenza | DRS |
| Lima et al. 2007 | Atlantic Forest | *Lippia alba* (Mill.) N.E.Br. | Verbenaceae | S | N | N | Influenza | DRS |
| Lima et al. 2007 | Atlantic Forest | *Mentha pulegium* L. | Lamiaceae | H | E | E | Cough | SSNEC |
| Lima et al. 2007 | Atlantic Forest | *Mentha pulegium* L. | Lamiaceae | H | E | E | Inflammation | SSNEC |
| Lima et al. 2007 | Atlantic Forest | *Mentha pulegium* L. | Lamiaceae | H | E | E | Influenza | DRS |
| Lima et al. 2007 | Atlantic Forest | *Mentha spicata* L. | Lamiaceae | H | E | E | Calming | MBD |
| Lima et al. 2007 | Atlantic Forest | *Mentha spicata* L. | Lamiaceae | H | E | E | Calming | MBD |
| Lima et al. 2007 | Atlantic Forest | *Mentha spicata* L. | Lamiaceae | H | E | E | Helminthiasis | CIPD |
| Lima et al. 2007 | Atlantic Forest | *Mentha spicata* L. | Lamiaceae | H | E | E | Helminthiasis | CIPD |
| Lima et al. 2007 | Atlantic Forest | *Mentha spicata* L. | Lamiaceae | H | E | E | Influenza | DRS |
| Lima et al. 2007 | Atlantic Forest | *Mentha spicata* L. | Lamiaceae | H | E | E | Influenza | DRS |
| Lima et al. 2007 | Atlantic Forest | *Mikania glomerata* Spreng. | Asteraceae | S | N | N | Cough | SSNEC |
| Lima et al. 2007 | Atlantic Forest | *Mikania glomerata* Spreng. | Asteraceae | S | N | N | Influenza | DRS |
| Lima et al. 2007 | Atlantic Forest | *Monstera deliciosa* Liebm. | Araceae | H | E | E | Wound | IPEC |
| Lima et al. 2007 | Atlantic Forest | *Ocimum gratissimum* L. | Lamiaceae | H | E | E | Bronchitis | DRS |
| Lima et al. 2007 | Atlantic Forest | *Ocimum gratissimum* L. | Lamiaceae | H | E | E | Cough | SSNEC |
| Lima et al. 2007 | Atlantic Forest | *Ocimum gratissimum* L. | Lamiaceae | H | E | E | Influenza | DRS |
| Lima et al. 2007 | Atlantic Forest | *Petiveria alliacea* L. | Phytolaccaceae | H | E | E | Fever | SSNEC |
| Lima et al. 2007 | Atlantic Forest | *Petiveria alliacea* L. | Phytolaccaceae | H | E | E | Wound | IPEC |
| Lima et al. 2007 | Atlantic Forest | *Petroselinum crispum* (Mill.) Fuss | Apiaceae | H | E | E | Kidney problems | DGS |
| Lima et al. 2007 | Atlantic Forest | *Petroselinum crispum* (Mill.) Fuss | Apiaceae | H | E | E | Urinary pain | DGS |
| Lima et al. 2007 | Atlantic Forest | *Phyllanthus niruri* L. | Phyllanthaceae | H | N | N | Kidney problems | DGS |
| Lima et al. 2007 | Atlantic Forest | *Plectranthus barbatus* Andr. | Lamiaceae | H | E | E | Hangover | MBD |
| Lima et al. 2007 | Atlantic Forest | *Plectranthus barbatus* Andr. | Lamiaceae | H | E | E | Liver problems | DDS |
| Lima et al. 2007 | Atlantic Forest | *Plectranthus barbatus* Andr. | Lamiaceae | H | E | E | Nausea | SSNEC |
| Lima et al. 2007 | Atlantic Forest | *Plectranthus barbatus* Andr. | Lamiaceae | H | E | E | Poor digestion | DDS |
| Lima et al. 2007 | Atlantic Forest | *Plectranthus barbatus* Andr. | Lamiaceae | H | E | E | Stomach problems | DDS |
| Lima et al. 2007 | Atlantic Forest | *Porophyllum ruderale* (Jacq.) Cass. | Asteraceae | H | N | E | Joint pain | DMS |
| Lima et al. 2007 | Atlantic Forest | *Porophyllum ruderale* (Jacq.) Cass. | Asteraceae | H | N | E | Wound | IPEC |
| Lima et al. 2007 | Atlantic Forest | *Psidium guajava* L. | Myrtaceae | S | E | E | Stomach pain/Diarrhea | SSNEC |
| Lima et al. 2007 | Atlantic Forest | *Punica granatum* L. | Lythraceae | T | E | E | Throat infection | DRS |
| Lima et al. 2007 | Atlantic Forest | *Rosmarinus officinalis* L. | Lamiaceae | S | E | E | Cardiac problems | DCS |
| Lima et al. 2007 | Atlantic Forest | *Rosmarinus officinalis* L. | Lamiaceae | S | E | E | Cardiac problems | DCS |
| Lima et al. 2007 | Atlantic Forest | *Rosmarinus officinalis* L. | Lamiaceae | S | E | E | Headache | SSNEC |
| Lima et al. 2007 | Atlantic Forest | *Rosmarinus officinalis* L. | Lamiaceae | S | E | E | Headache | SSNEC |
| Lima et al. 2007 | Atlantic Forest | *Ruta graveolens* L. | Rutaceae | H | E | E | Arthrosis | DMS |
| Lima et al. 2007 | Atlantic Forest | *Ruta graveolens* L. | Rutaceae | H | E | E | Conjunctivitis | DEA |
| Lima et al. 2007 | Atlantic Forest | *Ruta graveolens* L. | Rutaceae | H | E | E | Headache | SSNEC |
| Lima et al. 2007 | Atlantic Forest | *Ruta graveolens* L. | Rutaceae | H | E | E | Malaise | SSNEC |
| Lima et al. 2007 | Atlantic Forest | *Ruta graveolens* L. | Rutaceae | H | E | E | Pain | SSNEC |
| Lima et al. 2007 | Atlantic Forest | *Sedum dendroideum* DC. | Crassulaceae | H | E | E | Pain | SSNEC |
| Lima et al. 2007 | Atlantic Forest | *Sedum dendroideum* DC. | Crassulaceae | H | E | E | Stomach problems | DDS |
| Lima et al. 2007 | Atlantic Forest | *Sedum dendroideum* DC. | Crassulaceae | H | E | E | Wound | IPEC |
| Lima et al. 2007 | Atlantic Forest | *Symphytum* officinale L. | Boraginaceae | H | E | E | Pain | SSNEC |
| Lima et al. 2007 | Atlantic Forest | *Symphytum* officinale L. | Boraginaceae | H | E | E | Skin blemishes | DSST |
| Lima et al. 2007 | Atlantic Forest | *Symphytum* officinale L. | Boraginaceae | H | E | E | Wound | IPEC |
| Lima et al. 2007 | Atlantic Forest | *Zingiber officinale* Roscoe | Zingiberaceae | H | E | E | Influenza | DRS |
| Lima et al. 2007 | Atlantic Forest | *Zingiber officinale* Roscoe | Zingiberaceae | H | E | E | Influenza | DRS |
| Lima et al. 2007 | Atlantic Forest | *Zingiber officinale* Roscoe | Zingiberaceae | H | E | E | Throat problems | DRS |
| Lima et al. 2007 | Atlantic Forest | *Zingiber officinale* Roscoe | Zingiberaceae | H | E | E | Throat problems | DRS |
| Lima et al. 2009 | Atlantic Forest | *Anemopaegma arvense* (Vell.) Stellfeld ex de Souza | Bignoniaceae | H | N | N | Aphrodisiac | DGS |
| Lima et al. 2009 | Atlantic Forest | *Anemopaegma arvense* (Vell.) Stellfeld ex de Souza | Bignoniaceae | H | N | N | Stimulant | SSNEC |
| Lima et al. 2009 | Atlantic Forest | *Aristolochia cymbifera* Mart. & Zucc. | Aristolochiaceae | S | N | N | Aphrodisiac | DGS |
| Lima et al. 2009 | Atlantic Forest | *Aristolochia cymbifera* Mart. & Zucc. | Aristolochiaceae | S | N | N | Stomach problems | DDS |
| Lima et al. 2009 | Atlantic Forest | *Baccharis crispa* Spreng. | Asteraceae | H | N | N | Fever | SSNEC |
| Lima et al. 2009 | Atlantic Forest | *Baccharis crispa* Spreng. | Asteraceae | H | N | N | Liver problems | DDS |
| Lima et al. 2009 | Atlantic Forest | *Baccharis crispa* Spreng. | Asteraceae | H | N | N | Stomach problems | DDS |
| Lima et al. 2009 | Atlantic Forest | *Bowdichia virgilioides* Kunth | Fabaceae | T | N | N | Arthrosis | DMS |
| Lima et al. 2009 | Atlantic Forest | *Bowdichia virgilioides* Kunth | Fabaceae | T | N | N | Bone spur | DMS |
| Lima et al. 2009 | Atlantic Forest | *Bowdichia virgilioides* Kunth | Fabaceae | T | N | N | Problems with spinal column | DMS |
| Lima et al. 2009 | Atlantic Forest | *Cordia ecalyculata* Vell. | Boraginaceae | S | N | N | Diuretic | SSNEC |
| Lima et al. 2009 | Atlantic Forest | *Cordia ecalyculata* Vell. | Boraginaceae | S | N | N | Diuretic | SSNEC |
| Lima et al. 2009 | Atlantic Forest | *Cordia ecalyculata* Vell. | Boraginaceae | S | N | N | Kidney problems | DGS |
| Lima et al. 2009 | Atlantic Forest | *Cordia ecalyculata* Vell. | Boraginaceae | S | N | N | Kidney problems | DGS |
| Lima et al. 2009 | Atlantic Forest | *Cynara cardunculus* L. | Asteraceae | H | E | E | Diuretic | SSNEC |
| Lima et al. 2009 | Atlantic Forest | *EchinoPainus grandiflorus (Cham. & Schltr.) Micheli* | Alismataceae | H | N | N | Diuretic | SSNEC |
| Lima et al. 2009 | Atlantic Forest | *EchinoPainus grandiflorus (Cham. & Schltr.) Micheli* | Alismataceae | H | N | N | Kidney problems | DGS |
| Lima et al. 2009 | Atlantic Forest | *EchinoPainus grandiflorus (Cham. & Schltr.) Micheli* | Alismataceae | H | N | N | Kidney problems | DGS |
| Lima et al. 2009 | Atlantic Forest | *Ginkgo biloba* L. | Ginkgoaceae | T | E | E | Mental stimulant | DNS |
| Lima et al. 2009 | Atlantic Forest | *Ginkgo biloba* L. | Ginkgoaceae | T | E | E | Mental stimulant | DNS |
| Lima et al. 2009 | Atlantic Forest | *Heteropterys tomentosa* A.Juss. | Malpighiaceae | S | N | E | Aphrodisiac | DGS |
| Lima et al. 2009 | Atlantic Forest | *Heteropterys tomentosa* A.Juss. | Malpighiaceae | S | N | E | Stimulant | SSNEC |
| Lima et al. 2009 | Atlantic Forest | *Hymenaea courbaril* L. | Fabaceae | T | N | N | Depurative | DBBO |
| Lima et al. 2009 | Atlantic Forest | *Hymenaea courbaril* L. | Fabaceae | T | N | N | Expectorant | SSNEC |
| Lima et al. 2009 | Atlantic Forest | *Ipomoea purga* (Wender.) Hayne | Convolvulaceae | H | E | E | Helminthiasis | CIPD |
| Lima et al. 2009 | Atlantic Forest | *Ipomoea purga* (Wender.) Hayne | Convolvulaceae | H | E | E | STD | CIPD |
| Lima et al. 2009 | Atlantic Forest | *Laurus nobilis* L. | Lauraceae | T | E | E | Constipation | DDS |
| Lima et al. 2009 | Atlantic Forest | *Laurus nobilis* L. | Lauraceae | T | E | E | Liver problems | DDS |
| Lima et al. 2009 | Atlantic Forest | *Libidibia ferrea* (Mart. ex Tul.) L.P.Queiroz | Fabaceae | T | N | N | Diabetes | ENMD |
| Lima et al. 2009 | Atlantic Forest | *Libidibia ferrea* (Mart. ex Tul.) L.P.Queiroz | Fabaceae | T | N | N | Rheumatism | DMS |
| Lima et al. 2009 | Atlantic Forest | *Luffa operculata* (L.) Cogn. | Cucurbitaceae | H | N | E | Liver problems | DDS |
| Lima et al. 2009 | Atlantic Forest | *Luffa operculata* (L.) Cogn. | Cucurbitaceae | H | N | E | Sinusitis | DRS |
| Lima et al. 2009 | Atlantic Forest | *Matricaria chamomilla* L. | Asteraceae | H | E | E | Calming | MBD |
| Lima et al. 2009 | Atlantic Forest | *Matricaria chamomilla* L. | Asteraceae | H | E | E | Calming | MBD |
| Lima et al. 2009 | Atlantic Forest | *Matricaria chamomilla* L. | Asteraceae | H | E | E | Calming | MBD |
| Lima et al. 2009 | Atlantic Forest | *Matricaria chamomilla* L. | Asteraceae | H | E | E | Fever | SSNEC |
| Lima et al. 2009 | Atlantic Forest | *Matricaria chamomilla* L. | Asteraceae | H | E | E | Fever | SSNEC |
| Lima et al. 2009 | Atlantic Forest | *Matricaria chamomilla* L. | Asteraceae | H | E | E | Fever | SSNEC |
| Lima et al. 2009 | Atlantic Forest | *Maytenus ilicifolia* Mart. ex Reissek | Celastraceae | S | N | N | Gastritis | DDS |
| Lima et al. 2009 | Atlantic Forest | *Maytenus ilicifolia* Mart. ex Reissek | Celastraceae | S | N | N | Ulcer | DDS |
| Lima et al. 2009 | Atlantic Forest | *Mentha pulegium* L. | Lamiaceae | H | E | E | Influenza | DRS |
| Lima et al. 2009 | Atlantic Forest | *Momordica charantia* L. | Cucurbitaceae | H | E | E | Abortifacient | PCP |
| Lima et al. 2009 | Atlantic Forest | *Momordica charantia* L. | Cucurbitaceae | H | E | E | Abortifacient | PCP |
| Lima et al. 2009 | Atlantic Forest | *Momordica charantia* L. | Cucurbitaceae | H | E | E | Wound | IPEC |
| Lima et al. 2009 | Atlantic Forest | *Momordica charantia* L. | Cucurbitaceae | H | E | E | Wound | IPEC |
| Lima et al. 2009 | Atlantic Forest | *Ocimum basilicum* L. | Lamiaceae | H | E | E | Calming | MBD |
| Lima et al. 2009 | Atlantic Forest | *Plantago lanceolata* L. | Plantaginaceae | H | E | E | Inflammation | SSNEC |
| Lima et al. 2009 | Atlantic Forest | *Plantago lanceolata* L. | Plantaginaceae | H | E | E | Throat problems | DRS |
| Lima et al. 2009 | Atlantic Forest | *Polygonum punctatum* Elliott | Polygonaceae | H | N | N | Itchiness | DSST |
| Lima et al. 2009 | Atlantic Forest | *Quassia amara* L. | Simaroubaceae | S | N | N | Cough | SSNEC |
| Lima et al. 2009 | Atlantic Forest | *Quassia amara* L. | Simaroubaceae | S | N | N | Diabetes | ENMD |
| Lima et al. 2009 | Atlantic Forest | *Quassia amara* L. | Simaroubaceae | S | N | N | High cholesterol | ENMD |
| Lima et al. 2009 | Atlantic Forest | *Rosmarinus officinalis* L. | Lamiaceae | S | E | E | Calming | MBD |
| Lima et al. 2009 | Atlantic Forest | *Rosmarinus officinalis* L. | Lamiaceae | S | E | E | Calming | MBD |
| Lima et al. 2009 | Atlantic Forest | *Ruta graveolens* L. | Rutaceae | H | E | E | Cough | SSNEC |
| Lima et al. 2009 | Atlantic Forest | *Schinus molle* L. | Anacardiaceae | T | N | N | Wound | IPEC |
| Lima et al. 2009 | Atlantic Forest | *Senna alexandrina* Mill. | Fabaceae | S | N | E | Laxative | DDS |
| Lima et al. 2009 | Atlantic Forest | *Stryphnodendron aSTDringens (Mart.) Coville* | Fabaceae | T | N | E | Inflammation | SSNEC |
| Lima et al. 2009 | Atlantic Forest | *Stryphnodendron aSTDringens (Mart.) Coville* | Fabaceae | T | N | E | Wound | IPEC |
| Lima et al. 2009 | Atlantic Forest | *Uncaria tomentosa* (Willd. ex Roem. & Schult.) DC. | Rubiaceae | S | N | E | Arthrosis | DMS |
| Lima et al. 2009 | Atlantic Forest | *Uncaria tomentosa* (Willd. ex Roem. & Schult.) DC. | Rubiaceae | S | N | E | Arthrosis | DMS |
| Lima et al. 2009 | Atlantic Forest | *Uncaria tomentosa* (Willd. ex Roem. & Schult.) DC. | Rubiaceae | S | N | E | Osteoporosis | DMS |
| Lima et al. 2009 | Atlantic Forest | *Uncaria tomentosa* (Willd. ex Roem. & Schult.) DC. | Rubiaceae | S | N | E | Osteoporosis | DMS |
| Lima et al. 2009 | Atlantic Forest | *Vernonanthura phosphorica* (Vell.) H.Rob. | Asteraceae | S | N | N | Influenza | DRS |
| Lima et al. 2009 | Atlantic Forest | *Vernonanthura phosphorica* (Vell.) H.Rob. | Asteraceae | S | N | N | Pulmonary problems | DRS |
| Macedo et al. 2007 | Cerrado and Atlantic Forest | *Baccharis crispa* Spreng. | Asteraceae | H | N | N | Calming | MBD |
| Macedo et al. 2007 | Cerrado and Atlantic Forest | *Baccharis crispa* Spreng. | Asteraceae | H | N | N | Improves digestion | DDS |
| Macedo et al. 2007 | Cerrado and Atlantic Forest | *Camellia sinensis* (L.) Kuntze | Theaceae | T | E | E | Circulatory problems | DCS |
| Macedo et al. 2007 | Cerrado and Atlantic Forest | *Matricaria chamomilla* L. | Asteraceae | H | E | E | Calming | MBD |
| Macedo et al. 2007 | Cerrado and Atlantic Forest | *Matricaria chamomilla* L. | Asteraceae | H | E | E | Sinusitis | DRS |
| Macedo et al. 2007 | Cerrado and Atlantic Forest | *Maytenus ilicifolia* Mart. ex Reissek | Celastraceae | S | N | N | Stomach problems | DDS |
| Macedo et al. 2007 | Cerrado and Atlantic Forest | *Melissa officinalis* L. | Lamiaceae | H | E | E | Cardiac problems | DCS |
| Macedo et al. 2007 | Cerrado and Atlantic Forest | *Melissa officinalis* L. | Lamiaceae | H | E | E | Insomnia | DNS |
| Macedo et al. 2007 | Cerrado and Atlantic Forest | *Melissa officinalis* L. | Lamiaceae | H | E | E | Irritation | SSNEC |
| Macedo et al. 2007 | Cerrado and Atlantic Forest | *Melissa officinalis* L. | Lamiaceae | H | E | E | Stomach pain/Diarrhea | SSNEC |
| Macedo et al. 2007 | Cerrado and Atlantic Forest | *Mentha × piperita* L. | Lamiaceae | H | E | E | Influenza | DRS |
| Macedo et al. 2007 | Cerrado and Atlantic Forest | *Mentha × piperita* L. | Lamiaceae | H | E | E | Insomnia | DNS |
| Macedo et al. 2007 | Cerrado and Atlantic Forest | *Mikania glomerata* Spreng. | Asteraceae | S | N | N | Influenza | DRS |
| Macedo et al. 2007 | Cerrado and Atlantic Forest | *Morus alba* L. | Moraceae | T | E | E | Diabetes | ENMD |
| Macedo et al. 2007 | Cerrado and Atlantic Forest | *Peumus boldus* Molina | Monimiaceae | T | E | E | Improves digestion | DDS |
| Macedo et al. 2007 | Cerrado and Atlantic Forest | *Peumus boldus* Molina | Monimiaceae | T | E | E | Influenza | DRS |
| Macedo et al. 2007 | Cerrado and Atlantic Forest | *Peumus boldus* Molina | Monimiaceae | T | E | E | Malaise | SSNEC |
| Macedo et al. 2007 | Cerrado and Atlantic Forest | *Phyllanthus niruri* L. | Phyllanthaceae | H | N | N | Kidney problems | DGS |
| Macedo et al. 2007 | Cerrado and Atlantic Forest | *Pterodon emarginatus* Vogel | Fabaceae | T | N | N | Inflammation | SSNEC |
| Macedo et al. 2007 | Cerrado and Atlantic Forest | *Valeriana officinalis* L. | Caprifoliaceae | H | E | E | Stress | DGS |
| Maciel & Guarim-Neto 2006 | Amazon | *Allium sativum* L. | Alliaceae | H | E | E | Cough | SSNEC |
| Maciel & Guarim-Neto 2006 | Amazon | *Allium sativum* L. | Alliaceae | H | E | E | Helminthiasis | CIPD |
| Maciel & Guarim-Neto 2006 | Amazon | *Amburana cearensis* (Allemão) A.C.Sm. | Fabaceae | T | N | E | Influenza | DRS |
| Maciel & Guarim-Neto 2006 | Amazon | *Annona muricata* L. | Annonaceae | T | E | E | Influenza | DRS |
| Maciel & Guarim-Neto 2006 | Amazon | *Begonia aconitifolia* DC. | Begoniaceae | H | N | E | Vaginal discharge | DGS |
| Maciel & Guarim-Neto 2006 | Amazon | *Bidens pilosa* L. | Asteraceae | H | E | E | Hepatitis | CIPD |
| Maciel & Guarim-Neto 2006 | Amazon | *Carica papaya* L. | Caricaceae | T | E | E | Helminthiasis | CIPD |
| Maciel & Guarim-Neto 2006 | Amazon | *Carica papaya* L. | Caricaceae | T | E | E | Poor digestion | DDS |
| Maciel & Guarim-Neto 2006 | Amazon | *Carica papaya* L. | Caricaceae | T | E | E | Skin cleanser | DSST |
| Maciel & Guarim-Neto 2006 | Amazon | *Cedrela oPainata L.* | Meliaceae | T | N | N | Malaria | CIPD |
| Maciel & Guarim-Neto 2006 | Amazon | *Chenopodium ambrosioides* L. | Amaranthaceae | H | N | N | Helminthiasis | CIPD |
| Maciel & Guarim-Neto 2006 | Amazon | *Chenopodium ambrosioides* L. | Amaranthaceae | H | N | N | Shock in children | DGS |
| Maciel & Guarim-Neto 2006 | Amazon | *Cinnamomum verum* J. Presl | Lauraceae | T | E | E | Internal infection | CIPD |
| Maciel & Guarim-Neto 2006 | Amazon | *Copaifera langsPainffii Desf.* | Fabaceae | T | N | N | Wound | IPEC |
| Maciel & Guarim-Neto 2006 | Amazon | *Cucumis sativus* L. | Cucurbitaceae | H | E | E | High blood pressure | DCS |
| Maciel & Guarim-Neto 2006 | Amazon | *Cuphea carthagenensis* (Jacq.) J.Macbr. | Lythraceae | H | N | N | Diarrhea in children | DDS |
| Maciel & Guarim-Neto 2006 | Amazon | *EchinoPainus macrophyllus (Kunth) Micheli* | Alismataceae | H | N | N | Diuretic | SSNEC |
| Maciel & Guarim-Neto 2006 | Amazon | *Eugenia uniflora* L. | Myrtaceae | T | N | E | Low blood pressure | DCS |
| Maciel & Guarim-Neto 2006 | Amazon | *Eugenia uniflora* L. | Myrtaceae | T | N | E | Stomach pain/Diarrhea | SSNEC |
| Maciel & Guarim-Neto 2006 | Amazon | *Euphorbia potentilloides* Boiss. | Euphorbiaceae | H | N | E | Calming | MBD |
| Maciel & Guarim-Neto 2006 | Amazon | *Euphorbia potentilloides* Boiss. | Euphorbiaceae | H | N | E | Influenza | DRS |
| Maciel & Guarim-Neto 2006 | Amazon | *Euphorbia potentilloides* Boiss. | Euphorbiaceae | H | N | E | Pulmonary pain | DRS |
| Maciel & Guarim-Neto 2006 | Amazon | *Gossypium herbaceum* L. | Malvaceae | S | E | E | Infection | CIPD |
| Maciel & Guarim-Neto 2006 | Amazon | *Handroanthus heptaphyllus* Mattos | Bignoniaceae | T | N | E | Cough | SSNEC |
| Maciel & Guarim-Neto 2006 | Amazon | *Handroanthus heptaphyllus* Mattos | Bignoniaceae | T | N | E | Pulmonary problems | DRS |
| Maciel & Guarim-Neto 2006 | Amazon | *Handroanthus heptaphyllus* Mattos | Bignoniaceae | T | N | E | Uterine wound | DGS |
| Maciel & Guarim-Neto 2006 | Amazon | *Hymenaea stigonocarpa* Mart. ex Hayne | Fabaceae | T | N | N | Cough | SSNEC |
| Maciel & Guarim-Neto 2006 | Amazon | *Hymenaea stigonocarpa* Mart. ex Hayne | Fabaceae | T | N | N | Uterine wound | DGS |
| Maciel & Guarim-Neto 2006 | Amazon | *Jacaranda decurrens* Cham. | Bignoniaceae | S | N | E | Depurative | DBBO |
| Maciel & Guarim-Neto 2006 | Amazon | *Justicia pectoralis* Jacq. | Acanthaceae | H | N | N | Headache | SSNEC |
| Maciel & Guarim-Neto 2006 | Amazon | *Lippia alba* (Mill.) N.E.Br. | Verbenaceae | S | N | N | Calming | MBD |
| Maciel & Guarim-Neto 2006 | Amazon | *Lippia alba* (Mill.) N.E.Br. | Verbenaceae | S | N | N | Fortifier | SSNEC |
| Maciel & Guarim-Neto 2006 | Amazon | *Mangifera indica* L. | Anacardiaceae | T | E | E | Influenza | DRS |
| Maciel & Guarim-Neto 2006 | Amazon | *Manihot esculenta* Crantz | Euphorbiaceae | S | N | N | Malnutrition | ENMD |
| Maciel & Guarim-Neto 2006 | Amazon | *Matricaria chamomilla* L. | Asteraceae | H | E | E | Intestinal cleansing in children | DDS |
| Maciel & Guarim-Neto 2006 | Amazon | *Maytenus ilicifolia* Mart. ex Reissek | Celastraceae | S | N | E | Cancer | NEO |
| Maciel & Guarim-Neto 2006 | Amazon | *Melissa officinalis* L. | Lamiaceae | H | E | E | Calming | MBD |
| Maciel & Guarim-Neto 2006 | Amazon | *Melissa officinalis* L. | Lamiaceae | H | E | E | Infection | CIPD |
| Maciel & Guarim-Neto 2006 | Amazon | *Mentha × piperita* L. | Lamiaceae | H | E | E | Helminthiasis | CIPD |
| Maciel & Guarim-Neto 2006 | Amazon | *Mentha × piperita* L. | Lamiaceae | H | E | E | Shock in children | DGS |
| Maciel & Guarim-Neto 2006 | Amazon | *Mentha pulegium* L. | Lamiaceae | H | E | E | Calming (for children) | MBD |
| Maciel & Guarim-Neto 2006 | Amazon | *Momordica charantia* L. | Cucurbitaceae | H | E | E | Helminthiasis | CIPD |
| Maciel & Guarim-Neto 2006 | Amazon | *Ocimum basilicum* L. | Lamiaceae | H | E | E | Blow, punch | IPEC |
| Maciel & Guarim-Neto 2006 | Amazon | *Ocimum basilicum* L. | Lamiaceae | H | E | E | Helminthiasis | CIPD |
| Maciel & Guarim-Neto 2006 | Amazon | *Ocimum basilicum* L. | Lamiaceae | H | E | E | Influenza | DRS |
| Maciel & Guarim-Neto 2006 | Amazon | *Petiveria alliacea* L. | Phytolaccaceae | H | E | E | Rheumatism | DMS |
| Maciel & Guarim-Neto 2006 | Amazon | *Petroselinum crispum* (Mill.) Fuss | Apiaceae | H | E | E | Ear infection | DEMP |
| Maciel & Guarim-Neto 2006 | Amazon | *Petroselinum crispum* (Mill.) Fuss | Apiaceae | H | E | E | Hepatitis | CIPD |
| Maciel & Guarim-Neto 2006 | Amazon | *Phanera glabra* (Jacq.) Vaz | Fabaceae | S | N | N | Hemorrhoids | DCS |
| Maciel & Guarim-Neto 2006 | Amazon | *Phyllanthus orbiculatus* Rich. | Phyllanthaceae | H | N | N | Kidney stone | DGS |
| Maciel & Guarim-Neto 2006 | Amazon | *Pimpinella anisum* L. | Apiaceae | H | E | E | Breast milk production | COP |
| Maciel & Guarim-Neto 2006 | Amazon | *Pimpinella anisum* L. | Apiaceae | H | E | E | Stomach pain/Diarrhea | SSNEC |
| Maciel & Guarim-Neto 2006 | Amazon | *Piper nigrum* L. | Piperaceae | S | E | E | Depression | MBD |
| Maciel & Guarim-Neto 2006 | Amazon | *Plantago major* L. | Plantaginaceae | H | E | E | Infection of the uterus | DGS |
| Maciel & Guarim-Neto 2006 | Amazon | *Plinia cauliflora* (Mart.) Kausel | Myrtaceae | T | N | E | Influenza | DRS |
| Maciel & Guarim-Neto 2006 | Amazon | *Plinia cauliflora* (Mart.) Kausel | Myrtaceae | T | N | E | Stomach pain/Diarrhea | SSNEC |
| Maciel & Guarim-Neto 2006 | Amazon | *Pseudobrickellia brasiliensis* (Spreng.) R.M.King & H.Rob. | Asteraceae | S | N | N | Prostate problems | DGS |
| Maciel & Guarim-Neto 2006 | Amazon | *Psidium guajava* L. | Myrtaceae | S | E | E | Stomach pain/Diarrhea | SSNEC |
| Maciel & Guarim-Neto 2006 | Amazon | *Rosmarinus officinalis* L. | Lamiaceae | S | E | E | Cardiac problems | DCS |
| Maciel & Guarim-Neto 2006 | Amazon | *Rosmarinus officinalis* L. | Lamiaceae | S | E | E | Infection | CIPD |
| Maciel & Guarim-Neto 2006 | Amazon | *Ruta graveolens* L. | Rutaceae | H | E | E | Eyewash | DEA |
| Maciel & Guarim-Neto 2006 | Amazon | *Ruta graveolens* L. | Rutaceae | H | E | E | Helminthiasis | CIPD |
| Maciel & Guarim-Neto 2006 | Amazon | *Salvia officinalis* L. | Lamiaceae | S | E | E | Infection | CIPD |
| Maciel & Guarim-Neto 2006 | Amazon | *Salvia officinalis* L. | Lamiaceae | S | E | E | Influenza | DRS |
| Maciel & Guarim-Neto 2006 | Amazon | *Sechium edule* (Jacq.) Sw. | Cucurbitaceae | H | E | E | High blood pressure | DCS |
| Maciel & Guarim-Neto 2006 | Amazon | *Spondias purpurea* L. | Anacardiaceae | T | E | E | Influenza | DRS |
| Maciel & Guarim-Neto 2006 | Amazon | *Stryphnodendron aSTDringens (Mart.) Coville* | Fabaceae | T | N | E | Uterine wound | DGS |
| Maciel & Guarim-Neto 2006 | Amazon | *Symphytum* officinale L. | Boraginaceae | H | E | E | Inflammation | SSNEC |
| Maciel & Guarim-Neto 2006 | Amazon | *Syzygium aromaticum* (L.) Merr. & L. M. Perry | Myrtaceae | T | E | E | Infection | CIPD |
| Maciel & Guarim-Neto 2006 | Amazon | *Tabebuia aurea* (Silva Manso) Benth. & Hook.f. ex S.Moore | Bignoniaceae | T | N | N | Uterine wound | DGS |
| Maciel & Guarim-Neto 2006 | Amazon | *Tanacetum vulgare* L. | Asteraceae | H | E | E | Injury | IPEC |
| Maciel & Guarim-Neto 2006 | Amazon | *Theobroma grandiflorum* (Willd. ex Spreng.) K.Schum. | Malvaceae | T | N | N | Fortifier | SSNEC |
| Maciel & Guarim-Neto 2006 | Amazon | *Vernonanthura phosphorica* (Vell.) H.Rob. | Asteraceae | S | N | N | Abortifacient | PCP |
| Maciel & Guarim-Neto 2006 | Amazon | *Zingiber officinale* Roscoe | Zingiberaceae | H | E | E | Influenza | DRS |
| Maciel & Guarim-Neto 2006 | Amazon | *Zingiber officinale* Roscoe | Zingiberaceae | H | E | E | Throat problems | DRS |
| Merétika et al. 2010 | Atlantic Forest | *Achyrocline satureioides* (Lam.) DC. | Asteraceae | S | N | N | Poor digestion | DDS |
| Merétika et al. 2010 | Atlantic Forest | *Achyrocline satureioides* (Lam.) DC. | Asteraceae | S | N | N | Stomach pain/Diarrhea | SSNEC |
| Merétika et al. 2010 | Atlantic Forest | *Achyrocline satureioides* (Lam.) DC. | Asteraceae | S | N | N | Stomach problems | DDS |
| Merétika et al. 2010 | Atlantic Forest | *Ageratum conyzoides* L. | Asteraceae | H | N | N | Back pain | DMS |
| Merétika et al. 2010 | Atlantic Forest | *Ageratum conyzoides* L. | Asteraceae | H | N | N | Back pain | DMS |
| Merétika et al. 2010 | Atlantic Forest | *Ageratum conyzoides* L. | Asteraceae | H | N | N | Back pain | DMS |
| Merétika et al. 2010 | Atlantic Forest | *Ageratum conyzoides* L. | Asteraceae | H | N | N | Inflammation | SSNEC |
| Merétika et al. 2010 | Atlantic Forest | *Ageratum conyzoides* L. | Asteraceae | H | N | N | Inflammation | SSNEC |
| Merétika et al. 2010 | Atlantic Forest | *Ageratum conyzoides* L. | Asteraceae | H | N | N | Inflammation | SSNEC |
| Merétika et al. 2010 | Atlantic Forest | *Ageratum conyzoides* L. | Asteraceae | H | N | N | Leg pain | DMS |
| Merétika et al. 2010 | Atlantic Forest | *Ageratum conyzoides* L. | Asteraceae | H | N | N | Leg pain | DMS |
| Merétika et al. 2010 | Atlantic Forest | *Ageratum conyzoides* L. | Asteraceae | H | N | N | Leg pain | DMS |
| Merétika et al. 2010 | Atlantic Forest | *Ageratum conyzoides* L. | Asteraceae | H | N | N | Pain | SSNEC |
| Merétika et al. 2010 | Atlantic Forest | *Ageratum conyzoides* L. | Asteraceae | H | N | N | Pain | SSNEC |
| Merétika et al. 2010 | Atlantic Forest | *Ageratum conyzoides* L. | Asteraceae | H | N | N | Pain | SSNEC |
| Merétika et al. 2010 | Atlantic Forest | *Ageratum conyzoides* L. | Asteraceae | H | N | N | Rheumatism | DMS |
| Merétika et al. 2010 | Atlantic Forest | *Ageratum conyzoides* L. | Asteraceae | H | N | N | Rheumatism | DMS |
| Merétika et al. 2010 | Atlantic Forest | *Ageratum conyzoides* L. | Asteraceae | H | N | N | Rheumatism | DMS |
| Merétika et al. 2010 | Atlantic Forest | *Aloe arborescens* Mill. | Xanthorrhoeaceae | S | E | E | Depurative | DBBO |
| Merétika et al. 2010 | Atlantic Forest | *Aloe arborescens* Mill. | Xanthorrhoeaceae | S | E | E | Hair loss | DSST |
| Merétika et al. 2010 | Atlantic Forest | *Aloe arborescens* Mill. | Xanthorrhoeaceae | S | E | E | Heartburn | DGS |
| Merétika et al. 2010 | Atlantic Forest | *Aloe arborescens* Mill. | Xanthorrhoeaceae | S | E | E | Insect bite | IPEC |
| Merétika et al. 2010 | Atlantic Forest | *Aloe arborescens* Mill. | Xanthorrhoeaceae | S | E | E | Wound | IPEC |
| Merétika et al. 2010 | Atlantic Forest | *Aloysia citrioPaina Palau* | Verbenaceae | S | E | E | Influenza | DRS |
| Merétika et al. 2010 | Atlantic Forest | *Aloysia citrioPaina Palau* | Verbenaceae | S | E | E | Sedative | MBD |
| Merétika et al. 2010 | Atlantic Forest | *Alternanthera dentata* (Moench) Stuchlík ex R.E.Fr. | Amaranthaceae | H | N | N | Infection | CIPD |
| Merétika et al. 2010 | Atlantic Forest | *Aristolochia triangularis* Cham. & Schltdl. | Aristolochiaceae | S | N | N | Diabetes | ENMD |
| Merétika et al. 2010 | Atlantic Forest | *Aristolochia triangularis* Cham. & Schltdl. | Aristolochiaceae | S | N | N | Fatigue | SSNEC |
| Merétika et al. 2010 | Atlantic Forest | *Aristolochia triangularis* Cham. & Schltdl. | Aristolochiaceae | S | N | N | High cholesterol | ENMD |
| Merétika et al. 2010 | Atlantic Forest | *Artemisia absinthium* L. | Asteraceae | H | E | E | Intoxication | IPEC |
| Merétika et al. 2010 | Atlantic Forest | *Artemisia absinthium* L. | Asteraceae | H | E | E | Stomach pain/Diarrhea | SSNEC |
| Merétika et al. 2010 | Atlantic Forest | *Artemisia absinthium* L. | Asteraceae | H | E | E | Stomach problems | DDS |
| Merétika et al. 2010 | Atlantic Forest | *Baccharis crispa* Spreng. | Asteraceae | H | N | N | High cholesterol | ENMD |
| Merétika et al. 2010 | Atlantic Forest | *Baccharis crispa* Spreng. | Asteraceae | H | N | N | Kidney problems | DGS |
| Merétika et al. 2010 | Atlantic Forest | *Baccharis crispa* Spreng. | Asteraceae | H | N | N | Stomach problems | DDS |
| Merétika et al. 2010 | Atlantic Forest | *Baccharis crispa* Spreng. | Asteraceae | H | N | N | Weight reduction | ENMD |
| Merétika et al. 2010 | Atlantic Forest | *Bauhinia forficata* Link | Fabaceae | T | N | N | Cystitis | DGS |
| Merétika et al. 2010 | Atlantic Forest | *Bauhinia forficata* Link | Fabaceae | T | N | N | Diabetes | ENMD |
| Merétika et al. 2010 | Atlantic Forest | *Bauhinia forficata* Link | Fabaceae | T | N | N | Kidney problems | DGS |
| Merétika et al. 2010 | Atlantic Forest | *Bauhinia forficata* Link | Fabaceae | T | N | N | Kidney stone | DGS |
| Merétika et al. 2010 | Atlantic Forest | *Chenopodium ambrosioides* L. | Amaranthaceae | H | N | N | Helminthiasis | CIPD |
| Merétika et al. 2010 | Atlantic Forest | *Chenopodium ambrosioides* L. | Amaranthaceae | H | N | N | Helminthiasis | CIPD |
| Merétika et al. 2010 | Atlantic Forest | *Chenopodium ambrosioides* L. | Amaranthaceae | H | N | N | Lice | CIPD |
| Merétika et al. 2010 | Atlantic Forest | *Chenopodium ambrosioides* L. | Amaranthaceae | H | N | N | Lice | CIPD |
| Merétika et al. 2010 | Atlantic Forest | *Citrus aurantiifolia* (Christm.) Swingle | Rutaceae | T | E | E | Influenza | DRS |
| Merétika et al. 2010 | Atlantic Forest | *Citrus aurantiifolia* (Christm.) Swingle | Rutaceae | T | E | E | Influenza | DRS |
| Merétika et al. 2010 | Atlantic Forest | *Citrus aurantiifolia* (Christm.) Swingle | Rutaceae | T | E | E | Pneumonia | DRS |
| Merétika et al. 2010 | Atlantic Forest | *Citrus aurantiifolia* (Christm.) Swingle | Rutaceae | T | E | E | Pneumonia | DRS |
| Merétika et al. 2010 | Atlantic Forest | *Citrus aurantiifolia* (Christm.) Swingle | Rutaceae | T | E | E | Stomach pain/Diarrhea | SSNEC |
| Merétika et al. 2010 | Atlantic Forest | *Citrus aurantiifolia* (Christm.) Swingle | Rutaceae | T | E | E | Stomach pain/Diarrhea | SSNEC |
| Merétika et al. 2010 | Atlantic Forest | *Coffea arabica* L. | Rubiaceae | S | E | E | Pneumonia | DRS |
| Merétika et al. 2010 | Atlantic Forest | *Coffea arabica* L. | Rubiaceae | S | E | E | Wound | IPEC |
| Merétika et al. 2010 | Atlantic Forest | *Costus spicatus* (Jacq.) Sw. | Costaceae | S | E | E | Diuretic | SSNEC |
| Merétika et al. 2010 | Atlantic Forest | *Costus spicatus* (Jacq.) Sw. | Costaceae | S | E | E | Diuretic | SSNEC |
| Merétika et al. 2010 | Atlantic Forest | *Costus spicatus* (Jacq.) Sw. | Costaceae | S | E | E | Kidney stone | DGS |
| Merétika et al. 2010 | Atlantic Forest | *Costus spicatus* (Jacq.) Sw. | Costaceae | S | E | E | Kidney stone | DGS |
| Merétika et al. 2010 | Atlantic Forest | *Cuphea carthagenensis* (Jacq.) J.Macbr. | Lythraceae | H | N | N | Circulatory problems | DCS |
| Merétika et al. 2010 | Atlantic Forest | *Cuphea carthagenensis* (Jacq.) J.Macbr. | Lythraceae | H | N | N | Circulatory problems | DCS |
| Merétika et al. 2010 | Atlantic Forest | *Cuphea carthagenensis* (Jacq.) J.Macbr. | Lythraceae | H | N | N | Diabetes | ENMD |
| Merétika et al. 2010 | Atlantic Forest | *Cuphea carthagenensis* (Jacq.) J.Macbr. | Lythraceae | H | N | N | Diabetes | ENMD |
| Merétika et al. 2010 | Atlantic Forest | *Cuphea carthagenensis* (Jacq.) J.Macbr. | Lythraceae | H | N | N | Hypertension | DCS |
| Merétika et al. 2010 | Atlantic Forest | *Cuphea carthagenensis* (Jacq.) J.Macbr. | Lythraceae | H | N | N | Hypertension | DCS |
| Merétika et al. 2010 | Atlantic Forest | *Cuphea carthagenensis* (Jacq.) J.Macbr. | Lythraceae | H | N | N | Kidney problems | DGS |
| Merétika et al. 2010 | Atlantic Forest | *Cuphea carthagenensis* (Jacq.) J.Macbr. | Lythraceae | H | N | N | Kidney problems | DGS |
| Merétika et al. 2010 | Atlantic Forest | *Cuphea carthagenensis* (Jacq.) J.Macbr. | Lythraceae | H | N | N | Liver problems | DDS |
| Merétika et al. 2010 | Atlantic Forest | *Cuphea carthagenensis* (Jacq.) J.Macbr. | Lythraceae | H | N | N | Liver problems | DDS |
| Merétika et al. 2010 | Atlantic Forest | *Cuscuta racemosa* Mart. | Convolvulaceae | H | N | N | Blow, punch | IPEC |
| Merétika et al. 2010 | Atlantic Forest | *Cuscuta racemosa* Mart. | Convolvulaceae | H | N | N | Cough | SSNEC |
| Merétika et al. 2010 | Atlantic Forest | *Cuscuta racemosa* Mart. | Convolvulaceae | H | N | N | Kidney problems | DGS |
| Merétika et al. 2010 | Atlantic Forest | *Cymbopogon citratus* (DC.) Stapf | Poaceae | H | E | E | Dyspnea | SSNEC |
| Merétika et al. 2010 | Atlantic Forest | *Cymbopogon citratus* (DC.) Stapf | Poaceae | H | E | E | Influenza | DRS |
| Merétika et al. 2010 | Atlantic Forest | *Cymbopogon citratus* (DC.) Stapf | Poaceae | H | E | E | Sedative | MBD |
| Merétika et al. 2010 | Atlantic Forest | *EchinoPainus grandiflorus (Cham. & Schltr.) Micheli* | Alismataceae | H | N | N | Bladder problems | DGS |
| Merétika et al. 2010 | Atlantic Forest | *EchinoPainus grandiflorus (Cham. & Schltr.) Micheli* | Alismataceae | H | N | N | Cystitis | DGS |
| Merétika et al. 2010 | Atlantic Forest | *EchinoPainus grandiflorus (Cham. & Schltr.) Micheli* | Alismataceae | H | N | N | Kidney stone | DGS |
| Merétika et al. 2010 | Atlantic Forest | *Equisetum hyemale* L. | Equisetaceae | H | E | E | Bladder problems | DGS |
| Merétika et al. 2010 | Atlantic Forest | *Equisetum hyemale* L. | Equisetaceae | H | E | E | Bladder problems | DGS |
| Merétika et al. 2010 | Atlantic Forest | *Equisetum hyemale* L. | Equisetaceae | H | E | E | Bladder problems | DGS |
| Merétika et al. 2010 | Atlantic Forest | *Equisetum hyemale* L. | Equisetaceae | H | E | E | Kidney pain | DGS |
| Merétika et al. 2010 | Atlantic Forest | *Equisetum hyemale* L. | Equisetaceae | H | E | E | Kidney pain | DGS |
| Merétika et al. 2010 | Atlantic Forest | *Equisetum hyemale* L. | Equisetaceae | H | E | E | Kidney pain | DGS |
| Merétika et al. 2010 | Atlantic Forest | *Equisetum hyemale* L. | Equisetaceae | H | E | E | Kidney problems | DGS |
| Merétika et al. 2010 | Atlantic Forest | *Equisetum hyemale* L. | Equisetaceae | H | E | E | Kidney problems | DGS |
| Merétika et al. 2010 | Atlantic Forest | *Equisetum hyemale* L. | Equisetaceae | H | E | E | Kidney problems | DGS |
| Merétika et al. 2010 | Atlantic Forest | *Euphorbia tirucalli* L. | Euphorbiaceae | S | E | E | Cancer | NEO |
| Merétika et al. 2010 | Atlantic Forest | *Euphorbia tirucalli* L. | Euphorbiaceae | S | E | E | Cancer | NEO |
| Merétika et al. 2010 | Atlantic Forest | *Euphorbia tirucalli* L. | Euphorbiaceae | S | E | E | Kidney problems | DGS |
| Merétika et al. 2010 | Atlantic Forest | *Euphorbia tirucalli* L. | Euphorbiaceae | S | E | E | Kidney problems | DGS |
| Merétika et al. 2010 | Atlantic Forest | *Euphorbia tirucalli* L. | Euphorbiaceae | S | E | E | Leukemia | NEO |
| Merétika et al. 2010 | Atlantic Forest | *Euphorbia tirucalli* L. | Euphorbiaceae | S | E | E | Leukemia | NEO |
| Merétika et al. 2010 | Atlantic Forest | *Euphorbia tirucalli* L. | Euphorbiaceae | S | E | E | Warts | CIPD |
| Merétika et al. 2010 | Atlantic Forest | *Euphorbia tirucalli* L. | Euphorbiaceae | S | E | E | Warts | CIPD |
| Merétika et al. 2010 | Atlantic Forest | *Euphorbia tirucalli* L. | Euphorbiaceae | S | E | E | Wound | IPEC |
| Merétika et al. 2010 | Atlantic Forest | *Euphorbia tirucalli* L. | Euphorbiaceae | S | E | E | Wound | IPEC |
| Merétika et al. 2010 | Atlantic Forest | *Foeniculum vulgare* Mill. | Apiaceae | H | E | E | Cough | SSNEC |
| Merétika et al. 2010 | Atlantic Forest | *Foeniculum vulgare* Mill. | Apiaceae | H | E | E | Cough | SSNEC |
| Merétika et al. 2010 | Atlantic Forest | *Foeniculum vulgare* Mill. | Apiaceae | H | E | E | Flatulence | SSNEC |
| Merétika et al. 2010 | Atlantic Forest | *Foeniculum vulgare* Mill. | Apiaceae | H | E | E | Flatulence | SSNEC |
| Merétika et al. 2010 | Atlantic Forest | *Foeniculum vulgare* Mill. | Apiaceae | H | E | E | Sedative | MBD |
| Merétika et al. 2010 | Atlantic Forest | *Foeniculum vulgare* Mill. | Apiaceae | H | E | E | Sedative | MBD |
| Merétika et al. 2010 | Atlantic Forest | *Kalanchoe delagoensis* Eckl. & Zeyh. | Crassulaceae | H | E | E | Leg pain | DMS |
| Merétika et al. 2010 | Atlantic Forest | *Lantana canescens* Kunth | Verbenaceae | S | N | N | Blow, punch | IPEC |
| Merétika et al. 2010 | Atlantic Forest | *Lantana canescens* Kunth | Verbenaceae | S | N | N | Blow, punch | IPEC |
| Merétika et al. 2010 | Atlantic Forest | *Lantana canescens* Kunth | Verbenaceae | S | N | N | Diuretic | SSNEC |
| Merétika et al. 2010 | Atlantic Forest | *Lantana canescens* Kunth | Verbenaceae | S | N | N | Diuretic | SSNEC |
| Merétika et al. 2010 | Atlantic Forest | *Lantana canescens* Kunth | Verbenaceae | S | N | N | Toothache | DDS |
| Merétika et al. 2010 | Atlantic Forest | *Lantana canescens* Kunth | Verbenaceae | S | N | N | Toothache | DDS |
| Merétika et al. 2010 | Atlantic Forest | *Lippia alba* (Mill.) N.E.Br. | Verbenaceae | S | N | N | Discomfort | SSNEC |
| Merétika et al. 2010 | Atlantic Forest | *Lippia alba* (Mill.) N.E.Br. | Verbenaceae | S | N | N | Fever | SSNEC |
| Merétika et al. 2010 | Atlantic Forest | *Lippia alba* (Mill.) N.E.Br. | Verbenaceae | S | N | N | Influenza | DRS |
| Merétika et al. 2010 | Atlantic Forest | *Lippia alba* (Mill.) N.E.Br. | Verbenaceae | S | N | N | Sedative | MBD |
| Merétika et al. 2010 | Atlantic Forest | *Melissa officinalis* L. | Lamiaceae | H | E | E | Hypertension | DCS |
| Merétika et al. 2010 | Atlantic Forest | *Melissa officinalis* L. | Lamiaceae | H | E | E | Inflammation | SSNEC |
| Merétika et al. 2010 | Atlantic Forest | *Melissa officinalis* L. | Lamiaceae | H | E | E | Influenza | DRS |
| Merétika et al. 2010 | Atlantic Forest | *Melissa officinalis* L. | Lamiaceae | H | E | E | Ringworm | CIPD |
| Merétika et al. 2010 | Atlantic Forest | *Melissa officinalis* L. | Lamiaceae | H | E | E | Sedative | MBD |
| Merétika et al. 2010 | Atlantic Forest | *Mikania cordifolia* (L.f.) Willd. | Asteraceae | S | N | N | Bronchitis | DRS |
| Merétika et al. 2010 | Atlantic Forest | *Mikania cordifolia* (L.f.) Willd. | Asteraceae | S | N | N | Cough | SSNEC |
| Merétika et al. 2010 | Atlantic Forest | *Passiflora alata* Curtis | Passifloraceae | S | N | N | Hypertension | DCS |
| Merétika et al. 2010 | Atlantic Forest | *Passiflora alata* Curtis | Passifloraceae | S | N | N | Sedative | MBD |
| Merétika et al. 2010 | Atlantic Forest | *Persea americana* Mill. | Lauraceae | T | E | E | Constipation | DDS |
| Merétika et al. 2010 | Atlantic Forest | *Persea americana* Mill. | Lauraceae | T | E | E | Constipation | DDS |
| Merétika et al. 2010 | Atlantic Forest | *Persea americana* Mill. | Lauraceae | T | E | E | Diabetes | ENMD |
| Merétika et al. 2010 | Atlantic Forest | *Persea americana* Mill. | Lauraceae | T | E | E | Diabetes | ENMD |
| Merétika et al. 2010 | Atlantic Forest | *Persea americana* Mill. | Lauraceae | T | E | E | High cholesterol | ENMD |
| Merétika et al. 2010 | Atlantic Forest | *Persea americana* Mill. | Lauraceae | T | E | E | High cholesterol | ENMD |
| Merétika et al. 2010 | Atlantic Forest | *Persea americana* Mill. | Lauraceae | T | E | E | Kidney problems | DGS |
| Merétika et al. 2010 | Atlantic Forest | *Persea americana* Mill. | Lauraceae | T | E | E | Kidney problems | DGS |
| Merétika et al. 2010 | Atlantic Forest | *Petiveria alliacea* L. | Phytolaccaceae | H | E | E | Inflammation | SSNEC |
| Merétika et al. 2010 | Atlantic Forest | *Phyllanthus niruri* L. | Phyllanthaceae | H | N | N | Diuretic | SSNEC |
| Merétika et al. 2010 | Atlantic Forest | *Phyllanthus niruri* L. | Phyllanthaceae | H | N | N | Diuretic | SSNEC |
| Merétika et al. 2010 | Atlantic Forest | *Phyllanthus niruri* L. | Phyllanthaceae | H | N | N | Kidney problems | DGS |
| Merétika et al. 2010 | Atlantic Forest | *Phyllanthus niruri* L. | Phyllanthaceae | H | N | N | Kidney problems | DGS |
| Merétika et al. 2010 | Atlantic Forest | *Phyllanthus niruri* L. | Phyllanthaceae | H | N | N | Kidney stone | DGS |
| Merétika et al. 2010 | Atlantic Forest | *Phyllanthus niruri* L. | Phyllanthaceae | H | N | N | Kidney stone | DGS |
| Merétika et al. 2010 | Atlantic Forest | *Phyllanthus niruri* L. | Phyllanthaceae | H | N | N | Renal infection | DGS |
| Merétika et al. 2010 | Atlantic Forest | *Phyllanthus niruri* L. | Phyllanthaceae | H | N | N | Renal infection | DGS |
| Merétika et al. 2010 | Atlantic Forest | *Phyllanthus tenellus* Roxb. | Phyllanthaceae | H | N | N | Diuretic | SSNEC |
| Merétika et al. 2010 | Atlantic Forest | *Phyllanthus tenellus* Roxb. | Phyllanthaceae | H | N | N | Diuretic | SSNEC |
| Merétika et al. 2010 | Atlantic Forest | *Phyllanthus tenellus* Roxb. | Phyllanthaceae | H | N | N | Kidney problems | DGS |
| Merétika et al. 2010 | Atlantic Forest | *Phyllanthus tenellus* Roxb. | Phyllanthaceae | H | N | N | Kidney problems | DGS |
| Merétika et al. 2010 | Atlantic Forest | *Phyllanthus tenellus* Roxb. | Phyllanthaceae | H | N | N | Kidney stone | DGS |
| Merétika et al. 2010 | Atlantic Forest | *Phyllanthus tenellus* Roxb. | Phyllanthaceae | H | N | N | Kidney stone | DGS |
| Merétika et al. 2010 | Atlantic Forest | *Plantago major* L. | Plantaginaceae | H | E | E | Infection | CIPD |
| Merétika et al. 2010 | Atlantic Forest | *Plantago major* L. | Plantaginaceae | H | E | E | Inflammation of the throat | DRS |
| Merétika et al. 2010 | Atlantic Forest | *Plantago tomentosa* Lam. | Plantaginaceae | H | N | N | Cystitis | DGS |
| Merétika et al. 2010 | Atlantic Forest | *Plantago tomentosa* Lam. | Plantaginaceae | H | N | N | Cystitis | DGS |
| Merétika et al. 2010 | Atlantic Forest | *Plantago tomentosa* Lam. | Plantaginaceae | H | N | N | Infection | CIPD |
| Merétika et al. 2010 | Atlantic Forest | *Plantago tomentosa* Lam. | Plantaginaceae | H | N | N | Infection | CIPD |
| Merétika et al. 2010 | Atlantic Forest | *Plantago tomentosa* Lam. | Plantaginaceae | H | N | N | Inflammation | SSNEC |
| Merétika et al. 2010 | Atlantic Forest | *Plantago tomentosa* Lam. | Plantaginaceae | H | N | N | Inflammation | SSNEC |
| Merétika et al. 2010 | Atlantic Forest | *Plantago tomentosa* Lam. | Plantaginaceae | H | N | N | Inflammation of the throat | DRS |
| Merétika et al. 2010 | Atlantic Forest | *Plantago tomentosa* Lam. | Plantaginaceae | H | N | N | Pain | SSNEC |
| Merétika et al. 2010 | Atlantic Forest | *Plantago tomentosa* Lam. | Plantaginaceae | H | N | N | Pain | SSNEC |
| Merétika et al. 2010 | Atlantic Forest | *Plantago tomentosa* Lam. | Plantaginaceae | H | N | N | Throat infection | DRS |
| Merétika et al. 2010 | Atlantic Forest | *Plantago tomentosa* Lam. | Plantaginaceae | H | N | N | Throat infection | DRS |
| Merétika et al. 2010 | Atlantic Forest | *Plantago tomentosa* Lam. | Plantaginaceae | H | N | N | Throat problems | DRS |
| Merétika et al. 2010 | Atlantic Forest | *Plantago tomentosa* Lam. | Plantaginaceae | H | N | N | Throat problems | DRS |
| Merétika et al. 2010 | Atlantic Forest | *Plectranthus barbatus* Andr. | Lamiaceae | H | E | E | Hangover | MBD |
| Merétika et al. 2010 | Atlantic Forest | *Plectranthus barbatus* Andr. | Lamiaceae | H | E | E | Stomach pain/Diarrhea | SSNEC |
| Merétika et al. 2010 | Atlantic Forest | *Plectranthus barbatus* Andr. | Lamiaceae | H | E | E | Stomach problems | DDS |
| Merétika et al. 2010 | Atlantic Forest | *Plectranthus neochilus* Schltr. | Lamiaceae | H | E | E | Poor digestion | DDS |
| Merétika et al. 2010 | Atlantic Forest | *Plectranthus neochilus* Schltr. | Lamiaceae | H | E | E | Stomach pain/Diarrhea | SSNEC |
| Merétika et al. 2010 | Atlantic Forest | *Plectranthus neochilus* Schltr. | Lamiaceae | H | E | E | Stomach problems | DDS |
| Merétika et al. 2010 | Atlantic Forest | *Polygala paniculata* L. | Polygalaceae | H | N | N | Arm pain | DMS |
| Merétika et al. 2010 | Atlantic Forest | *Polygala paniculata* L. | Polygalaceae | H | N | N | Arm pain | DMS |
| Merétika et al. 2010 | Atlantic Forest | *Polygala paniculata* L. | Polygalaceae | H | N | N | Blow, punch | IPEC |
| Merétika et al. 2010 | Atlantic Forest | *Polygala paniculata* L. | Polygalaceae | H | N | N | Blow, punch | IPEC |
| Merétika et al. 2010 | Atlantic Forest | *Polygala paniculata* L. | Polygalaceae | H | N | N | Blow, punch | IPEC |
| Merétika et al. 2010 | Atlantic Forest | *Polygala paniculata* L. | Polygalaceae | H | N | N | Blow, punch | IPEC |
| Merétika et al. 2010 | Atlantic Forest | *Polygala paniculata* L. | Polygalaceae | H | N | N | Pain | SSNEC |
| Merétika et al. 2010 | Atlantic Forest | *Polygala paniculata* L. | Polygalaceae | H | N | N | Pain | SSNEC |
| Merétika et al. 2010 | Atlantic Forest | *Polygala paniculata* L. | Polygalaceae | H | N | N | Rheumatism | DMS |
| Merétika et al. 2010 | Atlantic Forest | *Polygala paniculata* L. | Polygalaceae | H | N | N | Rheumatism | DMS |
| Merétika et al. 2010 | Atlantic Forest | *Porophyllum ruderale* (Jacq.) Cass. | Asteraceae | H | N | E | Inflammation | SSNEC |
| Merétika et al. 2010 | Atlantic Forest | *Porophyllum ruderale* (Jacq.) Cass. | Asteraceae | H | N | E | Inflammation | SSNEC |
| Merétika et al. 2010 | Atlantic Forest | *Porophyllum ruderale* (Jacq.) Cass. | Asteraceae | H | N | E | Rheumatism | DMS |
| Merétika et al. 2010 | Atlantic Forest | *Porophyllum ruderale* (Jacq.) Cass. | Asteraceae | H | N | E | Rheumatism | DMS |
| Merétika et al. 2010 | Atlantic Forest | *Porophyllum ruderale* (Jacq.) Cass. | Asteraceae | H | N | E | Swelling | SSNEC |
| Merétika et al. 2010 | Atlantic Forest | *Porophyllum ruderale* (Jacq.) Cass. | Asteraceae | H | N | E | Swelling | SSNEC |
| Merétika et al. 2010 | Atlantic Forest | *Psidium guajava* L. | Myrtaceae | S | E | E | Helminthiasis | CIPD |
| Merétika et al. 2010 | Atlantic Forest | *Psidium guajava* L. | Myrtaceae | S | E | E | Stomach pain/Diarrhea | SSNEC |
| Merétika et al. 2010 | Atlantic Forest | *Psidium guajava* L. | Myrtaceae | S | E | E | Stomach pain/Diarrhea | SSNEC |
| Merétika et al. 2010 | Atlantic Forest | *Richardia brasiliensis* Gomes | Rubiaceae | H | N | N | Bronchitis | DRS |
| Merétika et al. 2010 | Atlantic Forest | *Richardia brasiliensis* Gomes | Rubiaceae | H | N | N | Bronchitis | DRS |
| Merétika et al. 2010 | Atlantic Forest | *Richardia brasiliensis* Gomes | Rubiaceae | H | N | N | Influenza | DRS |
| Merétika et al. 2010 | Atlantic Forest | *Richardia brasiliensis* Gomes | Rubiaceae | H | N | N | Influenza | DRS |
| Merétika et al. 2010 | Atlantic Forest | *Ruta graveolens* L. | Rutaceae | H | E | E | Cough | SSNEC |
| Merétika et al. 2010 | Atlantic Forest | *Ruta graveolens* L. | Rutaceae | H | E | E | Earache | DEMP |
| Merétika et al. 2010 | Atlantic Forest | *Ruta graveolens* L. | Rutaceae | H | E | E | Helminthiasis | CIPD |
| Merétika et al. 2010 | Atlantic Forest | *Sambucus australis* Cham. & Schltdl. | Adoxaceae | T | N | N | Allergy | IPEC |
| Merétika et al. 2010 | Atlantic Forest | *Sambucus australis* Cham. & Schltdl. | Adoxaceae | T | N | N | Cough | SSNEC |
| Merétika et al. 2010 | Atlantic Forest | *Sambucus australis* Cham. & Schltdl. | Adoxaceae | T | N | N | Depurative | DBBO |
| Merétika et al. 2010 | Atlantic Forest | *Schinus terebinthifolius* Raddi | Anacardiaceae | T | N | N | Inflammation | SSNEC |
| Merétika et al. 2010 | Atlantic Forest | *Schinus terebinthifolius* Raddi | Anacardiaceae | T | N | N | Inflammation | SSNEC |
| Merétika et al. 2010 | Atlantic Forest | *Schinus terebinthifolius* Raddi | Anacardiaceae | T | N | N | Leg pain | DMS |
| Merétika et al. 2010 | Atlantic Forest | *Schinus terebinthifolius* Raddi | Anacardiaceae | T | N | N | Leg pain | DMS |
| Merétika et al. 2010 | Atlantic Forest | *Schinus terebinthifolius* Raddi | Anacardiaceae | T | N | N | Toothache | DDS |
| Merétika et al. 2010 | Atlantic Forest | *Schinus terebinthifolius* Raddi | Anacardiaceae | T | N | N | Toothache | DDS |
| Merétika et al. 2010 | Atlantic Forest | *Sechium edule* (Jacq.) Sw. | Cucurbitaceae | H | E | E | High blood pressure | DCS |
| Merétika et al. 2010 | Atlantic Forest | *Sechium edule* (Jacq.) Sw. | Cucurbitaceae | H | E | E | High blood pressure | DCS |
| Merétika et al. 2010 | Atlantic Forest | *Sechium edule* (Jacq.) Sw. | Cucurbitaceae | H | E | E | Hypertension | DCS |
| Merétika et al. 2010 | Atlantic Forest | *Sechium edule* (Jacq.) Sw. | Cucurbitaceae | H | E | E | Hypertension | DCS |
| Merétika et al. 2010 | Atlantic Forest | *Sida rhombifolia* L. | Malvaceae | H | N | N | Depurative | DBBO |
| Merétika et al. 2010 | Atlantic Forest | *Sida rhombifolia* L. | Malvaceae | H | N | N | Fatigue | SSNEC |
| Merétika et al. 2010 | Atlantic Forest | *Smilax brasiliensis* Spreng. | Smilacaceae | S | N | E | Depurative | DBBO |
| Merétika et al. 2010 | Atlantic Forest | *Smilax brasiliensis* Spreng. | Smilacaceae | S | N | E | Helminthiasis | CIPD |
| Merétika et al. 2010 | Atlantic Forest | *Smilax brasiliensis* Spreng. | Smilacaceae | S | N | E | Leg pain | DMS |
| Merétika et al. 2010 | Atlantic Forest | *Solanum americanum* Mill. | Solanaceae | H | N | N | Helminthiasis | CIPD |
| Merétika et al. 2010 | Atlantic Forest | *Solidago chilensis* Meyen | Asteraceae | H | N | N | Back pain | DMS |
| Merétika et al. 2010 | Atlantic Forest | *Solidago chilensis* Meyen | Asteraceae | H | N | N | Back pain | DMS |
| Merétika et al. 2010 | Atlantic Forest | *Solidago chilensis* Meyen | Asteraceae | H | N | N | Muscle pain | DMS |
| Merétika et al. 2010 | Atlantic Forest | *Solidago chilensis* Meyen | Asteraceae | H | N | N | Muscle pain | DMS |
| Merétika et al. 2010 | Atlantic Forest | *Solidago chilensis* Meyen | Asteraceae | H | N | N | Rheumatism | DMS |
| Merétika et al. 2010 | Atlantic Forest | *Solidago chilensis* Meyen | Asteraceae | H | N | N | Rheumatism | DMS |
| Merétika et al. 2010 | Atlantic Forest | *Sphagneticola trilobata* (L.) Pruski | Asteraceae | H | N | N | Blow, punch | IPEC |
| Merétika et al. 2010 | Atlantic Forest | *Sphagneticola trilobata* (L.) Pruski | Asteraceae | H | N | N | Blow, punch | IPEC |
| Merétika et al. 2010 | Atlantic Forest | *Sphagneticola trilobata* (L.) Pruski | Asteraceae | H | N | N | Insect bite | IPEC |
| Merétika et al. 2010 | Atlantic Forest | *Sphagneticola trilobata* (L.) Pruski | Asteraceae | H | N | N | Insect bite | IPEC |
| Merétika et al. 2010 | Atlantic Forest | *Sphagneticola trilobata* (L.) Pruski | Asteraceae | H | N | N | Pain | SSNEC |
| Merétika et al. 2010 | Atlantic Forest | *Sphagneticola trilobata* (L.) Pruski | Asteraceae | H | N | N | Pain | SSNEC |
| Merétika et al. 2010 | Atlantic Forest | *Sphagneticola trilobata* (L.) Pruski | Asteraceae | H | N | N | Rheumatism | DMS |
| Merétika et al. 2010 | Atlantic Forest | *Sphagneticola trilobata* (L.) Pruski | Asteraceae | H | N | N | Rheumatism | DMS |
| Merétika et al. 2010 | Atlantic Forest | *Stachytarpheta cayennensis* (Rich.) Vahl | Verbenaceae | S | N | N | Cancer | NEO |
| Merétika et al. 2010 | Atlantic Forest | *Stachytarpheta cayennensis* (Rich.) Vahl | Verbenaceae | S | N | N | High cholesterol | ENMD |
| Merétika et al. 2010 | Atlantic Forest | *Stachytarpheta cayennensis* (Rich.) Vahl | Verbenaceae | S | N | N | Liver problems | DDS |
| Merétika et al. 2010 | Atlantic Forest | *Stachytarpheta cayennensis* (Rich.) Vahl | Verbenaceae | S | N | N | Stomach pain/Diarrhea | SSNEC |
| Merétika et al. 2010 | Atlantic Forest | *Symphytum* officinale L. | Boraginaceae | H | E | E | Cancer | NEO |
| Merétika et al. 2010 | Atlantic Forest | *Symphytum* officinale L. | Boraginaceae | H | E | E | Infection | CIPD |
| Merétika et al. 2010 | Atlantic Forest | *Symphytum* officinale L. | Boraginaceae | H | E | E | Stomach pain/Diarrhea | SSNEC |
| Merétika et al. 2010 | Atlantic Forest | *Symphytum* officinale L. | Boraginaceae | H | E | E | Wound | IPEC |
| Merétika et al. 2010 | Atlantic Forest | *Syzygium cumini* (L.) Skeels | Myrtaceae | T | E | E | Diabetes | ENMD |
| Merétika et al. 2010 | Atlantic Forest | *Syzygium cumini* (L.) Skeels | Myrtaceae | T | E | E | High cholesterol | ENMD |
| Merétika et al. 2010 | Atlantic Forest | *Tanacetum vulgare* L. | Asteraceae | H | E | E | Infection | CIPD |
| Merétika et al. 2010 | Atlantic Forest | *Tanacetum vulgare* L. | Asteraceae | H | E | E | Infection | CIPD |
| Merétika et al. 2010 | Atlantic Forest | *Tanacetum vulgare* L. | Asteraceae | H | E | E | Pain | SSNEC |
| Merétika et al. 2010 | Atlantic Forest | *Tanacetum vulgare* L. | Asteraceae | H | E | E | Pain | SSNEC |
| Merétika et al. 2010 | Atlantic Forest | *Tanacetum vulgare* L. | Asteraceae | H | E | E | Stomach problems | DDS |
| Merétika et al. 2010 | Atlantic Forest | *Tanacetum vulgare* L. | Asteraceae | H | E | E | Stomach problems | DDS |
| Merétika et al. 2010 | Atlantic Forest | *Terminalia catappa* L. | Combretaceae | T | E | E | Diabetes | ENMD |
| Merétika et al. 2010 | Atlantic Forest | *Terminalia catappa* L. | Combretaceae | T | E | E | High cholesterol | ENMD |
| Merétika et al. 2010 | Atlantic Forest | *Varronia curassavica* Jacq. | Boraginaceae | S | N | N | Back pain | DMS |
| Merétika et al. 2010 | Atlantic Forest | *Varronia curassavica* Jacq. | Boraginaceae | S | N | N | Inflammation | SSNEC |
| Merétika et al. 2010 | Atlantic Forest | *Varronia curassavica* Jacq. | Boraginaceae | S | N | N | Leg pain | DMS |
| Merétika et al. 2010 | Atlantic Forest | *Varronia curassavica* Jacq. | Boraginaceae | S | N | N | Pain | SSNEC |
| Merétika et al. 2010 | Atlantic Forest | *Varronia curassavica* Jacq. | Boraginaceae | S | N | N | Rheumatism | DMS |
| Merétika et al. 2010 | Atlantic Forest | *Verbena litoralis* Kunth | Verbenaceae | H | N | N | High cholesterol | ENMD |
| Merétika et al. 2010 | Atlantic Forest | *Verbena litoralis* Kunth | Verbenaceae | H | N | N | Leukemia | NEO |
| Merétika et al. 2010 | Atlantic Forest | *Verbena litoralis* Kunth | Verbenaceae | H | N | N | Liver problems | DDS |
| Merétika et al. 2010 | Atlantic Forest | *Verbena litoralis* Kunth | Verbenaceae | H | N | N | Stomach pain/Diarrhea | SSNEC |
| Merétika et al. 2010 | Atlantic Forest | *Verbena litoralis* Kunth | Verbenaceae | H | N | N | Stomach problems | DDS |
| Merétika et al. 2010 | Atlantic Forest | *Vernonanthura phosphorica* (Vell.) H.Rob. | Asteraceae | S | N | N | Inflammation | SSNEC |
| Merétika et al. 2010 | Atlantic Forest | *Vernonanthura phosphorica* (Vell.) H.Rob. | Asteraceae | S | N | N | Inflammation | SSNEC |
| Merétika et al. 2010 | Atlantic Forest | *Vernonanthura phosphorica* (Vell.) H.Rob. | Asteraceae | S | N | N | Leg pain | DMS |
| Merétika et al. 2010 | Atlantic Forest | *Vernonanthura phosphorica* (Vell.) H.Rob. | Asteraceae | S | N | N | Leg pain | DMS |
| Merétika et al. 2010 | Atlantic Forest | *Vernonanthura phosphorica* (Vell.) H.Rob. | Asteraceae | S | N | N | Rheumatism | DMS |
| Merétika et al. 2010 | Atlantic Forest | *Vernonanthura phosphorica* (Vell.) H.Rob. | Asteraceae | S | N | N | Rheumatism | DMS |
| Moreira & Guarim-Neto 2009 | Cerrado | *Acanthospermum hispidum* DC. | Asteraceae | H | N | N | Diabetes | ENMD |
| Moreira & Guarim-Neto 2009 | Cerrado | *Achyrocline satureioides* (Lam.) DC. | Asteraceae | S | N | N | Anti-emetic | SSNEC |
| Moreira & Guarim-Neto 2009 | Cerrado | *Achyrocline satureioides* (Lam.) DC. | Asteraceae | S | N | N | Stomach pain/Diarrhea | SSNEC |
| Moreira & Guarim-Neto 2009 | Cerrado | *Acrocomia aculeata* (Jacq.) Lodd. ex Mart. | Arecaceae | T | N | N | Bronchitis | DRS |
| Moreira & Guarim-Neto 2009 | Cerrado | *Alibertia edulis* (Rich.) A.Rich. | Rubiaceae | S | N | N | Influenza | DRS |
| Moreira & Guarim-Neto 2009 | Cerrado | *Amburana cearensis* (Allemão) A.C.Sm. | Fabaceae | T | N | N | Pneumonia | DRS |
| Moreira & Guarim-Neto 2009 | Cerrado | *Anacardium humile* A.St.-Hil. | Anacardiaceae | T | N | N | Diabetes | ENMD |
| Moreira & Guarim-Neto 2009 | Cerrado | *Anacardium humile* A.St.-Hil. | Anacardiaceae | T | N | N | Inflammation | SSNEC |
| Moreira & Guarim-Neto 2009 | Cerrado | *Anadenanthera peregrina* (L.) Speg. | Fabaceae | T | N | N | Bronchitis | DRS |
| Moreira & Guarim-Neto 2009 | Cerrado | *Ananas comosus* (L.) Merril | Bromeliaceae | H | N | N | Bronchitis | DRS |
| Moreira & Guarim-Neto 2009 | Cerrado | *Andira cujabensis* Benth. | Fabaceae | T | N | N | Helminthiasis | CIPD |
| Moreira & Guarim-Neto 2009 | Cerrado | *Anemopaegma arvense* (Vell.) Stellfeld ex de Souza | Bignoniaceae | H | N | N | Cardiac problems | DCS |
| Moreira & Guarim-Neto 2009 | Cerrado | *Anemopaegma arvense* (Vell.) Stellfeld ex de Souza | Bignoniaceae | H | N | N | Hypertension | DCS |
| Moreira & Guarim-Neto 2009 | Cerrado | *Apeiba tibourbou* Aubl. | Malvaceae | T | N | N | Muscle pain | DMS |
| Moreira & Guarim-Neto 2009 | Cerrado | *Aristolochia holostylis* F.González | Aristolochiaceae | H | N | N | Laxative | DDS |
| Moreira & Guarim-Neto 2009 | Cerrado | *Aristolochia holostylis* F.González | Aristolochiaceae | H | N | N | Liver problems | DDS |
| Moreira & Guarim-Neto 2009 | Cerrado | *Aristolochia holostylis* F.González | Aristolochiaceae | H | N | N | Stomach problems | DDS |
| Moreira & Guarim-Neto 2009 | Cerrado | *Aristolochia holostylis* F.González | Aristolochiaceae | H | N | N | Stroke | DCS |
| Moreira & Guarim-Neto 2009 | Cerrado | *Astronium fraxinifolium* Schott | Anacardiaceae | T | N | N | Influenza | DRS |
| Moreira & Guarim-Neto 2009 | Cerrado | *Attalea speciosa* Mart. ex Spreng. | Arecaceae | T | N | N | Anemia | DBBO |
| Moreira & Guarim-Neto 2009 | Cerrado | *Bauhinia rufa* (Bong.) Steud. | Fabaceae | T | N | N | Diabetes | ENMD |
| Moreira & Guarim-Neto 2009 | Cerrado | *Bauhinia rufa* (Bong.) Steud. | Fabaceae | T | N | N | Diabetes | ENMD |
| Moreira & Guarim-Neto 2009 | Cerrado | *Bromelia balansae* Mez | Bromeliaceae | H | N | N | Bronchitis | DRS |
| Moreira & Guarim-Neto 2009 | Cerrado | *Bromelia balansae* Mez | Bromeliaceae | H | N | N | Bronchitis | DRS |
| Moreira & Guarim-Neto 2009 | Cerrado | *Bromelia balansae* Mez | Bromeliaceae | H | N | N | Cough | SSNEC |
| Moreira & Guarim-Neto 2009 | Cerrado | *Bromelia balansae* Mez | Bromeliaceae | H | N | N | Cough | SSNEC |
| Moreira & Guarim-Neto 2009 | Cerrado | *Brosimum gaudichaudii* Trécul | Moraceae | T | N | N | Depurative | DBBO |
| Moreira & Guarim-Neto 2009 | Cerrado | *Brosimum gaudichaudii* Trécul | Moraceae | T | N | N | Depurative | DBBO |
| Moreira & Guarim-Neto 2009 | Cerrado | *Brosimum gaudichaudii* Trécul | Moraceae | T | N | N | Kidney problems | DGS |
| Moreira & Guarim-Neto 2009 | Cerrado | *Brosimum gaudichaudii* Trécul | Moraceae | T | N | N | Kidney problems | DGS |
| Moreira & Guarim-Neto 2009 | Cerrado | *Bulbostylis paradoxa* (Spreng.) Lindm. | Cyperaceae | H | N | N | Weakness in the joints | DMS |
| Moreira & Guarim-Neto 2009 | Cerrado | *Byrsonima coccolobifolia* Kunth | Malpighiaceae | T | N | N | Wound | IPEC |
| Moreira & Guarim-Neto 2009 | Cerrado | *Byrsonima pachyphylla* A. Juss. | Malpighiaceae | T | N | N | Wound | IPEC |
| Moreira & Guarim-Neto 2009 | Cerrado | Byttneria melastomaefolia A.St.-Hil. | Malvaceae | H | N | N | Rheumatism | DMS |
| Moreira & Guarim-Neto 2009 | Cerrado | Byttneria melastomaefolia A.St.-Hil. | Malvaceae | H | N | N | Throat infection | DRS |
| Moreira & Guarim-Neto 2009 | Cerrado | *Calliandra parvifolia* (Hook. & Arn.) Speg. | Fabaceae | S | N | N | Fever | SSNEC |
| Moreira & Guarim-Neto 2009 | Cerrado | *Callisthene fasciculata* Mart. | Vochysiaceae | T | N | N | Hepatitis | CIPD |
| Moreira & Guarim-Neto 2009 | Cerrado | *Callisthene fasciculata* Mart. | Vochysiaceae | T | N | N | Hepatitis | CIPD |
| Moreira & Guarim-Neto 2009 | Cerrado | *Callisthene fasciculata* Mart. | Vochysiaceae | T | N | N | Intestinal fever leading to dysentry | SSNEC |
| Moreira & Guarim-Neto 2009 | Cerrado | *Callisthene fasciculata* Mart. | Vochysiaceae | T | N | N | Intestinal fever leading to dysentry | SSNEC |
| Moreira & Guarim-Neto 2009 | Cerrado | *Camarea ericoides* A.St.-Hil. | Malpighiaceae | H | N | N | Depurative | DBBO |
| Moreira & Guarim-Neto 2009 | Cerrado | *Camarea ericoides* A.St.-Hil. | Malpighiaceae | H | N | N | Infection | CIPD |
| Moreira & Guarim-Neto 2009 | Cerrado | *Camarea ericoides* A.St.-Hil. | Malpighiaceae | H | N | N | Injury | IPEC |
| Moreira & Guarim-Neto 2009 | Cerrado | *Camarea ericoides* A.St.-Hil. | Malpighiaceae | H | N | N | Muscle pain | DMS |
| Moreira & Guarim-Neto 2009 | Cerrado | *Cariniana estrellensis* (Raddi) Kuntze | Lecythidaceae | T | N | N | Inflammation | SSNEC |
| Moreira & Guarim-Neto 2009 | Cerrado | *Cariniana estrellensis* (Raddi) Kuntze | Lecythidaceae | T | N | N | Inflammation | SSNEC |
| Moreira & Guarim-Neto 2009 | Cerrado | *Cariniana estrellensis* (Raddi) Kuntze | Lecythidaceae | T | N | N | Muscle pain | DMS |
| Moreira & Guarim-Neto 2009 | Cerrado | *Cariniana estrellensis* (Raddi) Kuntze | Lecythidaceae | T | N | N | Muscle pain | DMS |
| Moreira & Guarim-Neto 2009 | Cerrado | *Caryocar brasiliense* Cambess. | Caryocaraceae | T | N | N | Inflammation | SSNEC |
| Moreira & Guarim-Neto 2009 | Cerrado | *Casearia sylvestris* Sw. | Salicaceae | T | N | N | Anemia | DBBO |
| Moreira & Guarim-Neto 2009 | Cerrado | *Cassytha filiformis* L. | Lauraceae | H | N | N | Influenza | DRS |
| Moreira & Guarim-Neto 2009 | Cerrado | *Cecropia polystachya* Trécul | Urticaceae | T | N | E | Diabetes | ENMD |
| Moreira & Guarim-Neto 2009 | Cerrado | *Cecropia polystachya* Trécul | Urticaceae | T | N | E | Diabetes | ENMD |
| Moreira & Guarim-Neto 2009 | Cerrado | *Cecropia polystachya* Trécul | Urticaceae | T | N | E | Muscle pain | DMS |
| Moreira & Guarim-Neto 2009 | Cerrado | *Cecropia polystachya* Trécul | Urticaceae | T | N | E | Muscle pain | DMS |
| Moreira & Guarim-Neto 2009 | Cerrado | *Cecropia polystachya* Trécul | Urticaceae | T | N | E | Pain in spinal column | DMS |
| Moreira & Guarim-Neto 2009 | Cerrado | *Cecropia polystachya* Trécul | Urticaceae | T | N | E | Pain in spinal column | DMS |
| Moreira & Guarim-Neto 2009 | Cerrado | *Cecropia polystachya* Trécul | Urticaceae | T | N | E | Wound | IPEC |
| Moreira & Guarim-Neto 2009 | Cerrado | *Cecropia polystachya* Trécul | Urticaceae | T | N | E | Wound | IPEC |
| Moreira & Guarim-Neto 2009 | Cerrado | *Centaurium erythraea* Rafn | Gentianaceae | H | E | E | Kidney problems | DGS |
| Moreira & Guarim-Neto 2009 | Cerrado | *Chamaecrista desvauxii* (Collad.) Killip | Fabaceae | S | N | N | Menopause | ENMD |
| Moreira & Guarim-Neto 2009 | Cerrado | *Chamaecrista desvauxii* (Collad.) Killip | Fabaceae | S | N | N | PMT | DGS |
| Moreira & Guarim-Neto 2009 | Cerrado | *Cissampelos ovalifolia* DC. | Menispermaceae | S | N | N | Stomach problems | DDS |
| Moreira & Guarim-Neto 2009 | Cerrado | *Cissus verticillata* (L.) Nicolson & C.E.Jarvis | Vitaceae | S | N | N | Diabetes | ENMD |
| Moreira & Guarim-Neto 2009 | Cerrado | *Cochlospermum regium* (Mart. ex Schrank) Pilg. | Bixaceae | S | N | N | Inflammation | SSNEC |
| Moreira & Guarim-Neto 2009 | Cerrado | *Cochlospermum regium* (Mart. ex Schrank) Pilg. | Bixaceae | S | N | N | Prostate problems | DGS |
| Moreira & Guarim-Neto 2009 | Cerrado | *Copaifera langsPainffii Desf.* | Fabaceae | T | N | N | Bronchitis | DRS |
| Moreira & Guarim-Neto 2009 | Cerrado | *Copaifera langsPainffii Desf.* | Fabaceae | T | N | N | Bronchitis | DRS |
| Moreira & Guarim-Neto 2009 | Cerrado | *Copaifera langsPainffii Desf.* | Fabaceae | T | N | N | Influenza | DRS |
| Moreira & Guarim-Neto 2009 | Cerrado | *Copaifera langsPainffii Desf.* | Fabaceae | T | N | N | Influenza | DRS |
| Moreira & Guarim-Neto 2009 | Cerrado | *Copaifera langsPainffii Desf.* | Fabaceae | T | N | N | Problems with spinal column | DMS |
| Moreira & Guarim-Neto 2009 | Cerrado | *Copaifera langsPainffii Desf.* | Fabaceae | T | N | N | Problems with spinal column | DMS |
| Moreira & Guarim-Neto 2009 | Cerrado | *Copernicia alba* Morong ex Morong & Britton | Arecaceae | T | N | N | Stomach pain/Diarrhea | SSNEC |
| Moreira & Guarim-Neto 2009 | Cerrado | *Cordia insignis* Cham. | Boraginaceae | S | N | N | Pain | SSNEC |
| Moreira & Guarim-Neto 2009 | Cerrado | *Cordia insignis* Cham. | Boraginaceae | S | N | N | Rheumatism | DMS |
| Moreira & Guarim-Neto 2009 | Cerrado | *Cordiera macrophylla* (K.Schum.) Kuntze | Rubiaceae | T | N | N | Kidney problems | DGS |
| Moreira & Guarim-Neto 2009 | Cerrado | *Costus arabicus* L. | Costaceae | H | N | E | Kidney problems | DGS |
| Moreira & Guarim-Neto 2009 | Cerrado | *Craniolaria integrifolia* Cham. | Martyniaceae | H | N | E | Snake bite | IPEC |
| Moreira & Guarim-Neto 2009 | Cerrado | *Croton antisyphiliticus* Mart. | Euphorbiaceae | S | N | N | Kidney problems | DGS |
| Moreira & Guarim-Neto 2009 | Cerrado | *Croton urucurana* Baill. | Euphorbiaceae | T | N | N | Inflammation | SSNEC |
| Moreira & Guarim-Neto 2009 | Cerrado | *Curatella americana* L. | Dilleniaceae | T | N | N | Severe diarrhoea | CIPD |
| Moreira & Guarim-Neto 2009 | Cerrado | *Curatella americana* L. | Dilleniaceae | T | N | N | Severe diarrhoea | CIPD |
| Moreira & Guarim-Neto 2009 | Cerrado | *Curatella americana* L. | Dilleniaceae | T | N | N | Stomach pain/Diarrhea | SSNEC |
| Moreira & Guarim-Neto 2009 | Cerrado | *Curatella americana* L. | Dilleniaceae | T | N | N | Stomach pain/Diarrhea | SSNEC |
| Moreira & Guarim-Neto 2009 | Cerrado | *Curatella americana* L. | Dilleniaceae | T | N | N | Wound | IPEC |
| Moreira & Guarim-Neto 2009 | Cerrado | *Curatella americana* L. | Dilleniaceae | T | N | N | Wound | IPEC |
| Moreira & Guarim-Neto 2009 | Cerrado | *Cybistax antisyphilitica* (Mart.) Mart. | Bignoniaceae | T | N | N | Headache | SSNEC |
| Moreira & Guarim-Neto 2009 | Cerrado | *Davilla elliptica* A.St.-Hil. | Dilleniaceae | S | N | N | Hernia | DDS |
| Moreira & Guarim-Neto 2009 | Cerrado | *Davilla elliptica* A.St.-Hil. | Dilleniaceae | S | N | N | Wound | IPEC |
| Moreira & Guarim-Neto 2009 | Cerrado | *Dipteryx alata* Vogel | Fabaceae | T | N | N | Stomach pain/Diarrhea | SSNEC |
| Moreira & Guarim-Neto 2009 | Cerrado | *Dipteryx alata* Vogel | Fabaceae | T | N | N | Wound | IPEC |
| Moreira & Guarim-Neto 2009 | Cerrado | *Discocactus heptacanthus* (Rodrigues) Britton & Rose | Cactaceae | S | N | N | Swollen lymph nodes | DCS |
| Moreira & Guarim-Neto 2009 | Cerrado | *EchinoPainus grandiflorus (Cham. & Schltr.) Micheli* | Alismataceae | H | N | N | Kidney problems | DGS |
| Moreira & Guarim-Neto 2009 | Cerrado | *Enterolobium contortisiliquum* (Vell.) Morong | Fabaceae | T | N | N | Hemorrhoids | DCS |
| Moreira & Guarim-Neto 2009 | Cerrado | *Euphorbia hyssopifolia* L. | Euphorbiaceae | H | N | N | Anticoagulant | DCS |
| Moreira & Guarim-Neto 2009 | Cerrado | *Gallesia integrifolia* (Spreng.) Harms | Phytolaccaceae | T | N | N | Stroke | DCS |
| Moreira & Guarim-Neto 2009 | Cerrado | *Garcinia brasiliensis* Mart. | Clusiaceae | T | N | N | Bronchitis | DRS |
| Moreira & Guarim-Neto 2009 | Cerrado | *Gomphrena arborescens* L.f. | Amaranthaceae | H | N | N | Dental care | DDS |
| Moreira & Guarim-Neto 2009 | Cerrado | *Guarea guidonia* (L.) Sleumer | Meliaceae | T | N | N | Fortifier | SSNEC |
| Moreira & Guarim-Neto 2009 | Cerrado | *Guazuma ulmifolia* Lam. | Malvaceae | T | N | N | Burns | IPEC |
| Moreira & Guarim-Neto 2009 | Cerrado | *Guazuma ulmifolia* Lam. | Malvaceae | T | N | N | Wound | IPEC |
| Moreira & Guarim-Neto 2009 | Cerrado | *Guettarda viburnoides* Cham. & Schltdl. | Rubiaceae | T | N | N | Hip pain | DMS |
| Moreira & Guarim-Neto 2009 | Cerrado | *Guettarda viburnoides* Cham. & Schltdl. | Rubiaceae | T | N | N | Kidney problems | DGS |
| Moreira & Guarim-Neto 2009 | Cerrado | *Hancornia speciosa* Gomes | Apocynaceae | T | N | N | Diabetes | ENMD |
| Moreira & Guarim-Neto 2009 | Cerrado | *Hancornia speciosa* Gomes | Apocynaceae | T | N | N | Hernia | DDS |
| Moreira & Guarim-Neto 2009 | Cerrado | *Handroanthus impetiginosus* Mattos | Bignoniaceae | T | N | N | Cardiac problems | DCS |
| Moreira & Guarim-Neto 2009 | Cerrado | *Handroanthus ochraceus* (Cham.) Mattos | Bignoniaceae | T | N | N | Cough | SSNEC |
| Moreira & Guarim-Neto 2009 | Cerrado | *Handroanthus ochraceus* (Cham.) Mattos | Bignoniaceae | T | N | N | Fever | SSNEC |
| Moreira & Guarim-Neto 2009 | Cerrado | *Helicteres sacarolha* A.St.-Hil. et al. | Malvaceae | S | N | N | Depurative | DBBO |
| Moreira & Guarim-Neto 2009 | Cerrado | *Helicteres sacarolha* A.St.-Hil. et al. | Malvaceae | S | N | N | Postpartum rest | PCP |
| Moreira & Guarim-Neto 2009 | Cerrado | *Herreria salsaparilha* Mart. | Agavaceae | S | N | N | Depurative | DBBO |
| Moreira & Guarim-Neto 2009 | Cerrado | *Heteropterys tomentosa* A.Juss. | Malpighiaceae | S | N | N | Kidney problems | DGS |
| Moreira & Guarim-Neto 2009 | Cerrado | *Himatanthus obovatus* (Müll.Arg.) Woodson | Apocynaceae | T | N | N | Depurative | DBBO |
| Moreira & Guarim-Neto 2009 | Cerrado | *Hymenaea stigonocarpa* Mart. ex Hayne | Fabaceae | T | N | N | Bronchitis | DRS |
| Moreira & Guarim-Neto 2009 | Cerrado | *Hymenaea stigonocarpa* Mart. ex Hayne | Fabaceae | T | N | N | Influenza | DRS |
| Moreira & Guarim-Neto 2009 | Cerrado | *Hyptidendron canum* (Pohl ex Benth.) Harley | Lamiaceae | T | N | N | Helminthiasis | CIPD |
| Moreira & Guarim-Neto 2009 | Cerrado | *Hyptidendron canum* (Pohl ex Benth.) Harley | Lamiaceae | T | N | N | Influenza | DRS |
| Moreira & Guarim-Neto 2009 | Cerrado | *Hyptis suaveolens* Poit. | Lamiaceae | H | N | N | Hemorrhoids | DCS |
| Moreira & Guarim-Neto 2009 | Cerrado | *Hyptis suaveolens* Poit. | Lamiaceae | H | N | N | Hemorrhoids | DCS |
| Moreira & Guarim-Neto 2009 | Cerrado | *Imperata brasiliensis* Trin. | Poaceae | H | N | N | Intestinal fever leading to dysentry | SSNEC |
| Moreira & Guarim-Neto 2009 | Cerrado | *Jacaranda cuspidifolia* Mart. | Bignoniaceae | T | N | N | Inflammation | SSNEC |
| Moreira & Guarim-Neto 2009 | Cerrado | *Jacaranda cuspidifolia* Mart. | Bignoniaceae | T | N | N | Inflammation | SSNEC |
| Moreira & Guarim-Neto 2009 | Cerrado | *Jacaranda cuspidifolia* Mart. | Bignoniaceae | T | N | N | Vaginal discharge | DGS |
| Moreira & Guarim-Neto 2009 | Cerrado | *Jacaranda cuspidifolia* Mart. | Bignoniaceae | T | N | N | Vaginal discharge | DGS |
| Moreira & Guarim-Neto 2009 | Cerrado | *Jacaranda decurrens* Cham. | Bignoniaceae | S | N | N | Depurative | DBBO |
| Moreira & Guarim-Neto 2009 | Cerrado | *Jatropha elliptica* (Pohl) Oken | Euphorbiaceae | H | N | N | Laxative | DDS |
| Moreira & Guarim-Neto 2009 | Cerrado | *Lafoensia pacari* A.St.-Hil. | Lythraceae | T | N | N | Stomach problems | DDS |
| Moreira & Guarim-Neto 2009 | Cerrado | *Leptolobium elegans* Vogel | Fabaceae | T | N | N | Depurative | DBBO |
| Moreira & Guarim-Neto 2009 | Cerrado | *Mandevilla velame* (A.St.-Hil.) Pichon | Apocynaceae | S | N | N | Depurative | DBBO |
| Moreira & Guarim-Neto 2009 | Cerrado | *Mauritia flexuosa* L.f. | Arecaceae | T | N | N | Kidney problems | DGS |
| Moreira & Guarim-Neto 2009 | Cerrado | *Myracrodruon urundeuva* Allemão | Anacardiaceae | T | N | N | Stomach pain/Diarrhea | SSNEC |
| Moreira & Guarim-Neto 2009 | Cerrado | *Myrcia albidotomentosa* (Amshoff) McVaugh | Myrtaceae | S | N | E | Toothache | DDS |
| Moreira & Guarim-Neto 2009 | Cerrado | *Orthopappus angustifolius* (Sw.) Gleason | Asteraceae | H | N | N | Influenza | DRS |
| Moreira & Guarim-Neto 2009 | Cerrado | *Oxalis hirsutissima* Mart. ex Zucc. | Oxalidaceae | H | N | N | Eye pain | DEA |
| Moreira & Guarim-Neto 2009 | Cerrado | *Oxalis hirsutissima* Mart. ex Zucc. | Oxalidaceae | H | N | N | Stomach pain/Diarrhea | SSNEC |
| Moreira & Guarim-Neto 2009 | Cerrado | *Painstenia asaroides Gardner ex Hook.* | Moraceae | H | N | E | Depurative | DBBO |
| Moreira & Guarim-Neto 2009 | Cerrado | *Painstenia asaroides Gardner ex Hook.* | Moraceae | H | N | E | Influenza | DRS |
| Moreira & Guarim-Neto 2009 | Cerrado | *Palicourea rigida* Kunth | Rubiaceae | S | N | N | Kidney problems | DGS |
| Moreira & Guarim-Neto 2009 | Cerrado | *Peixotoa hirta* A.Juss. | Malpighiaceae | S | N | N | Pain | SSNEC |
| Moreira & Guarim-Neto 2009 | Cerrado | *Peixotoa hirta* A.Juss. | Malpighiaceae | S | N | N | Rheumatism | DMS |
| Moreira & Guarim-Neto 2009 | Cerrado | *Phanera glabra* (Jacq.) Vaz | Fabaceae | S | N | N | Stomach pain/Diarrhea | SSNEC |
| Moreira & Guarim-Neto 2009 | Cerrado | *Phanera glabra* (Jacq.) Vaz | Fabaceae | S | N | N | Stomach pain/Diarrhea | SSNEC |
| Moreira & Guarim-Neto 2009 | Cerrado | *Phlebodium decumanum* (Willd.) J.Sm. | Polypodiaceae | H | N | N | Hepatitis | CIPD |
| Moreira & Guarim-Neto 2009 | Cerrado | *Phlebodium decumanum* (Willd.) J.Sm. | Polypodiaceae | H | N | N | Intestinal fever leading to dysentry | SSNEC |
| Moreira & Guarim-Neto 2009 | Cerrado | *Phlebodium decumanum* (Willd.) J.Sm. | Polypodiaceae | H | N | N | Jaundice | SSNEC |
| Moreira & Guarim-Neto 2009 | Cerrado | *Plathymenia reticulata* Benth. | Fabaceae | T | N | N | Hemorrhoids | DCS |
| Moreira & Guarim-Neto 2009 | Cerrado | *Plathymenia reticulata* Benth. | Fabaceae | T | N | N | Rheumatism | DMS |
| Moreira & Guarim-Neto 2009 | Cerrado | *Protium heptaphyllum* (Aubl.) Marchand | Burseraceae | T | N | N | Headache | SSNEC |
| Moreira & Guarim-Neto 2009 | Cerrado | *Protium heptaphyllum* (Aubl.) Marchand | Burseraceae | T | N | N | Influenza | DRS |
| Moreira & Guarim-Neto 2009 | Cerrado | *Protium heptaphyllum* (Aubl.) Marchand | Burseraceae | T | N | N | Severe diarrhoea | CIPD |
| Moreira & Guarim-Neto 2009 | Cerrado | *Pseudobombax longiflorum* (Mart. & Zucc.) A.Robyns | Malvaceae | T | N | N | Hemorrhoids | DCS |
| Moreira & Guarim-Neto 2009 | Cerrado | *Psidium firmum* O.Berg | Myrtaceae | S | N | N | Diabetes | ENMD |
| Moreira & Guarim-Neto 2009 | Cerrado | *Pterodon pubescens* (Benth.) Benth. | Fabaceae | T | N | N | Infection | CIPD |
| Moreira & Guarim-Neto 2009 | Cerrado | *Pterodon pubescens* (Benth.) Benth. | Fabaceae | T | N | N | Influenza | DRS |
| Moreira & Guarim-Neto 2009 | Cerrado | *Qualea grandiflora* Mart. | Vochysiaceae | T | N | N | Chilblains | CIPD |
| Moreira & Guarim-Neto 2009 | Cerrado | *Qualea parviflora* Mart. | Vochysiaceae | T | N | N | Insomnia | DNS |
| Moreira & Guarim-Neto 2009 | Cerrado | *Qualea parviflora* Mart. | Vochysiaceae | T | N | N | Insomnia | DNS |
| Moreira & Guarim-Neto 2009 | Cerrado | *Qualea parviflora* Mart. | Vochysiaceae | T | N | N | Stomach pain/Diarrhea | SSNEC |
| Moreira & Guarim-Neto 2009 | Cerrado | *Qualea parviflora* Mart. | Vochysiaceae | T | N | N | Stomach pain/Diarrhea | SSNEC |
| Moreira & Guarim-Neto 2009 | Cerrado | *Qualea parviflora* Mart. | Vochysiaceae | T | N | N | Stomach problems | DDS |
| Moreira & Guarim-Neto 2009 | Cerrado | *Qualea parviflora* Mart. | Vochysiaceae | T | N | N | Stomach problems | DDS |
| Moreira & Guarim-Neto 2009 | Cerrado | *Rudgea viburnoides* (Cham.) Benth. | Rubiaceae | T | N | N | Kidney problems | DGS |
| Moreira & Guarim-Neto 2009 | Cerrado | *Salacia elliptica* (Mart. ex Schult.) G.Don | Celastraceae | T | N | N | Diabetes | ENMD |
| Moreira & Guarim-Neto 2009 | Cerrado | *Salvertia convallarioPaina A.St.-Hil.* | Vochysiaceae | T | N | N | Diabetes | ENMD |
| Moreira & Guarim-Neto 2009 | Cerrado | *Salvertia convallarioPaina A.St.-Hil.* | Vochysiaceae | T | N | N | Diabetes | ENMD |
| Moreira & Guarim-Neto 2009 | Cerrado | *Serjania caracasana* (Jacq.) Willd. | Sapindaceae | S | N | N | Wound | IPEC |
| Moreira & Guarim-Neto 2009 | Cerrado | *Serjania erecta* Radlk. | Santalaceae | S | N | N | Depurative | DBBO |
| Moreira & Guarim-Neto 2009 | Cerrado | *Simaba ferruginea* A.St.-Hil. | Simaroubaceae | T | N | N | Helminthiasis | CIPD |
| Moreira & Guarim-Neto 2009 | Cerrado | *Simaba ferruginea* A.St.-Hil. | Simaroubaceae | T | N | N | Stomach problems | DDS |
| Moreira & Guarim-Neto 2009 | Cerrado | *Siparuna guianensis* Aubl. | Siparunaceae | T | N | N | Headache | SSNEC |
| Moreira & Guarim-Neto 2009 | Cerrado | *Smilax goyazana* A.DC. | Smilacaceae | S | N | N | Earache | DEMP |
| Moreira & Guarim-Neto 2009 | Cerrado | *Solanum lycocarpum* A.St.-Hil. | Solanaceae | S | N | N | Hemorrhoids | DCS |
| Moreira & Guarim-Neto 2009 | Cerrado | *Solanum lycocarpum* A.St.-Hil. | Solanaceae | S | N | N | Liver problems | DDS |
| Moreira & Guarim-Neto 2009 | Cerrado | *Solanum viarum* Dunal | Solanaceae | H | N | N | Erysipelas | CIPD |
| Moreira & Guarim-Neto 2009 | Cerrado | *Spiranthera oPainatissima A.St.-Hil.* | Rutaceae | S | N | N | Rheumatism | DMS |
| Moreira & Guarim-Neto 2009 | Cerrado | *Stachytarpheta cayennensis* (Rich.) Vahl | Verbenaceae | S | N | N | Liver problems | DDS |
| Moreira & Guarim-Neto 2009 | Cerrado | *Strychnos pseudoquina* A.St.-Hil. | Loganiaceae | T | N | N | Anemia | DBBO |
| Moreira & Guarim-Neto 2009 | Cerrado | *Strychnos pseudoquina* A.St.-Hil. | Loganiaceae | T | N | N | Hair loss | DSST |
| Moreira & Guarim-Neto 2009 | Cerrado | *Stryphnodendron aSTDringens (Mart.) Coville* | Fabaceae | T | N | N | Inflammation | SSNEC |
| Moreira & Guarim-Neto 2009 | Cerrado | *Stryphnodendron aSTDringens (Mart.) Coville* | Fabaceae | T | N | N | Inflammation | SSNEC |
| Moreira & Guarim-Neto 2009 | Cerrado | *Stryphnodendron aSTDringens (Mart.) Coville* | Fabaceae | T | N | N | Stomach pain/Diarrhea | SSNEC |
| Moreira & Guarim-Neto 2009 | Cerrado | *Stryphnodendron aSTDringens (Mart.) Coville* | Fabaceae | T | N | N | Stomach pain/Diarrhea | SSNEC |
| Moreira & Guarim-Neto 2009 | Cerrado | *Stryphnodendron aSTDringens (Mart.) Coville* | Fabaceae | T | N | N | Vaginal discharge | DGS |
| Moreira & Guarim-Neto 2009 | Cerrado | *Stryphnodendron aSTDringens (Mart.) Coville* | Fabaceae | T | N | N | Vaginal discharge | DGS |
| Moreira & Guarim-Neto 2009 | Cerrado | *Syagrus oleracea* (Mart.) Becc. | Arecaceae | T | N | N | Liver problems | DDS |
| Moreira & Guarim-Neto 2009 | Cerrado | *Taccarum weddellianum* Brongn. ex Schott | Araceae | H | N | N | Snake bite | IPEC |
| Moreira & Guarim-Neto 2009 | Cerrado | *Terminalia argentea* Mart. | Combretaceae | T | N | N | Stomach pain/Diarrhea | SSNEC |
| Moreira & Guarim-Neto 2009 | Cerrado | *Trimezia juncifolia* (Klatt) Benth. & Hook. | Iridaceae | H | N | N | Depurative | DBBO |
| Moreira & Guarim-Neto 2009 | Cerrado | *Vatairea macrocarpa* (Benth.) Ducke | Fabaceae | T | N | N | Rheumatism | DMS |
| Moreira & Guarim-Neto 2009 | Cerrado | *Vernonanthura ferruginea* (Less.) H.Rob. | Asteraceae | S | N | N | Influenza | DRS |
| Moreira & Guarim-Neto 2009 | Cerrado | *Vitex cymosa* Bertero ex Spreng. | Lamiaceae | T | N | N | Toothache | DDS |
| Moreira & Guarim-Neto 2009 | Cerrado | *Vitex cymosa* Bertero ex Spreng. | Lamiaceae | T | N | N | Toothache | DDS |
| Moreira & Guarim-Neto 2009 | Cerrado | *Vochysia divergens* Pohl | Vochysiaceae | T | N | N | Cough | SSNEC |
| Moreira & Guarim-Neto 2009 | Cerrado | *Vochysia divergens* Pohl | Vochysiaceae | T | N | N | Influenza | DRS |
| Moreira & Guarim-Neto 2009 | Cerrado | *Vochysia rufa* Mart. | Vochysiaceae | T | N | N | Eyewash | DEA |
| Moreira & Guarim-Neto 2009 | Cerrado | *Waltheria communis* A.St.-Hil. | Malvaceae | H | N | N | Antimicrobial | CIPD |
| Moreira & Guarim-Neto 2009 | Cerrado | *Waltheria communis* A.St.-Hil. | Malvaceae | H | N | N | Inflammation of the female organs | DGS |
| Moreira & Guarim-Neto 2009 | Cerrado | *Xylopia aromatica* (Lam.) Mart. | Annonaceae | T | N | N | Cardiac problems | DCS |
| Moreira & Guarim-Neto 2009 | Cerrado | *Zamia boliviana* (Brongn.) A.DC. | Zamiaceae | H | N | N | Stomach pain/Diarrhea | SSNEC |
| Moreira & Guarim-Neto 2009 | Cerrado | *Zanthoxylum rhoifolium* Lam. | Rutaceae | T | N | N | Hemorrhoids | DCS |
| Negrelle & Fornazzari 2007 | Atlantic Forest | *Achillea millefolium* L. | Asteraceae | H | E | E | Colic | SSNEC |
| Negrelle & Fornazzari 2007 | Atlantic Forest | *Achillea millefolium* L. | Asteraceae | H | E | E | Colic | SSNEC |
| Negrelle & Fornazzari 2007 | Atlantic Forest | *Achillea millefolium* L. | Asteraceae | H | E | E | Colic | SSNEC |
| Negrelle & Fornazzari 2007 | Atlantic Forest | *Achillea millefolium* L. | Asteraceae | H | E | E | Colic | SSNEC |
| Negrelle & Fornazzari 2007 | Atlantic Forest | *Achillea millefolium* L. | Asteraceae | H | E | E | Improves digestion | DDS |
| Negrelle & Fornazzari 2007 | Atlantic Forest | *Achillea millefolium* L. | Asteraceae | H | E | E | Improves digestion | DDS |
| Negrelle & Fornazzari 2007 | Atlantic Forest | *Achillea millefolium* L. | Asteraceae | H | E | E | Improves digestion | DDS |
| Negrelle & Fornazzari 2007 | Atlantic Forest | *Achillea millefolium* L. | Asteraceae | H | E | E | Improves digestion | DDS |
| Negrelle & Fornazzari 2007 | Atlantic Forest | *Achillea millefolium* L. | Asteraceae | H | E | E | Pain | SSNEC |
| Negrelle & Fornazzari 2007 | Atlantic Forest | *Achillea millefolium* L. | Asteraceae | H | E | E | Pain | SSNEC |
| Negrelle & Fornazzari 2007 | Atlantic Forest | *Achillea millefolium* L. | Asteraceae | H | E | E | Pain | SSNEC |
| Negrelle & Fornazzari 2007 | Atlantic Forest | *Achillea millefolium* L. | Asteraceae | H | E | E | Pain | SSNEC |
| Negrelle & Fornazzari 2007 | Atlantic Forest | *Achillea millefolium* L. | Asteraceae | H | E | E | Poor digestion | DDS |
| Negrelle & Fornazzari 2007 | Atlantic Forest | *Achillea millefolium* L. | Asteraceae | H | E | E | Poor digestion | DDS |
| Negrelle & Fornazzari 2007 | Atlantic Forest | *Achillea millefolium* L. | Asteraceae | H | E | E | Poor digestion | DDS |
| Negrelle & Fornazzari 2007 | Atlantic Forest | *Achillea millefolium* L. | Asteraceae | H | E | E | Poor digestion | DDS |
| Negrelle & Fornazzari 2007 | Atlantic Forest | *Achyrocline satureioides* (Lam.) DC. | Asteraceae | S | N | N | Fever | SSNEC |
| Negrelle & Fornazzari 2007 | Atlantic Forest | *Achyrocline satureioides* (Lam.) DC. | Asteraceae | S | N | N | Improves digestion | DDS |
| Negrelle & Fornazzari 2007 | Atlantic Forest | *Ageratum conyzoides* L. | Asteraceae | H | N | N | Influenza | DRS |
| Negrelle & Fornazzari 2007 | Atlantic Forest | *Allium sativum* L. | Alliaceae | H | E | E | Bronchitis | DRS |
| Negrelle & Fornazzari 2007 | Atlantic Forest | *Allium sativum* L. | Alliaceae | H | E | E | Helminthiasis | CIPD |
| Negrelle & Fornazzari 2007 | Atlantic Forest | *Allium sativum* L. | Alliaceae | H | E | E | Influenza | DRS |
| Negrelle & Fornazzari 2007 | Atlantic Forest | *Aloe succotrina* Lam. | Xanthorrhoeaceae | S | E | E | Heartburn | DGS |
| Negrelle & Fornazzari 2007 | Atlantic Forest | *Aloe succotrina* Lam. | Xanthorrhoeaceae | S | E | E | Improves digestion | DDS |
| Negrelle & Fornazzari 2007 | Atlantic Forest | *Aloe succotrina* Lam. | Xanthorrhoeaceae | S | E | E | Rheumatism | DMS |
| Negrelle & Fornazzari 2007 | Atlantic Forest | *Aloe succotrina* Lam. | Xanthorrhoeaceae | S | E | E | Ulcer | DDS |
| Negrelle & Fornazzari 2007 | Atlantic Forest | *Aloe succotrina* Lam. | Xanthorrhoeaceae | S | E | E | Wound | IPEC |
| Negrelle & Fornazzari 2007 | Atlantic Forest | *Ambrosia artemisiifolia* L. | Asteraceae | H | N | N | Amenorrhea | DGS |
| Negrelle & Fornazzari 2007 | Atlantic Forest | *Ambrosia artemisiifolia* L. | Asteraceae | H | N | N | Cholagogue | DDS |
| Negrelle & Fornazzari 2007 | Atlantic Forest | *Ambrosia artemisiifolia* L. | Asteraceae | H | N | N | Helminthiasis | CIPD |
| Negrelle & Fornazzari 2007 | Atlantic Forest | *Araucaria angustifolia* (Bertol.) Kuntze | Araucariaceae | T | N | N | Rheumatism | DMS |
| Negrelle & Fornazzari 2007 | Atlantic Forest | *Artemisia absinthium* L. | Asteraceae | H | E | E | Helminthiasis | CIPD |
| Negrelle & Fornazzari 2007 | Atlantic Forest | *Artemisia absinthium* L. | Asteraceae | H | E | E | Improves digestion | DDS |
| Negrelle & Fornazzari 2007 | Atlantic Forest | *Artemisia absinthium* L. | Asteraceae | H | E | E | Pain | SSNEC |
| Negrelle & Fornazzari 2007 | Atlantic Forest | *Artemisia absinthium* L. | Asteraceae | H | E | E | Poor digestion | DDS |
| Negrelle & Fornazzari 2007 | Atlantic Forest | *Atropa belladonna* L. | Solanaceae | H | E | E | Calming | MBD |
| Negrelle & Fornazzari 2007 | Atlantic Forest | *Atropa belladonna* L. | Solanaceae | H | E | E | Cardiotonic | DCS |
| Negrelle & Fornazzari 2007 | Atlantic Forest | *Baccharis crispa* Spreng. | Asteraceae | H | N | N | Diuretic | SSNEC |
| Negrelle & Fornazzari 2007 | Atlantic Forest | *Baccharis crispa* Spreng. | Asteraceae | H | N | N | Improves digestion | DDS |
| Negrelle & Fornazzari 2007 | Atlantic Forest | *Baccharis crispa* Spreng. | Asteraceae | H | N | N | Rheumatism | DMS |
| Negrelle & Fornazzari 2007 | Atlantic Forest | *Bauhinia forficata* Link | Fabaceae | T | N | N | Diuretic | SSNEC |
| Negrelle & Fornazzari 2007 | Atlantic Forest | *Bauhinia forficata* Link | Fabaceae | T | N | N | Hypoglycemic agent | SSNEC |
| Negrelle & Fornazzari 2007 | Atlantic Forest | *Bauhinia forficata* Link | Fabaceae | T | N | N | Pain | SSNEC |
| Negrelle & Fornazzari 2007 | Atlantic Forest | *Bauhinia forficata* Link | Fabaceae | T | N | N | Rheumatism | DMS |
| Negrelle & Fornazzari 2007 | Atlantic Forest | *Bidens pilosa* L. | Asteraceae | H | E | N | Hepatoprotector | DDS |
| Negrelle & Fornazzari 2007 | Atlantic Forest | *Brassica oleracea* L. | Brassicaceae | H | E | E | Heartburn | DGS |
| Negrelle & Fornazzari 2007 | Atlantic Forest | *Brassica oleracea* L. | Brassicaceae | H | E | E | Nutraceutical | ENMD |
| Negrelle & Fornazzari 2007 | Atlantic Forest | *Brassica oleracea* L. | Brassicaceae | H | E | E | Ulcer | DDS |
| Negrelle & Fornazzari 2007 | Atlantic Forest | *Cecropia glaziovii* Snethl. | Urticaceae | T | N | N | High blood pressure | DCS |
| Negrelle & Fornazzari 2007 | Atlantic Forest | *Cedrela fissilis* Vell. | Meliaceae | T | N | N | Undefined | SSNEC |
| Negrelle & Fornazzari 2007 | Atlantic Forest | *Chenopodium ambrosioides* L. | Amaranthaceae | H | N | N | Helminthiasis | CIPD |
| Negrelle & Fornazzari 2007 | Atlantic Forest | *Chenopodium ambrosioides* L. | Amaranthaceae | H | N | N | Helminthiasis | CIPD |
| Negrelle & Fornazzari 2007 | Atlantic Forest | *Chenopodium ambrosioides* L. | Amaranthaceae | H | N | N | Improves digestion | DDS |
| Negrelle & Fornazzari 2007 | Atlantic Forest | *Chenopodium ambrosioides* L. | Amaranthaceae | H | N | N | Improves digestion | DDS |
| Negrelle & Fornazzari 2007 | Atlantic Forest | *Chenopodium ambrosioides* L. | Amaranthaceae | H | N | N | Pain | SSNEC |
| Negrelle & Fornazzari 2007 | Atlantic Forest | *Chenopodium ambrosioides* L. | Amaranthaceae | H | N | N | Pain | SSNEC |
| Negrelle & Fornazzari 2007 | Atlantic Forest | *Chenopodium ambrosioides* L. | Amaranthaceae | H | N | N | Undefined | SSNEC |
| Negrelle & Fornazzari 2007 | Atlantic Forest | *Chenopodium ambrosioides* L. | Amaranthaceae | H | N | N | Undefined | SSNEC |
| Negrelle & Fornazzari 2007 | Atlantic Forest | *Cinchona officinalis* L. | Rubiaceae | H | E | E | Dysentry | CIPD |
| Negrelle & Fornazzari 2007 | Atlantic Forest | *Cinchona officinalis* L. | Rubiaceae | H | E | E | Improves digestion | DDS |
| Negrelle & Fornazzari 2007 | Atlantic Forest | *Cinchona officinalis* L. | Rubiaceae | H | E | E | Pain | SSNEC |
| Negrelle & Fornazzari 2007 | Atlantic Forest | *Citrus aurantium* L. | Rutaceae | T | E | E | Abortifacient | PCP |
| Negrelle & Fornazzari 2007 | Atlantic Forest | *Citrus aurantium* L. | Rutaceae | T | E | E | Colic | SSNEC |
| Negrelle & Fornazzari 2007 | Atlantic Forest | *Citrus limon* (L.) Osbeck | Rutaceae | T | E | E | Influenza | DRS |
| Negrelle & Fornazzari 2007 | Atlantic Forest | *Citrus limon* (L.) Osbeck | Rutaceae | T | E | E | Influenza | DRS |
| Negrelle & Fornazzari 2007 | Atlantic Forest | *Citrus limon* (L.) Osbeck | Rutaceae | T | E | E | Nutraceutical | ENMD |
| Negrelle & Fornazzari 2007 | Atlantic Forest | *Citrus limon* (L.) Osbeck | Rutaceae | T | E | E | Nutraceutical | ENMD |
| Negrelle & Fornazzari 2007 | Atlantic Forest | *Citrus sinensis* (L.) Osbeck | Rutaceae | T | E | E | Calming | MBD |
| Negrelle & Fornazzari 2007 | Atlantic Forest | *Citrus sinensis* (L.) Osbeck | Rutaceae | T | E | E | Influenza | DRS |
| Negrelle & Fornazzari 2007 | Atlantic Forest | *Citrus sinensis* (L.) Osbeck | Rutaceae | T | E | E | Measles | CIPD |
| Negrelle & Fornazzari 2007 | Atlantic Forest | *Citrus sinensis* (L.) Osbeck | Rutaceae | T | E | E | Mumps | CIPD |
| Negrelle & Fornazzari 2007 | Atlantic Forest | *Citrus sinensis* (L.) Osbeck | Rutaceae | T | E | E | Rubella | CIPD |
| Negrelle & Fornazzari 2007 | Atlantic Forest | *Corymbia citrioPaina (Hook.) K. D. Hill & L. A. S. Johnson* | Myrtaceae | T | E | E | Hoarseness | DRS |
| Negrelle & Fornazzari 2007 | Atlantic Forest | *Corymbia citrioPaina (Hook.) K. D. Hill & L. A. S. Johnson* | Myrtaceae | T | E | E | Influenza | DRS |
| Negrelle & Fornazzari 2007 | Atlantic Forest | *Corymbia citrioPaina (Hook.) K. D. Hill & L. A. S. Johnson* | Myrtaceae | T | E | E | Sinusitis | DRS |
| Negrelle & Fornazzari 2007 | Atlantic Forest | *Cunila microcephala* Benth. | Lamiaceae | H | N | N | Bronchitis | DRS |
| Negrelle & Fornazzari 2007 | Atlantic Forest | *Cunila microcephala* Benth. | Lamiaceae | H | N | N | Bronchitis | DRS |
| Negrelle & Fornazzari 2007 | Atlantic Forest | *Cunila microcephala* Benth. | Lamiaceae | H | N | N | Cough | SSNEC |
| Negrelle & Fornazzari 2007 | Atlantic Forest | *Cunila microcephala* Benth. | Lamiaceae | H | N | N | Cough | SSNEC |
| Negrelle & Fornazzari 2007 | Atlantic Forest | *Cunila microcephala* Benth. | Lamiaceae | H | N | N | Influenza | DRS |
| Negrelle & Fornazzari 2007 | Atlantic Forest | *Cunila microcephala* Benth. | Lamiaceae | H | N | N | Influenza | DRS |
| Negrelle & Fornazzari 2007 | Atlantic Forest | *Cunila microcephala* Benth. | Lamiaceae | H | N | N | Pain | SSNEC |
| Negrelle & Fornazzari 2007 | Atlantic Forest | *Cunila microcephala* Benth. | Lamiaceae | H | N | N | Pain | SSNEC |
| Negrelle & Fornazzari 2007 | Atlantic Forest | *Cuphea calophylla* Cham. & Schltdl. | Lythraceae | H | N | N | Depurative | DBBO |
| Negrelle & Fornazzari 2007 | Atlantic Forest | *Cuphea calophylla* Cham. & Schltdl. | Lythraceae | H | N | N | Depurative | DBBO |
| Negrelle & Fornazzari 2007 | Atlantic Forest | *Cuphea calophylla* Cham. & Schltdl. | Lythraceae | H | N | N | Hypoglycemic agent | SSNEC |
| Negrelle & Fornazzari 2007 | Atlantic Forest | *Cuphea calophylla* Cham. & Schltdl. | Lythraceae | H | N | N | Hypoglycemic agent | SSNEC |
| Negrelle & Fornazzari 2007 | Atlantic Forest | *Cuphea calophylla* Cham. & Schltdl. | Lythraceae | H | N | N | Inflammation | SSNEC |
| Negrelle & Fornazzari 2007 | Atlantic Forest | *Cuphea calophylla* Cham. & Schltdl. | Lythraceae | H | N | N | Inflammation | SSNEC |
| Negrelle & Fornazzari 2007 | Atlantic Forest | *Cuphea calophylla* Cham. & Schltdl. | Lythraceae | H | N | N | Rheumatism | DMS |
| Negrelle & Fornazzari 2007 | Atlantic Forest | *Cuphea calophylla* Cham. & Schltdl. | Lythraceae | H | N | N | Rheumatism | DMS |
| Negrelle & Fornazzari 2007 | Atlantic Forest | *Cymbopogon citratus* (DC.) Stapf | Poaceae | H | E | E | Calming | MBD |
| Negrelle & Fornazzari 2007 | Atlantic Forest | *Cymbopogon citratus* (DC.) Stapf | Poaceae | H | E | E | High blood pressure | DCS |
| Negrelle & Fornazzari 2007 | Atlantic Forest | *Cymbopogon citratus* (DC.) Stapf | Poaceae | H | E | E | Improves digestion | DDS |
| Negrelle & Fornazzari 2007 | Atlantic Forest | *Cymbopogon citratus* (DC.) Stapf | Poaceae | H | E | E | Low blood pressure | DCS |
| Negrelle & Fornazzari 2007 | Atlantic Forest | *Cymbopogon citratus* (DC.) Stapf | Poaceae | H | E | E | Nutraceutical | ENMD |
| Negrelle & Fornazzari 2007 | Atlantic Forest | *Cymbopogon citratus* (DC.) Stapf | Poaceae | H | E | E | Pain | SSNEC |
| Negrelle & Fornazzari 2007 | Atlantic Forest | *Cymbopogon citratus* (DC.) Stapf | Poaceae | H | E | E | Somnific | DNS |
| Negrelle & Fornazzari 2007 | Atlantic Forest | *Cynara cardunculus* L. | Asteraceae | H | E | E | Depurative | DBBO |
| Negrelle & Fornazzari 2007 | Atlantic Forest | *Cynara cardunculus* L. | Asteraceae | H | E | E | Hypoglycemic agent | SSNEC |
| Negrelle & Fornazzari 2007 | Atlantic Forest | *Cynara cardunculus* L. | Asteraceae | H | E | E | Improves digestion | DDS |
| Negrelle & Fornazzari 2007 | Atlantic Forest | *Cynara cardunculus* L. | Asteraceae | H | E | E | Weight reduction | ENMD |
| Negrelle & Fornazzari 2007 | Atlantic Forest | *Desmodium adscendens* (Sw.) DC. | Fabaceae | H | N | N | Inflammation | SSNEC |
| Negrelle & Fornazzari 2007 | Atlantic Forest | *EchinoPainus grandiflorus (Cham. & Schltr.) Micheli* | Alismataceae | H | N | N | Cholagogue | DDS |
| Negrelle & Fornazzari 2007 | Atlantic Forest | *EchinoPainus grandiflorus (Cham. & Schltr.) Micheli* | Alismataceae | H | N | N | Diuretic | SSNEC |
| Negrelle & Fornazzari 2007 | Atlantic Forest | *EchinoPainus grandiflorus (Cham. & Schltr.) Micheli* | Alismataceae | H | N | N | Improves digestion | DDS |
| Negrelle & Fornazzari 2007 | Atlantic Forest | *EchinoPainus grandiflorus (Cham. & Schltr.) Micheli* | Alismataceae | H | N | N | Pain | SSNEC |
| Negrelle & Fornazzari 2007 | Atlantic Forest | *Eriobotrya japonica* (Thunb.) Lindl. | Rosaceae | T | E | E | Cough | SSNEC |
| Negrelle & Fornazzari 2007 | Atlantic Forest | *Eriobotrya japonica* (Thunb.) Lindl. | Rosaceae | T | E | E | Inflammation | SSNEC |
| Negrelle & Fornazzari 2007 | Atlantic Forest | *Foeniculum vulgare* Mill. | Apiaceae | H | E | E | Colic | SSNEC |
| Negrelle & Fornazzari 2007 | Atlantic Forest | *Foeniculum vulgare* Mill. | Apiaceae | H | E | E | Colic | SSNEC |
| Negrelle & Fornazzari 2007 | Atlantic Forest | *Foeniculum vulgare* Mill. | Apiaceae | H | E | E | Colic | SSNEC |
| Negrelle & Fornazzari 2007 | Atlantic Forest | *Foeniculum vulgare* Mill. | Apiaceae | H | E | E | Heartburn | DGS |
| Negrelle & Fornazzari 2007 | Atlantic Forest | *Gymnanthemum amygdalinum* (Delile) Sch.Bip. ex Walp. | Asteraceae | S | N | N | Cholagogue | DDS |
| Negrelle & Fornazzari 2007 | Atlantic Forest | *Gymnanthemum amygdalinum* (Delile) Sch.Bip. ex Walp. | Asteraceae | S | N | N | Improves digestion | DDS |
| Negrelle & Fornazzari 2007 | Atlantic Forest | *Handroanthus impetiginosus* Mattos | Bignoniaceae | T | N | N | Antimicrobial | CIPD |
| Negrelle & Fornazzari 2007 | Atlantic Forest | *Handroanthus impetiginosus* Mattos | Bignoniaceae | T | N | N | Cancer | NEO |
| Negrelle & Fornazzari 2007 | Atlantic Forest | *Handroanthus impetiginosus* Mattos | Bignoniaceae | T | N | N | Inflammation | SSNEC |
| Negrelle & Fornazzari 2007 | Atlantic Forest | *Juglans regia* L. | Juglandaceae | T | E | E | Cardiotonic | DCS |
| Negrelle & Fornazzari 2007 | Atlantic Forest | *Juglans regia* L. | Juglandaceae | T | E | E | Depurative | DBBO |
| Negrelle & Fornazzari 2007 | Atlantic Forest | *Lavandula angustifolia* Mill. | Lamiaceae | H | E | E | Low blood pressure | DCS |
| Negrelle & Fornazzari 2007 | Atlantic Forest | *Leonurus japonicus* Houtt. | Lamiaceae | H | E | E | Cholagogue | DDS |
| Negrelle & Fornazzari 2007 | Atlantic Forest | *Lepidium sativum* L. | Brassicaceae | H | E | E | Bronchitis | DRS |
| Negrelle & Fornazzari 2007 | Atlantic Forest | *Lepidium sativum* L. | Brassicaceae | H | E | E | Diuretic | SSNEC |
| Negrelle & Fornazzari 2007 | Atlantic Forest | *Lepidium sativum* L. | Brassicaceae | H | E | E | Emollient | DSST |
| Negrelle & Fornazzari 2007 | Atlantic Forest | *Lepidium sativum* L. | Brassicaceae | H | E | E | Nutraceutical | ENMD |
| Negrelle & Fornazzari 2007 | Atlantic Forest | *Lepidium sativum* L. | Brassicaceae | H | E | E | Rheumatism | DMS |
| Negrelle & Fornazzari 2007 | Atlantic Forest | *Malva sylvestris* L. | Malvaceae | H | E | E | Antimicrobial | CIPD |
| Negrelle & Fornazzari 2007 | Atlantic Forest | *Malva sylvestris* L. | Malvaceae | H | E | E | Inflammation | SSNEC |
| Negrelle & Fornazzari 2007 | Atlantic Forest | *Malva sylvestris* L. | Malvaceae | H | E | E | Wound | IPEC |
| Negrelle & Fornazzari 2007 | Atlantic Forest | *Maranta arundinacea* L. | Marantaceae | H | E | E | Wound | IPEC |
| Negrelle & Fornazzari 2007 | Atlantic Forest | *Matricaria chamomilla* L. | Asteraceae | H | E | E | Amenorrhea | DGS |
| Negrelle & Fornazzari 2007 | Atlantic Forest | *Matricaria chamomilla* L. | Asteraceae | H | E | E | Colic | SSNEC |
| Negrelle & Fornazzari 2007 | Atlantic Forest | *Matricaria chamomilla* L. | Asteraceae | H | E | E | Improves digestion | DDS |
| Negrelle & Fornazzari 2007 | Atlantic Forest | *Matricaria chamomilla* L. | Asteraceae | H | E | E | Inflammation | SSNEC |
| Negrelle & Fornazzari 2007 | Atlantic Forest | *Maytenus ilicifolia* Mart. ex Reissek | Celastraceae | S | N | N | Depurative | DBBO |
| Negrelle & Fornazzari 2007 | Atlantic Forest | *Maytenus ilicifolia* Mart. ex Reissek | Celastraceae | S | N | N | Dermatitis | DSST |
| Negrelle & Fornazzari 2007 | Atlantic Forest | *Maytenus ilicifolia* Mart. ex Reissek | Celastraceae | S | N | N | Diuretic | SSNEC |
| Negrelle & Fornazzari 2007 | Atlantic Forest | *Maytenus ilicifolia* Mart. ex Reissek | Celastraceae | S | N | N | High blood pressure | DCS |
| Negrelle & Fornazzari 2007 | Atlantic Forest | *Melissa officinalis* L. | Lamiaceae | H | E | E | Calming | MBD |
| Negrelle & Fornazzari 2007 | Atlantic Forest | *Melissa officinalis* L. | Lamiaceae | H | E | E | Cholagogue | DDS |
| Negrelle & Fornazzari 2007 | Atlantic Forest | *Melissa officinalis* L. | Lamiaceae | H | E | E | High blood pressure | DCS |
| Negrelle & Fornazzari 2007 | Atlantic Forest | *Melissa officinalis* L. | Lamiaceae | H | E | E | Improves digestion | DDS |
| Negrelle & Fornazzari 2007 | Atlantic Forest | *Melissa officinalis* L. | Lamiaceae | H | E | E | Low blood pressure | DCS |
| Negrelle & Fornazzari 2007 | Atlantic Forest | *Mentha × piperita* L. | Lamiaceae | H | E | E | Aphrodisiac | DGS |
| Negrelle & Fornazzari 2007 | Atlantic Forest | *Mentha × piperita* L. | Lamiaceae | H | E | E | Helminthiasis | CIPD |
| Negrelle & Fornazzari 2007 | Atlantic Forest | *Mentha × piperita* L. | Lamiaceae | H | E | E | Improves digestion | DDS |
| Negrelle & Fornazzari 2007 | Atlantic Forest | *Mentha × piperita* L. | Lamiaceae | H | E | E | Pain | SSNEC |
| Negrelle & Fornazzari 2007 | Atlantic Forest | *Mentha × piperita* L. | Lamiaceae | H | E | E | Undefined | SSNEC |
| Negrelle & Fornazzari 2007 | Atlantic Forest | *Muehlenbeckia platyclada* (F. Muell.) Meisn. | Polygonaceae | S | E | E | Pain | SSNEC |
| Negrelle & Fornazzari 2007 | Atlantic Forest | *Nasturtium officinale* W. T. Aiton | Brassicaceae | H | E | E | Emollient | DSST |
| Negrelle & Fornazzari 2007 | Atlantic Forest | *Nasturtium officinale* W. T. Aiton | Brassicaceae | H | E | E | Nutraceutical | ENMD |
| Negrelle & Fornazzari 2007 | Atlantic Forest | *Ocimum basilicum* L. | Lamiaceae | H | E | E | Calming | MBD |
| Negrelle & Fornazzari 2007 | Atlantic Forest | *Ocimum basilicum* L. | Lamiaceae | H | E | E | Colic | SSNEC |
| Negrelle & Fornazzari 2007 | Atlantic Forest | *Ocimum basilicum* L. | Lamiaceae | H | E | E | Dermatitis | DSST |
| Negrelle & Fornazzari 2007 | Atlantic Forest | *Ocimum basilicum* L. | Lamiaceae | H | E | E | Dysentry | CIPD |
| Negrelle & Fornazzari 2007 | Atlantic Forest | *Ocimum basilicum* L. | Lamiaceae | H | E | E | Hoarseness | DRS |
| Negrelle & Fornazzari 2007 | Atlantic Forest | *Ocimum basilicum* L. | Lamiaceae | H | E | E | Nutraceutical | ENMD |
| Negrelle & Fornazzari 2007 | Atlantic Forest | *Ocimum basilicum* L. | Lamiaceae | H | E | E | Pain | SSNEC |
| Negrelle & Fornazzari 2007 | Atlantic Forest | *Pelargonium × hortorum* L. H. Bailey | Geraniaceae | H | E | E | Inflammation | SSNEC |
| Negrelle & Fornazzari 2007 | Atlantic Forest | *Persea americana* Mill. | Lauraceae | T | E | E | Diuretic | SSNEC |
| Negrelle & Fornazzari 2007 | Atlantic Forest | *Persea americana* Mill. | Lauraceae | T | E | E | Hypoglycemic agent | SSNEC |
| Negrelle & Fornazzari 2007 | Atlantic Forest | *Persea major* (Meisn.) L.E.Kopp | Lamiaceae | T | N | N | Hypoglycemic agent | SSNEC |
| Negrelle & Fornazzari 2007 | Atlantic Forest | *Petiveria alliacea* L. | Phytolaccaceae | H | E | E | Dermatitis | DSST |
| Negrelle & Fornazzari 2007 | Atlantic Forest | *Petiveria alliacea* L. | Phytolaccaceae | H | E | E | Dermatitis | DSST |
| Negrelle & Fornazzari 2007 | Atlantic Forest | *Petiveria alliacea* L. | Phytolaccaceae | H | E | E | Dermatitis | DSST |
| Negrelle & Fornazzari 2007 | Atlantic Forest | *Petiveria alliacea* L. | Phytolaccaceae | H | E | E | Pain | SSNEC |
| Negrelle & Fornazzari 2007 | Atlantic Forest | *Petiveria alliacea* L. | Phytolaccaceae | H | E | E | Pain | SSNEC |
| Negrelle & Fornazzari 2007 | Atlantic Forest | *Petiveria alliacea* L. | Phytolaccaceae | H | E | E | Pain | SSNEC |
| Negrelle & Fornazzari 2007 | Atlantic Forest | *Petroselinum crispum* (Mill.) Fuss | Apiaceae | H | E | E | Diuretic | SSNEC |
| Negrelle & Fornazzari 2007 | Atlantic Forest | *Phyllanthus niruri* L. | Phyllanthaceae | H | N | N | Diuretic | SSNEC |
| Negrelle & Fornazzari 2007 | Atlantic Forest | *Plectranthus barbatus* Andr. | Lamiaceae | H | E | E | Cholagogue | DDS |
| Negrelle & Fornazzari 2007 | Atlantic Forest | *Plectranthus barbatus* Andr. | Lamiaceae | H | E | E | Cough | SSNEC |
| Negrelle & Fornazzari 2007 | Atlantic Forest | *Plectranthus barbatus* Andr. | Lamiaceae | H | E | E | Improves digestion | DDS |
| Negrelle & Fornazzari 2007 | Atlantic Forest | *Plectranthus barbatus* Andr. | Lamiaceae | H | E | E | Pain | SSNEC |
| Negrelle & Fornazzari 2007 | Atlantic Forest | *Polygala paniculata* L. | Polygalaceae | H | N | N | Emollient | DSST |
| Negrelle & Fornazzari 2007 | Atlantic Forest | *Polygala paniculata* L. | Polygalaceae | H | N | N | Emollient | DSST |
| Negrelle & Fornazzari 2007 | Atlantic Forest | *Polygala paniculata* L. | Polygalaceae | H | N | N | Influenza | DRS |
| Negrelle & Fornazzari 2007 | Atlantic Forest | *Polygala paniculata* L. | Polygalaceae | H | N | N | Influenza | DRS |
| Negrelle & Fornazzari 2007 | Atlantic Forest | *Polygala paniculata* L. | Polygalaceae | H | N | N | Pain | SSNEC |
| Negrelle & Fornazzari 2007 | Atlantic Forest | *Polygala paniculata* L. | Polygalaceae | H | N | N | Pain | SSNEC |
| Negrelle & Fornazzari 2007 | Atlantic Forest | *Polygonum punctatum* Elliott | Polygonaceae | H | N | N | Hemorrhoids | DCS |
| Negrelle & Fornazzari 2007 | Atlantic Forest | *Polygonum punctatum* Elliott | Polygonaceae | H | N | N | Influenza | DRS |
| Negrelle & Fornazzari 2007 | Atlantic Forest | *Protium kleinii* Cuatrec. | Burseraceae | T | N | N | Emollient | DSST |
| Negrelle & Fornazzari 2007 | Atlantic Forest | *Psidium guajava* L. | Myrtaceae | S | E | E | Dysentry | CIPD |
| Negrelle & Fornazzari 2007 | Atlantic Forest | *Punica granatum* L. | Lythraceae | T | E | E | Dysentry | CIPD |
| Negrelle & Fornazzari 2007 | Atlantic Forest | *Renealmia brasiliensis* K.Schum. | Zingiberaceae | H | N | N | Helminthiasis | CIPD |
| Negrelle & Fornazzari 2007 | Atlantic Forest | *Renealmia brasiliensis* K.Schum. | Zingiberaceae | H | N | N | Helminthiasis | CIPD |
[truncated: 425,564 more chars]
